# Immunohistochemical Typing of Adenocarcinomas of the Pancreatobiliary System Improves Diagnosis and Prognostic Stratification

**DOI:** 10.1371/journal.pone.0166067

**Published:** 2016-11-09

**Authors:** Carlos Fernández Moro, Alejandro Fernandez-Woodbridge, Melroy Alistair D'souza, Qianni Zhang, Benedek Bozoky, Senthil Vasan Kandaswamy, Piera Catalano, Rainer Heuchel, Sonia Shtembari, Marco Del Chiaro, Olof Danielsson, Mikael Björnstedt, J. Matthias Löhr, Bengt Isaksson, Caroline Verbeke, Béla Bozóky

**Affiliations:** 1 Department of Laboratory Medicine (LABMED) Division of Pathology, Karolinska Institute, Stockholm, Sweden; 2 Department of Clinical Pathology/Cytology, Karolinska University Hospital, Stockholm, Sweden; 3 Department of Oncology-Pathology, Science for Life Laboratory, Karolinska Institute, Stockholm, Sweden; 4 Department of Clinical Science Intervention and Technology (CLINTEC), Karolinska Institute, Stockholm, Sweden; 5 Center for Digestive Diseases, Karolinska University Hospital, Stockholm, Sweden; 6 School of Electronic Engineering and Computer Science, Queen Mary University of London, London, United Kingdom; 7 Department of Medical Epidemiology and Biostatistics (MEB), Karolinska Institute, Stockholm, Sweden; 8 Service of Pathology, Ospedale Fatebenefratelli "S. Giovanni Calibita", Rome, Italy; Centro Nacional de Investigaciones Oncologicas, SPAIN

## Abstract

**Background & Aims:**

Adenocarcinomas of the pancreatobiliary system are currently classified by their primary anatomical location. In particular, the pathological diagnosis of intrahepatic cholangiocarcinoma is still considered as a diagnosis of exclusion of metastatic adenocarcinoma. Periampullary cancers have been previously classified according to the histological type of differentiation (pancreatobiliary, intestinal), but overlapping morphological features hinder their differential diagnosis. We performed an integrative immunohistochemical analysis of pancreato-biliary tumors to improve their diagnosis and prediction of outcome.

**Methods:**

This was a retrospective observational cohort study on patients with adenocarcinoma of the pancreatobiliary system who underwent diagnostic core needle biopsy or surgical resection at a tertiary referral center. 409 tumor samples were analyzed with up to 27 conventional antibodies used in diagnostic pathology. Immunohistochemical scoring system was the percentage of stained tumor cells. Bioinformatic analysis, internal validation, and survival analysis were performed.

**Results:**

Hierarchical clustering and differential expression analysis identified three immunohistochemical tumor types (extrahepatic pancreatobiliary, intestinal, and intrahepatic cholangiocarcinoma) and the discriminant markers between them. Among patients who underwent surgical resection of their primary tumor with curative intent, the intestinal type showed an adjusted hazard ratio of 0.19 for overall survival (95% confidence interval 0.05–0.72; p value = 0.014) compared to the extrahepatic pancreatobiliary type.

**Conclusions:**

Integrative immunohistochemical classification of adenocarcinomas of the pancreatobiliary system results in a characteristic immunohistochemical profile for intrahepatic cholangiocarcinoma and intestinal type adenocarcinoma, which helps in distinguishing them from metastatic and pancreatobiliary type adenocarcinoma, respectively. A diagnostic immunohistochemical panel and additional extended panels of discriminant markers are proposed as guidance for their pathological diagnosis.

## Introduction

Adenocarcinomas of the pancreatobiliary system are among the most lethal cancers. With a dismal 5-year survival of 6%[1], ductal pancreatic adenocarcinoma ranks currently as the fourth leading cause of cancer death in the United States and Europe[2]. It is a medical emergency[3]. Similarly, an increasing incidence rate is also reported for intrahepatic cholangiocarcinoma, which is the second most common type of liver cancer[4].

Currently, tumors of the pancreatobiliary system are classified and staged based on the anatomical site of origin (AJCC, UICC-TNM 7^th^ edition)[5,6], as ampullary carcinoma, ductal pancreatic adenocarcinoma, distal bile duct cancer, gallbladder carcinoma, perihilar cholangiocarcinoma, and intrahepatic cholangiocarcinoma.

In particular, the pathological diagnosis of intrahepatic cholangiocarcinoma can be challenging, e.g. in case of radiologic detection of tumor nodule(s) in the liver. Reliable pathological diagnostic criteria are currently lacking; indeed, according to current standards, the diagnosis of intrahepatic cholangiocarcinoma is reached by exclusion of metastatic adenocarcinoma[7] and immunohistochemistry has previously not been considered as contributory to a positive diagnosis[8].

The traditional concept of adenocarcinomas of the pancreatobiliary system is that of a single group of tumors with an overall poor prognosis. However, studying these tumors for possible phenotypic diversity may reveal differences of potential prognostic and therapeutic relevance. Attempts at classifying periampullary carcinomas have already introduced two histological types of differentiation: pancreatobiliary and intestinal[9,10]. Evidence supports that ampullary carcinomas of the intestinal type have a more favorable prognosis than those of the "classical" pancreatobiliary type. Consequently, a more “colorectal type” chemotherapy with the possibility of resection of liver metastasis has been considered[11].

In this study, we present a novel, integrative immunohistochemical classification of adenocarcinomas of the pancreatobiliary system that refines diagnosis and prediction of outcome and has potential therapeutic implications. In particular, the immunohistochemical diagnosis of intrahepatic cholangiocarcinoma and intestinal type adenocarcinoma will be addressed.

## Materials and Methods

### Patients and data

This was a retrospective observational cohort study. The series included patients with adenocarcinoma arising in the pancreatobiliary system who underwent diagnostic core needle biopsy or surgical resection with curative intent at the Karolinska University Hospital, Huddinge, Sweden, which is a tertiary referral center. Patients were diagnosed between years 2002–2013 and followed until August 2016. On histopathology tumors were staged according to the 7^th^ edition of the American Joint Committee on Cancer (AJCC)–Union for International Cancer Control (UICC) tumor node metastasis (TNM) classification[5,6]. The study included only adenocarcinomas and histological variants, as defined by the World Health Organization (WHO) classification 2010[12]. Adenosquamous carcinomas were excluded from the series in order to avoid excessive study complexity. Tumor samples of hepatocellular carcinoma, a tumor type with a well-known immunohistochemical profile[13,14], were included as an internal control group for the semi-supervised evaluation of clustering results.

Data on patient demographics, diagnosis, surgical resection, and outcome were retrieved from the medical records in the hospital database.

The study was approved by the Regional Ethical Review Board, Stockholm (2015/259-31/2). Written or verbal informed consent from participant patients was not obtained specifically for this study, because i) all the diagnostic and therapeutic procedures in this study were already standard of care in routine clinical practice and did not require further specific permission from the patients; ii) only histopathological samples for which patients consented inclusion and storage in the biobank, according to biobank legislation, were included in the study; and iii) further specific written consent was considered as unfeasible due to the retrospective nature of the study, the long study period (2002–2016), and the general poor prognosis associated with the tumor types object of this study. The described procedure for inclusion in the study was approved by the Regional Ethical Review Board, Stockholm.

### Immunohistochemistry

For each diagnostic probe (resection specimen or biopsy), a representative block based on the hematoxylin-eosin stain was selected for immunohistochemistry. This was stained with a panel consisting of up to 38 antibodies, of which finally 27 (S1 Table) were considered for the analysis, after pruning of missing data. These were all conventional antibodies widely used in diagnostic pathology, including markers for intermediate filaments[15] (CK5, CK7, CK17, CK18, CK19, CK20, vimentin) and mucins[16] (MUC1, MUC2, MUC5AC, MUC6), which are commonly employed to differentiate between the different types (e.g. squamous, glandular) and subtypes (e.g. glandular intestinal and pancreatobiliary) of epithelia; markers usually expressed in adenocarcinomas of gastrointestinal and pancreatobiliary origin (BerEP4, EMA, monoclonal CEA[17], polyclonal CEA, CA19-9, CA125[18], maspin[19]); the intestinal transcription factor CDX2, tumor suppressor proteins[20] (p53, SMAD4), the proliferation marker Ki67, p63 for squamous cell differentiation, the neuroendocrine marker chromogranin A, CD10 for staining of the microvilli/brush-border, CD56 marker of cholangiolocellular[21] differentiation, and Wilms tumor protein WT1, whose expression has been described in a variety of tumor types[22].

The immunoreactivity was quantitatively assessed by participant pathologists (BB, CFM) by visual examination of the whole section and recorded using a continuous numeric score (from 1 to 100) based on the percentage of stained tumor cells[23,24]. Intensity of staining was only taken into account for scoring of p53, for which only moderate and strong immunoreactivity was considered positive. All immunohistochemical data were recorded in a database.

### Bioinformatics data analysis

**Fig 1:**
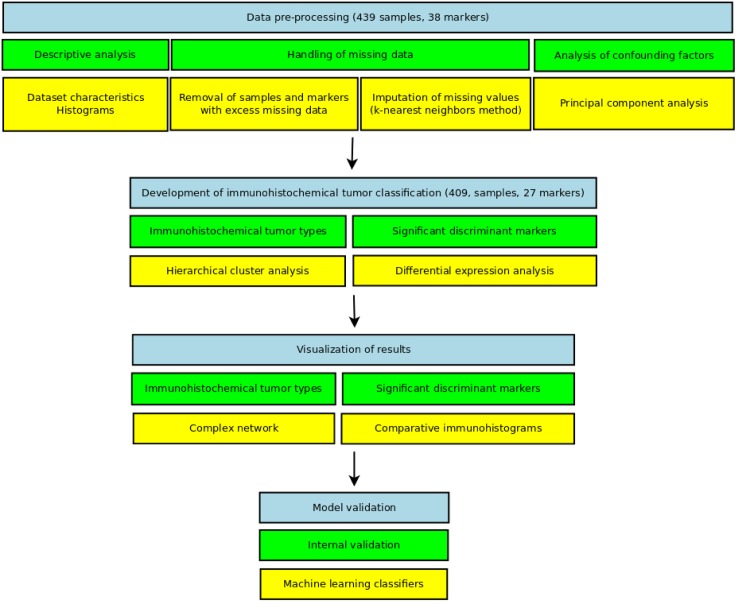
Schematic overview of the data analysis performed in this study.

The analysis of immunohistochemical data comprised four major steps (Fig 1): data pre-processing, cluster and differential expression analysis, graphical visualizations and internal model validation. The first three steps were mostly implemented using R[25] statistical computing software (version 3.0.2). Internal model validation was performed using Weka[26] data mining software (version 3.6.9). The complete dataset, code and reproducible computer environment for the analysis, including a Docker[27] image are provided on-line (http://dx.doi.org/10.5061/dryad.g8h71).

#### Data pre-processing

***Descriptive analysis*:** Dataset characteristics and histograms for immunohistochemical markers and anatomical diagnoses were generated.

***Pruning of missing data and imputation*:** In this order, antibody markers (out of the initial 38) with more than 40% missing values and tumor samples with more than 50% missing values were disregarded (S1 File). The remaining missing values were imputed using k-nearest neighbors method with Bioconductor package “impute”[28]. Subsequent analyses were carried out on the imputed dataset.

***Analysis of confounding factors*:** Principal component analysis was used to investigate the set of analyzed immunohistochemical markers and the type of probe (biopsy/resection) as potential confounding factors.

#### Cluster and differential expression analysis

Several hierarchical clusterings were generated using different distances and linkages. The ability to correctly separate known tumor types, such as hepatocellular carcinoma (the control group), was used as a guide for the evaluation of clustering results. Clustering with Pearson distance and average linkage yielded the most meaningful result according to the current state of knowledge on hepato-pancreato-biliary tumors. Its dendrogram was finally pruned to capture the clusters of interest that were identified by visual assessment, disregarding outliers. The clusters were regarded “of interest” using a semi-supervised approach, based on homogeneity of the diagnoses, distinct patterns of marker expression (according to heatmap bands), and greatest distance between clusters dominated by known tumor types with divergent immunohistochemical profiles (such as ductal pancreatic adenocarcinoma and hepatocellular carcinoma). The clusters of interest will be referred as the “immunohistochemical tumor types” throughout the remainder of this article.

Differential expression analysis was used to define pairwise the panels of statistically significant markers between the immunohistochemical tumor types. The clinically most relevant differential diagnoses were tested using three different non-parametric methods, RankProd[29], SAM[30], and MultTest[31]. To minimize the risk of false positivity, only those markers that consistently showed statistical significance by all three methods were considered as truly significant.

#### Graphical visualizations

We generated comparative immunohistograms to represent the differences in marker expression between the immunohistochemical tumor types, according to the results of differential expression analysis.

A complex network was also built using an alternative, robust clustering approach in which missing data were imputed with random values through 15000 iterations. The network visualization was generated in Gephi[32] (version 0.8.2-beta) using the force atlas2 layout algorithm.

#### Internal model validation

We tested the robustness of the resulting immunohistochemical classification by evaluating the performance of four machine learning classifiers (Bayes network, simple logic, sequential minimal optimization, and random tree) in correctly discriminating between the immunohistochemical tumor types along a suite of classification experiments. Several metrics were considered for evaluation: true positive rate, false positive rate, precision, recall, F-measure, and receiver operating characteristic (ROC) area.

#### Diagnostic immunohistochemical panel

A simplified diagnostic immunohistochemical panel was finally developed by summarizing in table format the most discriminant markers and their expected immunoreactivity.

### Survival analysis

We used Kaplan–Meier survival curves, with time to death censored at the end of follow-up (August 2016) as the outcome. Cox proportional hazards models were used to estimate the crude and adjusted hazard ratios and 95% confidence intervals for mortality associated with tumor type by the immunohistochemical and anatomy-based classifications. The adjusted models included pathological tumor stage (pT) and lymph node status (pN) as covariates. The distant metastasis pathological descriptor (pM) was not included, because it could not be assessed microscopically in the majority of cases (pMX) and preoperative detection of distant metastasis often precluded resection of the primary tumor. The Cox model proportional hazards assumption was tested. Survival analysis was performed using R statistical packages “survival”[33] and “rms”[34].

## Results

### Bioinformatic data analysis

#### Data pre-processing

***Descriptive analysis and handling of missing data*:** The raw dataset comprised 439 tumor samples and 38 immunohistochemical markers. Histograms for every marker and anatomy-based diagnosis showed discrete distributions of immunohistochemical scoring values. 36% of values in the raw dataset were missing. As this was a retrospective study on diagnostic tumor samples, the individual immunohistochemical panels were in first instance determined by the specific differential diagnoses in consideration and the limited availability of tissue, which precluded the analysis of all samples with the same antibody panel. By the method described above, 11 markers and 30 samples were removed, which reduced the proportion of missing values before imputation to 18%.

The final dataset for the analysis was composed of 409 tumor samples (264 core needle biopsies and 145 resection specimens) from 370 individual patients and 27 immunohistochemical markers. For 29 patients, we recorded the results of immunohistochemical analysis on more than one tumor sample (e.g. diagnostic biopsy and resection specimen). Adenocarcinomas from the main anatomical locations were included in this series: ampullary carcinoma (n = 24), ductal pancreatic adenocarcinoma (n = 139), distal bile duct cancer (n = 7), gallbladder cancer (n = 37), perihilar cholangiocarcinoma (n = 27), and intrahepatic cholangiocarcinoma (n = 97). As described in Methods, tumor samples of hepatocellular carcinoma (n = 78) were included as a control group for the evaluation of clustering results.

***Analysis of confounding factors*:** First, the set of markers analyzed in the several anatomical tumor types was tested by replacing in each tumor sample the immunohistochemical scores (data values) by a binary constant indicating whether the marker had been analyzed or not. The corresponding plot of principal component analysis (PCA) (S1 Fig) showed an admixture of all pancreatic and biliary anatomical diagnoses and clustering tendency for hepatocellular carcinoma (the control group). The former disregarded bias due to marker selection for the pancreatic and biliary tumors object of this study.

PCA of the immunohistochemical scores labelled by the type of probe (biopsy/resection) showed an admixture of probe types throughout the entire plot (S1 Fig), disregarding its role as confounding factor.

#### Cluster analysis

**Fig 2:**
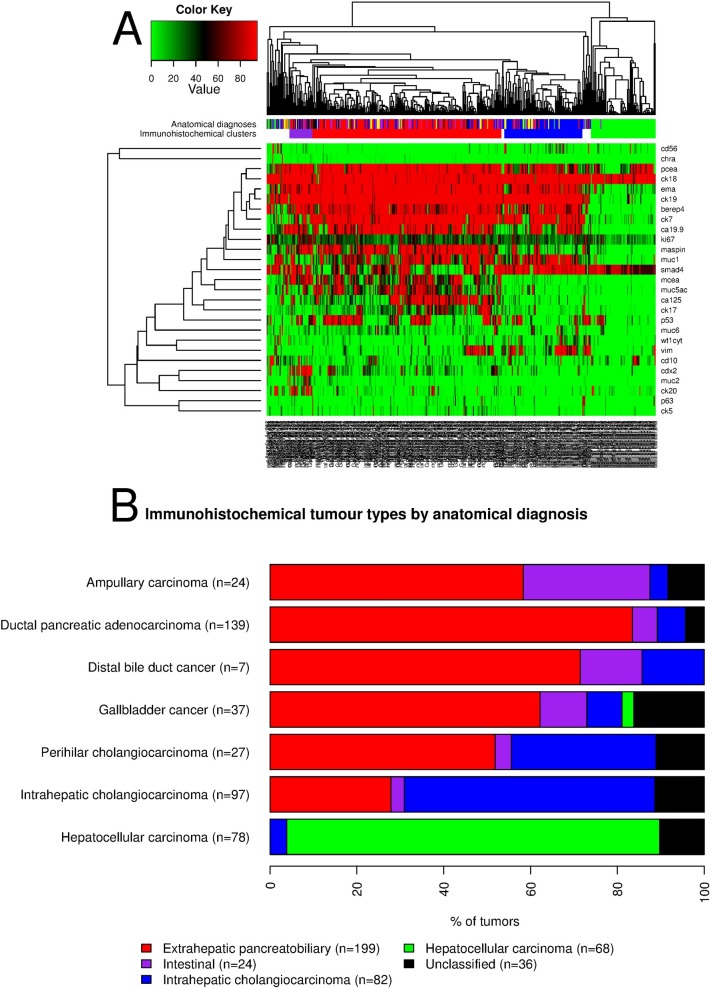
(A) Semi-supervised hierarchical cluster analysis and associated heatmap of tumor samples (n = 409) and immunohistochemical markers (n = 27). (B) Bar chart showing the relative distribution of the immunohistochemical tumor types within each anatomy-based diagnosis (n tumor samples = 409), according to the results of hierarchical cluster analysis.

Hierarchical clustering (Fig 2A) identified three major groups: extrahepatic pancreatobiliary, intrahepatic cholangiocarcinoma, and hepatocellular carcinoma; and one minor group, the intestinal.

Fig 2B and S2 Table detail the relationship between the immunohistochemical tumor types (clusters) and the anatomical diagnoses. In summary, the three major immunohistochemical types were largely composed of the anatomy-based diagnoses referred by their denomination. The intestinal type (n = 24) was dominated by an admixture of tumors from the pancreas (n = 8), ampulla (n = 7), and gallbladder (n = 4). 14 perihilar cholangiocarcinomas (n = 27) and 27 intrahepatic cholangiocarcinomas (n = 97) showed the extrahepatic pancreatobiliary immunohistochemical type.

#### Graphical visualizations

**Fig 3:**
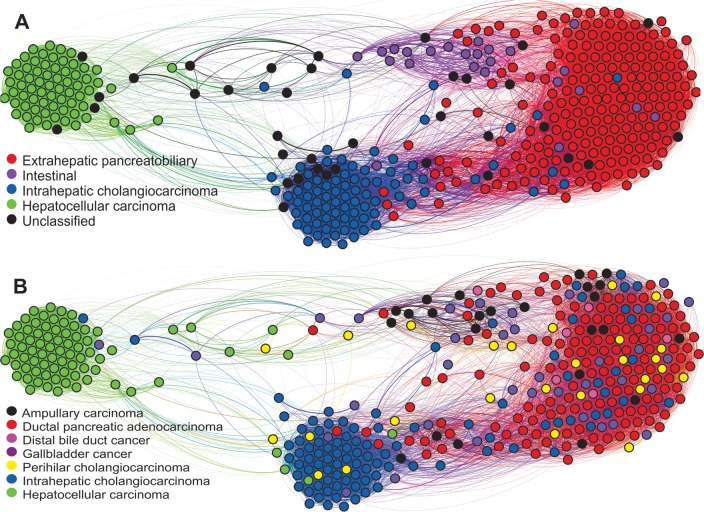
Complex network visualizations of the hepato-pancreato-biliary tumors in the study (n = 409) based on immunohistochemical data. Each circle (node) represents an individual tumor sample and is colored according to (A) the immunohistochemical type and (B) the anatomy-based diagnosis.

The complex network (Fig 3) consistently separated all the immunohistochemical tumor types. The topographical disposition and connections between the nodes of the network provides an integrative insight into the spectrum of differentiation of hepato-pancreato-biliary tumors.

**Fig 4:**
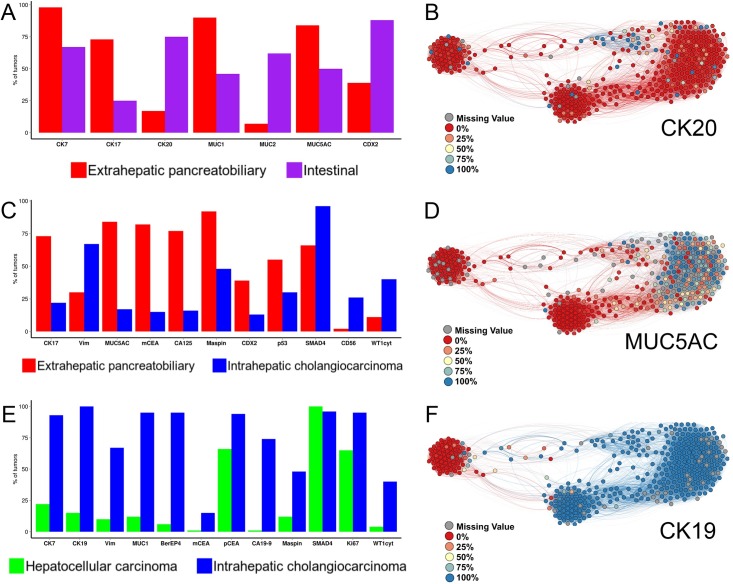
(A, C, E) Comparative immunohistograms between the immunohistochemical tumor types. The X-axis shows the significant markers, according to the results of differential expression analysis. The Y-axis indicates the percentage of tumor samples in which marker expression was positive, defined as immunoreactivity in more than 10% of the tumor cells. (B, D, F) Immunohistochemical profile visualizations on the complex network. Each circle (node) represents an individual tumor sample and is colored according to the percentage of stained tumor cells for one characteristic marker of the (B) intestinal, (D) extrahepatic pancreatobiliary, and (F) intrahepatic cholangiocarcinoma immunohistochemical types.

Comparative immunohistograms including only the statistically significant markers (Fig 4) illustrate the differences in marker expression between the immunohistochemical tumor types, according to the results of differential expression analysis (S2 File). In addition, comprehensive immunohistochemical profiles for every immunohistochemical tumor type and marker are provided on-line (as immunohistograms: S2 Fig; as complex network visualizations: S3 Fig).

#### Internal model validation

In a first experiment, the reproducibility and robustness of the immunohistochemical tumor types were tested by evaluating their classification using machine-learning models. Three sets of classifications were conducted using the full set of (27) markers: extrahepatic pancreatobiliary vs. intestinal, extrahepatic pancreatobiliary vs. intrahepatic cholangiocarcinoma, and intrahepatic cholangiocarcinoma vs. hepatocellular carcinoma.

The average true positive rate was around 0.96 across the three sets, while the average false positive rate was around 0.1. The average *ROC area* value in the classifications was about 0.96.

The second experiment tested the hypothesis that the performance of the machine learning models remained stable when the same pairwise classifications were carried out using only the significant markers, according to the results of differential expression analysis, instead of the whole set of markers.

The average true positive rate and false positive rate were around 0.96 and 0.12, while the average *ROC area* value was 0.95.

The full details of machine learning experiments are provided on-line (S3 Table).

#### Diagnostic immunohistochemical panel

Finally, we developed based on the results of differential expression analysis and characteristic immunoprofiles (S2 Fig) a simplified diagnostic immunohistochemical panel including eight highly discriminant markers between the immunohistochemical tumor types (Table 1): CK19, CK20, MUC2, MUC5AC, CA19-9, monoclonal CEA, CA125 and SMAD4.

**Table 1:** Proposal of diagnostic immunohistochemical panel for the classification of the immunohistochemical tumor types.

	Extrahepatic pancreatobiliary	Intestinal	Intrahepatic cholangiocarcinoma	Hepatocellular carcinoma
CK19	+	+	+	-
CK20	-/+	+/-	-	-
MUC2	-	+/-	-	-
MUC5AC	+/-	-/+	-	-
CA19-9	+	+	+/-	-
mCEA	+/-	+	-	-
CA125	+/-	-/+	-	-
SMAD4	-/+	+	+	+

**Fig 5:**
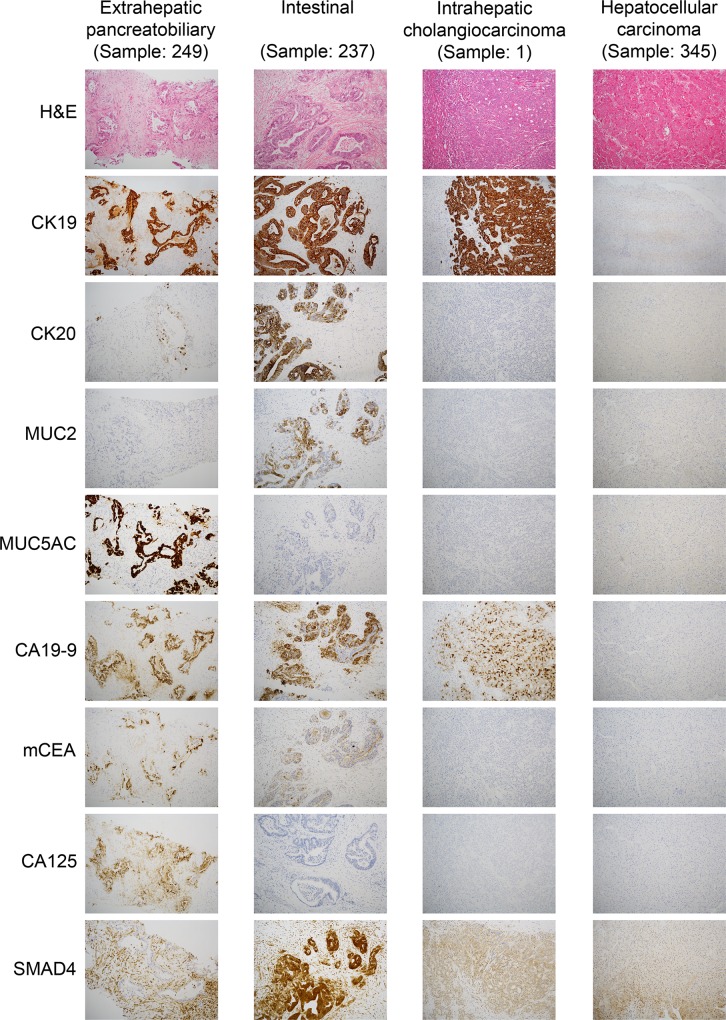
Photomicrographs (10x) of one representative tumor sample for each immunohistochemical tumor type, illustrating their characteristic morphological patterns of marker expression.

As a real-world example, the photomicrographs in Fig 5 illustrate how the diverging immunohistochemical profiles can be morphologically identified to diagnose the immunohistochemical tumor types.

### Survival analysis

Most tumors were locally advanced or/and with metastatic disease at the time of diagnosis. Survival analysis was performed on 86 patients whose primary tumor had been surgically resected with curative intent, of whom 47 were women. Median age was 66 (range 32–83) years. Patients with only diagnostic needle core biopsy had tumors deemed as non-eligible for resection; their generally short survival time (median overall survival (OS) = 6 months; S3 File) did not allow a meaningful survival analysis.

Survival analysis was therefore performed for patients whose primary tumor had been resected with curative intent according to tumor type by both the immunohistochemical and anatomy-based classifications (Table 2).

**Table 2:** Clinicopathological data and results of univariate and multivariate analysis of prognostic factors for overall survival in patients with resected adenocarcinoma of the pancreato-biliary system (n = 86). In multivariate analysis, overall survival was adjusted by pathological tumor stage (pT) and lymph node status (pN).

	Number of patients	Median overall survival (months)	Univariate		Multivariate	
			Crude HR (95% CI)	p value	Adjusted HR (95% CI)	p value
**Age (years)**						
> 66^a^	42	32	1			
≤ 66	44	30	1.14 (0.69–1.88)	0.601		
**Gender**						
F^a^	47	30	1			
M	39	33	0.77 (0.47–1.29)	0.322		
**Pathological tumor stage**				<0.001 ***		
pTis	1					
pT1	10	78	0.46 (0.19–1.10)	0.081 .		
pT2	27	34	0.75 (0.42–1.34)	0.330		
pT3^a^	39	25	1			
pT4	9	7	3.37 (1.51–7.52)	0.003 **		
**Lymph node status**				<0.001 ***		
pNX	10					
pN0	28	49	0.36 (0.20–0.65)	<0.001 ***		
pN1 / 2 (n = 2)	48	19	1			
**Immunohistochemical type**				<0.001 ***		0.001 **
Extrahepaticpancreatobiliary^a^	57	24	1		1	
Intestinal	8	54	0.31 (0.11–0.87)	0.026 *	0.19 (0.05–0.72)	0.014 *
Intrahepaticcholangiocarcinoma	14	109	0.26 (0.11–0.61)	0.002 **	0.61 (0.22–1.69)	0.340
**Anatomicalclassification**				0.068 .		0.005 **
Ductal pancreatic adenocarcinoma^a^	30	19	1		1	
Ampullarycarcinoma	12	31	0.66 (0.31–1.42)	0.290	0.90 (0.40–2.04)	0.799
Distal bile duct cancer	6	60	0.52 (0.18–1.49)	0.223	0.68 (0.20–2.31)	0.536
Gallbladder cancer	11	26	0.91 (0.42–1.95)	0.803	1.40 (0.59–3.32)	0.447
Perihilar cholangiocarcinoma	9	NA^b^	0.25 (0.08–0.71)	0.010 *	0.58 (0.19–1.83)	0.356
Intrahepatic cholangiocarcinoma	18	56	0.49 (0.24–1.00)	0.051 .	1.78 (0.74–4.26)	0.195

HR, hazard ratio; CI, confidence interval; NA, not available (median survival time not reached)

^a^ Baseline category

^b^ Median overall survival not reached at end of follow-up; restricted mean = 77 months

Significance codes

*** P ≤ 0.001

** P ≤ 0.01

* P ≤ 0.05, and P ≤ 0.1

Lymph node status determined OS in univariate (p value < 0.001) and multivariate analysis (by immunohistochemical type: p value = 0.045; by anatomical diagnosis: p value = 0.020).

**Fig 6:**
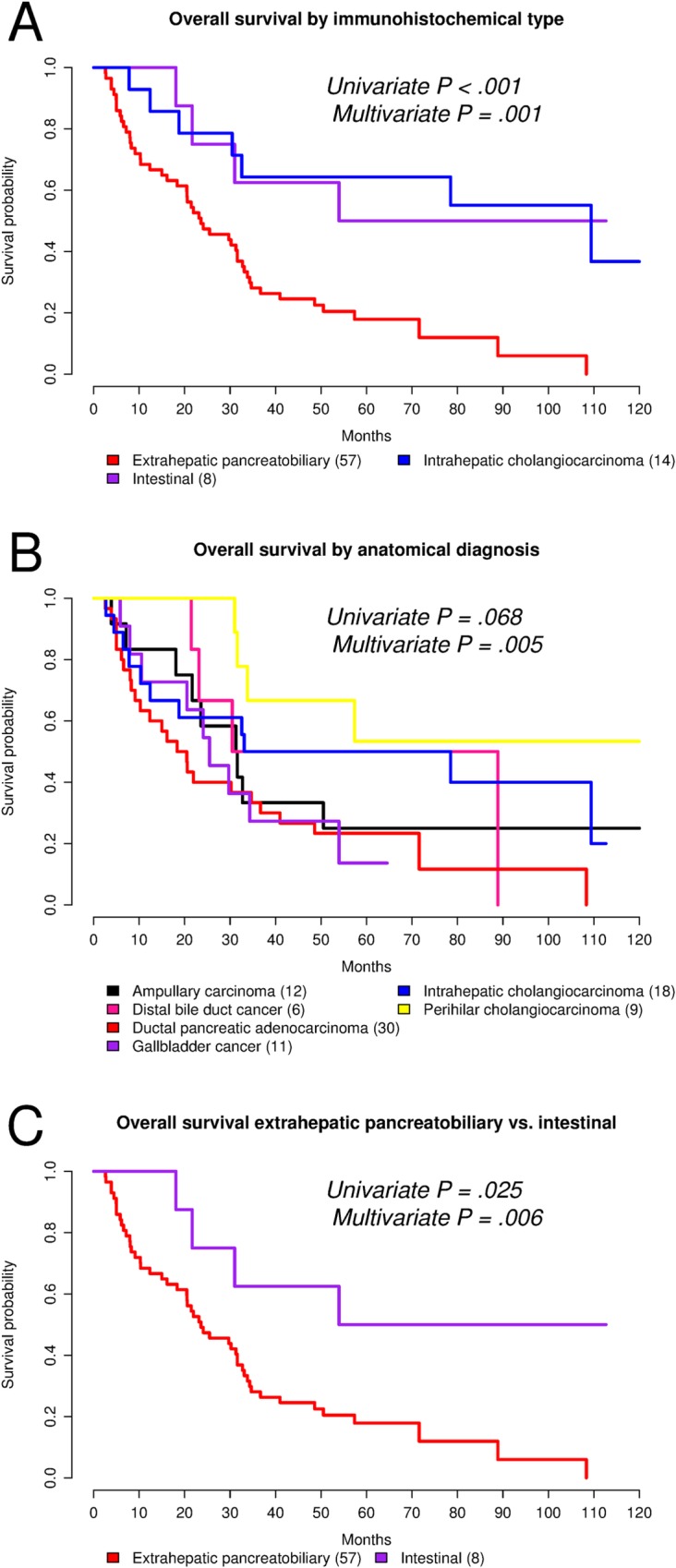
(A-C) Kaplan-Meier plots for overall survival. The p values correspond to log rank test. In multivariate analysis, overall survival was adjusted by pathological tumor stage (pT) and lymph node status (pN).

When considering the immunohistochemical tumor type (Fig 6A), we identified two definite trends: one of poor overall survival that corresponded to the extrahepatic pancreatobiliary type (median OS = 24 months), and a more favorable overall survival for the intestinal (median OS = 54 months) and intrahepatic cholangiocarcinoma (median OS = 109 months) types. Overall, the differences in overall survival by immunohistochemical type had a p value < 0.001 in univariate and just above 0.001 in multivariate analysis. In the latter, the intestinal type (p value = 0.014), pT4 (p value = 0.002) and pN0 (p value = 0.045) were independent predictors of OS.

By anatomy-based diagnosis (Fig 6B), ductal pancreatic adenocarcinoma (the reference group) showed the poorest overall survival (median = 19 months). Ampullary carcinoma (median OS = 31 months), distal bile duct cancer (median OS = 60 months), gallbladder cancer (median OS = 26 months), and intrahepatic cholangiocarcinoma (median OS = 56 months) showed statistically comparable overall survival in both univariate and multivariate analysis. Only perihilar cholangiocarcinoma (median OS not reached at end of follow-up; restricted mean OS = 77 months) showed a statistically significant better overall survival on univariate analysis (p value = 0.010). Globally, the differences in overall survival by anatomy-based diagnosis had a univariate p value = 0.068 and multivariate = 0.005. In the latter, only pT4 (p value = 0.035) and pN0 (p value = 0.020) were independent predictors of OS.

When comparing extrahepatic cancers of pancreatobiliary and intestinal immunohistochemical type (Fig 6C), the intestinal group was associated with a better overall survival (univariate: crude hazard ratio = 0.33, 95% confidence interval = 0.12–0.91, p value = 0.025; multivariate: adjusted hazard ratio = 0.19; 95% confidence interval = 0.05–0.71; p value = 0.014).

The Cox model proportional hazards assumption was tested as well in univariate and multivariate analysis (S3 File). It yielded overall a p value > 0.05, indicating lack of evidence to contradict this assumption.

## Discussion

Using an extended panel of immunohistochemical markers, we derived several tumor types based on their marker profile. To the best of our knowledge, this is the first study to develop an integrative immunohistochemical classification of adenocarcinomas from the main anatomical locations of the pancreatobiliary system. It introduces three distinct subtypes, extrahepatic pancreatobiliary, intestinal, and intrahepatic cholangiocarcinoma, with differences in prognosis, biology, and potentially in response to treatment.

The three immunohistochemical types presented in this study simplify and integrate the six tumor classes in the anatomy-based (AJCC, UICC-TNM 7^th^ edition) classification[5,6]: ampullary carcinoma, ductal pancreatic adenocarcinoma, distal bile duct cancer, gallbladder carcinoma, perihilar cholangiocarcinoma, and intrahepatic cholangiocarcinoma.

Due to the intrinsic limitations in interobserver agreement linked to the assessment of hematoxylin-eosin stained slides, the current lack of reliable diagnostic criteria, especially when tumors show mixed or intermediate histological features[10,35,36], and to minimize design bias to predefined categories, a pure histomorphological classification based on hematoxylin-eosin staining was not addressed.

### Intrahepatic cholangiocarcinoma

On light microscopy (hematoxylin-eosin stain), intrahepatic cholangiocarcinoma can be very difficult to distinguish from metastatic adenocarcinoma, especially in the presence of multiple radiological tumor nodules in liver.

A combination of “generic” adenocarcinoma markers (CK7, CK19, BerEP4, and polyclonal CEA) together with vimentin and a limited number of “pancreatobiliary” markers (MUC1 and CA19-9) defines its characteristic immunohistochemical profile (Fig 4C and 4E, S2 Fig). The present study identified cytoplasmic WT1 as a novel marker for intrahepatic cholangiocarcinoma. This finding warrants further validation in external cohorts.

The type of probe was not a confounding variable. This supports the adequacy of both core needle biopsy and resection specimen for the immunohistochemical diagnosis of intrahepatic cholangiocarcinoma.

### Extrahepatic pancreatobiliary type

The extrahepatic pancreatobiliary immunohistochemical profile is characterized by expression of a considerable number of markers (Fig 4A and 4C, S2 Fig), including cytokeratins (CK7, CK17, and CK19), mucins (MUC1 and MUC5AC), and tumor-associated epithelial markers (CA19-9, monoclonal CEA, CA125, and maspin).

This profile was the most common among adenocarcinomas originated in the pancreas and the extrahepatic biliary tract. Half of perihilar cholangiocarcinomas were of the extrahepatic pancreatobiliary type, but only about a third of intrahepatic cholangiocarcinomas. This may be a reflection of the different proportions of large and minor biliary branches that are present in the perihilar and peripheral regions of the liver.

### Intestinal type

Several studies on periampullary adenocarcinomas have demonstrated that the histological type (pancreatobiliary vs. intestinal) rather than the primary tumor location determines survival[9–11]. There is growing evidence that the intestinal type is associated with a less aggressive tumor biology and a better prognosis.

In the present series, the intestinal type expressed CK20, MUC2, and CDX2, which is in agreement with the literature[37]. As reported in previous morphological studies[9,10], we observed overlap with the pancreatobiliary type also at the immunohistochemical level. As depicted in Fig 4A, tumors of the intestinal type co-expressed pancreatobiliary markers, such as CK7, CK17, MUC1, and MUC5AC, although to a lesser extent. In particular, CK7 was expressed in approximately 70% of tumors of the intestinal type, MUC1 and MUC5AC in 50%, and CK17 in 25%. Furthermore, other pancreatobiliary markers, i.e. CA19-9 and CA125, were also expressed in 90% and 50% of tumors of the intestinal type, respectively (S2 Fig). There were no defined subgroups in our cluster analysis within the intestinal type. Rather, its “mixed” instead of “purely” colorectal-like immunophenotype seems to be a distinctive feature of intestinal type adenocarcinomas arising from the pancreatobiliary system.

Previous studies have pursued the immunohistochemical distinction between the intestinal and pancreatobiliary type[10,38]. However, they tested only a small number of predefined markers and this could explain the lower discriminant power yielded by their diagnostic algorithms. Future clinical trials may use more effective and extended panels of markers, such as those proposed (Table 1, Fig 4) and internally validated in this study, to stratify patients according to the immunohistochemical tumor type.

### Immunohistochemical diagnosis

We propose the diagnostic immunohistochemical panel in Table 1 and additional extended discriminant panels in Fig 4 as guidance for the diagnosis of the immunohistochemical tumor types. This panel may be of particular diagnostic use in cases with a complex differential diagnostic context (e.g. cancer of unknown primary or a patient history of multiple primary cancers) or for cancers with an immunohistochemical profile that is not consistent with traditionally described patterns. In such cases, the comprehensive discriminant panels in Fig 4 may prove useful to reach a diagnosis.

### Integrative perspective

To the best of our knowledge, this is the first study to employ complex networks [39] for visualizing immunohistochemical tumour types and the relationships between them. The depicted network (Fig 3) provides an integrative insight into the spectrum of differentiation of hepato-pancreato-biliary tumours.

In the spectrum, from hepatocellular carcinoma to the extrahepatic pancreatobiliary type, tumours showed progressively increasing numbers and levels of marker expression. Between them, the network depicted the intrahepatic cholangiocarcinoma and the intestinal clusters.

This model is consistent with the current state of knowledge on the development and carcinogenesis of the pancreatobiliary system. Both the ventral pancreas and the extrahepatic biliary tract arise from a contiguous region of the endoderm and share several developmental transcription factors. Recent studies [40,41] have demonstrated overexpression of PDX1 and alterations in HES1 expression not only in ductal pancreatic adenocarcinoma, but also in cholangiocarcinoma and biliary intraepithelial neoplasia.

The fact that ductal pancreatic adenocarcinoma and extrahepatic biliary tract cancer present comparable prognostic and clinicopathological features [41] seems to be in line with their similar histological and immunohistochemical phenotypes.

### Classification systems

The current (AJCC, UICC-TNM 7^th^ edition)[5,6] staging of tumors of the pancreatobiliary system is based on the anatomical site of origin. It provides a useful stratification scheme, but cannot identify prognostically relevant immunohistochemical/histological tumor types, like the intestinal. In our survival analysis, the extrahepatic pancreatobiliary type had the poorest overall survival while the intestinal type correlated with a better prognosis, which is in agreement with the literature[9–11]. Advanced tumor stage (pT4) and lymph node status (pN) were also independent predictors of overall survival in both the immunohistochemical and anatomical classifications, which highlights the critical impact of lymph node metastasis on the prognosis of pancreatobiliary cancer. It is therefore conceivable that differences in survival according to the anatomical classification may largely reflect the prognostic impact of site-specific features, i.e. the invaded locoregional structures, resectability options and pathways of tumor spread (lymphovascular, perineural) rather than intrinsic differences in tumor biology.

In line with other groups[10,11], we endorse a rational combination of the anatomical and immunohistochemical/histological classifications as a means to achieving improvement in prognostic stratification, treatment and survival for patients affected with pancreatobiliary cancer.

Regarding subgroup analysis, perihilar cholangiocarcinoma presented in univariate analysis a statistically significant better overall survival compared to ductal pancreatic adenocarcinoma, despite being dominated by the extrahepatic pancreatobiliary immunohistochemical type. This finding warrants further investigation in larger series addressing even the pathways of tumor spread.

### Semi-supervised clustering approach and internal model validation

A semi-supervised approach was pursued to evaluate from the perspective of diagnostic and clinical relevance the hierarchical clustering results and select the clusters of interest. The aim of this part of the work was to reveal the underlying categories (tumor types) in the distribution of the immunohistochemical data. When prior knowledge on the categories is not available, an unsupervised learning needs to be assumed. However, it is recognized in the data analysis field that there is no so called “perfect clustering”[42]. Clustering techniques attempt to organize data items based on their distribution or similarities and determine their grouping. Depending on the notion of similarity, the grouping is meaningful in terms of the data itself, but not necessarily in a semantic sense i.e. in the context of the specific domain knowledge.

A semi-supervised approach can provide a limited form of supervision in the clustering process, and align the computational grouping with semantically meaningful classes. In this research, a limited amount of human guidance was provided, enabling the clustering results to become most meaningful from a clinical-diagnostic perspective. Two features enabled the use of the semi-supervised approach in this study, the relatively low number of markers (e.g. compared to usual omics analyses that include thousands of them) and the team’s previous knowledge and expertise in the pathological diagnosis of pancreatobiliary tumors.

Nonetheless, we have strived to test the proposed clusters (immunohistochemical tumor types) by implementing an alternative, robust complex network approach as well as machine learning-based supervised classification. For the latter, we chose to use several and different reliable classifiers, not with the aim of pursuing the best classification performance, but rather to validate the proposed semi-supervised clustering results. Both techniques, very different algorithmically from hierarchical clustering, reproduced the immunohistochemical tumor groups, which we think supports the robustness of our semi-supervised clustering approach as well as the consistency and reproducibility of the immunohistochemical classification.

### Study limitations

This study has some limitations. During the study period (2002–2013) not all patients diagnosed at our institution could be included, but all consecutive cases routinely reported by the three consultant pathologists of the hepato-pancreato-biliary team (all of whom are co-authors; BB, CFM, OD). Still, 36 tumor samples remained as “unclassified” by the semi-supervised approach employed to derive the clusters of interest. Because the methods of investigation determine the study findings, the presented immunohistochemical types do not preclude the existence of further molecular types based on DNA or RNA data, as recently described[43,44]. Although the immunohistochemical types and their discrimination were internally validated, external validation in separate datasets and cohorts is desirable to reach a greater level of evidence. Likewise, a diagnostic algorithm based on a decision tree with precise cut-off values for the various markers would be valuable, but such level of specification would require a separate validation cohort. Finally, we acknowledge that survival analysis is limited by statistical power. Furthermore, because of the lack of detailed data on the cause of death, we were able to assess only overall survival and not cancer-specific death. However, as evident from autopsy and clinical follow-up studies[45,46], the vast majority of these patients die from their underlying disease, i.e. the cancer. Survival analysis for each individual anatomical diagnosis based on immunohistochemical type could not be done because of insufficient number of resected patients in each of the subgroups. However, we consider that the above presented survival results are essentially correct and in agreement with the literature[9–11].

## Conclusions

This study presents an integrative immunohistochemical classification of adenocarcinomas of the pancreatobiliary system that improves diagnosis and prognostic stratification and has potential therapeutic implications. It defines three immunohistochemical types, extrahepatic pancreatobiliary, intestinal, and intrahepatic cholangiocarcinoma and their discriminant markers.

The characteristic immunohistochemical profile of intrahepatic cholangiocarcinoma positively supports its pathological diagnosis, which no longer needs to be regarded as a diagnosis of exclusion (of metastatic adenocarcinoma).

The prognostically more favorable intestinal type can be distinguished from the more aggressive pancreatobiliary type.

A diagnostic immunohistochemical panel and additional extended panels of discriminant markers are proposed as guidance for the diagnosis of the immunohistochemical tumor types.

## Supporting Information

S1 Fig Principal component analysis for the analysis of potential confounding factors. (PDF)

S1 File Analysis of missing data and imputation. (PDF)

S1 Table Antibodies and staining protocols. (PDF)

S2 Fig Simple immunohistograms for the immunohistochemical tumor types and significant markers. (PDF)

S2 File Differential expression analysis. (PDF)

S2 Table Relationship between the immunohistochemical tumor types and anatomy-based diagnoses. (PDF)

S3 Fig Complex network-based immunoprofiles. (PDF)

S3 File Survival analysis. (PDF)

S3 Table Machine learning classification/internal model validation. (PDF)
