# Correction: Immunohistochemical Typing of Adenocarcinomas of the Pancreatobiliary System Improves Diagnosis and Prognostic Stratification

**DOI:** 10.1371/journal.pone.0171283

**Published:** 2017-01-26

**Authors:** Carlos Fernández Moro, Alejandro Fernandez-Woodbridge, Melroy Alistair D'souza, Qianni Zhang, Benedek Bozoky, Senthil Kandaswamy Vasan, Piera Catalano, Rainer Heuchel, Sonia Shtembari, Marco Del Chiaro, Olof Danielsson, Mikael Björnstedt, J. Matthias Löhr, Bengt Isaksson, Caroline Verbeke, Béla Bozóky

The sixth author’s name appears incorrectly in the published article. The correct name is: Senthil Kandaswamy Vasan. The correct citation is: Fernández Moro C, Fernandez-Woodbridge A, Alistair D'souza M, Zhang Q, Bozoky B, Vasan SK, et al. (2016) Immunohistochemical Typing of Adenocarcinomas of the Pancreatobiliary System Improves Diagnosis and Prognostic Stratification. PLoS ONE 11(11): e0166067. doi:10.1371/journal.pone.0166067

## References

[1] Fernández Moro C, Fernandez-Woodbridge A, Alistair D'souza M, Zhang Q, Bozoky B, Kandaswamy SV, et al (2016) Immunohistochemical Typing of Adenocarcinomas of the Pancreatobiliary System Improves Diagnosis and Prognostic Stratification. PLoS ONE 11(11): e0166067 doi:10.1371/journal.pone.0166067 27829047 10.1371/journal.pone.0166067 PMC5102456

