# Supplementary material for: Immunohistochemical Typing of Adenocarcinomas of the Pancreatobiliary System Improves Diagnosis and Prognostic Stratification
Source: PLoS One. 2016 Nov 9;11(11):e0166067. doi: 10.1371/journal.pone.0166067 (PMC5102456; doi:10.1371/journal.pone.0166067)

Supplementary Figure 1. Principal component analysis (PCA) plots used for the analysis of potential confounding factors. (A) The set of markers analyzed in the anatomical tumor types was tested by replacing in each tumor sample the immunohistochemical scores (data values) by a binary constant indicating if the marker had been analyzed or not. The plot shows an admixture of all pancreatic and biliary anatomical tumor types and clustering tendency for hepatocellular carcinoma (the control group). The former disregards bias due to marker selection for the pancreatic and biliary tumors object of this study. (B) Shows an admixture of probe types throughout the entire plot, disregarding its role as confounding factor.

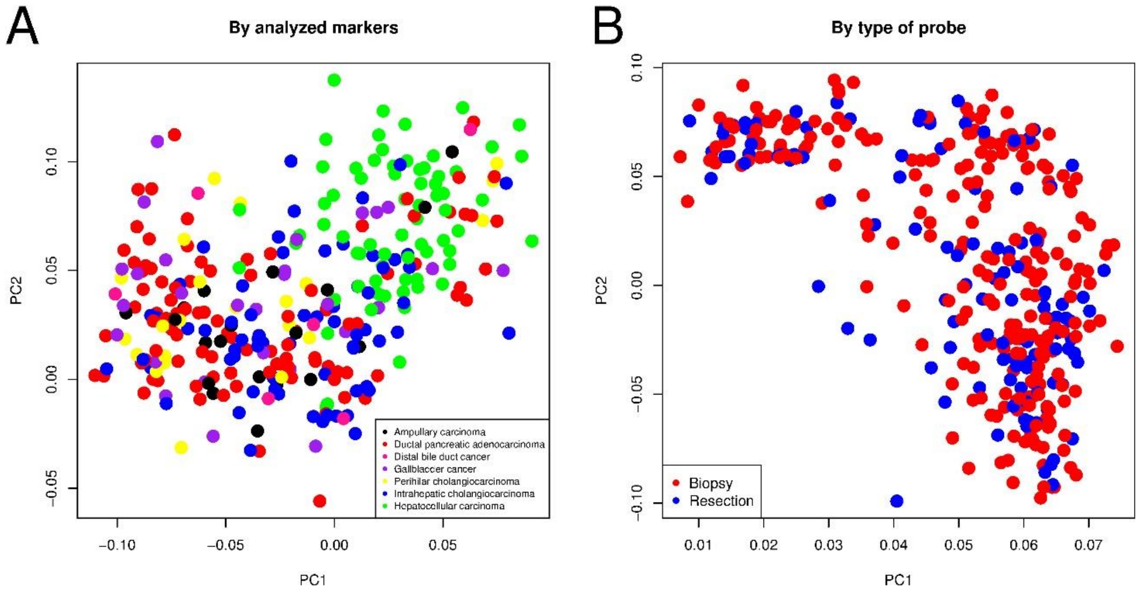

Supplement: S1 Fig — (PDF) [file pone.0166067.s001.pdf]
