# Supplementary material for: Immunohistochemical Typing of Adenocarcinomas of the Pancreatobiliary System Improves Diagnosis and Prognostic Stratification
Source: PLoS One. 2016 Nov 9;11(11):e0166067. doi: 10.1371/journal.pone.0166067 (PMC5102456; doi:10.1371/journal.pone.0166067)
Supplement: S1 File — (PDF) [file pone.0166067.s002.pdf]

Supplementary File 1

Immunoprofiling of adenocarcinomas of the pancreatobiliary system

Analysis of missing data & imputation

Generated on: 2016-03-03 17:26:10

Import required packages

```
# Build pre-requisites
# install.packages("plyr")
# install.packages("Amelia")
# source("http://bioconductor.org/biocLite.R")
# biocLite("impute")

library(plyr)
library(Amelia)
```

```
## Loading required package: Rcpp
## ##
## ## Amelia II: Multiple Imputation
## ## (Version 1.7.3, built: 2014-11-14)
## ## Copyright (C) 2005-2016 James Honaker, Gary King and Matthew Blackwell
## ## Refer to http://gking.harvard.edu/amelia/ for more information
## ##
```

```
library(impute)
```

Configure file names

```
#myWorkDirectory <- "~/Workspace/research/pcbil/"
myWorkDirectory <-  "/home/guest/pcbil/"
rawDataFileName <- paste(myWorkDirectory, "raw/pcbil_raw.csv", sep="")
imputedDataFileName  <- paste(myWorkDirectory, "data_analysis/tidy_datasets/
pcbil_imputed.csv", sep="")
```

Importing raw dataset /home/guest/pcbil/raw/pcbil_raw.csv …

```
pcbilDataRaw <- read.csv(file = rawDataFileName, row.names = 39, colClasses
= c(rep("numeric",38), rep("character",2)), na.strings = "?",quote="'" )
```

Creating working dataset for processing

```
pcbilDataWork <- pcbilDataRaw
```

Process column names

```
names(pcbilDataWork) <- tolower(names(pcbilDataWork))
names(pcbilDataWork)[39] <- "clin_diag"
pcbilDataWork$clin_diag <- as.factor(pcbilDataWork$clin_diag)
```

Recode clinical diagnoses according to AJCC/IUCC-TNM 7 nomenclature

```
revalue(pcbilDataWork$clin_diag, c("Ampulla Ac" = "Ampullary carcinoma"))
-> pcbilDataWork$clin_diag
revalue(pcbilDataWork$clin_diag, c("Distal Bile Duct Ac" = "Distal bile duc
t cancer")) -> pcbilDataWork$clin_diag
revalue(pcbilDataWork$clin_diag, c("Gallbladder Ac" = "Gallbladder cance
r")) -> pcbilDataWork$clin_diag
revalue(pcbilDataWork$clin_diag, c("Hepatocellular Cancer" = "Hepatocellula
r carcinoma")) -> pcbilDataWork$clin_diag
revalue(pcbilDataWork$clin_diag, c("Intrahepatic Cholangiocarcinoma" = "Intr
ahepatic cholangiocarcinoma")) -> pcbilDataWork$clin_diag
revalue(pcbilDataWork$clin_diag, c("Pancreas Ac" = "Ductal pancreatic adenoc
arcinoma")) -> pcbilDataWork$clin_diag
revalue(pcbilDataWork$clin_diag, c("Perihilary Ac" = "Perihilar cholangiocar
cinoma")) -> pcbilDataWork$clin_diag
```

Show fixed column names

```
head(pcbilDataWork[1:5])
```

```
##                                      ck5  ck7 ck17 ck18 ck19
## Pancreas Ac|397                        0 92.5   20   NA 92.5
## Hepatocellular Cancer|190              0 20.0    0 92.5 50.0
## Pancreas Ac|25                         0 92.5    0 92.5 92.5
## Gallbladder Ac|108                    NA 70.0   NA 92.5 85.0
## Hepatocellular Cancer|336              0  0.0   NA 92.5 40.0
## Intrahepatic Cholangiocarcinoma|293    5 92.5    5   NA 92.5
```

Report raw dataset composition

```
#Total number of tumor samples
dim(pcbilDataWork)[1]
```

```
## [1] 439
```

```
#Total number of markers
dim(pcbilDataWork)[2]
```

```
## [1] 39
```

```
#List of markers
names(pcbilDataWork)
```

```
##  [1] "ck5"        "ck7"        "ck17"       "ck18"       "ck19"      
##  [6] "ck20"       "vim"        "muc1"       "muc2"       "muc5ac"    
## [11] "muc6"       "berep4"     "ema"        "mcea"       "pcea"      
## [16] "ca125"      "ca19.9"     "maspin"     "wt1cyt"     "ttf1"      
## [21] "cdx2"       "p53"        "p63"        "ki67"       "ezh2"      
## [26] "smad4"      "chra"       "synap"      "cd56"       "cd10"      
## [31] "cd146"      "cd146_nucl" "calretinin" "hbme1"      "mesothelin"
## [36] "hepatocyte" "glypican_3" "ngfr_str"   "clin_diag"
```

Fix one incorrect anatomical-based diagnosis

```
pcbilDataWork[rownames(pcbilDataWork) == "Intrahepatic Cholangiocarcinoma|36
6" , "clin_diag"] <- "Gallbladder cancer"
```

Composition of the raw dataset by atanomical-based diagnosis

```
table(pcbilDataWork$clin_diag)
```

```
## 
##             Ampullary carcinoma          Distal bile duct cancer 
##                              24                                8 
##              Gallbladder cancer         Hepatocellular carcinoma 
##                              38                              100 
##  Intrahepatic cholangiocarcinoma Ductal pancreatic adenocarcinoma 
##                              98                              143 
##     Perihilar cholangiocarcinoma 
##                              28
```

Summary of missing values by marker

```
propmiss <- function(dataframe) {
  m <- sapply(dataframe, function(x) {
    data.frame(
            nmiss=sum(is.na(x)),
            n=length(x),
            propmiss=round(sum(is.na(x))/length(x),2)
        )
    })
    d <- data.frame(t(m))
    d <- sapply(d, unlist)
    d <- as.data.frame(d)
    d$variable <- row.names(d)
    row.names(d) <- NULL
    d <- cbind(d[ncol(d)],d[-ncol(d)])
    return(d[order(d$propmiss), ])
}

reportmiss <- function(dataframe) {
  propMiss <- propmiss(dataframe)
  print(propMiss)
  totalNumValues <- dim(dataframe)[1] * (dim(dataframe)[2] - 1)
  totalMissingValues <- sum(propMiss$nmiss)
  return ( (100 / totalNumValues) * totalMissingValues  )
}

pcbilDataWorkMissingValues <- reportmiss(pcbilDataWork)
```

```
##      variable nmiss   n propmiss
## 39  clin_diag     0 439     0.00
## 2         ck7    14 439     0.03
## 6        ck20    20 439     0.05
## 7         vim    31 439     0.07
## 22        p53    46 439     0.10
## 24       ki67    44 439     0.10
## 5        ck19    49 439     0.11
## 15       pcea    64 439     0.15
## 16      ca125    66 439     0.15
## 17     ca19.9    71 439     0.16
## 21       cdx2    69 439     0.16
## 4        ck18    75 439     0.17
## 9        muc2    79 439     0.18
## 10     muc5ac    79 439     0.18
## 8        muc1    83 439     0.19
## 11       muc6    92 439     0.21
## 3        ck17   100 439     0.23
## 29       cd56    99 439     0.23
## 1         ck5   113 439     0.26
## 19     wt1cyt   114 439     0.26
## 14       mcea   120 439     0.27
## 18     maspin   125 439     0.28
## 23        p63   132 439     0.30
## 12     berep4   164 439     0.37
## 13        ema   167 439     0.38
## 27       chra   171 439     0.39
## 30       cd10   171 439     0.39
## 26      smad4   175 439     0.40
## 20       ttf1   211 439     0.48
## 31      cd146   247 439     0.56
## 38   ngfr_str   273 439     0.62
## 32 cd146_nucl   293 439     0.67
## 33 calretinin   313 439     0.71
## 37 glypican_3   320 439     0.73
## 35 mesothelin   357 439     0.81
## 34      hbme1   365 439     0.83
## 36 hepatocyte   365 439     0.83
## 25       ezh2   389 439     0.89
## 28      synap   397 439     0.90
```

```
#Total percentage of missing data in processed dataset (%):
round(pcbilDataWorkMissingValues, 0)
```

```
## [1] 36
```

Summary of missing values by tumour sample

```
reportmissperrow <- function(dataframe) {

  pads <- c()
  numMissingValues <- c()
  propMissingValues <- c()

  aRow <- 1
  while(aRow <= nrow(dataframe)) {
    pads <- c(pads, row.names(dataframe)[aRow])
    numMissing <- sum(is.na(dataframe[aRow,]))
    propMissingValues <- c(propMissingValues, round((100 / (dim(dataframe)
[2] -1)) *numMissing,2))
    numMissingValues <- c(numMissingValues, numMissing)
    aRow <- aRow + 1
  }
  missingD <- data.frame(pad = pads, nmissing = numMissingValues, propmissin
g = propMissingValues, stringsAsFactors=F)
  missingD$propmiss_interv <- cut(missingD$propmissing, c(0,10,20,30,40,50,6
0,70,80,90,100))

  print(table(missingD$propmiss_interv))

  return (subset(missingD, propmissing > 50)[, 1])
}

numCasesMissGreat50 <- reportmissperrow(pcbilDataWork)
```

```
##
##   (0,10]  (10,20]  (20,30]  (30,40]  (40,50]  (50,60]  (60,70]  (70,80]
##       10       48      107      123       72       49       18        5
##  (80,90] (90,100]
##        6        1
```

```
#Number of cases in work dataset with more than 50% missing data:
length(numCasesMissGreat50)
```

```
## [1] 79
```

Amelia Missingness Map

```
missmap(pcbilDataWork, main = "Missingness map pcbilDataWork")
```

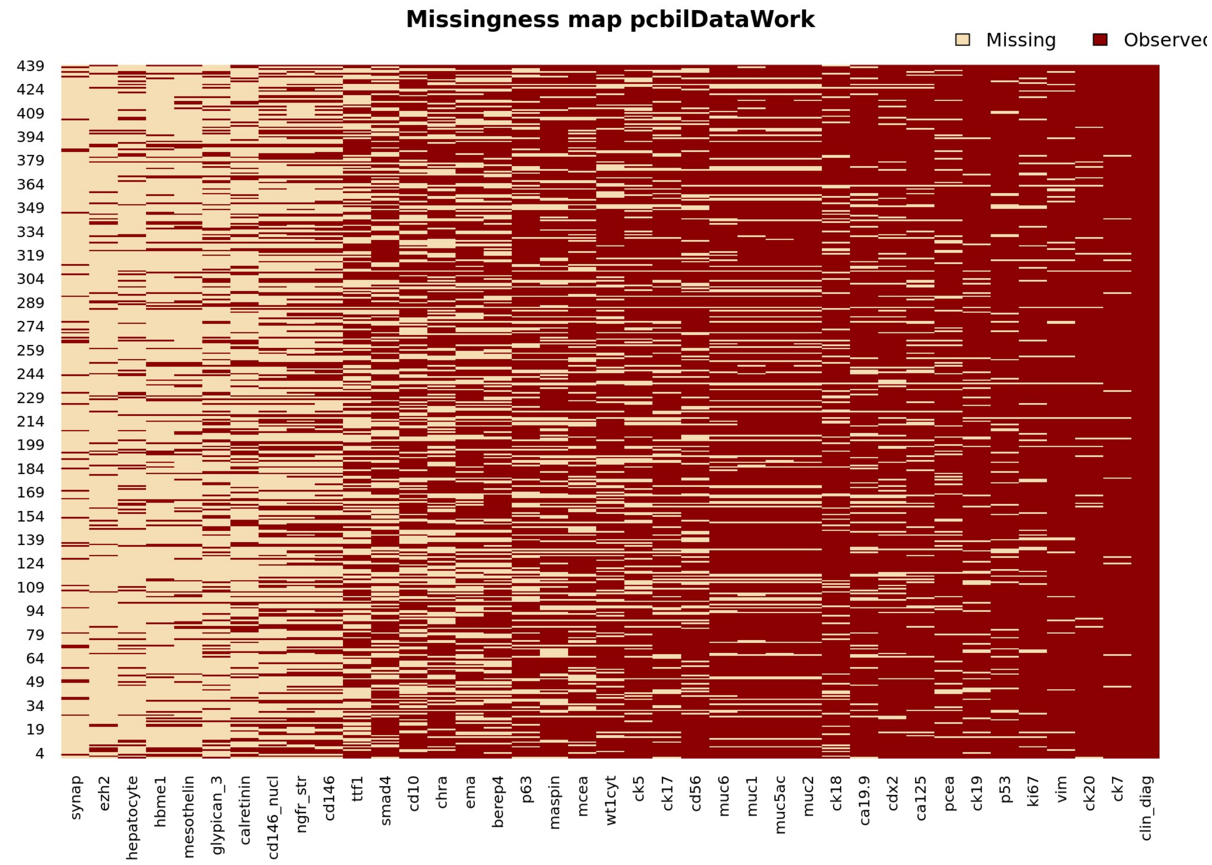

Mising values handling strategy, step 1 - remove markers with > 40% missing values: ttf1, cd146, cd146_nucl, ngfr_str, calretinin, glypican_3, ezh2, synap, hbme1, mesothelin, hepatocyte

```
pcbilDataMarkerTrimmed <- subset(pcbilDataWork, select=-c(ttf1, cd146, cd146_nucl, ngfr_str, calretinin, glypican_3, ezh2, synap, hbme1, mesothelin, hepatocyte))
```

Check the missing values in the marker trimmed dataset

```
pcbilDataMarkerTrimmedMissingValues <- reportmiss(pcbilDataMarkerTrimmed)
```

```
##     variable nmiss   n propmiss
## 28 clin_diag     0 439     0.00
## 2        ck7    14 439     0.03
## 6       ck20    20 439     0.05
## 7        vim    31 439     0.07
## 21       p53    46 439     0.10
## 23      ki67    44 439     0.10
## 5       ck19    49 439     0.11
## 15      pcea    64 439     0.15
## 16     ca125    66 439     0.15
## 17    ca19.9    71 439     0.16
## 20      cdx2    69 439     0.16
## 4       ck18    75 439     0.17
## 9       muc2    79 439     0.18
## 10    muc5ac    79 439     0.18
## 8       muc1    83 439     0.19
## 11      muc6    92 439     0.21
## 3       ck17   100 439     0.23
## 26      cd56    99 439     0.23
## 1        ck5   113 439     0.26
## 19    wt1cyt   114 439     0.26
## 14      mcea   120 439     0.27
## 18    maspin   125 439     0.28
## 22       p63   132 439     0.30
## 12    berep4   164 439     0.37
## 13       ema   167 439     0.38
## 25      chra   171 439     0.39
## 27      cd10   171 439     0.39
## 24     smad4   175 439     0.40
```

```
#Total percentage missing data in dataset after trimming of markers: (%)
round(pcbilDataMarkerTrimmedMissingValues, 0)
```

```
## [1] 21
```

```
missmap(pcbilDataMarkerTrimmed)
```

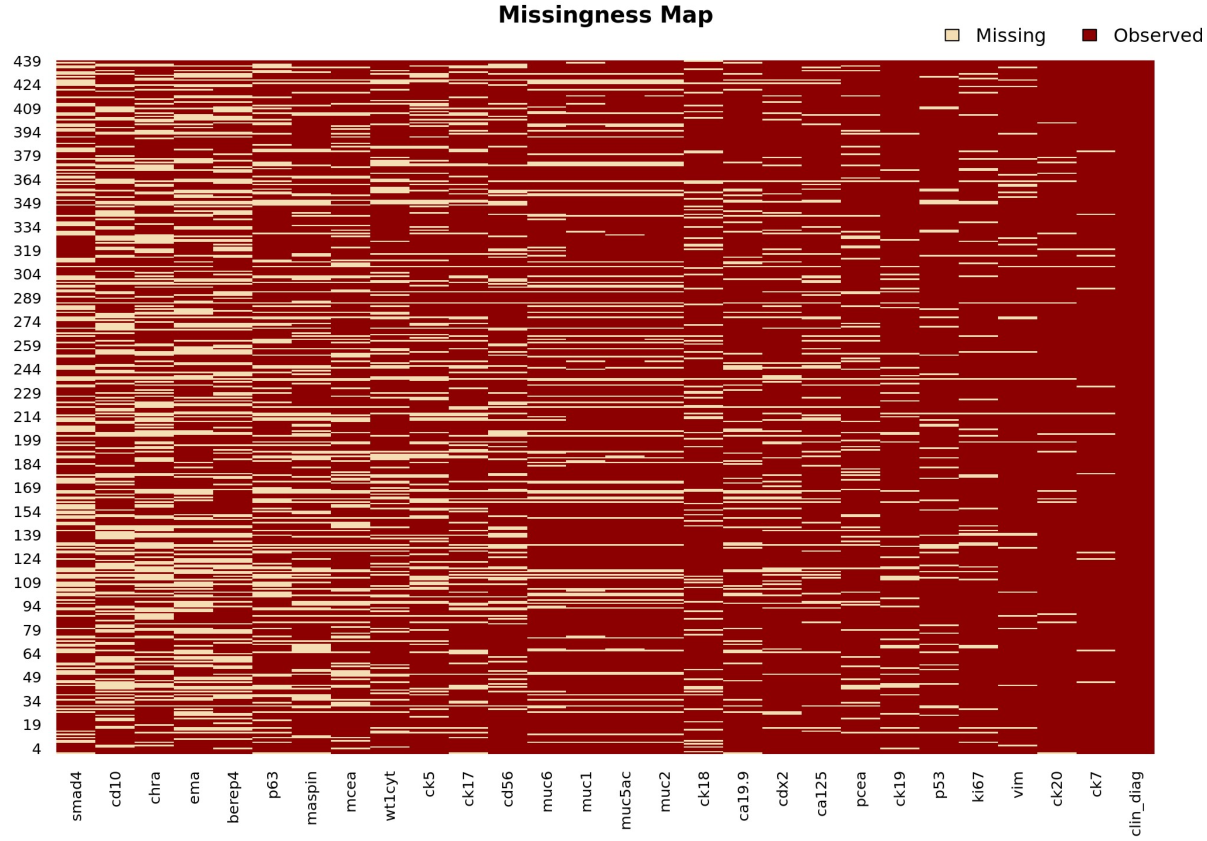

Filtering of markers with >40% missing data resulted in 15% improvement on data-value coverture

Missing values in marker trimmed dataset, by tumor sample

```
samplesWithMissingGreater50 <- reportmissperrow(pcbilDataMarkerTrimmed)
```

```
##
##   (0,10]  (10,20]  (20,30]  (30,40]  (40,50]  (50,60]  (60,70]  (70,80]
##       94      148       75       39       34       14        6        6
##  (80,90] (90,100]
##        3        1
```

```
#Number of cases in filtered dataset with more than 50% missing data:
length(samplesWithMissingGreater50)
```

```
## [1] 30
```

```
print(samplesWithMissingGreater50)
```

```
##  [1] "Hepatocellular Cancer|135"
##  [2] "Hepatocellular Cancer|263"
##  [3] "Hepatocellular Cancer|368"
##  [4] "Pancreas Ac|409"
##  [5] "Pancreas Ac|146"
##  [6] "Pancreas Ac|245"
##  [7] "Hepatocellular Cancer|120"
##  [8] "Hepatocellular Cancer|11"
##  [9] "Hepatocellular Cancer|35"
## [10] "Hepatocellular Cancer|240"
## [11] "Hepatocellular Cancer|308"
## [12] "Hepatocellular Cancer|378"
## [13] "Hepatocellular Cancer|438"
## [14] "Perihilary Ac|239"
## [15] "Hepatocellular Cancer|155"
## [16] "Hepatocellular Cancer|90"
## [17] "Hepatocellular Cancer|408"
## [18] "Hepatocellular Cancer|21"
## [19] "Hepatocellular Cancer|436"
## [20] "Hepatocellular Cancer|242"
## [21] "Distal Bile Duct Ac|222"
## [22] "Hepatocellular Cancer|321"
## [23] "Hepatocellular Cancer|319"
## [24] "Hepatocellular Cancer|62"
## [25] "Hepatocellular Cancer|121"
## [26] "Intrahepatic Cholangiocarcinoma|29"
## [27] "Pancreas Ac|136"
## [28] "Hepatocellular Cancer|411"
## [29] "Hepatocellular Cancer|149"
## [30] "Gallbladder Ac|422"
```

Mising values handling strategy, step 2 - remove tumor samples with > 50% missing values.

```
pcbilDataMarkerSamplesTrimmed <- pcbilDataMarkerTrimmed[!rownames(pcbilDataM
arkerTrimmed) %in% samplesWithMissingGreater50, ]
```

Check the new status of missing values in the marker and sample filtered dataset

```
pcbilDataMarkerTrimmedMissingValues <- reportmiss(pcbilDataMarkerSamplesTrim
med)
```

```
##     variable nmiss   n propmiss
## 28 clin_diag     0 409     0.00
## 6       ck20    10 409     0.02
## 2        ck7    12 409     0.03
## 7        vim    23 409     0.06
## 5       ck19    38 409     0.09
## 21       p53    38 409     0.09
## 23      ki67    35 409     0.09
## 16     ca125    40 409     0.10
## 20      cdx2    42 409     0.10
## 17    ca19.9    51 409     0.12
## 9       muc2    52 409     0.13
## 10    muc5ac    52 409     0.13
## 8       muc1    56 409     0.14
## 15      pcea    56 409     0.14
## 11      muc6    65 409     0.16
## 4       ck18    69 409     0.17
## 3       ck17    74 409     0.18
## 26      cd56    74 409     0.18
## 1        ck5    91 409     0.22
## 19    wt1cyt    88 409     0.22
## 18    maspin    96 409     0.23
## 14      mcea    98 409     0.24
## 22       p63   104 409     0.25
## 25      chra   145 409     0.35
## 24     smad4   147 409     0.36
## 12    berep4   152 409     0.37
## 13       ema   151 409     0.37
## 27      cd10   153 409     0.37
```

```
# Total percentage missing data the marker and sample filtered dataset: (%)
round(pcbilDataMarkerTrimmedMissingValues, 0)
```

```
## [1] 18
```

```
missmap(pcbilDataMarkerSamplesTrimmed, main = "Missingness map pcbilDataMark
erSamplesTrimmed")
```

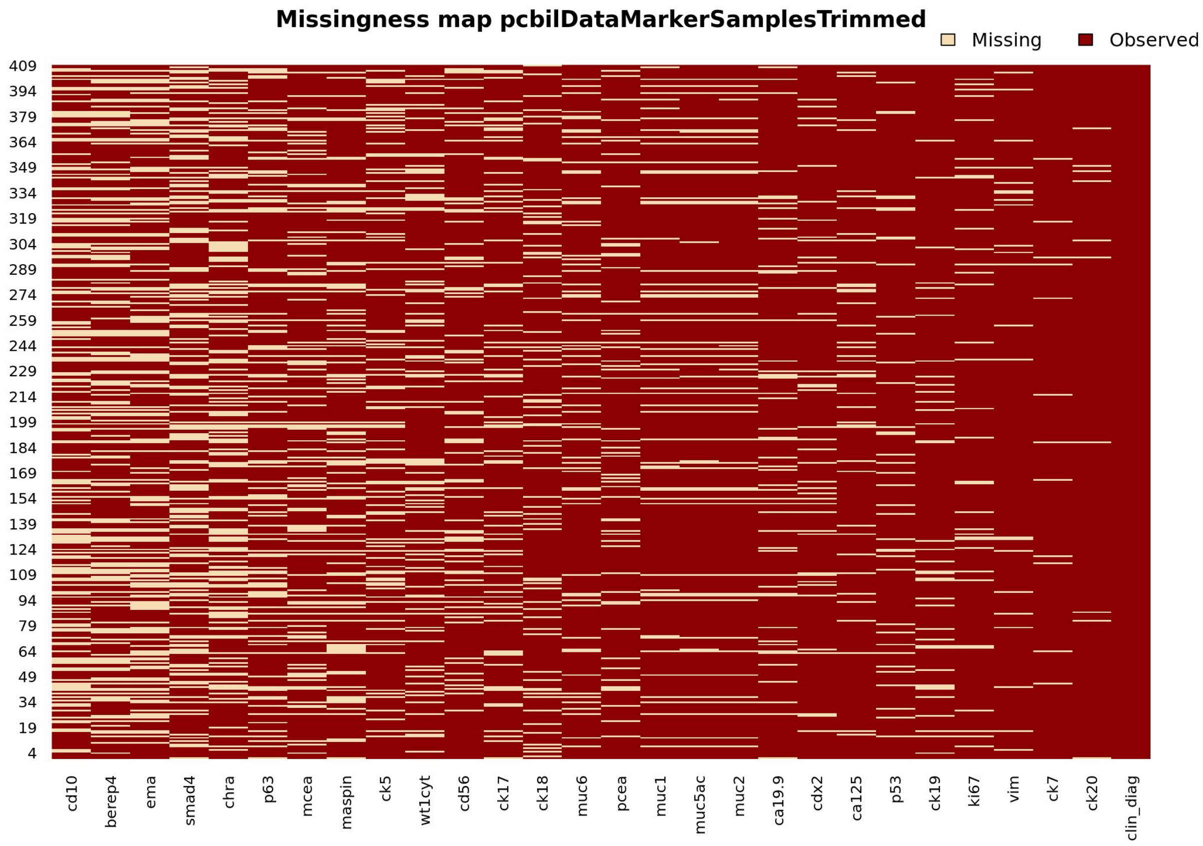

Missing values in the marker and sample filtered dataset, by tumor sample

```
reportmissperrow(pcbilDataMarkerSamplesTrimmed)
```

```
##
##   (0,10]  (10,20]  (20,30]  (30,40]  (40,50]  (50,60]  (60,70]  (70,80]
##       94      148       75       39       34        0        0        0
##  (80,90] (90,100]
##        0        0
```

```
## character(0)
```

Filtering of markers with > 40 % and rows with > 50 % missing data resulted in 18 % improvement of global data coverture

KNN-based imputation of remaining missing values

```
pcbilMatrixImputed <- impute.knn(as.matrix(pcbilDataMarkerSamplesTrimmed[1:2
7]))
pcbilDataImputed <- as.data.frame(pcbilMatrixImputed$data)
pcbilDataImputed$clin_diag <- pcbilDataMarkerSamplesTrimmed$clin_diag
```

Security check: missing values in the imputed dataset

```
pcbilDataImputedMissingValues <-reportmiss(pcbilDataImputed)
```

```
##     variable nmiss   n propmiss
## 1        ck5     0 409        0
## 2        ck7     0 409        0
## 3       ck17     0 409        0
## 4       ck18     0 409        0
## 5       ck19     0 409        0
## 6       ck20     0 409        0
## 7        vim     0 409        0
## 8       muc1     0 409        0
## 9       muc2     0 409        0
## 10    muc5ac     0 409        0
## 11      muc6     0 409        0
## 12    berep4     0 409        0
## 13       ema     0 409        0
## 14      mcea     0 409        0
## 15      pcea     0 409        0
## 16     ca125     0 409        0
## 17    ca19.9     0 409        0
## 18    maspin     0 409        0
## 19    wt1cyt     0 409        0
## 20      cdx2     0 409        0
## 21       p53     0 409        0
## 22       p63     0 409        0
## 23      ki67     0 409        0
## 24     smad4     0 409        0
## 25      chra     0 409        0
## 26      cd56     0 409        0
## 27      cd10     0 409        0
## 28 clin_diag     0 409        0
```

```
# Total percentage missing data in imputed dataset: %
round(pcbilDataImputedMissingValues, 0)
```

```
## [1] 0
```

```
missmap(pcbilDataImputed, main = "Missingness map pcbilDataImputed")
```

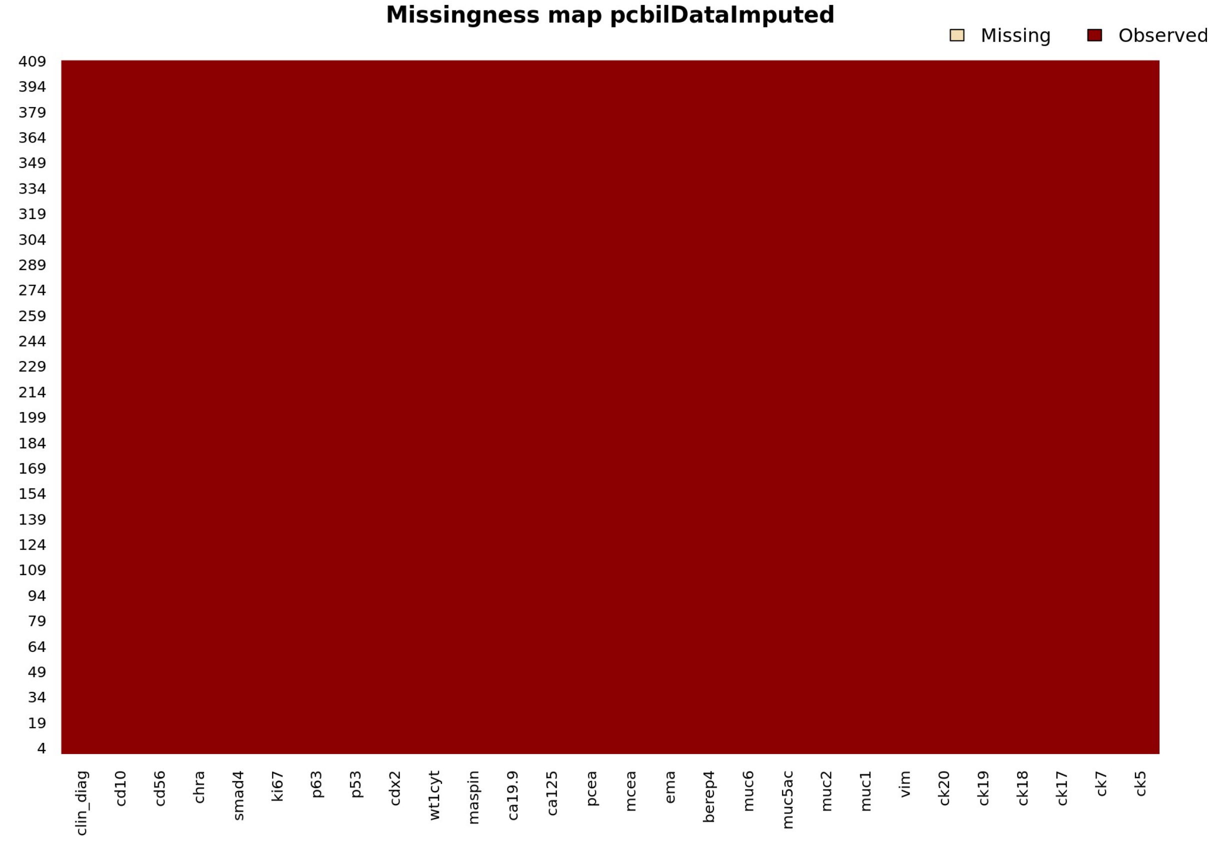

Save imputed data set

```
pcbilDataImputed$pad <- rownames(pcbilDataImputed)
write.csv(pcbilDataImputed, imputedDataFileName, row.names=F, na="")
```

– End of analysis of missing data & imputation –
