# Supplementary material for: Immunohistochemical Typing of Adenocarcinomas of the Pancreatobiliary System Improves Diagnosis and Prognostic Stratification
Source: PLoS One. 2016 Nov 9;11(11):e0166067. doi: 10.1371/journal.pone.0166067 (PMC5102456; doi:10.1371/journal.pone.0166067)
Supplement: S1 Table — (PDF) [file pone.0166067.s003.pdf]

**Supplementary Table 1:** Antibodies (n = 27) and staining protocols (BOND-MAX automated stainer) used for immunohistochemical analysis of the tumor samples.

| Antibody | Clone | Dilution | Pretreatment | Manufacturer-product code |
|---|---|---|---|---|
| CK5 | XM26 | 1:100 | H2 | NCL-L-CK5 |
| CK7 | RN7 | 1:200 | H2 | NCL-L-CK7-560 |
| CK17 | E3 | 1:25 | H2 | NCL-CK17 |
| CK18 | DC-10 | 1:100 | H2 | NCL-CK18 |
| CK19 | b170 | 1:100 | H2 | NCL-CK19 |
| CK20 | PW31 | 1:25 | H2 | NCL-L-CK20-561 |
| Vimentin | V9 | 1:1500 | H1 | Dako-M0725 |
| MUC1 | Ma695 | 1:50 | H1 | NCL-MUC1 |
| MUC2 | Ccp58 | 1:100 | H2 | NCL-MUC2 |
| MUC5AC | CLH2 | 1:50 | H2 | NCL-MUC-5Ac |
| MUC6 | CLH5 | 1:50 | H2 | NCL-MUC-6 |
| BerEp4 | Ber-EP4 | 1:100 | Enz 1 | Dako-M0804 |
| EMA | E29 | 1:100 | H1 | Dako-M0613 |
| M-CEA | 11-7 | 1:400 | H2 | Dako-M7072 |
| P-CEA | Polyclonal | 1:800 | Enz 1 | Dako-A0115 |
| CA125 | OV185:1 | 1:100 | H2 | NCl-L-Ca125 |
| CA19-9 | CA241:5:1:4 | 1:400 | H2 | NCL-L-CA19-9 |
| Maspin | EAW24 | 1:50 | H2 | NCL-Maspin |
| WT1 | 6F-H2 | 1:100 | H2 | Dako-M3561 |
| CDX2 | AMT28 | 1:25 | H2 | NCL-CDX2 |
| p53 | Do-7 | 1:300 | H1 | NCL-L-P53-D07 |
| p63 | DAK-p63 | 1:50 | H2 | Dako-M7317 |
| MIB1/KI-67 | MIB-1 | 1:150 | H2 | Dako-M7240 |
| SMAD4 | B-8 | 1:300 | H2 | Santa Cruz-sc-7966 |
| Chromogranin A | 5H7 | 1:100 | H1 | NCL-CHROM-430 |
| CD56 | CD564 | 1:50 | H2 | NCL-L-CD56-1B6 |
| CD10 | 56C6 | 1:75 | H2 | NCL-L-CD10-270 |

<u>Pretreatments BOND-MAX automated stainer:</u>

H1 = Bond Epitope Retrieval Solution 1 Citrate 20 Minutes

H2 = Bond Epitope Retrieval Solution 2 EDTA 20 Minutes

Enz 1 = 50 μl enzyme + 7 ml Bond enzyme solution

<u>Antibody manufacturers</u>:

NCL: Novocastra Leica Biosystems Ltd, Newcastle Upon Tyne, United Kingdom

Dako: Dako, Glostrup, Denmark

Santa Cruz: Santa Cruz Biotechnology, Inc., Dallas, U.S.A.
