# Supplementary material for: Immunohistochemical Typing of Adenocarcinomas of the Pancreatobiliary System Improves Diagnosis and Prognostic Stratification
Source: PLoS One. 2016 Nov 9;11(11):e0166067. doi: 10.1371/journal.pone.0166067 (PMC5102456; doi:10.1371/journal.pone.0166067)
Supplement: S2 Fig — (PDF) [file pone.0166067.s004.pdf]

Supplementary Figure 2

Immunoprofiling of adenocarcinomas of the pancreatobiliary tree

Simple immunohistograms - for the immunohistochemical tumor types and significant markers

Generated on: 2016-03-03 17:42:50

Import required packages

```
# Build pre-requisites
# install.packages("ggplot2")
# install.packages("gridExtra")
# install.packages("scales")
# install.packages("plyr")
# install.packages("reshape")

library(ggplot2)
library(gridExtra)
```

```
## Loading required package: grid
```

```
library(scales)
library(plyr)
library(reshape)
```

```
##
## Attaching package: 'reshape'
##
## The following objects are masked from 'package:plyr':
##
##     rename, round_any
```

Configure file names

```
#myWorkDirectory <- "~/Workspace/research/pcbil/"
myWorkDirectory <- "/home/guest/pcbil/"
clusteredPcbilFileName <- paste(myWorkDirectory, "data_analysis/tidy_dataset
s/pcbil_clustered.csv", sep="")
```

Import clustered dataset

```
pcbilDataClustered <- read.csv(file = clusteredPcbilFileName, row.names = 3
0, colClasses= c(rep("numeric",27), rep("factor",2), "character"), na.string
s = "",quote="\"" )
```

Simple immunohistograms - for the immmunohistochemical tumor types and significant markers

```r
ihcTypes <- c("extrahepatic pancreatobiliary", "intestinal", "intrahepatic c
holangiocarcinoma", "hepatocellular carcinoma")
colorsIhcTypes <- c("red", "blueviolet", "blue", "green")

diffMarkers <- c("ck7", "ck17", "ck19", "ck20", "vim", "muc1", "muc2", "muc5
ac", "berep4", "mcea", "pcea", "ca19.9", "ca125", "maspin", "cdx2", "p53",
"smad4", "cd56", "wt1cyt")
diffMarkersQualit <- c(paste(diffMarkers, "_qualit", sep=''), 'cluster')
types2Markers <- list("Intermediate filaments" = c("ck7", "ck17", "ck19", "c
k20", "vim"),
                      "Mucins" = c("muc1", "muc2", "muc5ac", "muc6"),
                      "Epithelial markers" = c("berep4", "mcea", "pcea", "ca
19.9", "ca125",  "maspin"),
                      "Nuclear markers" = c("cdx2", "p53", "smad4"),
                      "Other" = c("cd56", "wt1cyt"))
# Function to retrieve the type of a marker by its name
get_marker_type <- function(marker) {
  for(aType in names(types2Markers)) {
       if(marker %in% types2Markers[[aType]]) {
         return(aType)
       }
  }
  return("error")
}

cont <- 1
while(cont <= length(ihcTypes)) {

    # Subset current ihc type, initially with quantitative ihc values
    pcbilDataQuantit <- subset(pcbilDataClustered, pcbilDataClustered$cluste
r == ihcTypes[cont])

  # Recode for significant markers quantitative -> qualitative values: pos/n
eg, cut-off 10
  pcbilDataQualit <- pcbilDataQuantit
  for(aMarker in diffMarkers) {
    quantitValues <- pcbilDataQualit[, aMarker]
    qualitValues <- cut(quantitValues, c(-1, 10, 100), include.lowest=TRUE,
ordered_result=TRUE, labels = c('N', 'P'))
    pcbilDataQualit[, paste(aMarker, "_qualit", sep='')] <- qualitValue
s
  }
  # Filter-out quantitative data
  pcbilDataQualit <- pcbilDataQualit[, diffMarkersQualit]

  # Calculate proportions (%) of each value (N, P) for evvery qualitative ma
rker
  pcbilDataProportions <- ddply(pcbilDataQualit, .(cluster), summarise,
                             ck7 = round(prop.table(table(ck7_qualit)),2) *1
00,
                             ck17 = round(prop.table(table(ck17_qualit)),2)
* 100,
```

```
                              ck19 = round(prop.table(table(ck19_qualit)),2)
*100,
                              ck20 = round(prop.table(table(ck20_qualit)),2)
* 100,
                              vim = round(prop.table(table(vim_qualit)),2) *
100,
                              muc1 = round(prop.table(table(muc1_qualit)),2)
* 100,
                              muc2 = round(prop.table(table(muc2_qualit)),2)
* 100,
                              muc5ac = round(prop.table(table(muc5ac_quali
t)),2) * 100,
                              berep4 = round(prop.table(table(berep4_quali
t)),2) *100,
                              ca19.9 = round(prop.table(table(ca19.9_quali
t)),2) * 100,
                              ca125 = round(prop.table(table(ca125_qualit)),
2) * 100,
                              mcea = round(prop.table(table(mcea_qualit)),2)
* 100,
                              pcea = round(prop.table(table(pcea_qualit)),2)
* 100,
                              maspin = round(prop.table(table(maspin_quali
t)),2) *100,
                              p53 = round(prop.table(table(p53_qualit)),2) *
100,
                              cdx2 = round(prop.table(table(cdx2_qualit)),2)
* 100,
                              cd56 = round(prop.table(table(cd56_qualit)),2)
* 100,
                              wt1cyt = round(prop.table(table(wt1cyt_quali
t)),2) * 100,
                              smad4 = round(prop.table(table(smad4_qualit)),
2) * 100,
                              score_cats = names(table(ck7_qualit)))

  # Relevel qualitative categories so N is first
  pcbilDataProportions$score_cats <- factor(pcbilDataProportions$score_cat
s, levels = c('N', 'P'))

  # Filter-out negative (N) values
  pcbilDataProportions <- subset(pcbilDataProportions, score_cats == "P")
  # We have now a wide format dataset

  # For ggplot faceting, we need to transfor the data set to the long format
  pcbilDataProportionsLong <- melt(pcbilDataProportions, measure.vars= diffM
arkers, variable_name="marker")

  # Add to long data set an additional column for marker type and relevel
  pcbilDataProportionsLong$marker_type <- sapply(pcbilDataProportionsLong$ma
rker, get_marker_type)
  pcbilDataProportionsLong$marker_type <- factor(pcbilDataProportionsLong$ma
```

```
rker_type, levels = c('Intermediate filaments', 'Mucins', 'Epithelial marker
s',  'Nuclear markers', 'Other'))

  # Relevel also markers so they are displayed in a logical order
  pcbilDataProportionsLong$marker <- factor(pcbilDataProportionsLong$marke
r, levels = c(types2Markers[[1]], types2Markers[[2]], types2Markers[[3]], ty
pes2Markers[[4]], types2Markers[[5]]))


  ihcPlot <- ggplot(pcbilDataProportionsLong, aes(marker, value)) + geom_bar
(stat="identity", position="identity", fill= colorsIhcTypes[cont]) + facet_w
rap(~ marker_type, ncol=2, scales="free_x") + xlab("Markers") + ylab("% of t
umors") + ggtitle(paste("Immunohistogram for type: ",ihcTypes[cont], sep
=""))

  labs <- c("")
  g <- tableGrob(labs, gpar.coretext = gpar(fontsize = 10), gpar.corefill =
gpar(col = "white"))   # core.just='left'
  grid.arrange(ihcPlot, g, nrow = 1, ncol = 2, widths = (c(6, 1)))

  cont <- cont + 1
}
```

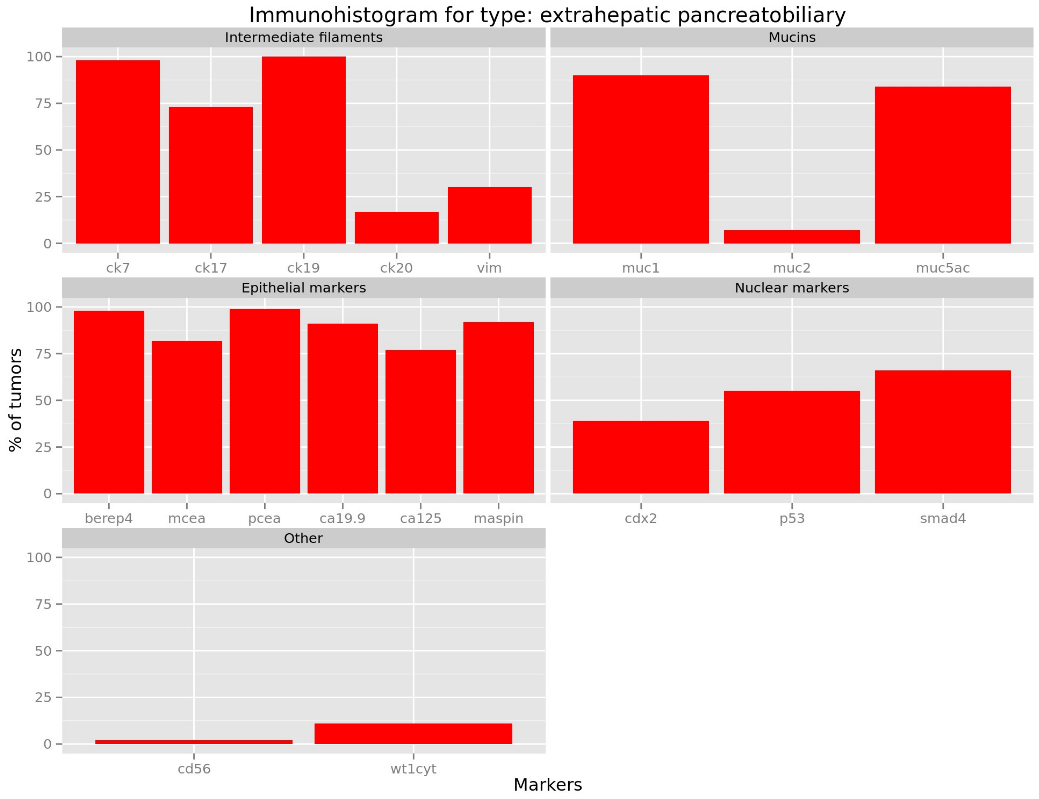

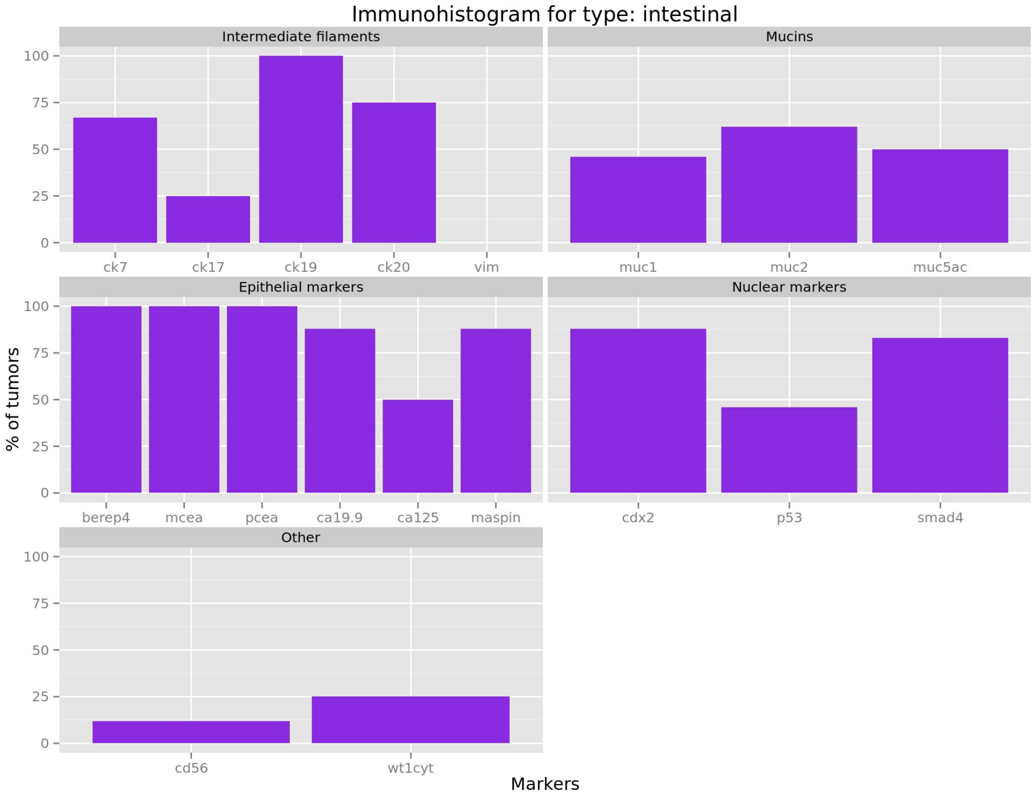
Immunohistogram for type: intestinal
Intermediate filaments
Mucins
Epithelial markers
Nuclear markers
Other
% of tumors
Markers
0
25
50
75
100
ck7
ck17
ck19
ck20
vim
muc1
muc2
muc5ac
berep4
mcea
pcea
ca19.9
ca125
maspin
cdx2
p53
smad4
cd56
wt1cyt

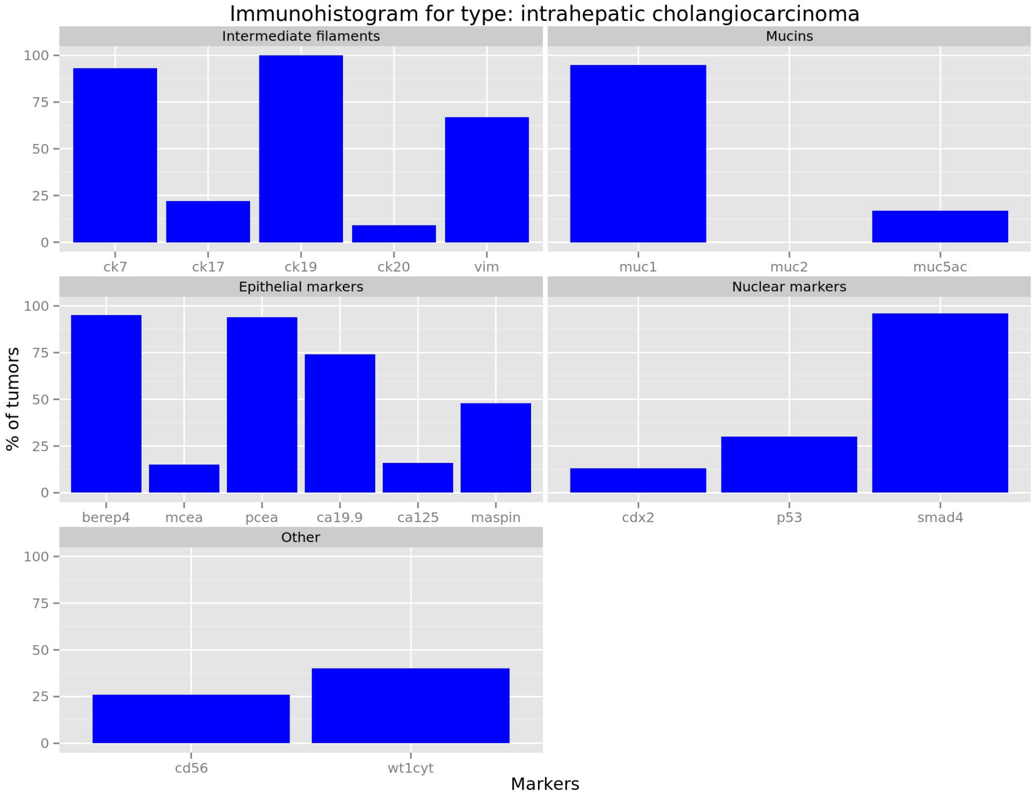
Immunohistogram for type: intrahepatic cholangiocarcinoma
Intermediate filaments
Mucins
Epithelial markers
Nuclear markers
Other
% of tumors
Markers
0
25
50
75
100
ck7
ck17
ck19
ck20
vim
muc1
muc2
muc5ac
berep4
mcea
pcea
ca19.9
ca125
maspin
cdx2
p53
smad4
cd56
wt1cyt

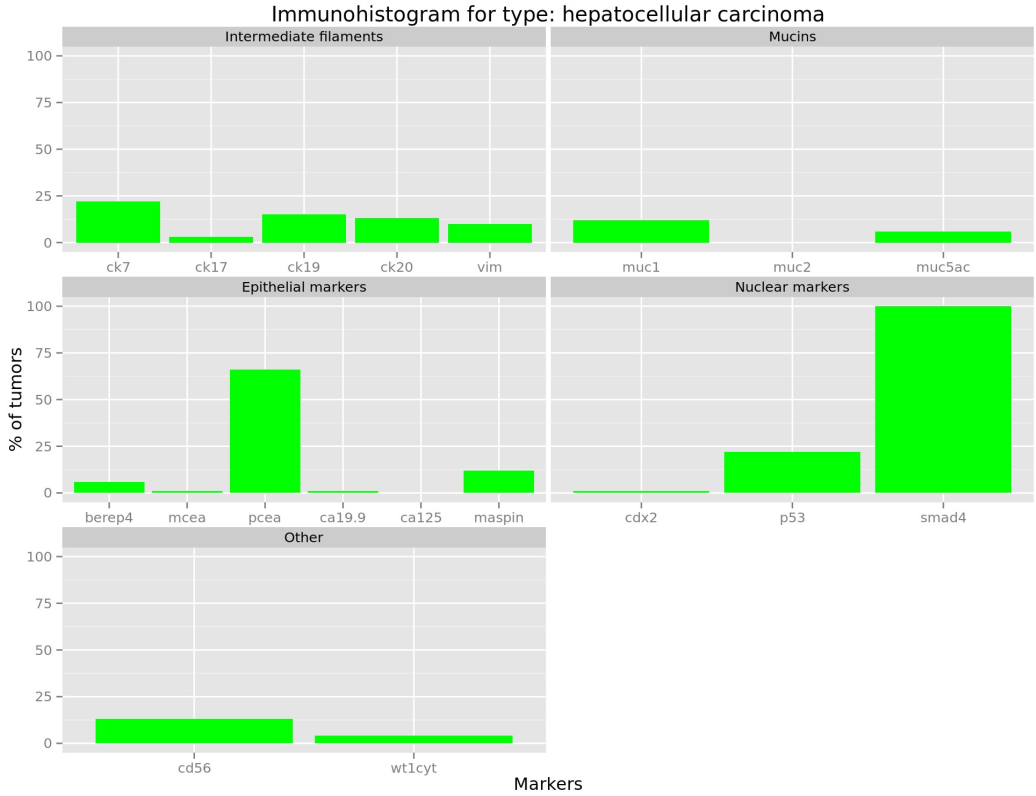

– End of simple immunohistograms –
