# Supplementary material for: Immunohistochemical Typing of Adenocarcinomas of the Pancreatobiliary System Improves Diagnosis and Prognostic Stratification
Source: PLoS One. 2016 Nov 9;11(11):e0166067. doi: 10.1371/journal.pone.0166067 (PMC5102456; doi:10.1371/journal.pone.0166067)
Supplement: S2 File — (PDF) [file pone.0166067.s005.pdf]

Supplementary File 2

Immunoprofiling of adenocarcinomas of the pancreatobiliary tree

Differential expression analysis

Generated on: 2016-03-03 17:28:09

Import required packages

```
# Build pre-requisites
# install.packages("pander")
# install.packages("samr")
# source("http://bioconductor.org/biocLite.R")
# biocLite("multtest")
# biocLite("RankProd")

library(pander)
library(multtest)
```

```
## Loading required package: Biobase
## Loading required package: BiocGenerics
## Loading required package: parallel
##
## Attaching package: 'BiocGenerics'
##
## The following objects are masked from 'package:parallel':
##
##     clusterApply, clusterApplyLB, clusterCall, clusterEvalQ,
##     clusterExport, clusterMap, parApply, parCapply, parLapply,
##     parLapplyLB, parRapply, parSapply, parSapplyLB
##
## The following object is masked from 'package:stats':
##
##     xtabs
##
## The following objects are masked from 'package:base':
##
##     Filter, Find, Map, Position, Reduce, anyDuplicated, append,
##     as.data.frame, as.vector, cbind, colnames, duplicated, eval,
##     evalq, get, intersect, is.unsorted, lapply, mapply, match,
##     mget, order, paste, pmax, pmax.int, pmin, pmin.int, rank,
##     rbind, rep.int, rownames, sapply, setdiff, sort, table,
##     tapply, union, unique, unlist
##
## Welcome to Bioconductor
##
##     Vignettes contain introductory material; view with
##     'browseVignettes()'. To cite Bioconductor, see
##     'citation("Biobase")', and for packages 'citation("pkgname")'.
```

```
library(RankProd)
library(samr)
```

```
## Loading required package: impute
## Loading required package: matrixStats
## matrixStats v0.14.2 (2015-06-23) successfully loaded. See ?matrixStats fo
r help.
##
## Attaching package: 'matrixStats'
##
## The following objects are masked from 'package:Biobase':
##
##     anyMissing, rowMedians
```

Configure file names

```
#myWorkDirectory <- "~/Workspace/research/pcbil/"
myWorkDirectory <- "/home/guest/pcbil/"
clusteredPcbilFileName <- paste(myWorkDirectory, "data_analysis/tidy_dataset
s/pcbil_clustered.csv", sep="")
```

Import clustered dataset

```
pcbilDataClustered <- read.csv(file = clusteredPcbilFileName, row.names = 3
0, colClasses= c(rep("numeric",27), rep("factor",2), "character"), na.string
s = "?",quote="\"" )
```

Alpha (cut-off) value for statistical significance - used in RankProd and SAM

```
alpha <- 0.001
```

Configure differential diagnosis to evaluate and seeds

```
diffDiagn1 <- c("extrahepatic pancreatobiliary", "extrahepatic pancreatobili
ary", "intrahepatic cholangiocarcinoma")
diffDiagn2 <- c("intrahepatic cholangiocarcinoma", "intestinal", "hepatocell
ular carcinoma")

seeds  <- c(1097695, 434343,12345)
```

Differential expresion analysis - multtest

```
multtestResults <- list()

cont <- 1
while(cont <= length(diffDiagn1)) {

  # Subset tumor clusters to be compared
  pcbilDataTumorClusters <- subset(pcbilDataClustered, pcbilDataClustered$cl
uster == diffDiagn1[cont] | pcbilDataClustered$cluster == diffDiagn2[cont])

  aMTP <- MTP(X = t(pcbilDataTumorClusters[1:27]), Y = pcbilDataTumorCluster
s$cluster, test="t.twosamp.unequalvar", robust=TRUE, seed = seeds[cont])

  print(plot(aMTP))

  aMulttestResult <- data.frame(aMTP@rawp[aMTP@adjp <= alpha], aMTP@adjp[aMT
P@adjp <= alpha])
  aMulttestResult <- cbind(rownames(aMulttestResult), aMulttestResult)
  meansDiffDiagn1 <- c()
  meansDiffDiagn2 <- c()
  meanDiffs <- c()
  for(marker in aMulttestResult[,1]) {
    meansDiffDiagn1 <- c(meansDiffDiagn1, mean(pcbilDataTumorClusters[pcbilD
ataTumorClusters$cluster==diffDiagn1[cont], marker]))
    meansDiffDiagn2 <- c(meansDiffDiagn2, mean(pcbilDataTumorClusters[pcbilD
ataTumorClusters$cluster==diffDiagn2[cont], marker]))
  }
  meanDiffs <- round((meansDiffDiagn1 - meansDiffDiagn2),2)
  aMulttestResult <- cbind(aMulttestResult, meansDiffDiagn1, meansDiffDiagn
2, meanDiffs)
  colnames(aMulttestResult) <- c("marker", "raw-pval","adj-pval", "mean clas
s 1", "mean class 2", "mean diffs")

  aMulttestResult <- aMulttestResult[order(-aMulttestResult[6]), ]
  rownames(aMulttestResult) <- NULL

  caption_str <- paste("class 1: ", diffDiagn1[cont], " | class 2: ", diffDi
agn2[cont], sep="")
  pandoc.table(aMulttestResult, caption= caption_str, style="grid", split.ta
bles=100, justify="right")

  multtestResults <- append(multtestResults, list(as.vector(aMulttestResult
[,1])))

  cont <- cont + 1
}
```

```
## running bootstrap...
## iteration = 100 200 300 400 500 600 700 800 900 1000
```

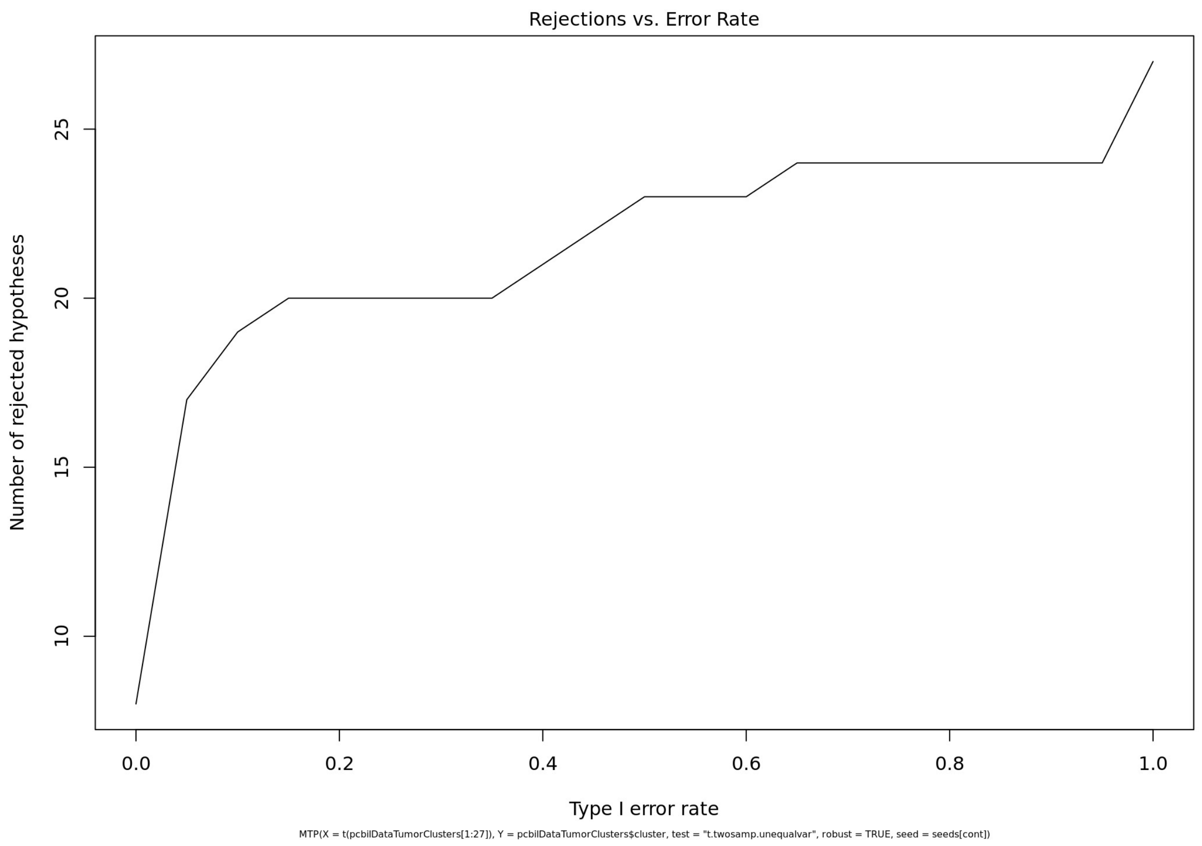
Rejections vs. Error Rate
Number of rejected hypotheses
Type I error rate
MTP(X = t(pcbilDataTumorClusters[1:27]), Y = pcbilDataTumorClusters$cluster, test = "t.twosamp.unequalvar", robust = TRUE, seed = seeds[cont])

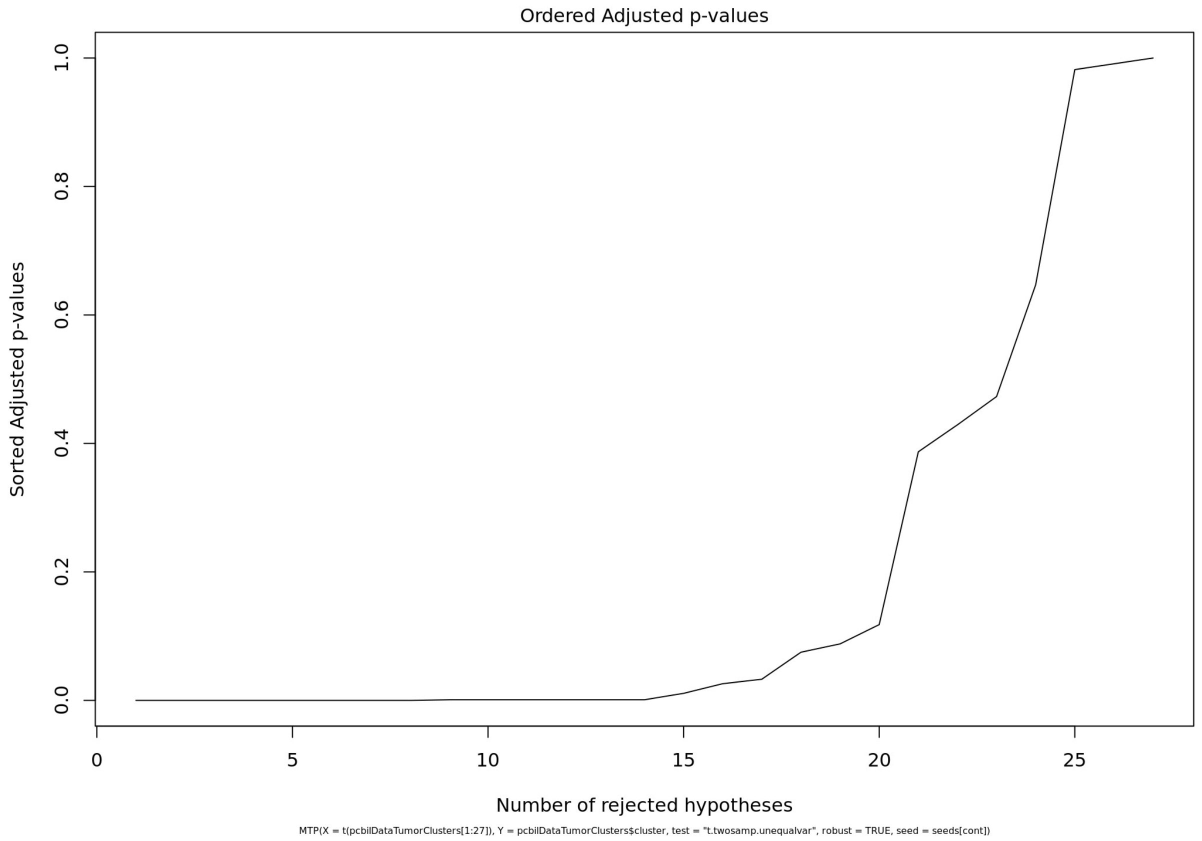
Ordered Adjusted p-values
Sorted Adjusted p-values
Number of rejected hypotheses
MTP(X = t(pcbilDataTumorClusters[1:27]), Y = pcbilDataTumorClusters$cluster, test = "t.twosamp.unequalvar", robust = TRUE, seed = seeds[cont])

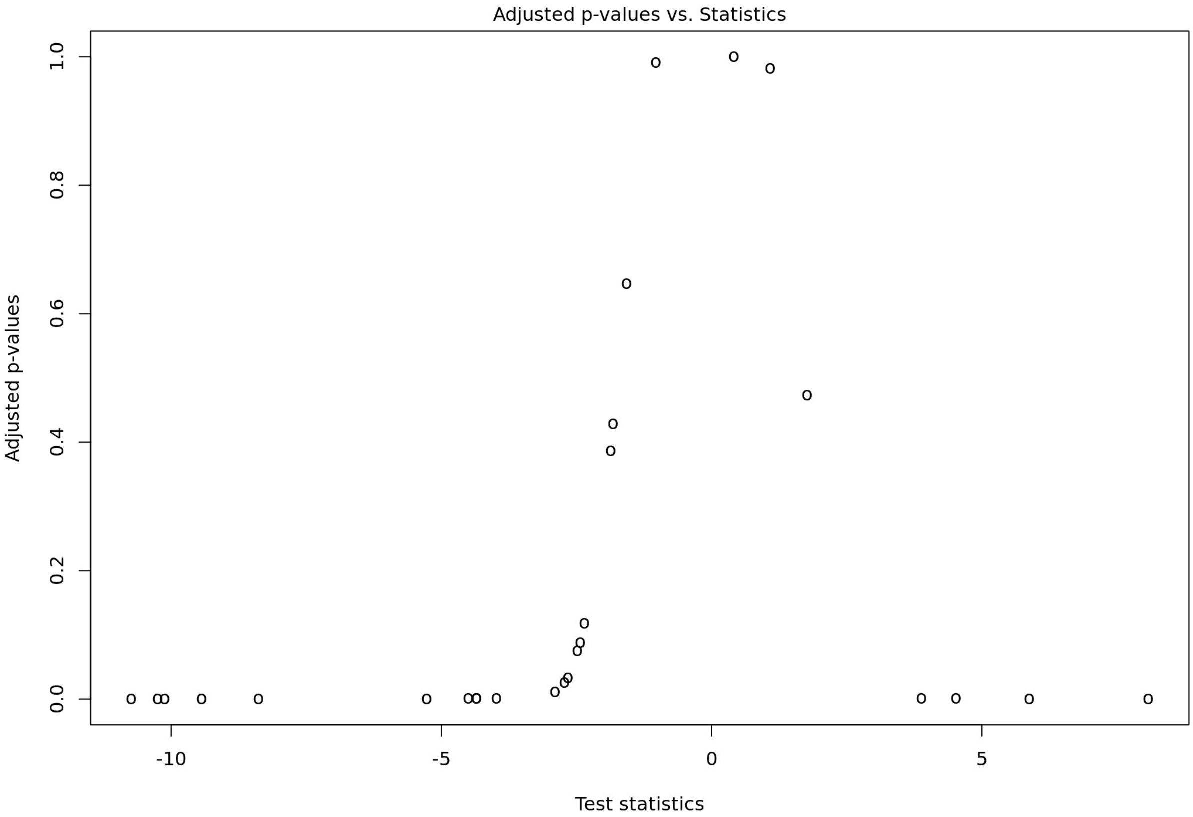

MTP(X = t(pcbilDataTumorClusters[1:27]), Y = pcbilDataTumorClusters$cluster, test = "t.twosamp.unequalvar", robust = TRUE, seed = seeds[cont])

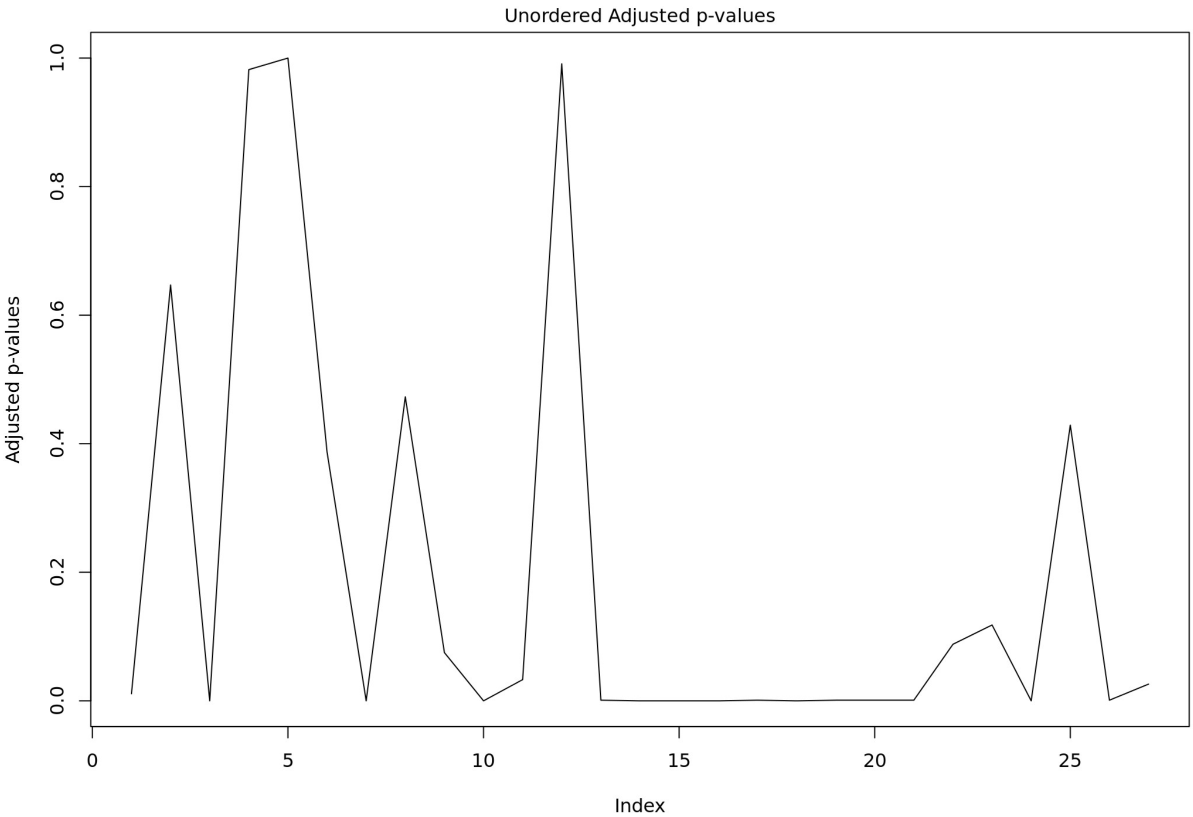

MTP(X = t(pcbilDataTumorClusters[1:27]), Y = pcbilDataTumorClusters$cluster, test = "t.twosamp.unequalvar", robust = TRUE, seed = seeds[cont])

```
## NULL
##
##
## +----------+-----------+-----------+---------------+---------------+-
-------------+
## |   marker |   raw-pval |   adj-pval |   mean class 1 |   mean class 2
|   mean diffs |
## +==========+===========+===========+===============+===============+=
=============+
## |   maspin |          0 |          0 |          73.85 |          22.18
|        51.67 |
## +----------+-----------+-----------+---------------+---------------+-
-------------+
## |    ca125 |          0 |          0 |          52.42 |          4.085
|        48.33 |
## +----------+-----------+-----------+---------------+---------------+-
-------------+
## |     mcea |          0 |          0 |          48.53 |          3.349
|        45.19 |
## +----------+-----------+-----------+---------------+---------------+-
-------------+
## |   muc5ac |          0 |          0 |          51.72 |          7.377
|        44.34 |
## +----------+-----------+-----------+---------------+---------------+-
-------------+
## |     ck17 |          0 |          0 |          33.68 |          6.428
|        27.26 |
## +----------+-----------+-----------+---------------+---------------+-
-------------+
## |      p53 |          0 |      0.001 |          42.23 |             18
|        24.22 |
## +----------+-----------+-----------+---------------+---------------+-
-------------+
## |   ca19.9 |          0 |      0.001 |          75.54 |          51.99
|        23.55 |
## +----------+-----------+-----------+---------------+---------------+-
-------------+
## |     pcea |          0 |          0 |           87.7 |          66.18
|        21.52 |
## +----------+-----------+-----------+---------------+---------------+-
-------------+
## |      ema |          0 |      0.001 |          91.12 |          79.47
|        11.65 |
## +----------+-----------+-----------+---------------+---------------+-
-------------+
## |     cdx2 |          0 |      0.001 |          16.02 |          6.353
|         9.67 |
## +----------+-----------+-----------+---------------+---------------+-
-------------+
## |     cd56 |          0 |      0.001 |          0.638 |          10.26
|        -9.63 |
## +----------+-----------+-----------+---------------+---------------+-
```

```
-------------+
## |   wt1cyt |          0 |      0.001 |           3.82 |          15.67
|       -11.85 |
## +----------+------------+------------+----------------+----------------+-
-------------+
## |      vim |          0 |          0 |          15.93 |          42.93
|          -27 |
## +----------+------------+------------+----------------+----------------+-
-------------+
## |    smad4 |          0 |          0 |          41.98 |          85.08
|        -43.1 |
## +----------+------------+------------+----------------+----------------+-
-------------+
##
## Table: class 1: extrahepatic pancreatobiliary | class 2: intrahepatic cho
langiocarcinoma
##
## running bootstrap...
## iteration = 100 200 300 400 500 600 700 800 900 1000
```

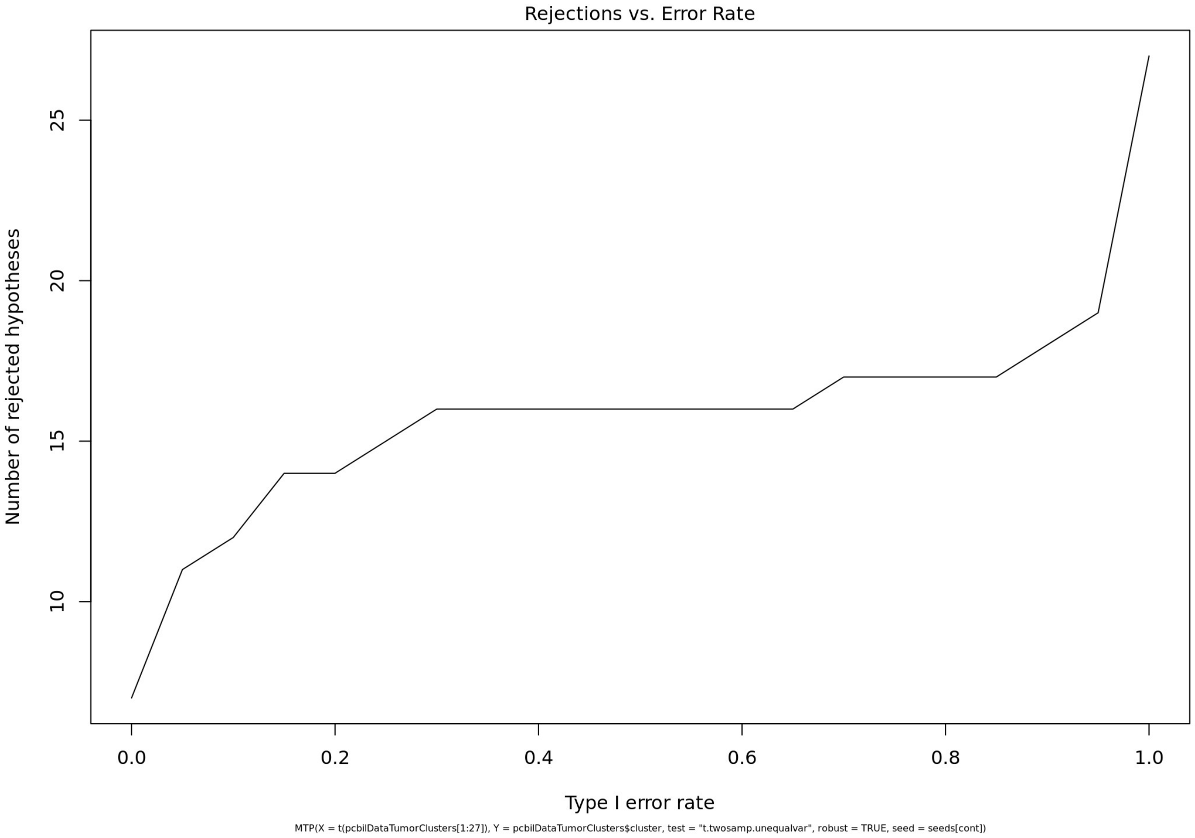

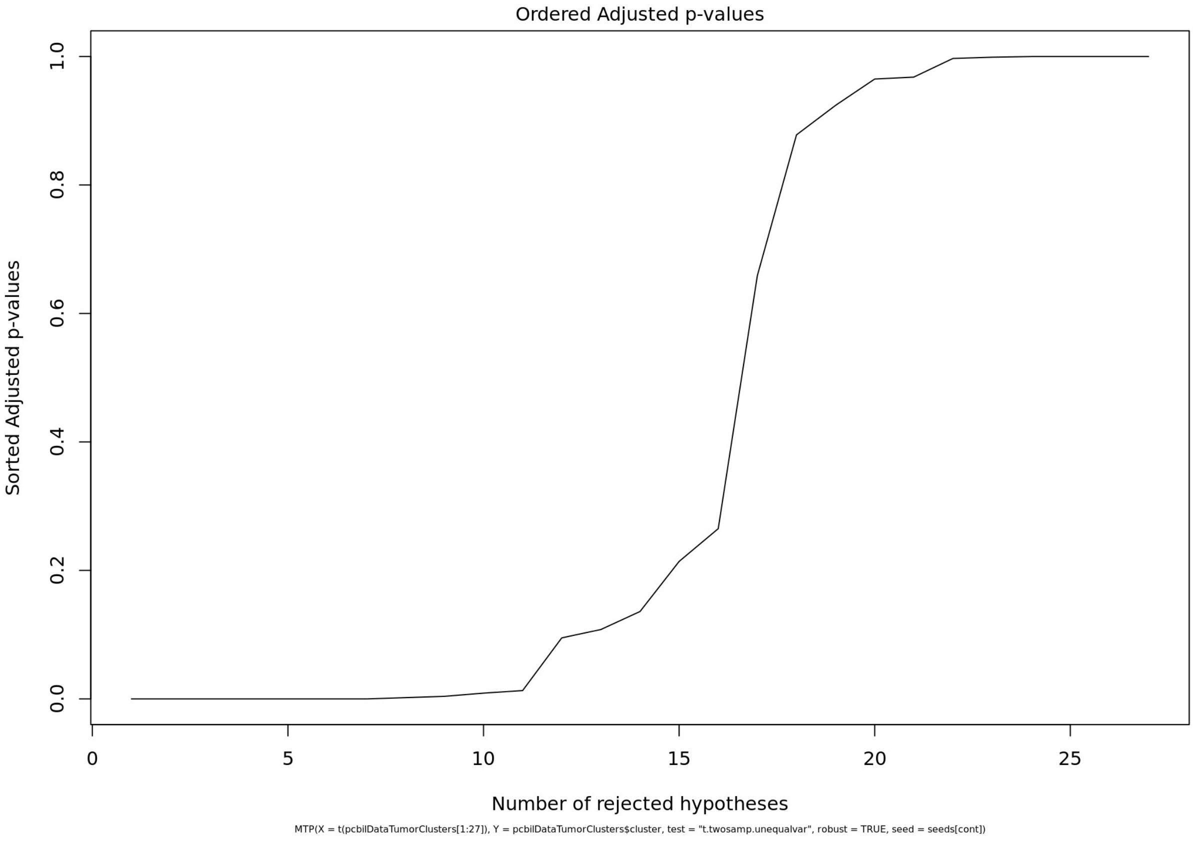
Ordered Adjusted p-values
Sorted Adjusted p-values
0.0
0.2
0.4
0.6
0.8
1.0
0
5
10
15
20
25
Number of rejected hypotheses
MTP(X = t(pcbilDataTumorClusters[1:27]), Y = pcbilDataTumorClusters$cluster, test = "t.twosamp.unequalvar", robust = TRUE, seed = seeds[cont])

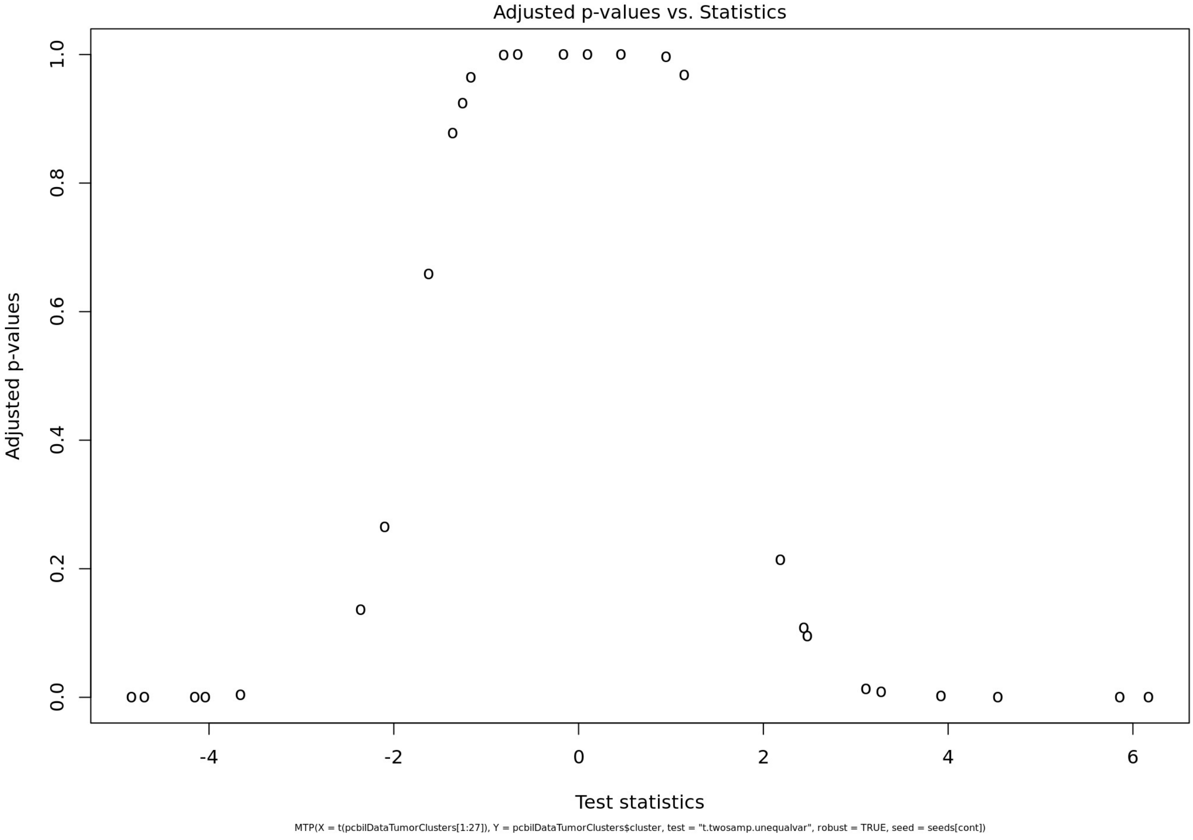
Adjusted p-values vs. Statistics
Adjusted p-values
0.0
0.2
0.4
0.6
0.8
1.0
-4
-2
0
2
4
6
Test statistics
MTP(X = t(pcbilDataTumorClusters[1:27]), Y = pcbilDataTumorClusters$cluster, test = "t.twosamp.unequalvar", robust = TRUE, seed = seeds[cont])

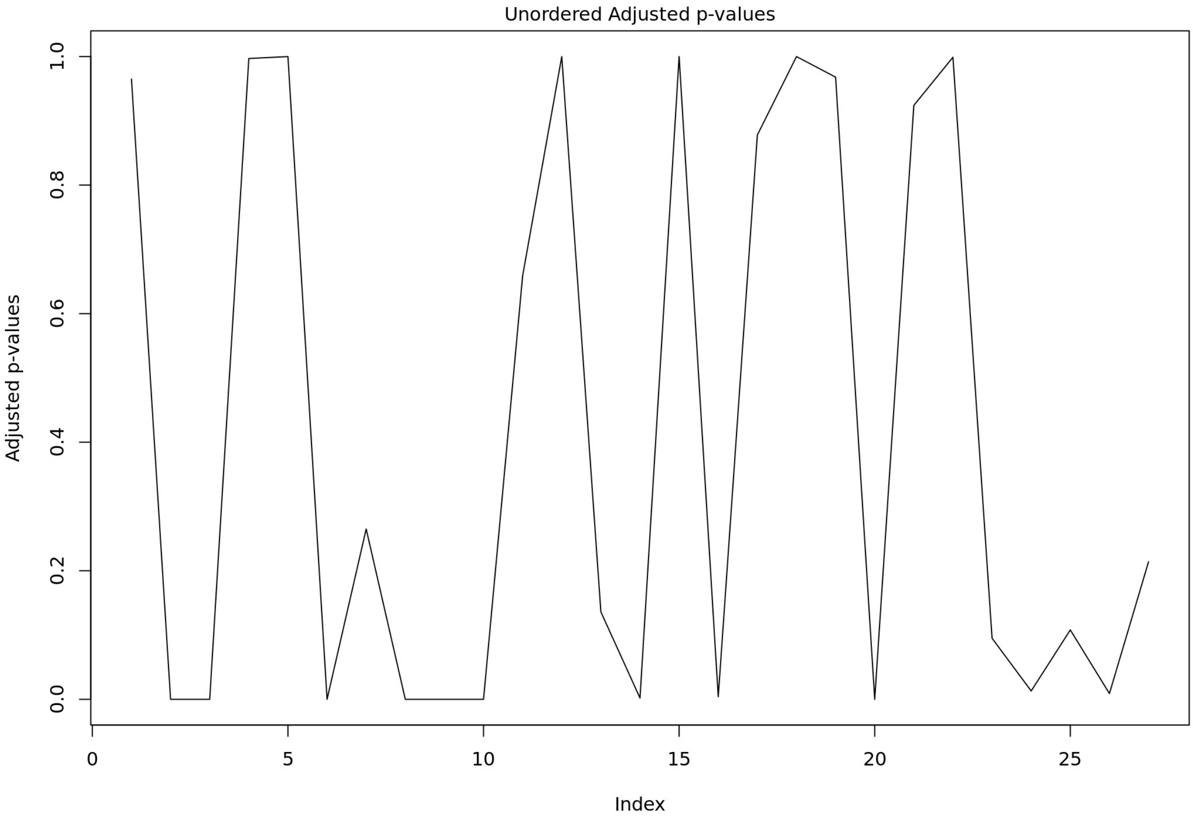

MTP(X = t(pcbilDataTumorClusters[1:27]), Y = pcbilDataTumorClusters$cluster, test = "t.twosamp.unequalvar", robust = TRUE, seed = seeds[cont])

```
## NULL
##
##
## +----------+------------+------------+----------------+----------------+-
-------------+
## |  marker  |  raw-pval  |  adj-pval  |  mean class 1  |  mean class 2
|  mean diffs  |
## +==========+============+============+================+================+=
=============+
## |    ck7   |          0 |          0 |          88.43 |          46.28
|       42.15  |
## +----------+------------+------------+----------------+----------------+-
-------------+
## |   muc1   |          0 |          0 |             61 |          28.26
|       32.74  |
## +----------+------------+------------+----------------+----------------+-
-------------+
## |  muc5ac  |          0 |          0 |          51.72 |          22.96
|       28.76  |
## +----------+------------+------------+----------------+----------------+-
-------------+
## |   ck17   |          0 |          0 |          33.68 |          8.125
|       25.56  |
## +----------+------------+------------+----------------+----------------+-
-------------+
## |   muc2   |          0 |          0 |          2.026 |          32.15
|      -30.13  |
## +----------+------------+------------+----------------+----------------+-
-------------+
## |   ck20   |          0 |          0 |           7.17 |          58.75
|      -51.58  |
## +----------+------------+------------+----------------+----------------+-
-------------+
## |   cdx2   |          0 |          0 |          16.02 |          75.08
|      -59.05  |
## +----------+------------+------------+----------------+----------------+-
-------------+
##
## Table: class 1: extrahepatic pancreatobiliary | class 2: intestinal
##
## running bootstrap...
## iteration = 100 200 300 400 500 600 700 800 900 1000
```

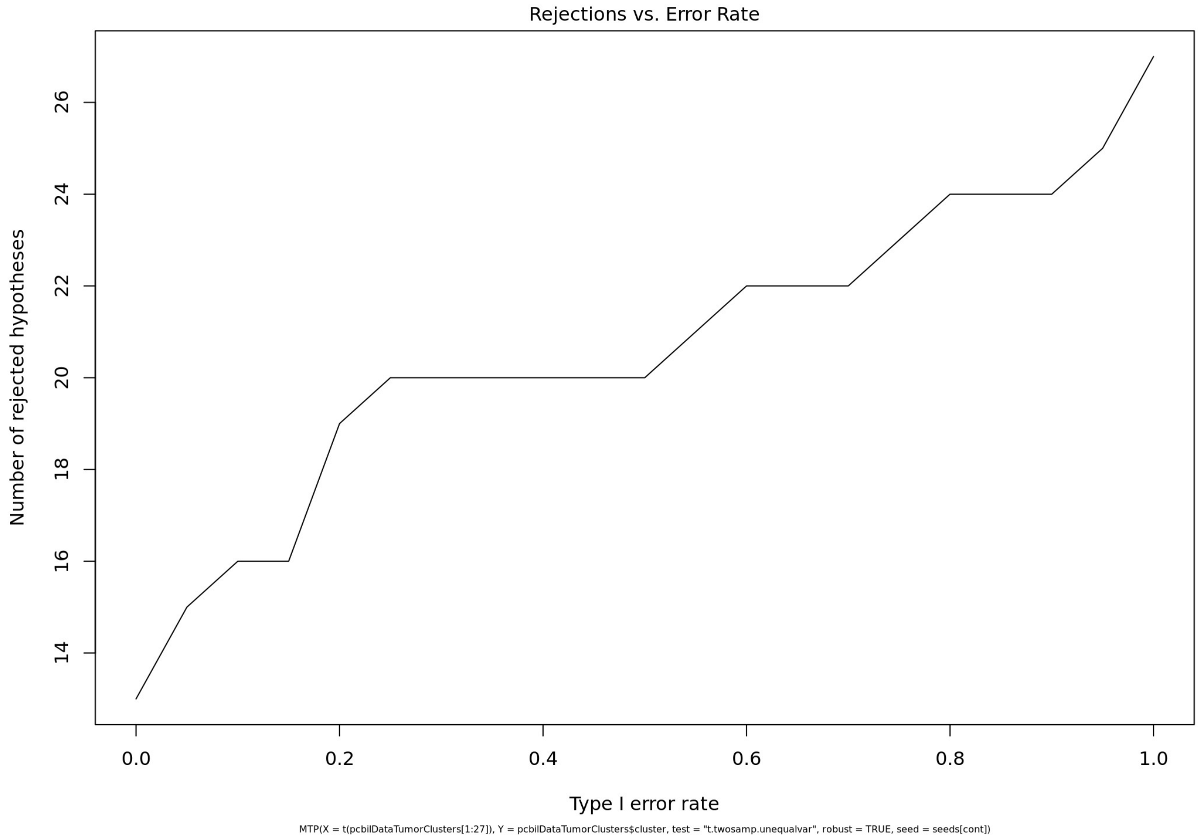
Rejections vs. Error Rate
Number of rejected hypotheses
Type I error rate
14 16 18 20 22 24 26
0.0 0.2 0.4 0.6 0.8 1.0
MTP(X = t(pcbilDataTumorClusters[1:27]), Y = pcbilDataTumorClusters$cluster, test = "t.twosamp.unequalvar", robust = TRUE, seed = seeds[cont])

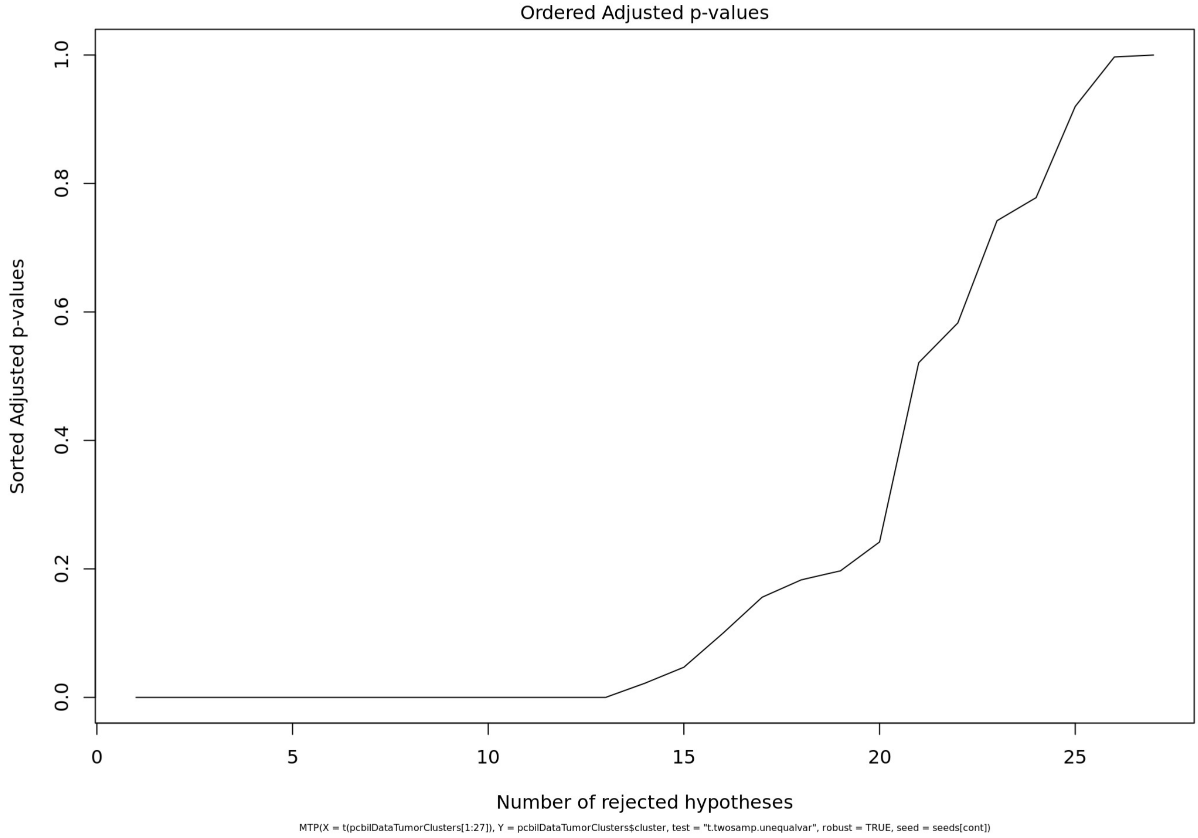
Ordered Adjusted p-values
Sorted Adjusted p-values
Number of rejected hypotheses
0.0 0.2 0.4 0.6 0.8 1.0
0 5 10 15 20 25
MTP(X = t(pcbilDataTumorClusters[1:27]), Y = pcbilDataTumorClusters$cluster, test = "t.twosamp.unequalvar", robust = TRUE, seed = seeds[cont])

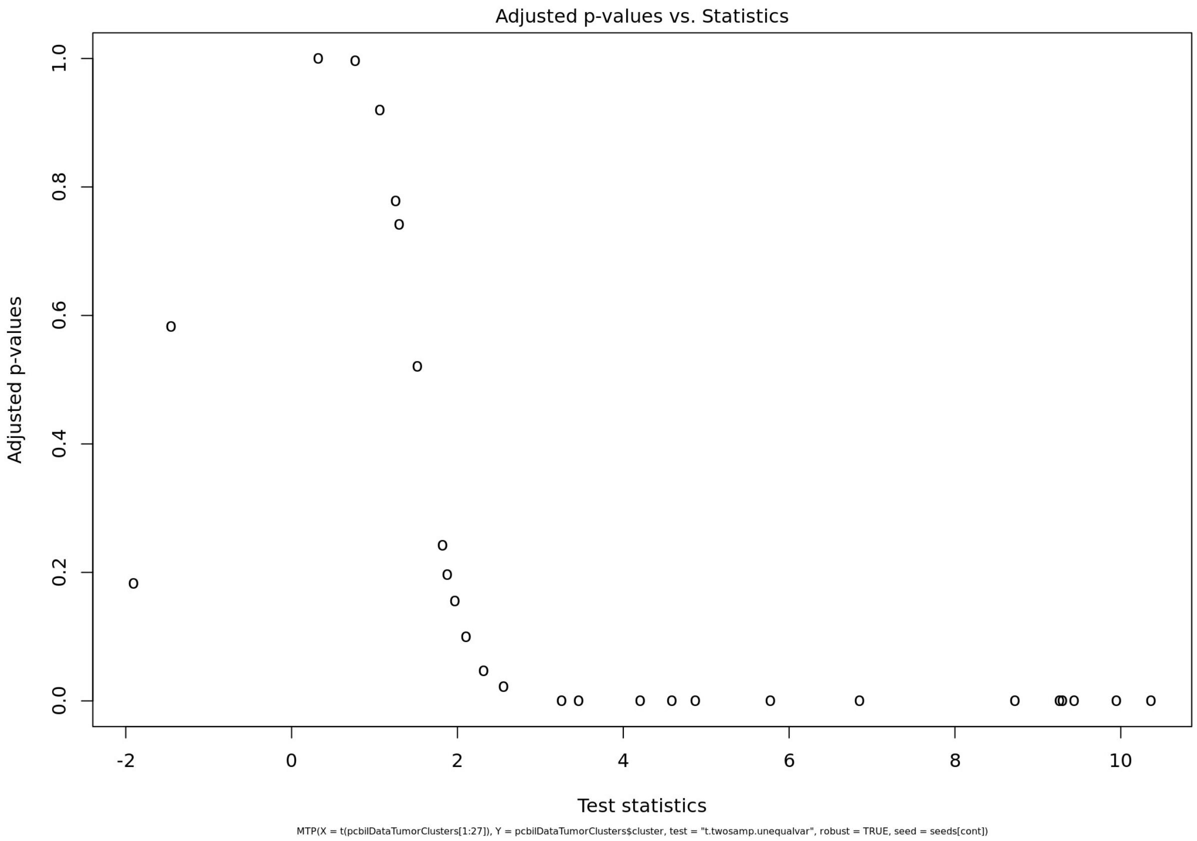
Adjusted p-values vs. Statistics
Adjusted p-values
0.0
0.2
0.4
0.6
0.8
1.0
-2
0
2
4
6
8
10
Test statistics
MTP(X = t(pcbilDataTumorClusters[1:27]), Y = pcbilDataTumorClusters$cluster, test = "t.twosamp.unequalvar", robust = TRUE, seed = seeds[cont])

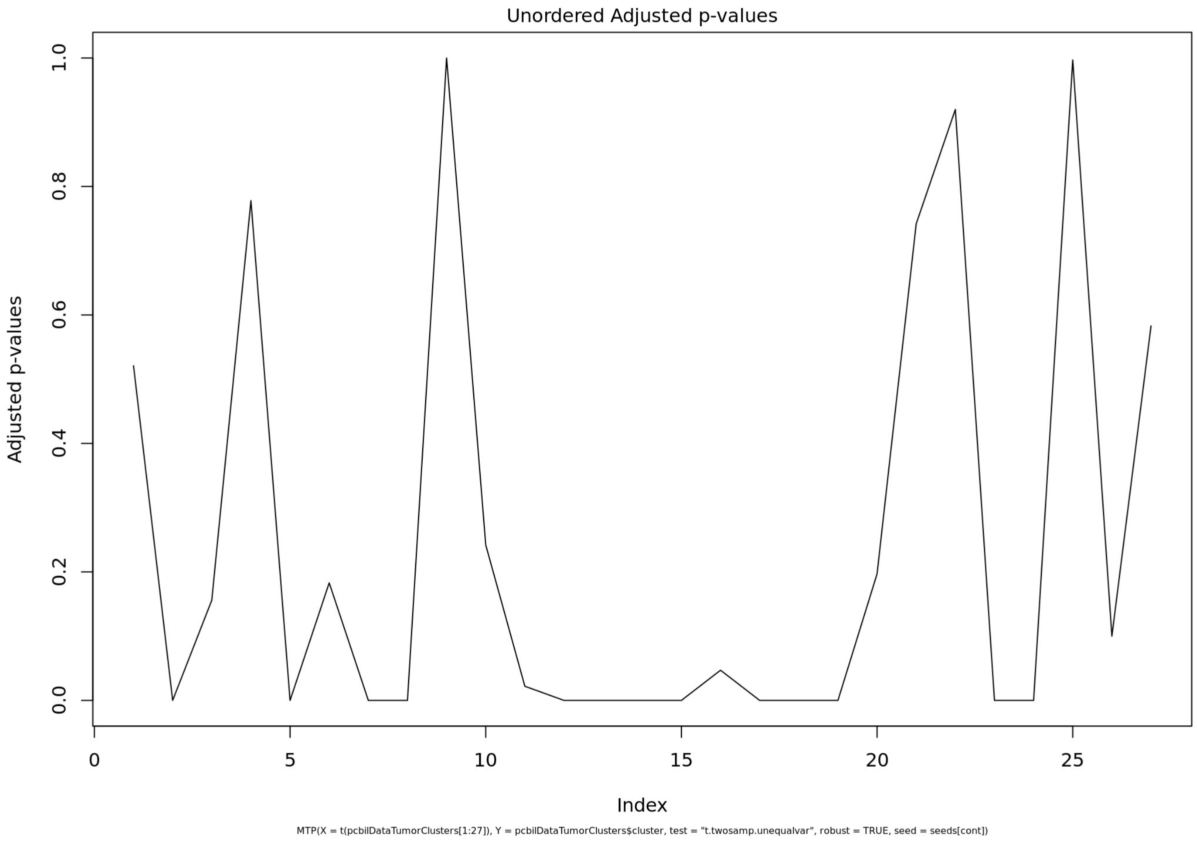
Unordered Adjusted p-values
Adjusted p-values
0.0
0.2
0.4
0.6
0.8
1.0
0
5
10
15
20
25
Index
MTP(X = t(pcbilDataTumorClusters[1:27]), Y = pcbilDataTumorClusters$cluster, test = "t.twosamp.unequalvar", robust = TRUE, seed = seeds[cont])

```
## NULL
##
##
## +----------+------------+------------+---------------+---------------+-
-------------+
## |  marker  |  raw-pval  |  adj-pval  |  mean class 1 |  mean class 2
|  mean diffs |
## +==========+============+============+===============+===============+=
=============+
## |   ck19   |          0 |          0 |          92.4 |         6.077
|       86.32 |
## +----------+------------+------------+---------------+---------------+-
-------------+
## |  berep4  |          0 |          0 |         75.94 |         1.119
|       74.82 |
## +----------+------------+------------+---------------+---------------+-
-------------+
## |    ck7   |          0 |          0 |         80.13 |         7.066
|       73.07 |
## +----------+------------+------------+---------------+---------------+-
-------------+
## |    ema   |          0 |          0 |         79.47 |         12.32
|       67.15 |
## +----------+------------+------------+---------------+---------------+-
-------------+
## |   muc1   |          0 |          0 |          67.8 |          5.84
|       61.95 |
## +----------+------------+------------+---------------+---------------+-
-------------+
## |  ca19.9  |          0 |          0 |         51.99 |        0.6473
|       51.34 |
## +----------+------------+------------+---------------+---------------+-
-------------+
## |    vim   |          0 |          0 |         42.93 |         4.222
|       38.71 |
## +----------+------------+------------+---------------+---------------+-
-------------+
## |   pcea   |          0 |          0 |         66.18 |         44.09
|       22.09 |
## +----------+------------+------------+---------------+---------------+-
-------------+
## |  maspin  |          0 |          0 |         22.18 |         3.223
|       18.96 |
## +----------+------------+------------+---------------+---------------+-
-------------+
## |  wt1cyt  |          0 |          0 |         15.67 |         1.015
|       14.66 |
## +----------+------------+------------+---------------+---------------+-
-------------+
## |   smad4  |          0 |          0 |         85.08 |         72.72
|       12.36 |
## +----------+------------+------------+---------------+---------------+-
```

```
-------------+
## |     ki67 |          0 |          0 |          31.35 |          21.57
|         9.78 |
## +----------+------------+------------+---------------+---------------+-
-------------+
## |     mcea |          0 |          0 |          3.349 |         0.4095
|         2.94 |
## +----------+------------+------------+---------------+---------------+-
-------------+
##
## Table: class 1: intrahepatic cholangiocarcinoma | class 2: hepatocellula
r carcinoma
```

Differential expresion analysis - RankProd

```
rankProdResults <- list()

cont <- 1
while(cont <= length(diffDiagn1)) {

  # Subset tumor clusters to be compared
  pcbilDataTumorClusters <- subset(pcbilDataClustered, pcbilDataClustered$cl
uster == diffDiagn1[cont] | pcbilDataClustered$cluster == diffDiagn2[cont])

  subsetClasses <- pcbilDataTumorClusters$cluster == diffDiagn1[cont]
  subsetClasses <- as.numeric(subsetClasses)

  aRP <- RP(t(pcbilDataTumorClusters[1:27]), subsetClasses, num.perm=100, lo
gged=FALSE, plot=FALSE, rand=seeds[cont])

  print(plotRP(aRP, cutoff= alpha))

  tg <- topGene(aRP,cutoff= alpha,method="pfp",logged=FALSE, gene.names=coln
ames(pcbilDataTumorClusters)[1:27])
  print(tg)
  print(paste("class 1: ", diffDiagn1[cont], " | class 2: ", diffDiagn2[con
t], sep=""))

  rankProdResults <- append(rankProdResults, list(c(rownames(tg[[1]]), rowna
mes(tg[[2]]))))

  cont <- cont + 1
}
```

```
## Rank Product analysis for two-class case
##
## Starting 100 permutations...
## Computing pfp ..
## Outputing the results ..
```

Identification of Up-regulated genes under class 2

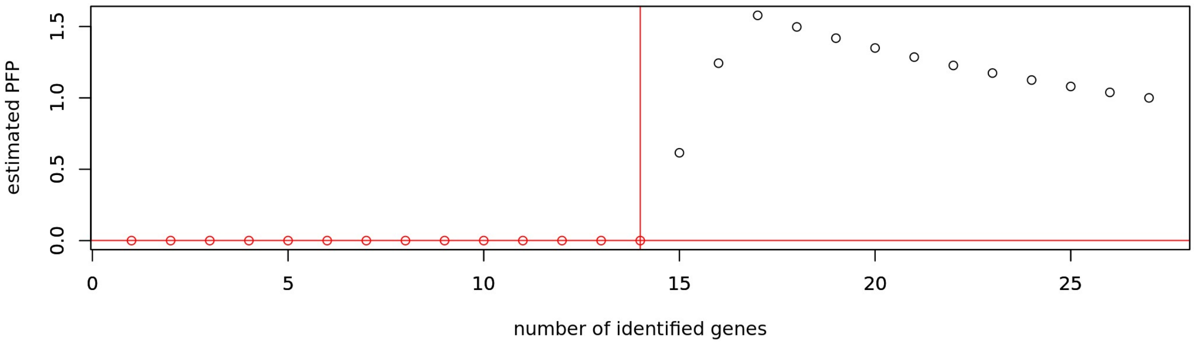

Identification of down-regulated genes under class 2

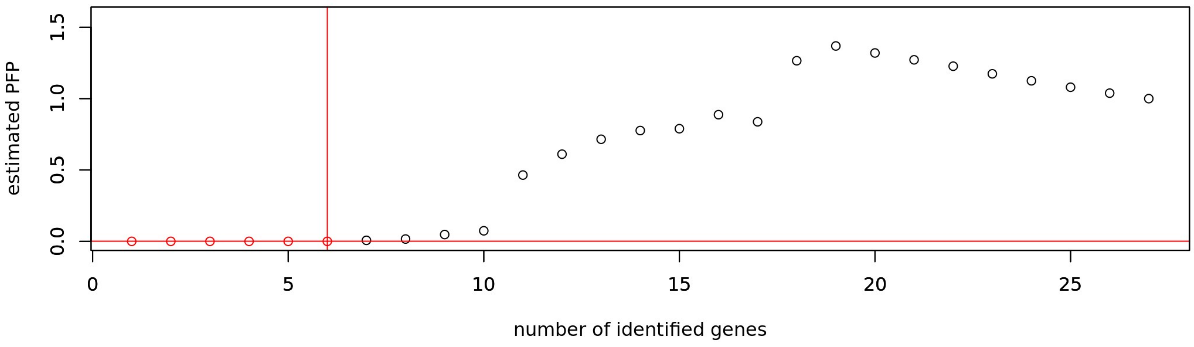

```
## NULL
## Table1: Genes called significant under class1 < class2
##
## Table2: Genes called significant under class1 > class2
##
## $Table1
##        gene.index RP/Rsum FC:(class1/class2) pfp P.value
## ca125          16  4.6318             0.0779   0       0
## muc5ac         10  5.0123             0.1426   0       0
## mcea           14  5.1853             0.0690   0       0
## muc2            9  5.7301             0.0601   0       0
## ck17            3  6.1118             0.1908   0       0
## chra           25  6.1808             0.2226   0       0
## cdx2           20  6.1881             0.3965   0       0
## maspin         18  6.4716             0.3004   0       0
## p63            22  6.6425             0.3675   0       0
## ck5             1  7.0921             0.2391   0       0
## cd10           27  7.1886             1.0219   0       0
## ck20            6  7.2860             0.4359   0       0
## p53            21  7.3868             0.4263   0       0
## muc6           11  7.6806             1.0934   0       0
##
## $Table2
##        gene.index RP/Rsum FC:(class1/class2) pfp P.value
## cd56           26  3.0509            16.0889   0       0
## vim             7  3.8711             2.6945   0       0
## wt1cyt         19  3.9181             4.1034   0       0
## smad4          24  4.0844             2.0266   0       0
## muc1            8  6.9345             1.1113   0       0
## ck18            4  7.8315             1.0137   0       0
##
## [1] "class 1: extrahepatic pancreatobiliary | class 2: intrahepatic chola
ngiocarcinoma"
## Rank Product analysis for two-class case
##
## Starting 100 permutations...
## Computing pfp ..
## Outputing the results ..
```

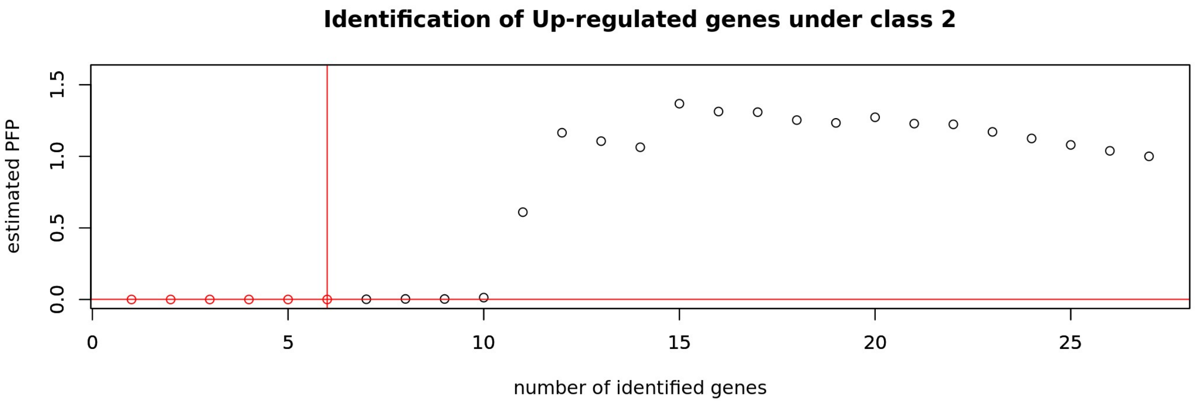
Identification of Up-regulated genes under class 2
estimated PFP
0.0
0.5
1.0
1.5
0
5
10
15
20
25
number of identified genes

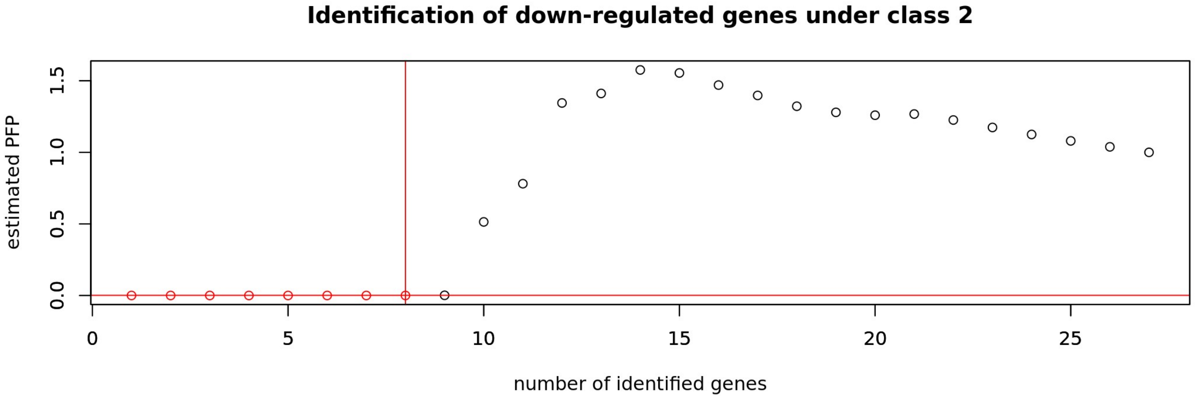
Identification of down-regulated genes under class 2
estimated PFP
0.0
0.5
1.0
1.5
0
5
10
15
20
25
number of identified genes

```
## NULL
## Table1: Genes called significant under class1 < class2
##
## Table2: Genes called significant under class1 > class2
##
## $Table1
##        gene.index RP/Rsum FC:(class1/class2) pfp P.value
## vim             7  4.5681             0.0392   0       0
## ck17            3  4.5926             0.2412   0       0
## muc1            8  5.3751             0.4633   0       0
## ca125          16  5.8329             0.4464   0       0
## muc5ac         10  6.1011             0.4439   0       0
## ck7             2  6.4176             0.5234   0       0
##
## $Table2
##        gene.index RP/Rsum FC:(class1/class2) pfp P.value
## ck20            6  3.9158             8.1943   0       0
## cd56           26  3.9433             6.0285   0       0
## muc2            9  4.2625            15.8726   0       0
## cdx2           20  5.0283             4.6856   0       0
## chra           25  5.1662             2.4745   0       0
## wt1cyt         19  6.8115             1.3162   0       0
## cd10           27  7.0140             2.7287   0       0
## smad4          24  7.1322             1.6647   0       0
##
## [1] "class 1: extrahepatic pancreatobiliary | class 2: intestinal"
## Rank Product analysis for two-class case
##
## Starting 100 permutations...
## Computing pfp ..
## Outputing the results ..
```

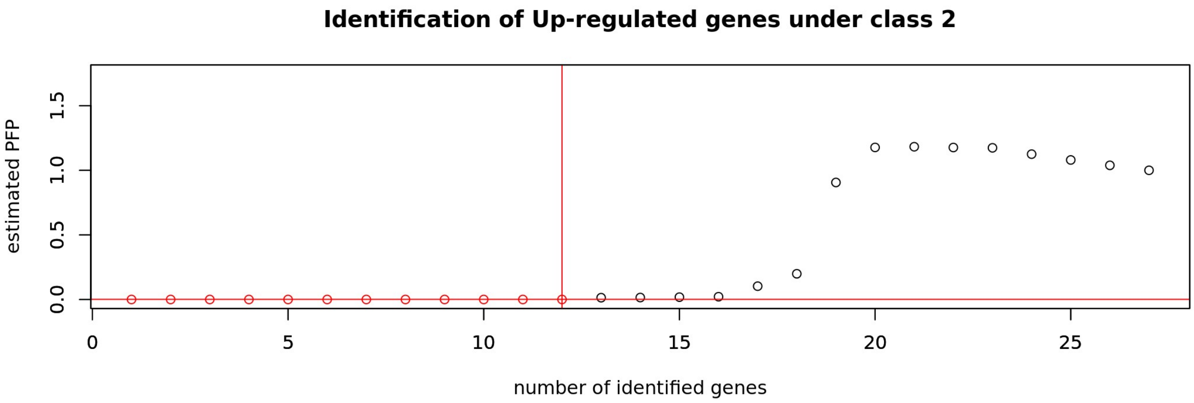

Identification of Up-regulated genes under class 2
estimated PFP
number of identified genes

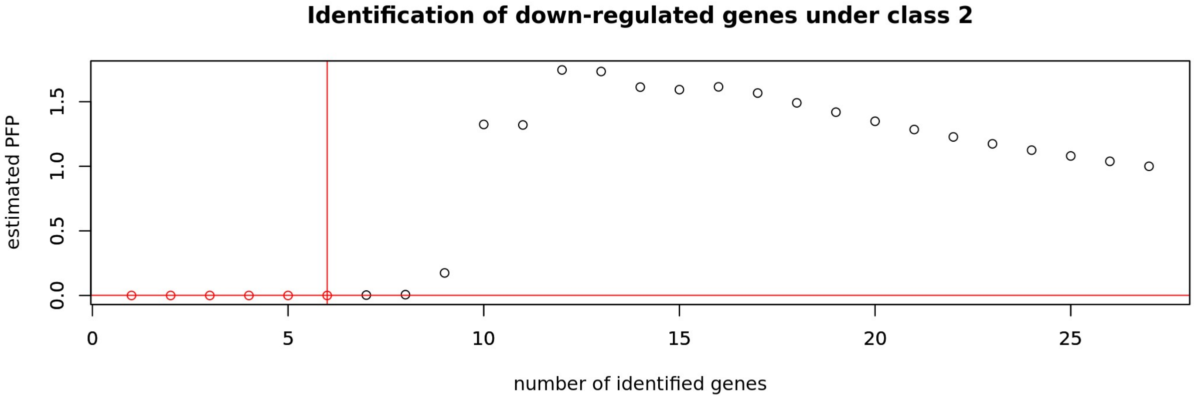

Identification of down-regulated genes under class 2
estimated PFP
number of identified genes

```
## NULL
## Table1: Genes called significant under class1 < class2
##
## Table2: Genes called significant under class1 > class2
##
## $Table1
##        gene.index RP/Rsum FC:(class1/class2) pfp P.value
## chra           25  5.1464             0.0000   0       0
## berep4         12  5.1851             0.0147   0       0
## ca19.9         17  5.3694             0.0125   0       0
## muc1            8  5.6560             0.0861   0       0
## ck19            5  5.6654             0.0658   0       0
## ca125          16  5.7073             0.0160   0       0
## vim             7  6.0833             0.0983   0       0
## mcea           14  6.2989             0.1223   0       0
## wt1cyt         19  6.3333             0.0648   0       0
## cdx2           20  6.8809             0.1338   0       0
## maspin         18  6.9948             0.1453   0       0
## ck7             2  7.0633             0.0882   0       0
##
## $Table2
##       gene.index RP/Rsum FC:(class1/class2) pfp P.value
## ck20           6  2.9247             2.1025   0       0
## ck18           4  3.4015             0.9556   0       0
## cd10          27  3.4277             1.9647   0       0
## smad4         24  3.9683             0.8547   0       0
## ki67          23  4.5387             0.6879   0       0
## pcea          15  5.2410             0.6662   0       0
##
## [1] "class 1: intrahepatic cholangiocarcinoma | class 2: hepatocellular c
arcinoma"
```

Differential expresion analysis - SAM

```r
samResults <- list()

cont <- 1
while(cont <= length(diffDiagn1)) {

  # Subset tumor clusters to be compared
  pcbilDataTumorClusters <- subset(pcbilDataClustered, pcbilDataClustered$cl
uster == diffDiagn1[cont] | pcbilDataClustered$cluster == diffDiagn2[cont])

  subsetClasses <- pcbilDataTumorClusters$cluster == diffDiagn1[cont]
  subsetClasses <- as.numeric(subsetClasses)
  subsetClasses <- subsetClasses + 1

  aSAM <- SAM(t(pcbilDataTumorClusters[1:27]), subsetClasses, resp.type="Tw
o class unpaired", testStatistic = "wilcoxon", regression.method= "ranks" ,n
perms = 1000, genenames = colnames(pcbilDataTumorClusters)[1:27], fdr.output
= alpha)

  print(plot(aSAM))
  print(aSAM)
  print(paste("class 1: ", diffDiagn1[cont], " | class 2: ", diffDiagn2[con
t], sep=""))

  samResults <- append(samResults, list(c(aSAM$siggenes.table$genes.up[,1],
aSAM$siggenes.table$genes.lo[,1])) )

  cont <- cont + 1
}
```

```
## Warning in factorial(length(y)): value out of range in 'gammafn'
```

```
## perm= 1 ... 1000
##
## Computing delta table
## 1 ...  50
```

```
## NULL
## Call:
## SAM(x = t(pcbilDataTumorClusters[1:27]), y = subsetClasses, resp.type =
"Two class unpaired",
##     genenames = colnames(pcbilDataTumorClusters)[1:27], nperms = 1000,
##     testStatistic = "wilcoxon", regression.method = "ranks",
##     fdr.output = alpha)
##
## Genes up
##       Gene ID Gene Name Score(d) Numerator(r) Denominator(s+s0)
##  [1,] mcea    14        5.369    6649.5       1238.504
##  [2,] muc5ac  10        5.124    6346.5       1238.504
##  [3,] ca125   16        5.058    6264.5       1238.504
##  [4,] maspin  18        4.719    5844.5       1238.504
##  [5,] ck17    3         4.193    5192.5       1238.504
##  [6,] pcea    15        2.633    3261.5       1238.504
##  [7,] ca19.9  17        2.252    2789.5       1238.504
##  [8,] cdx2    20        2.176    2695         1238.504
##  [9,] ema     13        2.174    2693         1238.504
## [10,] p53     21        1.989    2463         1238.504
## [11,] ck5     1         1.449    1795         1238.504
## [12,] cd10    27        1.358    1682.5       1238.504
## [13,] muc6    11        1.328    1645         1238.504
## [14,] muc2    9         1.241    1537.5       1238.504
##       Fold Change q-value(%)
##  [1,] 14.494      0
##  [2,] 7.011       0
##  [3,] 12.83       0
##  [4,] 3.329       0
##  [5,] 5.24        0
##  [6,] 1.325       0
##  [7,] 1.453       0
##  [8,] 2.522       0
##  [9,] 1.147       0
## [10,] 2.346       0
## [11,] 4.182       0
## [12,] 0.979       0
## [13,] 0.915       0
## [14,] 16.626      0
##
## Genes down
##      Gene ID Gene Name Score(d) Numerator(r) Denominator(s+s0) Fold Chang
e
## [1,] smad4   24        -4.04    -5003        1238.504          0.49
3
## [2,] vim     7         -2.941   -3642        1238.504          0.37
1
## [3,] wt1cyt  19        -2.261   -2800.5      1238.504          0.24
4
## [4,] cd56    26        -1.941   -2404        1238.504          0.06
2
## [5,] muc1    8         -0.884   -1094.5      1238.504          0.
```

```
9
## [6,] ck18    4         -0.542   -671         1238.504          0.98
7
##      q-value(%)
## [1,] 0
## [2,] 0
## [3,] 0
## [4,] 0
## [5,] 0
## [6,] 0.741
## [1] "class 1: extrahepatic pancreatobiliary | class 2: intrahepatic chola
ngiocarcinoma"
```

```
## Warning in factorial(length(y)): value out of range in 'gammafn'
```

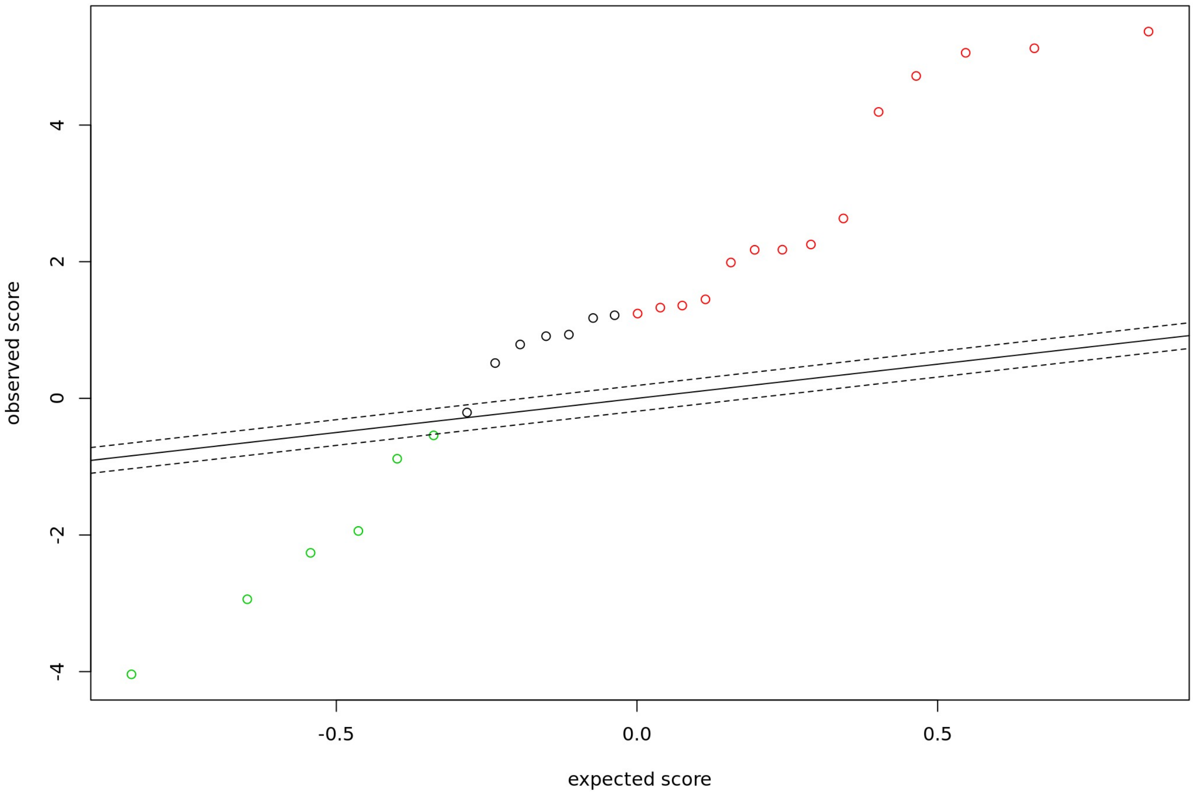


```
## perm= 1 ...1000
##
## Computing delta table
## 1 ... 50
```

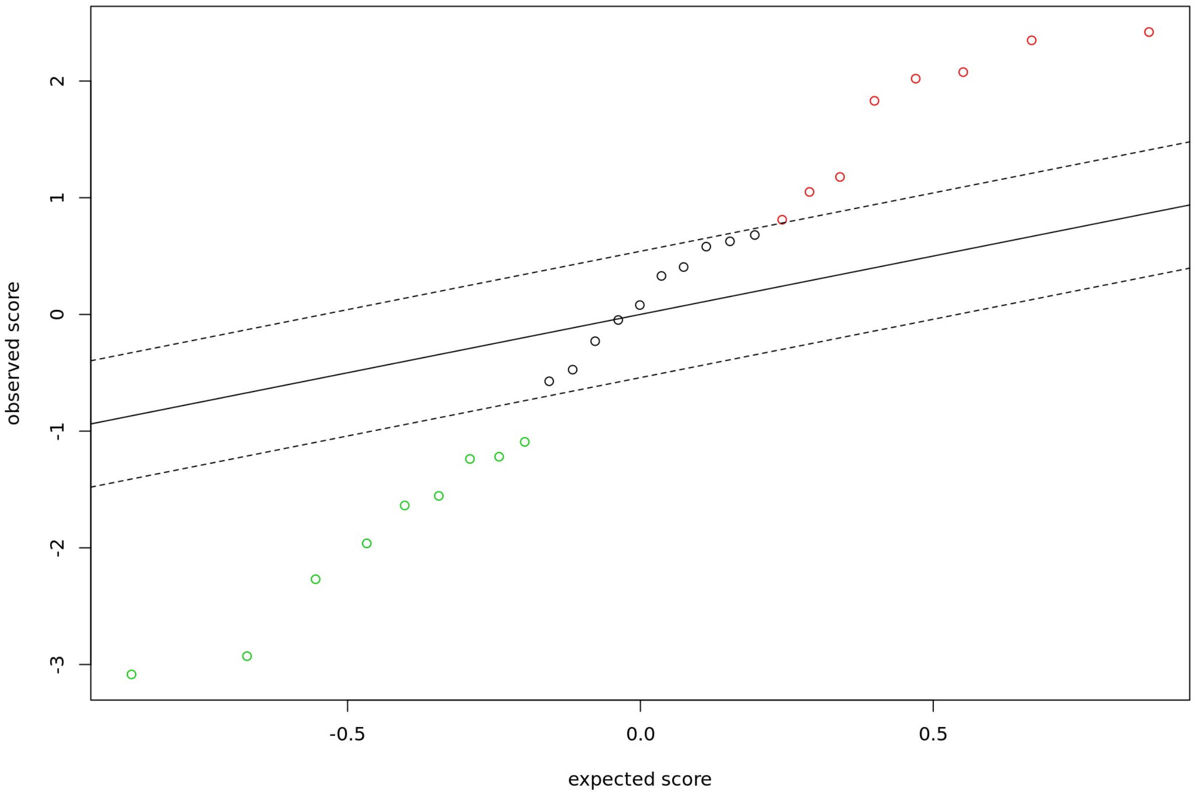

observed score
expected score
2
1
0
-1
-2
-3
-0.5
0.0
0.5

```
## NULL
## Call:
## SAM(x = t(pcbilDataTumorClusters[1:27]), y = subsetClasses, resp.type =
"Two class unpaired",
##     genenames = colnames(pcbilDataTumorClusters)[1:27], nperms = 1000,
##     testStatistic = "wilcoxon", regression.method = "ranks",
##     fdr.output = alpha)
##
## Genes up
##      Gene ID Gene Name Score(d) Numerator(r) Denominator(s+s0) Fold Chang
e
## [1,] ck17    3         2.42     1445         597.167           4.14
6
## [2,] ck7     2         2.349    1402.5       597.167           1.91
1
## [3,] muc1    8         2.076    1240         597.167           2.15
9
## [4,] muc5ac  10        2.02     1206.5       597.167           2.25
3
## [5,] ca125   16        1.83     1093         597.167           2.2
4
## [6,] ema     13        1.178    703.5        597.167           1.07
8
## [7,] vim     7         1.05     627          597.167           25.4
9
## [8,] muc6    11        0.811    484.5        597.167           1.58
4
##      q-value(%)
## [1,] 0
## [2,] 0
## [3,] 0
## [4,] 0
## [5,] 0
## [6,] 0
## [7,] 0
## [8,] 1.307
##
## Genes down
##       Gene ID Gene Name Score(d) Numerator(r) Denominator(s+s0)
##  [1,] cdx2    20        -3.085   -1842        597.167
##  [2,] ck20    6         -2.928   -1748.5      597.167
##  [3,] muc2    9         -2.269   -1355        597.167
##  [4,] mcea    14        -1.962   -1171.5      597.167
##  [5,] cd56    26        -1.637   -977.5       597.167
##  [6,] smad4   24        -1.555   -928.5       597.167
##  [7,] ki67    23        -1.238   -739.5       597.167
##  [8,] chra    25        -1.219   -728         597.167
##  [9,] cd10    27        -1.092   -652         597.167
##       Fold Change q-value(%)
##  [1,] 0.213       0
##  [2,] 0.122       0
##  [3,] 0.063       0
```

```
##   [4,] 0.64        0
##   [5,] 0.166       0
##   [6,] 0.601       0
##   [7,] 0.756       0
##   [8,] 0.404       0
##   [9,] 0.366       0
## [1] "class 1: extrahepatic pancreatobiliary | class 2: intestinal"
## perm= 1 ... 1000
##
## Computing delta table
## 1 ... 50
```

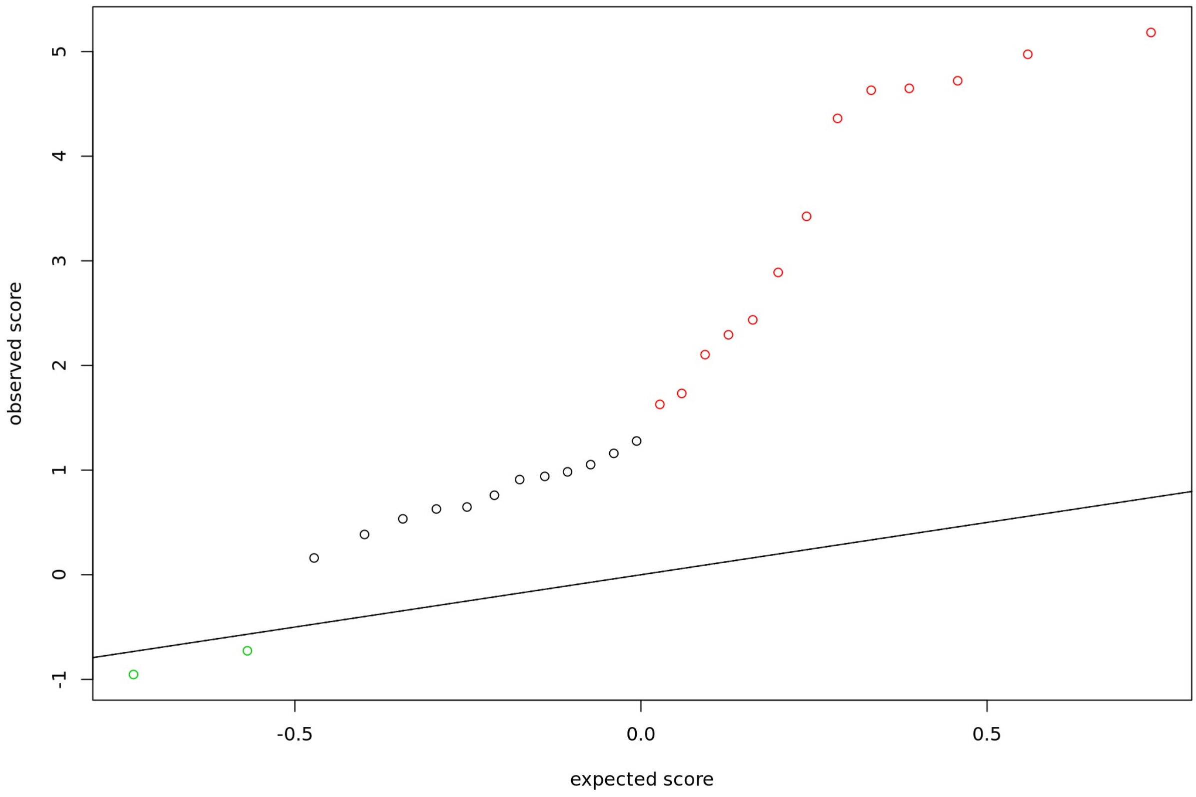

```
## NULL
## Call:
## SAM(x = t(pcbilDataTumorClusters[1:27]), y = subsetClasses, resp.type =
"Two class unpaired",
##     genenames = colnames(pcbilDataTumorClusters)[1:27], nperms = 1000,
##     testStatistic = "wilcoxon", regression.method = "ranks",
##     fdr.output = alpha)
##
## Genes up
##       Gene ID Gene Name Score(d) Numerator(r) Denominator(s+s0)
##  [1,] ck19    5         5.182    2745.5       529.772
##  [2,] berep4  12        4.974    2635         529.772
##  [3,] ema     13        4.721    2501         529.772
##  [4,] muc1    8         4.648    2462.5       529.772
##  [5,] ck7     2         4.63     2453         529.772
##  [6,] ca19.9  17        4.361    2310.5       529.772
##  [7,] vim     7         3.425    1814.5       529.772
##  [8,] smad4   24        2.889    1530.5       529.772
##  [9,] wt1cyt  19        2.436    1290.5       529.772
## [10,] ki67    23        2.293    1215         529.772
## [11,] maspin  18        2.104    1114.5       529.772
## [12,] pcea    15        1.733    918          529.772
## [13,] mcea    14        1.628    862.5        529.772
##       Fold Change q-value(%)
##  [1,] 15.205      0
##  [2,] 67.847      0
##  [3,] 6.452       0
##  [4,] 11.608      0
##  [5,] 11.34       0
##  [6,] 80.313      0
##  [7,] 10.168      0
##  [8,] 1.17        0
##  [9,] 15.439      0
## [10,] 1.454       0
## [11,] 6.881       0
## [12,] 1.501       0
## [13,] 8.178       0
##
## Genes down
##      Gene ID Gene Name Score(d) Numerator(r) Denominator(s+s0) Fold Chang
e
## [1,] ck20    6         -0.953   -505         529.772           0.47
6
## [2,] cd10    27        -0.727   -385         529.772           0.50
9
##      q-value(%)
## [1,] 0
## [2,] 0
## [1] "class 1: intrahepatic cholangiocarcinoma | class 2: hepatocellular c
arcinoma"
```

Differential expresion analysis - extract markers that where consistently significant by all three tests (multtest, RankProd, and SAM)

```
cont <- 1
while(cont <= length(diffDiagn1)) {

  temp <- intersect(multtestResults[[cont]], rankProdResults[[cont]])
  final <- intersect(temp, samResults[[cont]])

  print(paste(diffDiagn1[cont], " vs ", diffDiagn2[cont], ":", sep=""))
  print(final)
  print("-------------------------------------------------------")
  print("")

  cont <- cont + 1
}
```

```
## [1] "extrahepatic pancreatobiliary vs intrahepatic cholangiocarcinoma:"
##  [1] "maspin" "ca125"  "mcea"   "muc5ac" "ck17"   "p53"    "cdx2"  
##  [8] "cd56"   "wt1cyt" "vim"    "smad4" 
## [1] "-------------------------------------------------------"
## [1] ""
## [1] "extrahepatic pancreatobiliary vs intestinal:"
## [1] "ck7"    "muc1"   "muc5ac" "ck17"   "muc2"   "ck20"   "cdx2"  
## [1] "-------------------------------------------------------"
## [1] ""
## [1] "intrahepatic cholangiocarcinoma vs hepatocellular carcinoma:"
##  [1] "ck19"   "berep4" "ck7"    "muc1"   "ca19.9" "vim"    "pcea"  
##  [8] "maspin" "wt1cyt" "smad4"  "ki67"   "mcea"  
## [1] "-------------------------------------------------------"
## [1] ""
```

– End of differential expression analysis –
