# Supplementary material for: Immunohistochemical Typing of Adenocarcinomas of the Pancreatobiliary System Improves Diagnosis and Prognostic Stratification
Source: PLoS One. 2016 Nov 9;11(11):e0166067. doi: 10.1371/journal.pone.0166067 (PMC5102456; doi:10.1371/journal.pone.0166067)
Supplement: S2 Table — (PDF) [file pone.0166067.s006.pdf]

**Supplementary Table 2:** Relationship between the immunohistochemical tumor types and anatomically-based diagnoses (n = 409), according to the results of hierarchical cluster analysis.

| Immunohistochemical types | Anatomical diagnoses | | | | | | |
|---|---|---|---|---|---|---|---|
| | Ampullary carcinoma (n = 24) | Ductal pancreatic adenocarcinoma (n = 139) | Distal bile duct cancer (n = 7) | Gallbladder cancer (n = 37) | Perihilar cholangiocarcinoma (n = 27) | Intrahepatic cholangiocarcinoma (n = 97) | Hepatocellular carcinoma (n = 78) |
| Extrahepatic pancreatobiliary (n = 199) | 14 | 116 | 5 | 23 | 14 | 27 | 0 |
| Intestinal (n = 24) | 7 | 8 | 1 | 4 | 1 | 3 | 0 |
| Intrahepatic cholangiocarcinoma (n = 82) | 1 | 9 | 1 | 3 | 9 | 56 | 3 |
| Hepatocellular carcinoma (n = 68) | 0 | 0 | 0 | 1 | 0 | 0 | 67 |
| Unclassified (n = 36) | 2 | 6 | 0 | 6 | 3 | 11 | 8 |
