# Supplementary material for: Immunohistochemical Typing of Adenocarcinomas of the Pancreatobiliary System Improves Diagnosis and Prognostic Stratification
Source: PLoS One. 2016 Nov 9;11(11):e0166067. doi: 10.1371/journal.pone.0166067 (PMC5102456; doi:10.1371/journal.pone.0166067)

Supplementary Figure 3: Complex network-based immunoprofiles

immunohistochemical type

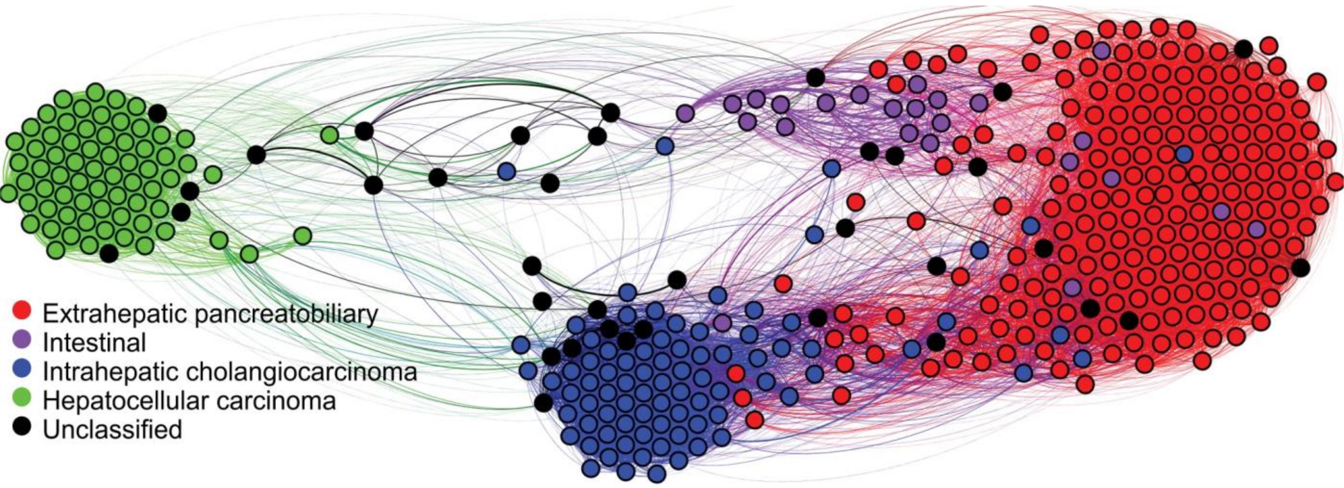

anatomical diagnosis

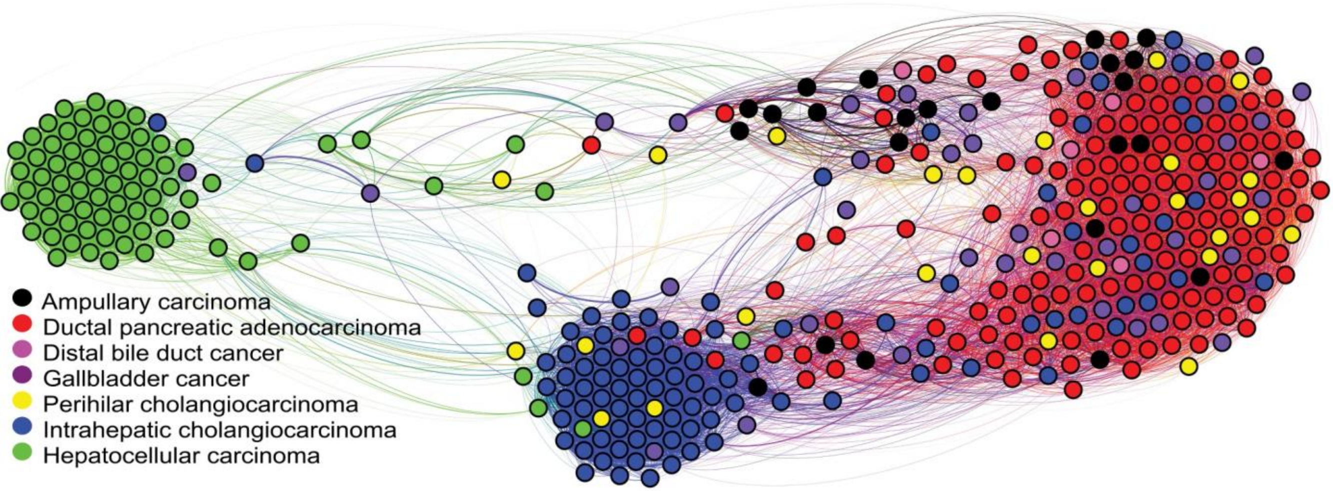

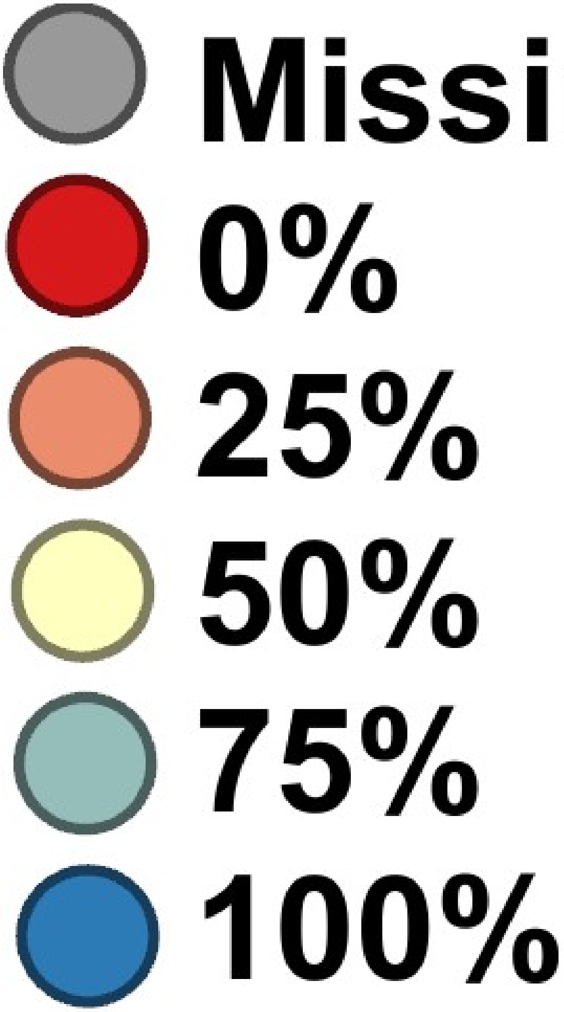
Missing Value
0%
25%
50%
75%
100%

ck5

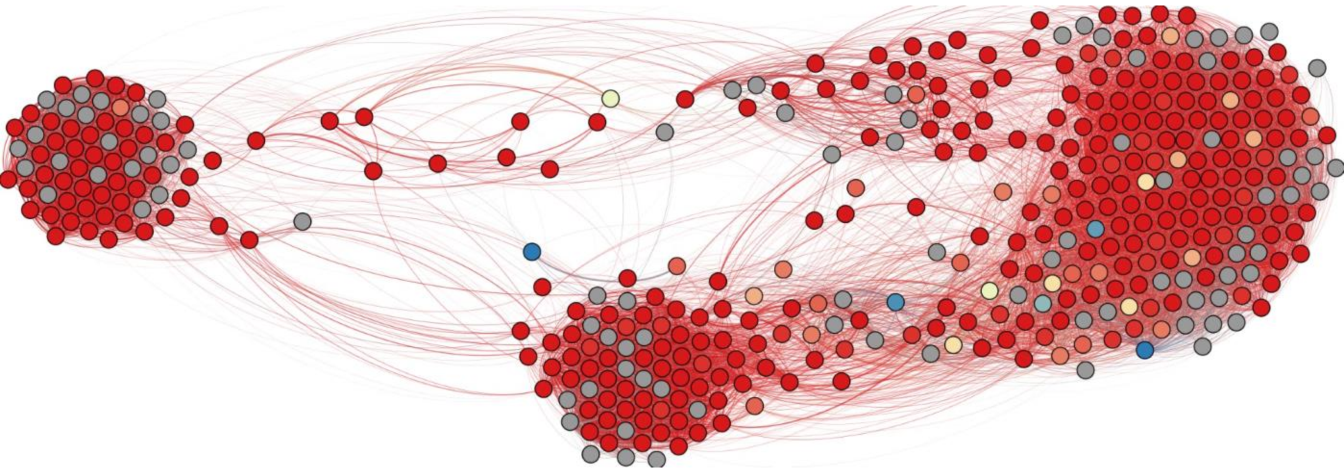

ck7

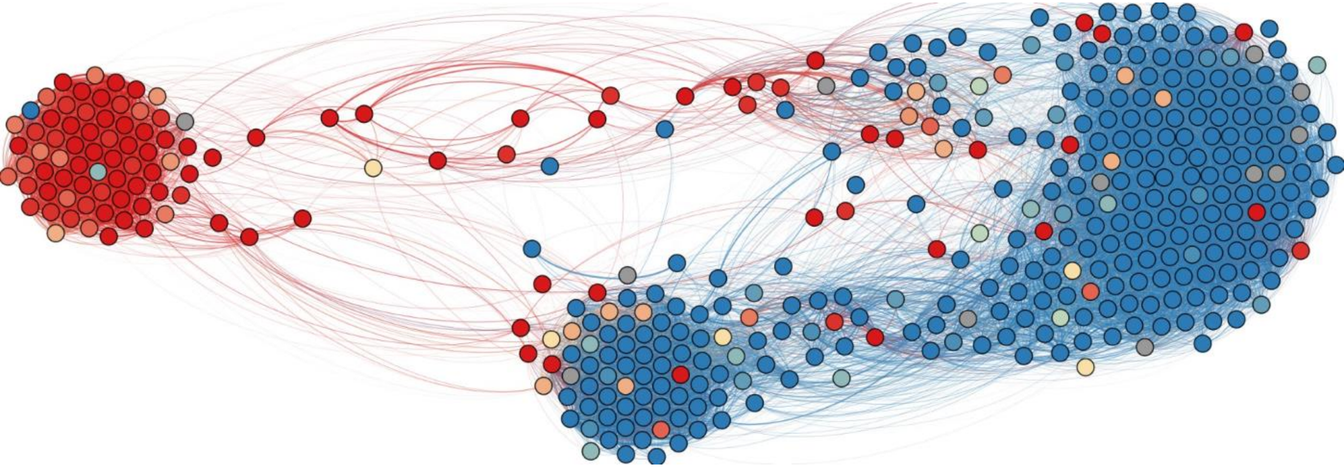

ck17

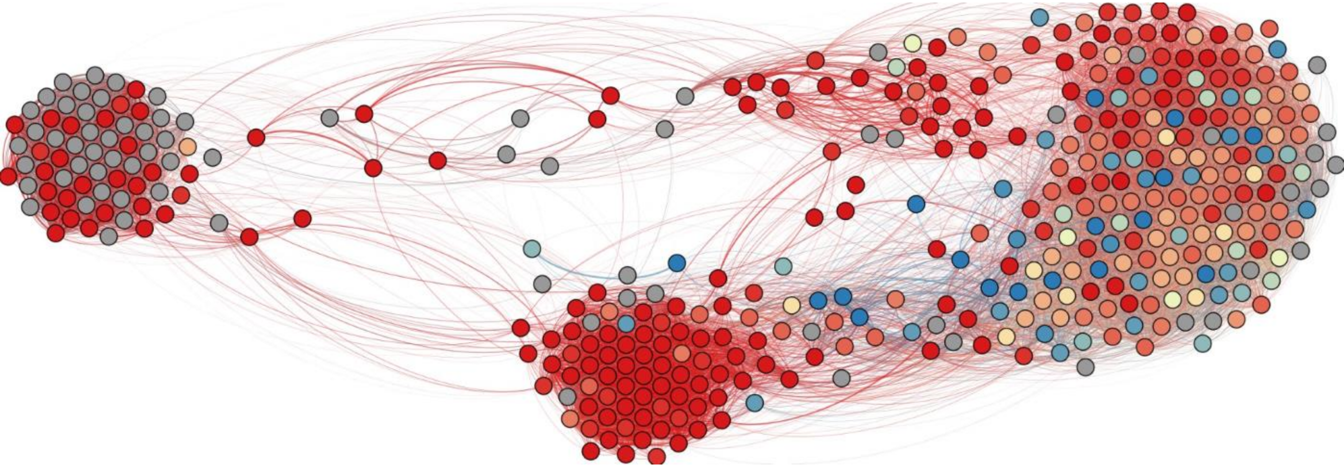

ck18

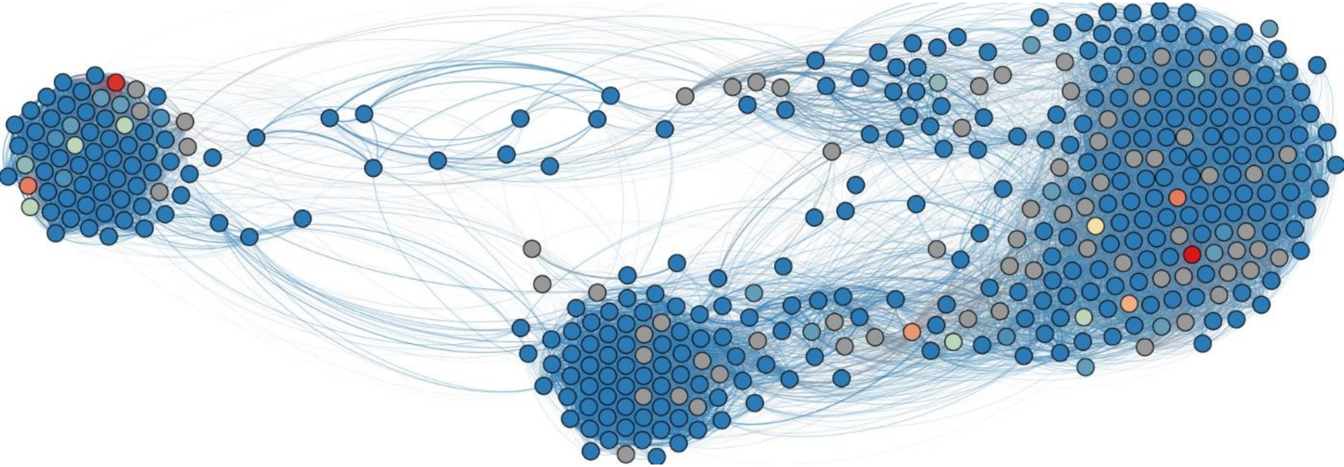

ck19

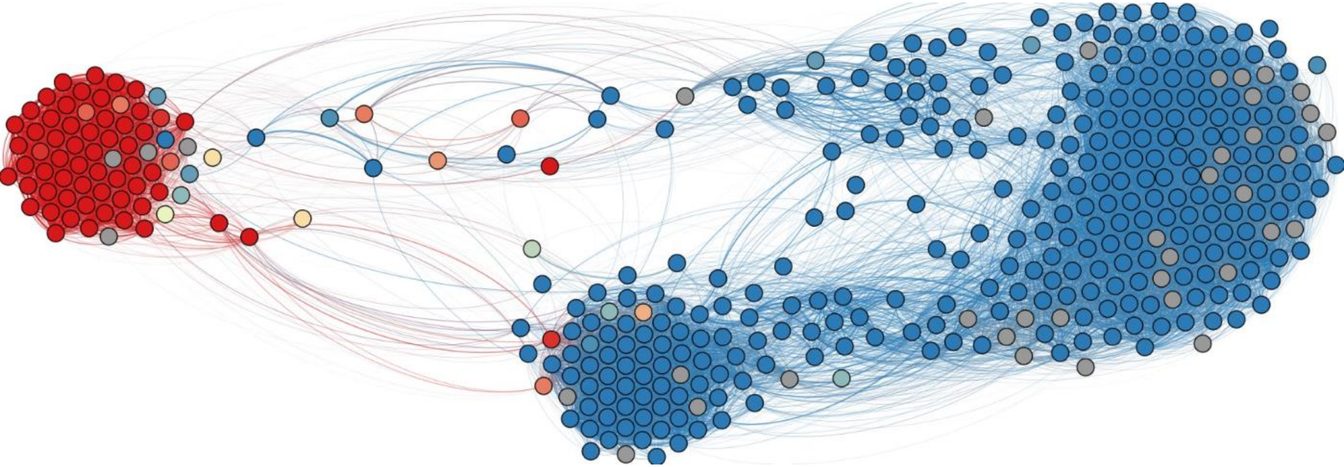

ck20

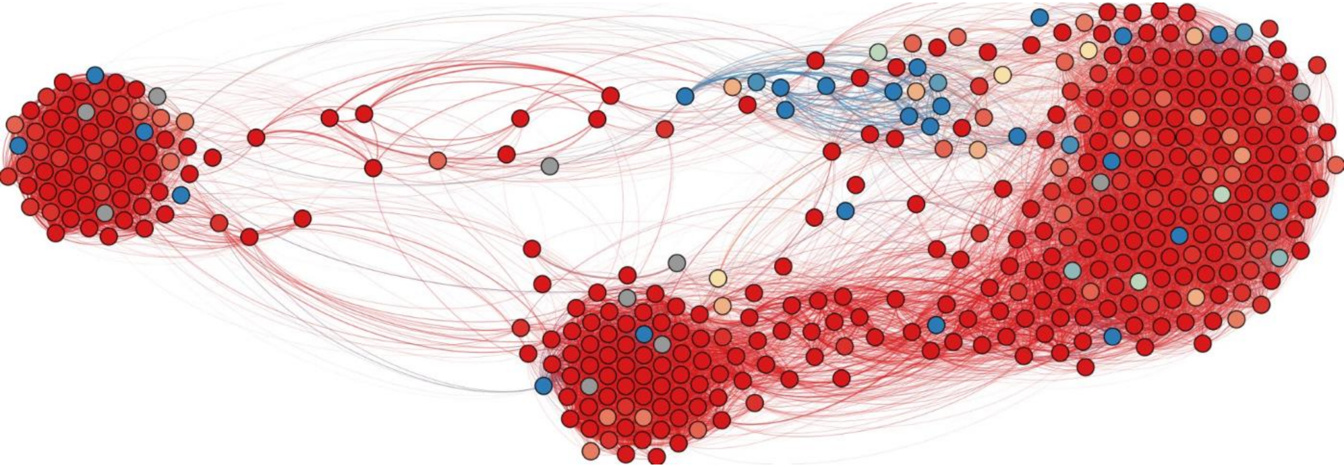

vim

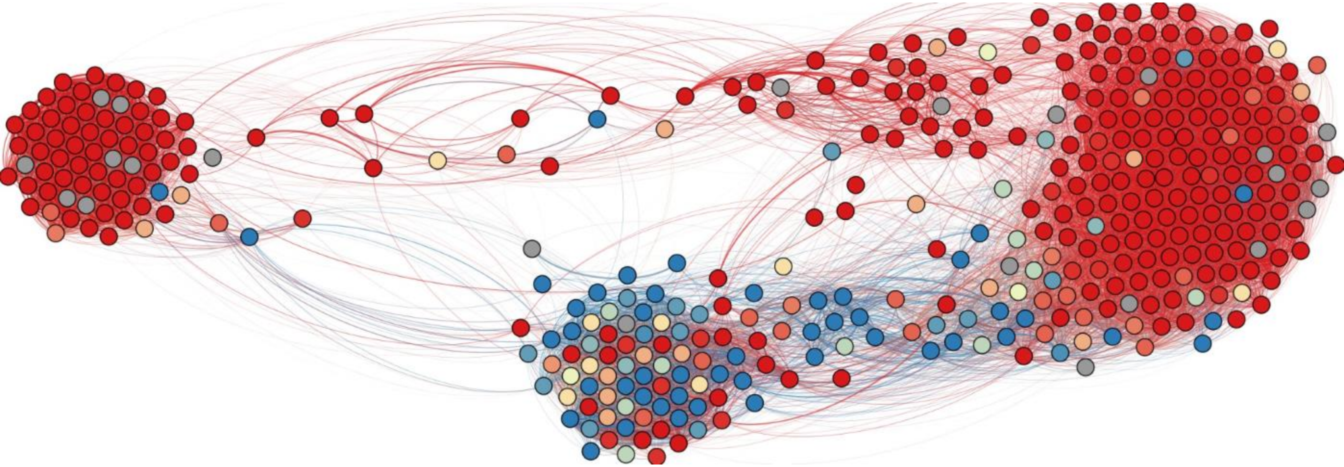

muc1

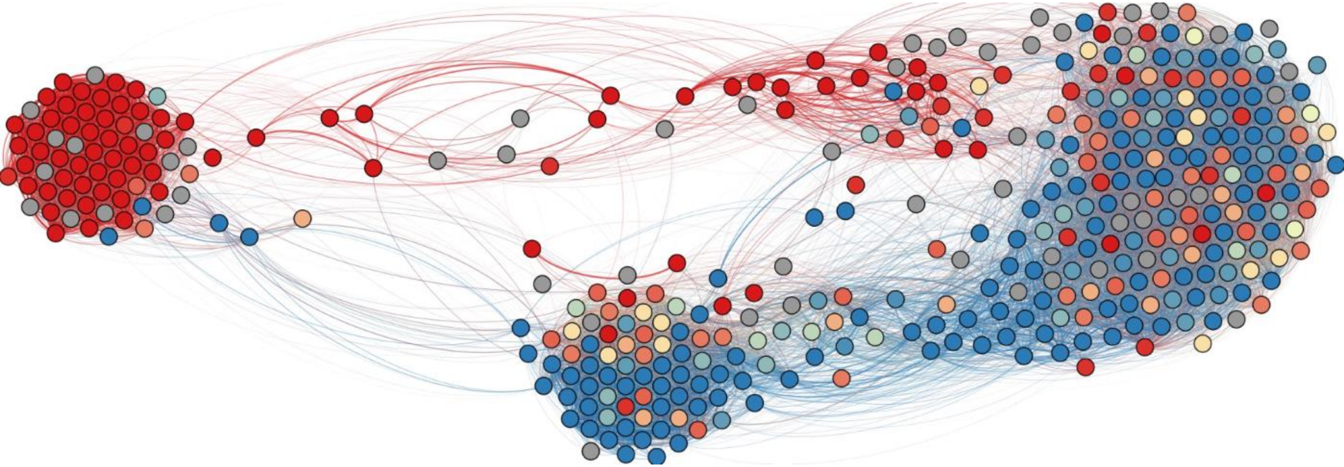

muc2

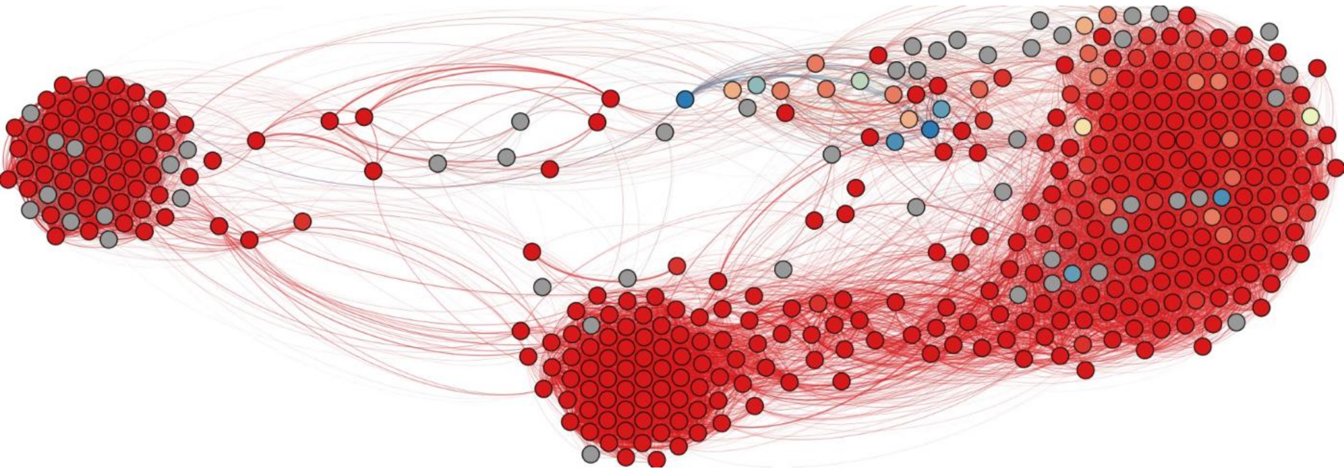

muc5ac

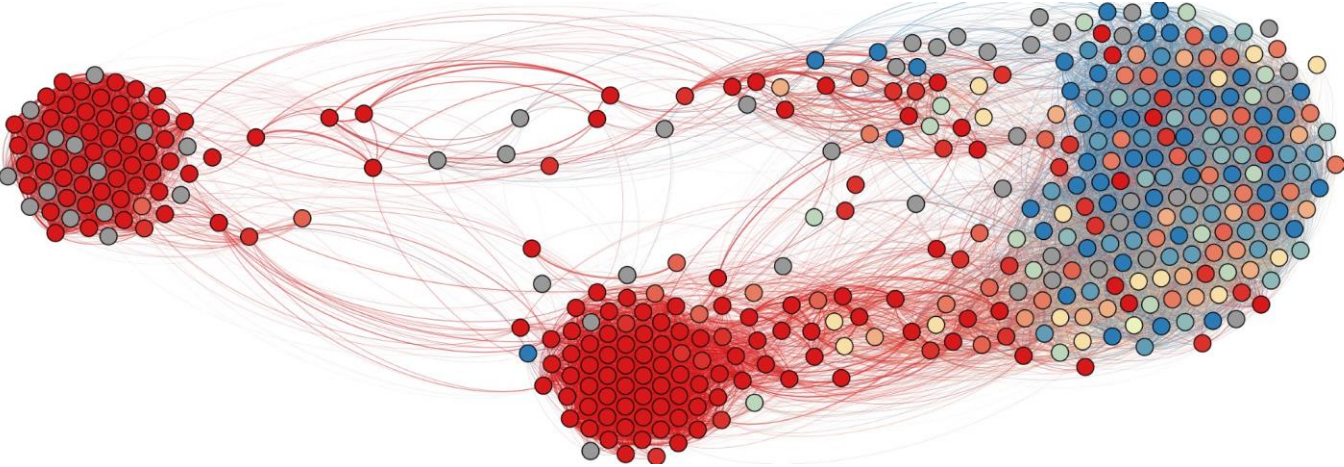

muc6

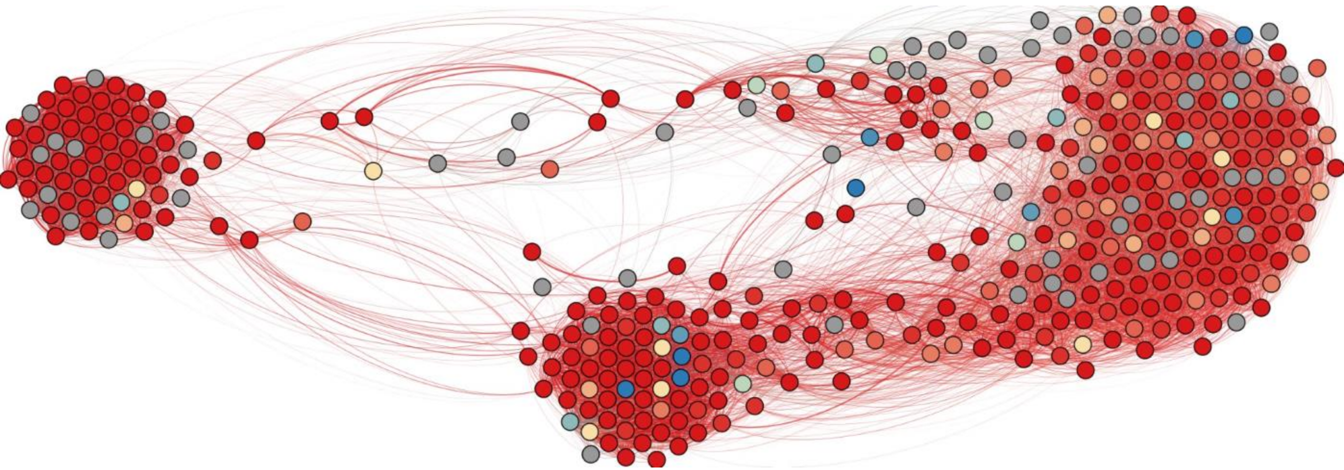

berep4

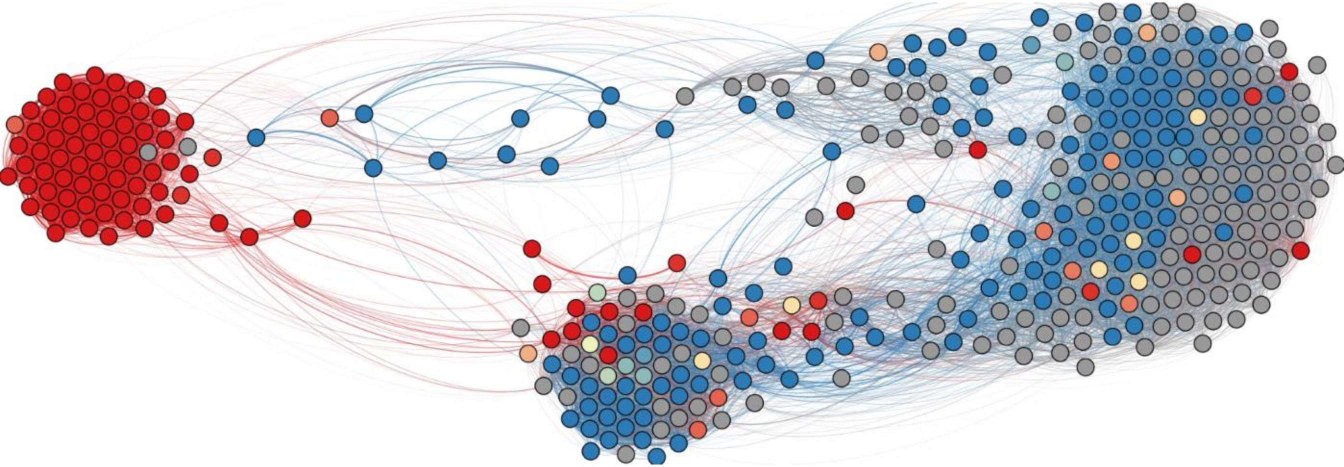

ema

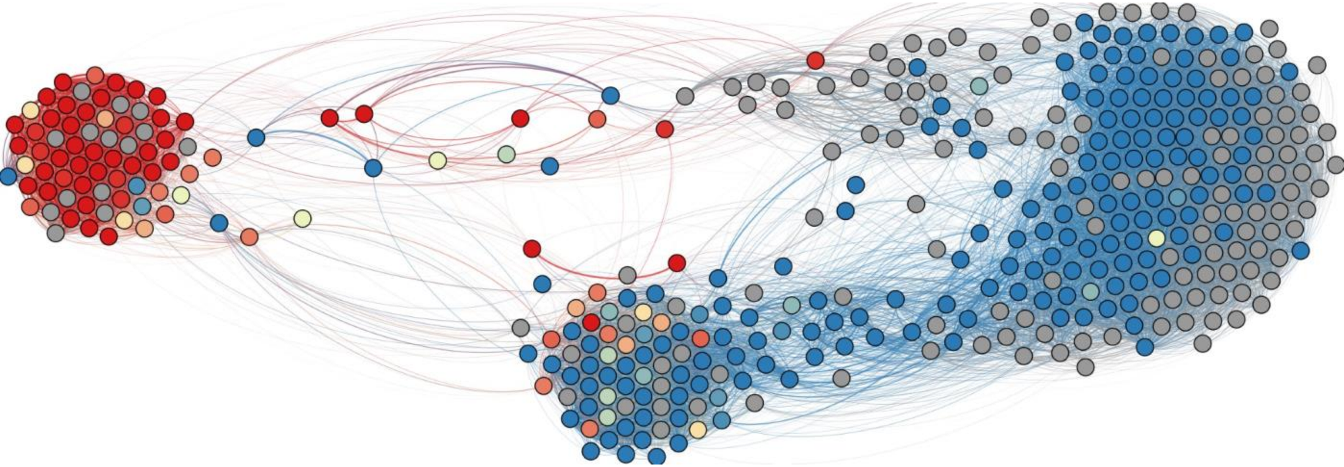

cea_m

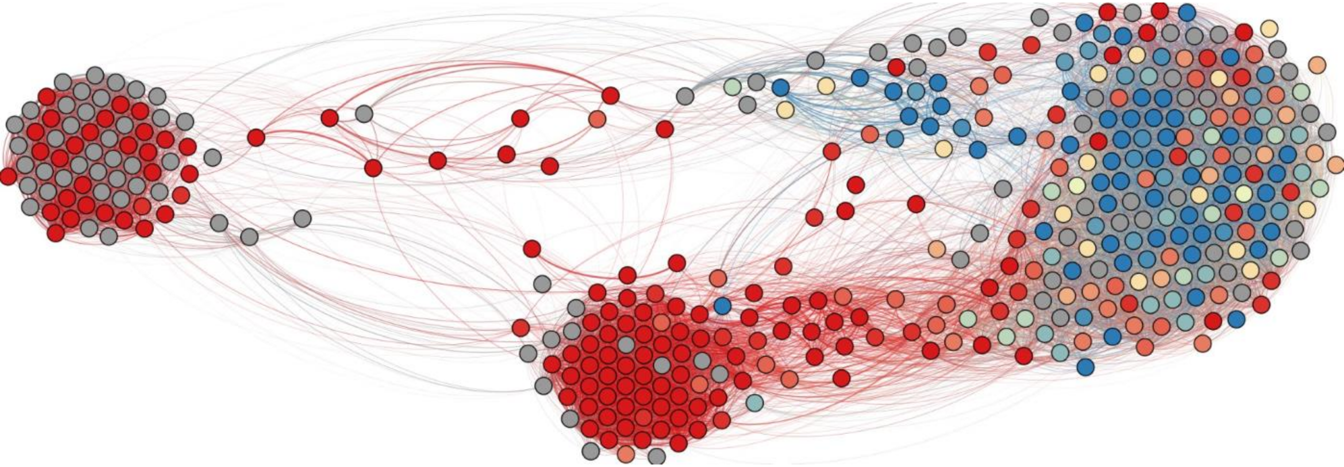

cea_p

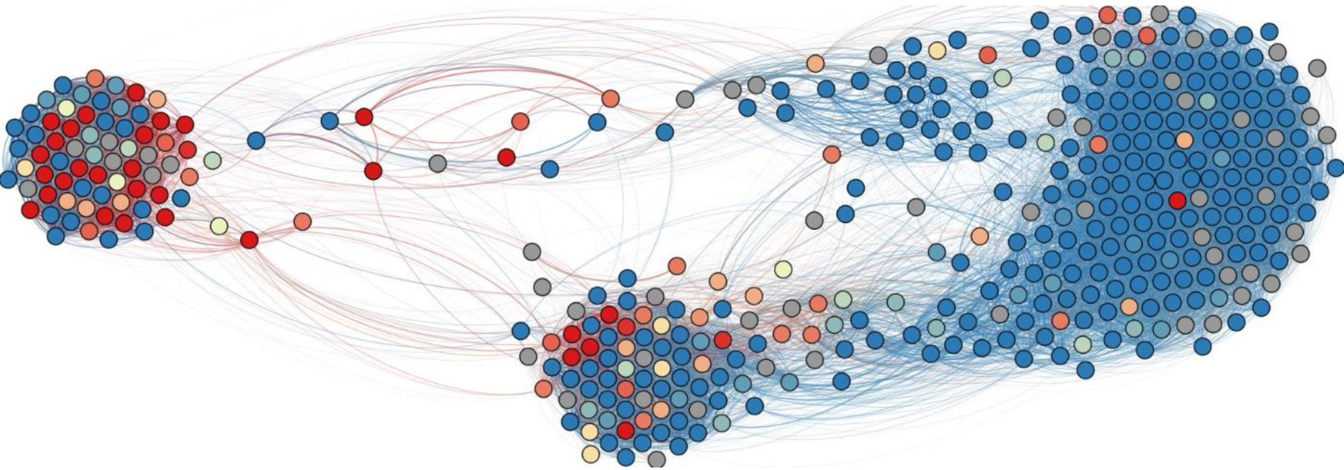

ca125

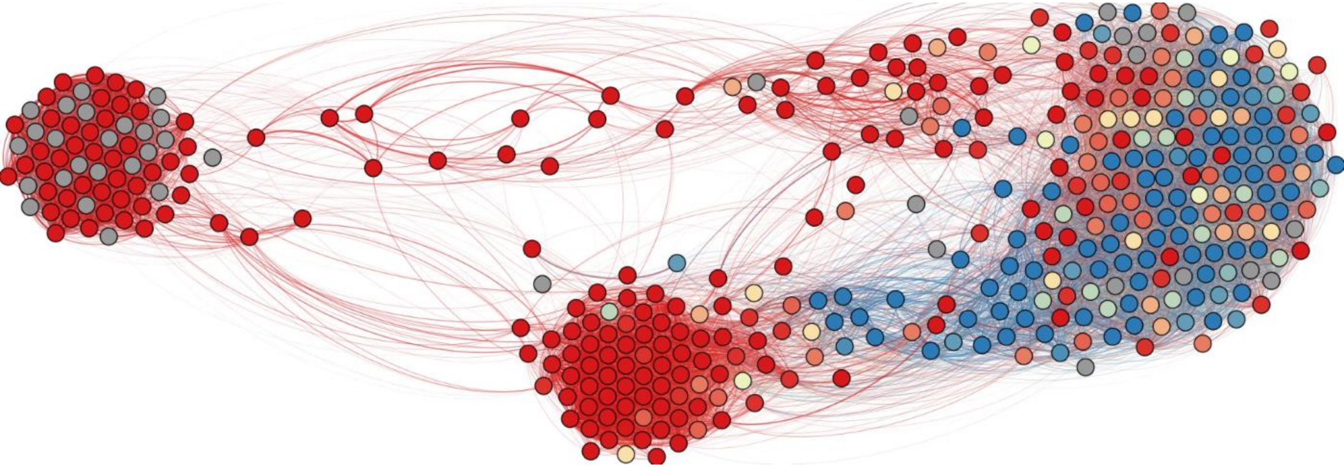

ca19_9

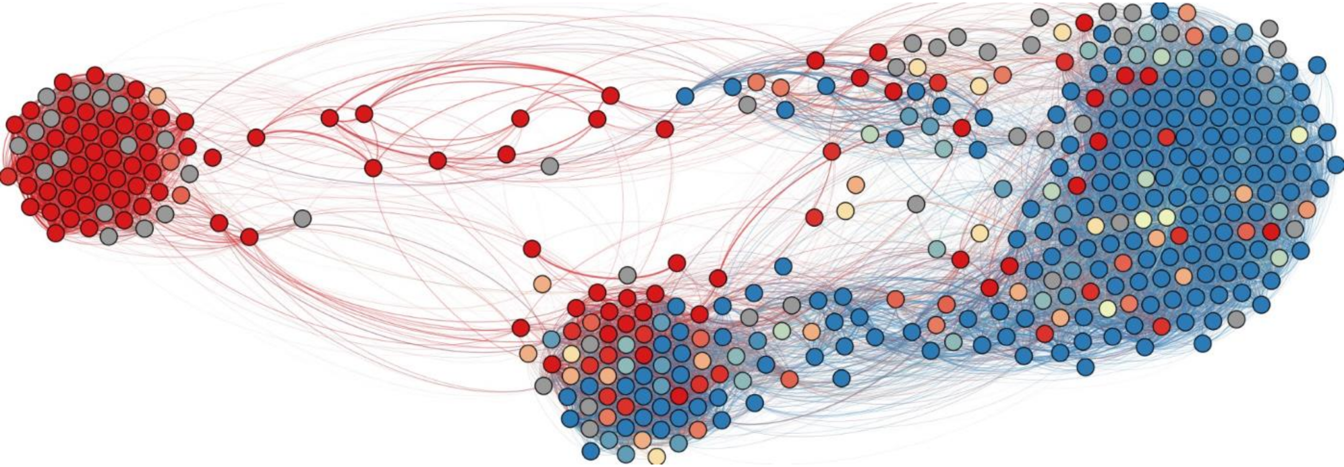

maspin

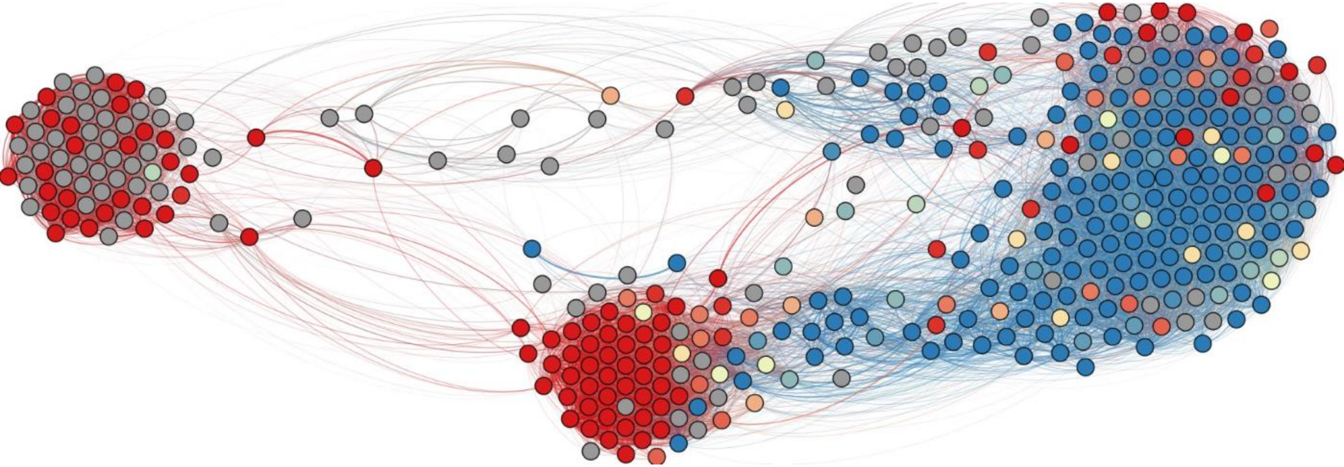

wt1cyt

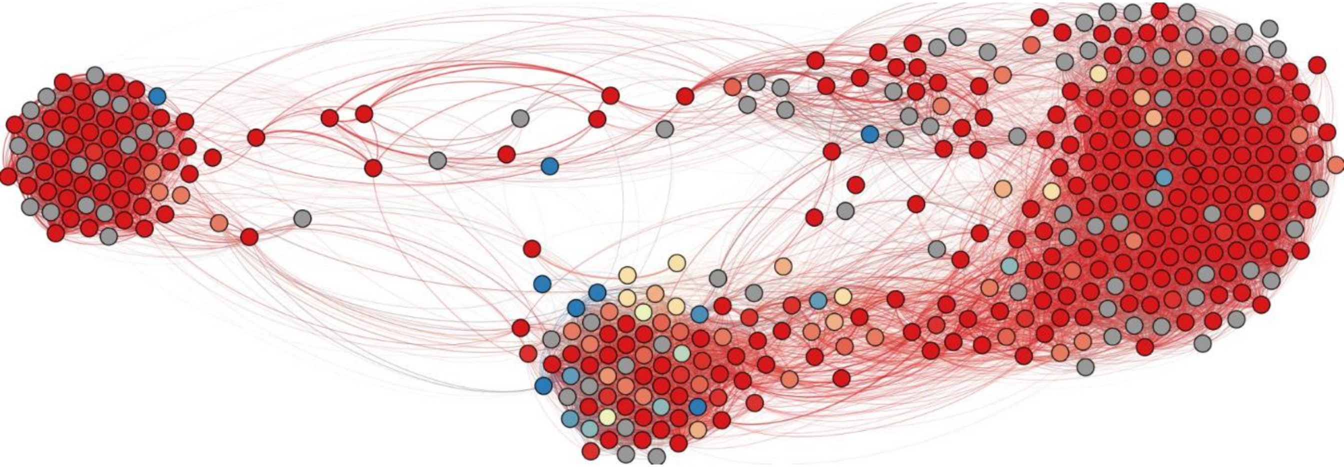

cdx2

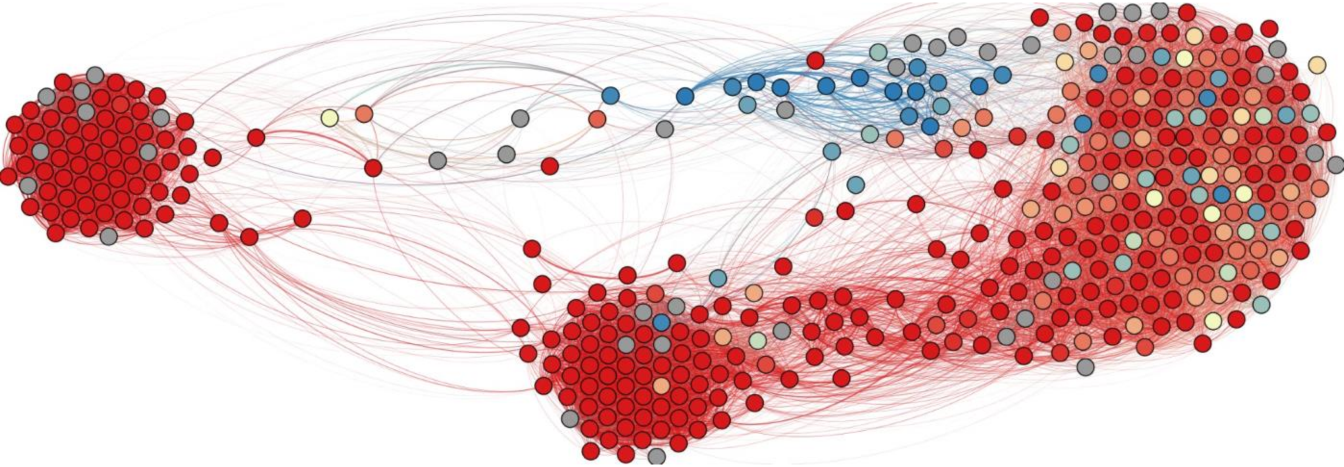

p53

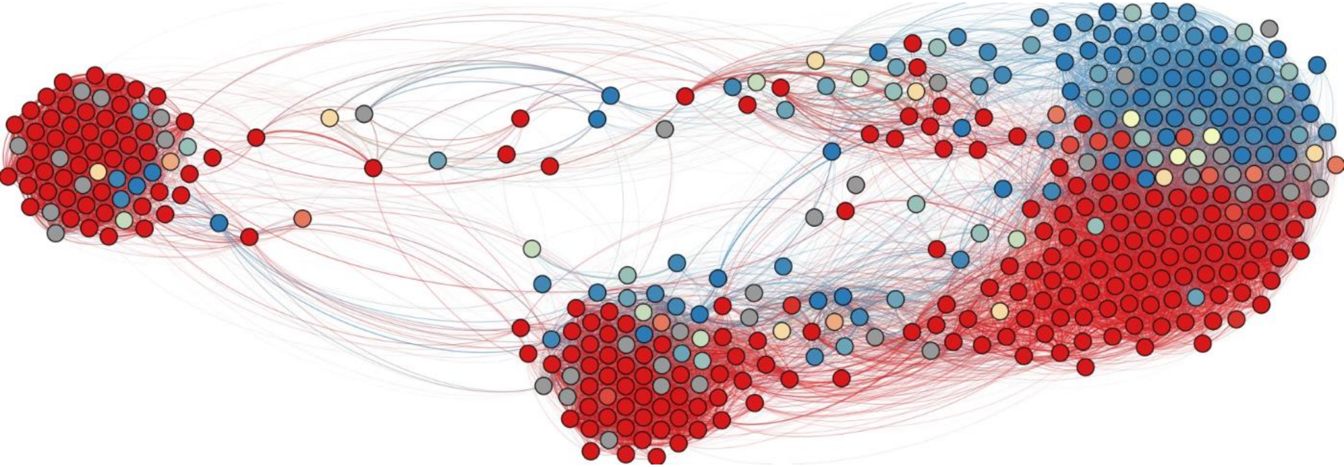

p63

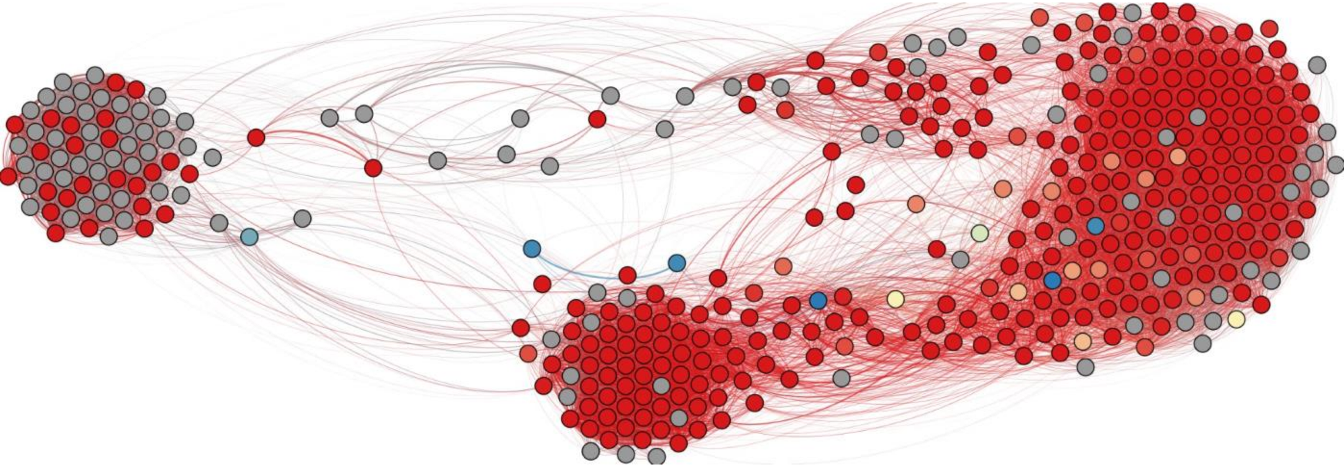

ki67

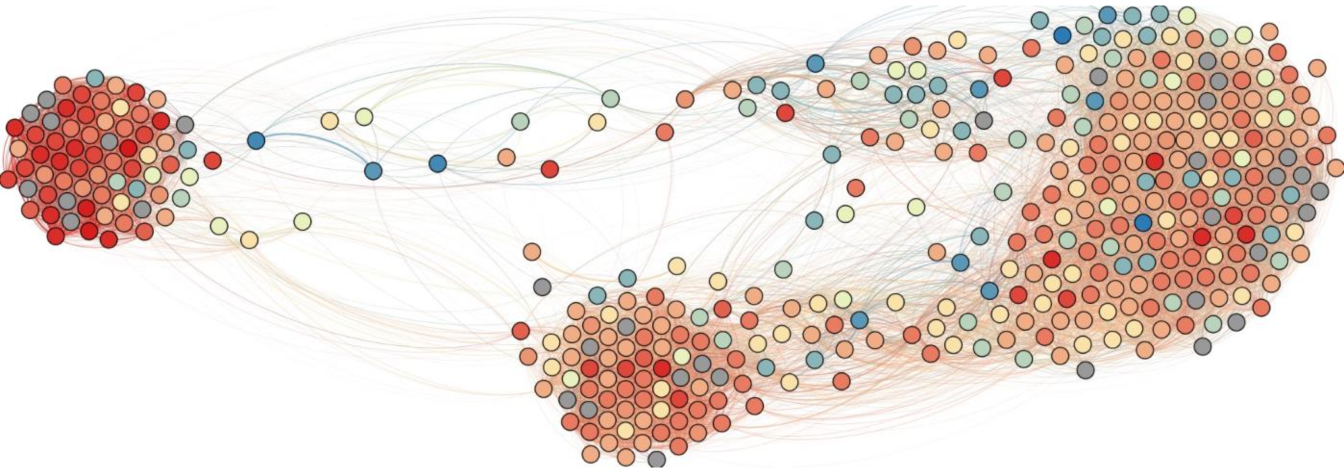

smad4

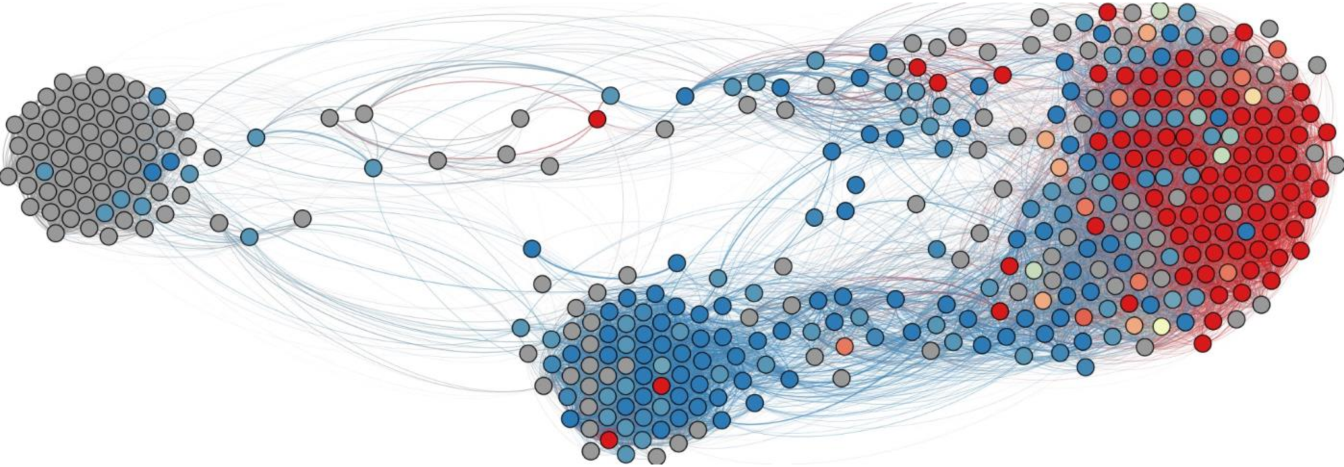

chra

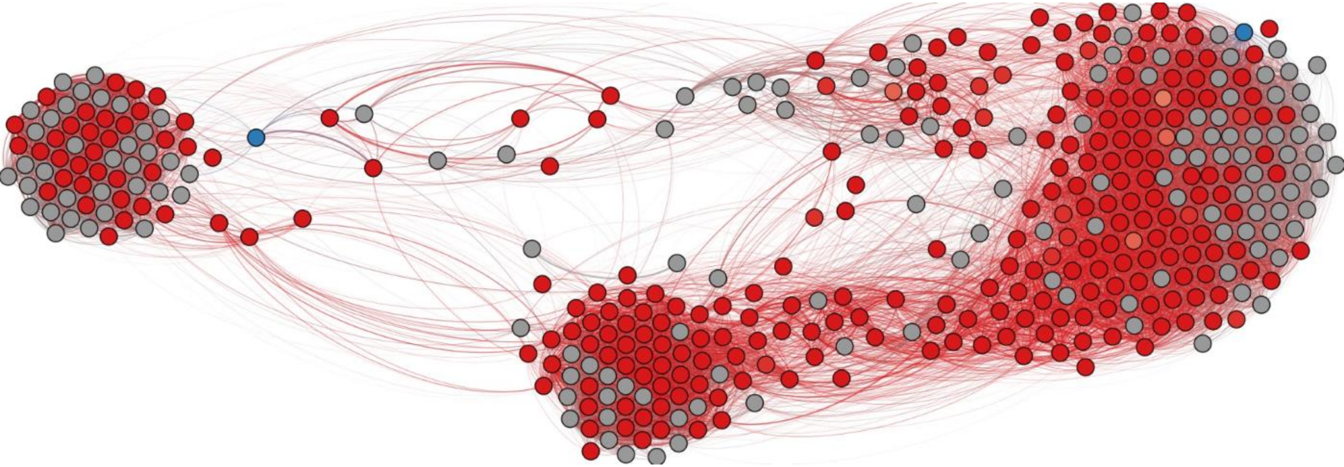

cd56

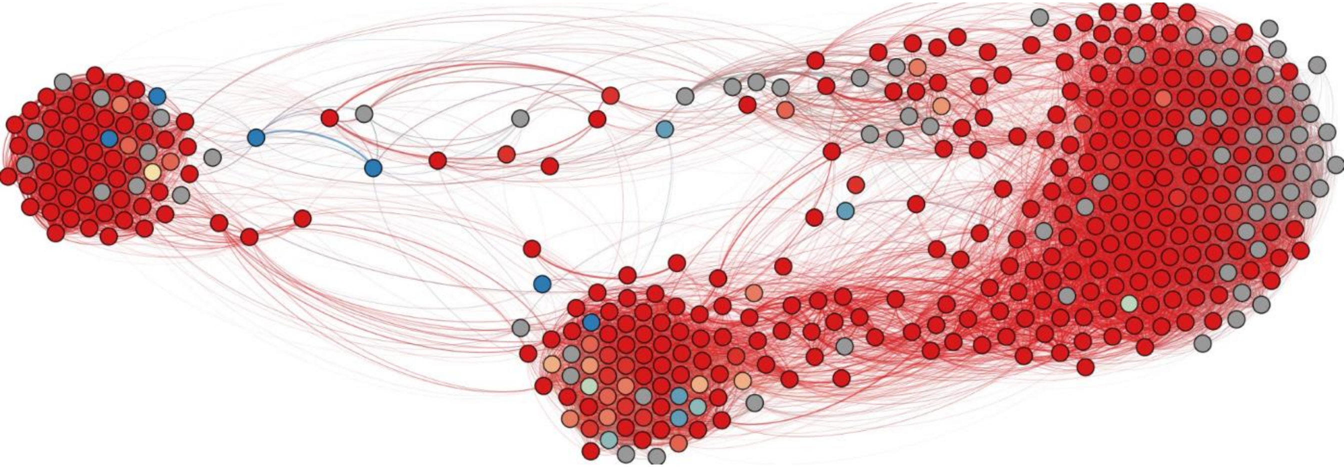

cd10

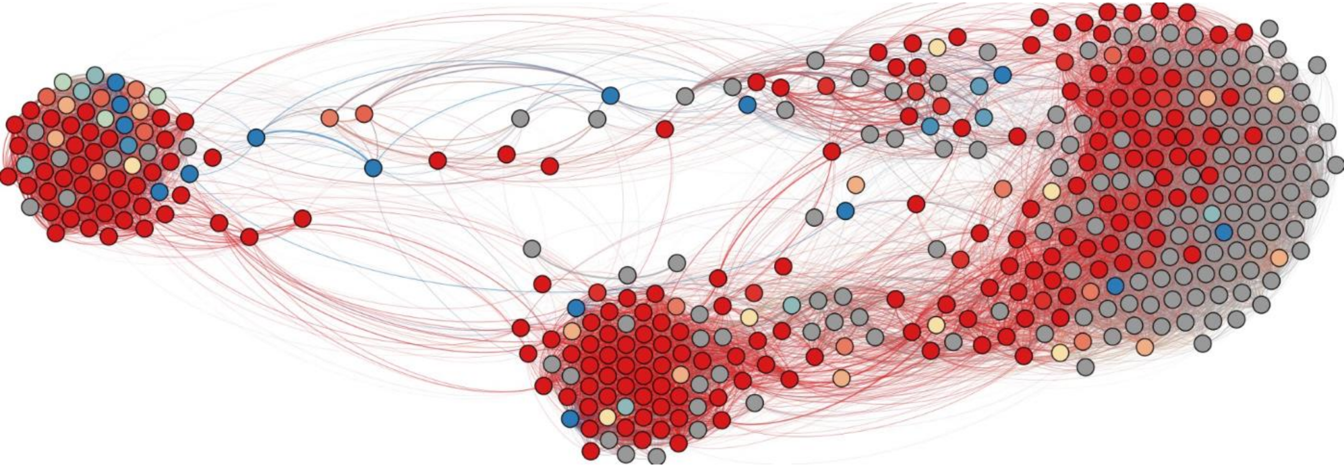

Supplement: S3 Fig — (PDF) [file pone.0166067.s007.pdf]
