# Supplementary material for: Immunohistochemical Typing of Adenocarcinomas of the Pancreatobiliary System Improves Diagnosis and Prognostic Stratification
Source: PLoS One. 2016 Nov 9;11(11):e0166067. doi: 10.1371/journal.pone.0166067 (PMC5102456; doi:10.1371/journal.pone.0166067)
Supplement: S3 File — (PDF) [file pone.0166067.s008.pdf]

Supplementary File 3

Immunoprofiling of adenocarcinomas of the pancreatobiliary tree

Survival analysis - resected patients

Generated on: 2016-08-07 19:47:49

Import required packages

```
# Build pre-requisites
# install.packages("survival")
# install.packages("AICcmodavg")
# install.packages("pastecs")
# install.packages("rms")
# install.packages("xtable")

library(survival)
```

```
## Loading required package: splines
```

```
library(AICcmodavg)
library(pastecs)
```

```
## Loading required package: boot
##
## Attaching package: 'boot'
##
## The following object is masked from 'package:survival':
##
##     aml
```

```
library(rms)
```

```
## Loading required package: Hmisc
## Loading required package: grid
## Loading required package: lattice
##
## Attaching package: 'lattice'
##
## The following object is masked from 'package:boot':
##
##     melanoma
##
## Loading required package: Formula
## Loading required package: ggplot2
##
## Attaching package: 'Hmisc'
##
## The following objects are masked from 'package:base':
##
##     format.pval, round.POSIXt, trunc.POSIXt, units
##
## Loading required package: SparseM
##
## Attaching package: 'SparseM'
##
## The following object is masked from 'package:base':
##
##     backsolve
##
##
## Attaching package: 'rms'
##
## The following object is masked from 'package:pastecs':
##
##     specs
```

```
library(xtable)
```

```
##
## Attaching package: 'xtable'
##
## The following objects are masked from 'package:Hmisc':
##
##     label, label<-
```

Configure file names

```
#myWorkDir <- "~/Workspace/research/pcbil/"
myWorkDirectory <- "/home/guest/pcbil/"

survDataFileName <- paste(myWorkDirectory, "data_analysis/tidy_datasets/pcbi
l_survival.csv", sep="")
```

Import all survival data

```
completeSurvData <- read.csv(file = survDataFileName, na.strings = "")

completeSurvData$date_birth <- as.Date(completeSurvData$date_birth)
completeSurvData$date_pad <- as.Date(completeSurvData$date_pad)
completeSurvData$date_death <- as.Date(completeSurvData$date_death)

# Remove unclassified from immunohistochemial clusters, as they are not a re
al cluster
# and hepatocellular carcinoma, as it was only the control group for the clu
stering
table(completeSurvData$cluster, useNA = "always")
```

```
##
##   extrahepatic pancreatobiliary        hepatocellular carcinoma
##                             172                              58
##                      intestinal intrahepatic cholangiocarcinoma
##                              21                              74
##                    unclassified                            <NA>
##                              31                               0
```

```
completeSurvData[ completeSurvData$cluster == "unclassified" , c("cluster")
] <- NA
completeSurvData$cluster <- droplevels(completeSurvData$cluster)
table(completeSurvData$cluster, useNA = "always")
```

```
##
##   extrahepatic pancreatobiliary        hepatocellular carcinoma
##                             172                              58
##                      intestinal intrahepatic cholangiocarcinoma
##                              21                              74
##                            <NA>
##                              31
```

```
# Relevel clusters
completeSurvData$cluster = factor(completeSurvData$cluster,levels(completeSu
rvData$cluster)[c(1,3,4,2)])

# Relevel clinical diagnoses
completeSurvData$clin_diag = factor(completeSurvData$clin_diag,levels(comple
teSurvData$clin_diag)[c(3, 1, 2, 4, 7, 6, 5)])

# Relevel so the largest group (pT3) is baseline category for cox and hr est
imates
table(completeSurvData$p_t)
```

```
##
##  1  2  3  4 is
## 10 35 41 12  1
```

```
completeSurvData$p_t <- relevel(completeSurvData$p_t, "3")
completeSurvData <- subset(completeSurvData, clin_diag != "Hepatocellular ca
rcinoma")

# Integrity check
str(completeSurvData)
```

```
## 'data.frame':    289 obs. of  14 variables:
##  $ pad           : Factor w/ 356 levels "Ampulla Ac|124",..: 1 2 3 4 5 6
7 8 9 10 ...
##  $ date_birth    : Date, format: "1936-03-05" "1917-05-27" ...
##  $ gender        : Factor w/ 2 levels "f","m": 2 1 1 2 2 1 2 2 1 2 ...
##  $ date_pad      : Date, format: "2005-04-26" "2005-09-20" ...
##  $ type_probe    : Factor w/ 2 levels "b","r": 2 1 1 2 1 2 1 2 2 2 ...
##  $ p_t           : Factor w/ 5 levels "3","1","2","4",..: 1 NA NA 3 NA 2
NA 1 1 2 ...
##  $ p_n           : Factor w/ 4 levels "0","1","2","x": 2 NA NA 1 NA 1 NA
2 1 1 ...
##  $ date_death    : Date, format: "2007-04-13" "2006-05-28" ...
##  $ age_pad       : num  830 1060 956 893 929 ...
##  $ age_pad_years : num  69.1 88.3 79.7 74.4 77.5 ...
##  $ surv_time     : num  23.59 8.26 10.23 3.87 0.16 ...
##  $ death_observed: int  1 1 1 1 1 1 1 0 1 1 ...
##  $ clin_diag     : Factor w/ 7 levels "Ductal pancreatic adenocarcinom
a",..: 2 2 2 2 2 2 2 2 2 2 ...
##  $ cluster       : Factor w/ 4 levels "extrahepatic pancreatobiliar
y",..: 1 1 1 1 2 2 1 2 NA 1 ...
```

```
table(is.na(completeSurvData$type_probe))
```

```
##
## FALSE
##   289
```

```
# Survival summary, whole series
dim(completeSurvData)
```

```
## [1] 289  14
```

```
summary(completeSurvData$surv_time)
```

```
##    Min. 1st Qu.  Median    Mean 3rd Qu.    Max.
##    0.03    2.86    8.03   18.14   20.96  143.40
```

```
round(stat.desc(completeSurvData$surv_time, basic =  FALSE, norm = TRUE), di
gits=3)
```

```
##       median         mean      SE.mean CI.mean.0.95          var
##        8.030       18.144        1.480        2.912      632.765
##      std.dev     coef.var     skewness     skew.2SE     kurtosis
##       25.155        1.386        2.346        8.183        5.838
##     kurt.2SE   normtest.W   normtest.p
##       10.216        0.687        0.000
```

```
# Obtain resection data set AND filter out early postoperative death (2 mont
hs)
resCompleteSurvData <- subset(completeSurvData, (type_probe == "r" & surv_ti
me > 2))
resCompleteSurvData$cluster <- droplevels(resCompleteSurvData$cluster)
resCompleteSurvData$clin_diag <- droplevels(resCompleteSurvData$clin_diag)
```

Function to generate a table report of cox analysis; based on R code: getAnywhere (summary.coxph)

```r
getCoxTable <- function(cox, rows) {

# Prepare the columns
beta <- coef(cox)
HR <- exp(beta)
se   <- sqrt(diag(cox$var))
p    <- 1 - pchisq((beta/se)^2, 1)

conf.int = 0.95
z <- qnorm((1 + conf.int)/2, 0, 1)
LCI  <- exp(beta - z * se)
UCI  <- exp(beta + z * se)

# Bind columns together, and select desired rows
res <- cbind(HR, LCI, UCI, p)
res <- res[rows,]

return(res)
}
```

Type of probe

```r
# Biopsy vs resection
bx_resPcbilSurvFit <- npsurv(formula = Surv(surv_time, death_observed) ~ typ
e_probe, data = completeSurvData)
print(bx_resPcbilSurvFit, print.rmean = TRUE)
```

```
## Call: npsurv(formula = Surv(surv_time, death_observed) ~ type_probe,
##     data = completeSurvData)
##
##              records n.max n.start events *rmean *se(rmean) median 0.95LC
L
## type_probe=b     199   199     199    194   9.76       1.17   6.19    4.9
6
## type_probe=r      90    90      90     67  46.53       4.57  30.73   21.6
6
##              0.95UCL
## type_probe=b     6.9
## type_probe=r    36.7
##     * restricted mean with upper limit =  114
```

```r
survplot(bx_resPcbilSurvFit, conf = c("none","bands","bars")[1], xlim = c
(0, 150), main = "KM - Biopsy vs resection", xlab = "Time (months)", ylab =
"Survival", time.inc = 10, n.risk   = TRUE,  y.n.risk = -0.3, cex.n.risk =
0.6) # Add to shor 6-yrs, xlim = c(0, 72) # Remove CI c("none","bands","bar
s")[1]
```

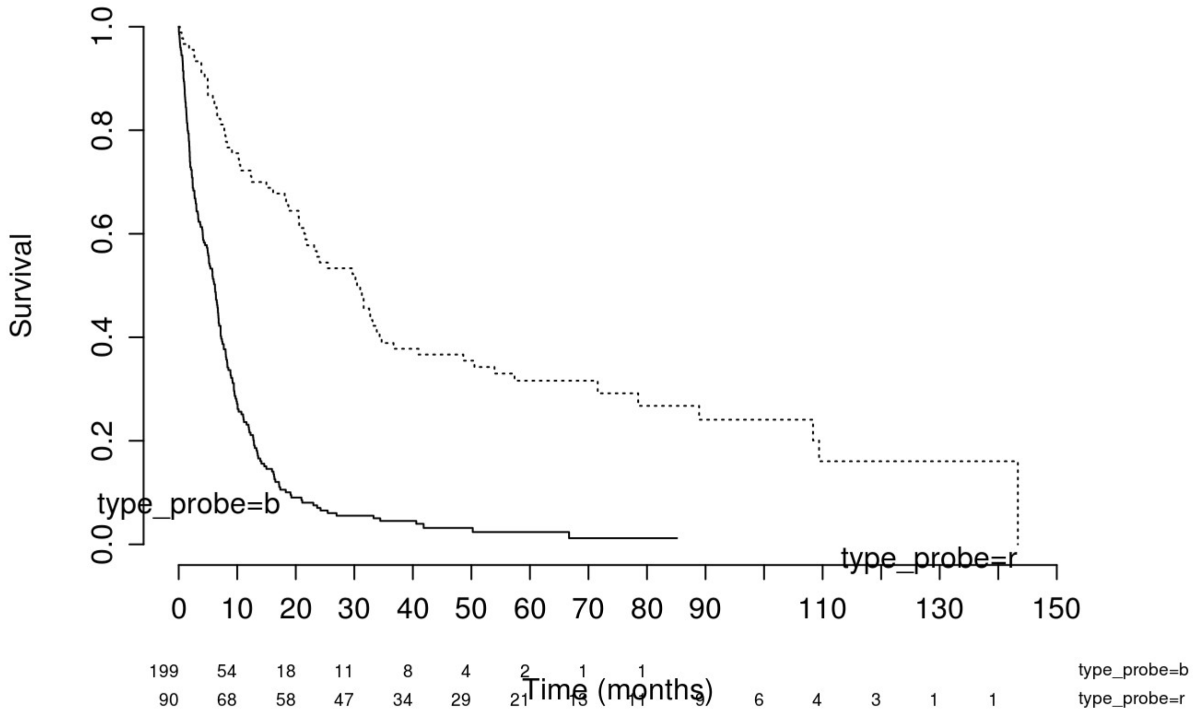


```
# Univariate: Cox proportional hazards regression model.
summary(coxph(Surv(surv_time, death_observed) ~ type_probe, data = completeS
urvData))
```

```
## Call:
## coxph(formula = Surv(surv_time, death_observed) ~ type_probe,
##     data = completeSurvData)
##
##   n= 289, number of events= 261
##
##                  coef exp(coef) se(coef)      z Pr(>|z|)
## type_prober -1.3720    0.2536   0.1551 -8.844   <2e-16 ***
## ---
## Signif. codes:  0 '***' 0.001 '**' 0.01 '*' 0.05 '.' 0.1 ' ' 1
##
##             exp(coef) exp(-coef) lower .95 upper .95
## type_prober    0.2536      3.943    0.1871    0.3437
##
## Concordance= 0.638  (se = 0.017 )
## Rsquare= 0.271   (max possible= 1 )
## Likelihood ratio test= 91.25  on 1 df,   p=0
## Wald test            = 78.22  on 1 df,   p=0
## Score (logrank) test = 86.73  on 1 df,   p=0
```

Age

```
# Missing data check
table(is.na(resCompleteSurvData$age_pad_years))
```

```
##
## FALSE
##    86
```

```
# descriptives age surgery
summary(resCompleteSurvData$age_pad_years)
```

```
##    Min. 1st Qu.  Median    Mean 3rd Qu.    Max.
##   32.16   59.29   65.68   64.75   72.08   82.74
```

```
# Age categories
resCompleteSurvData$age_pad_years_cat <- cut(resCompleteSurvData$age_pad_yea
rs, breaks=c(0, 66, 83))
head(resCompleteSurvData[, c("age_pad_years", "age_pad_years_cat")])
```

```
##    age_pad_years age_pad_years_cat
## 1          69.14           (66,83]
## 4          74.40           (66,83]
## 6          52.86            (0,66]
## 8          36.82            (0,66]
## 9          78.86           (66,83]
## 10         70.20           (66,83]
```

```
# Relevel age cats
resCompleteSurvData$age_pad_years_cat = factor(resCompleteSurvData$age_pad_y
ears_cat, levels(resCompleteSurvData$age_pad_years_cat)[c(2,1)])


# KM
age_pad_years_cat_resPcbilSurvFit <- npsurv(formula = Surv(surv_time, death_
observed) ~ age_pad_years_cat, data = resCompleteSurvData)
print(age_pad_years_cat_resPcbilSurvFit, print.rmean = TRUE)
```

```
## Call: npsurv(formula = Surv(surv_time, death_observed) ~ age_pad_years_ca
t,
##     data = resCompleteSurvData)
##
##                             records n.max n.start events *rmean *se(rmean)
## age_pad_years_cat=(66,83]       42    42      42     31   54.7       7.87
## age_pad_years_cat=(0,66]        44    44      44     32   49.4       7.53
##                             median 0.95LCL 0.95UCL
## age_pad_years_cat=(66,83]   32.1    24.1    78.5
## age_pad_years_cat=(0,66]    30.3    18.7    57.4
##     * restricted mean with upper limit =  136
```

```
survplot(age_pad_years_cat_resPcbilSurvFit, conf = c("none","bands","bars")
[1], xlim = c(0, 150), main = "KM - By age", xlab = "Time (months)", ylab =
"Survival", time.inc = 10, n.risk   = TRUE,  y.n.risk = -0.3, cex.n.risk =
0.6) # Add to shor 6-yrs, xlim = c(0, 72) # Remove CI c("none","bands","bar
s")[1]
```

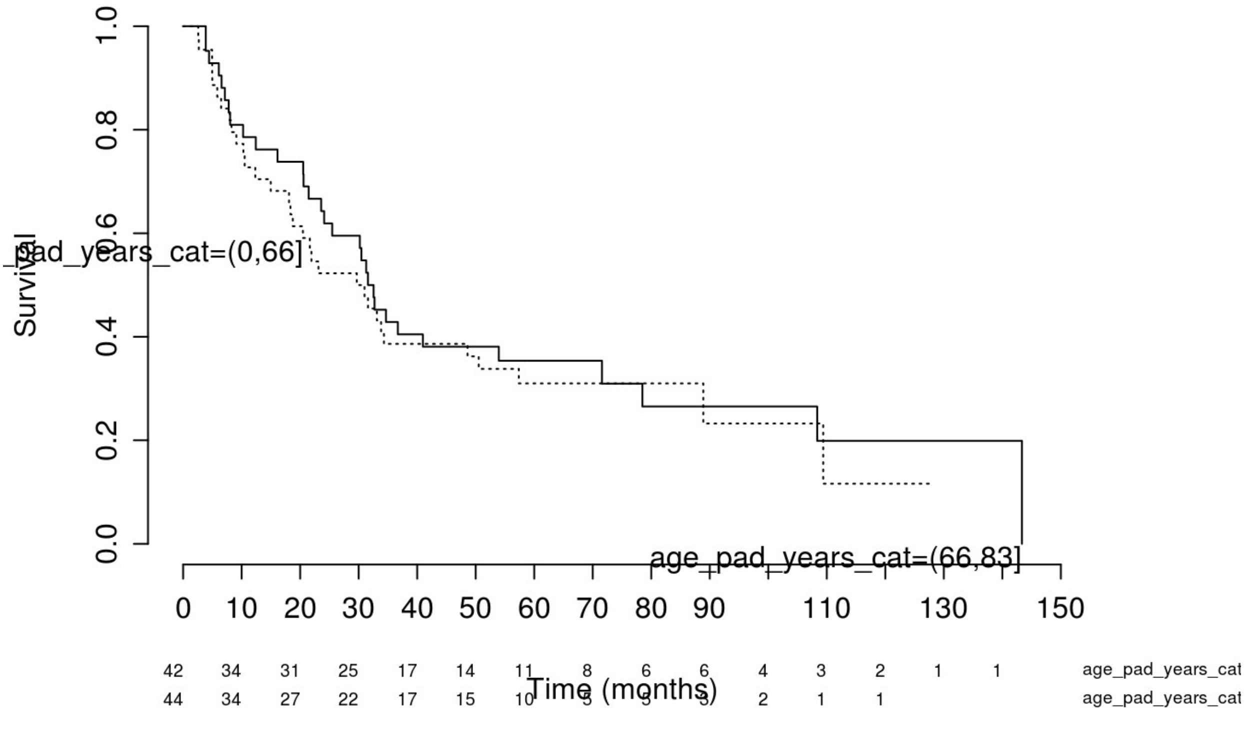


```
# Univariate: Cox proportional hazards regression model.
summary(coxph(Surv(surv_time, death_observed) ~ age_pad_years_cat, data = re
sCompleteSurvData))
```

```
## Call:
## coxph(formula = Surv(surv_time, death_observed) ~ age_pad_years_cat,
##     data = resCompleteSurvData)
##
##   n= 86, number of events= 63
##
##                           coef exp(coef) se(coef)     z Pr(>|z|)
## age_pad_years_cat(0,66] 0.1331    1.1424   0.2547 0.522    0.601
##
##                         exp(coef) exp(-coef) lower .95 upper .95
## age_pad_years_cat(0,66]     1.142     0.8754    0.6934     1.882
##
## Concordance= 0.522  (se = 0.035 )
## Rsquare= 0.003   (max possible= 0.996 )
## Likelihood ratio test= 0.27  on 1 df,   p=0.6012
## Wald test            = 0.27  on 1 df,   p=0.6013
## Score (logrank) test = 0.27  on 1 df,   p=0.6011
```

Gender

```
# Missing data check
table(is.na(resCompleteSurvData$gender))
```

```
##
## FALSE
##    86
```

```
# descriptives gender
summary(resCompleteSurvData$gender)
```

```
##  f  m
## 47 39
```

```
# KM
gender_resPcbilSurvFit <- npsurv(formula = Surv(surv_time, death_observed)
~ gender, data = resCompleteSurvData)
print(gender_resPcbilSurvFit, print.rmean = TRUE)
```

```
## Call: npsurv(formula = Surv(surv_time, death_observed) ~ gender, data = r
esCompleteSurvData)
##
##            records n.max n.start events *rmean *se(rmean) median 0.95LCL
## gender=f        47    47      47     37   47.2       6.87   29.7    18.1
## gender=m        39    39      39     26   54.4       7.77   33.1    24.1
##            0.95UCL
## gender=f        41
## gender=m        NA
##      * restricted mean with upper limit =  128
```

```
survplot(gender_resPcbilSurvFit, conf = c("none","bands","bars")[1], xlim =
c(0, 150), main = "KM - By gender", xlab = "Time (months)", ylab = "Surviva
l", time.inc = 10, n.risk   = TRUE,  y.n.risk = -0.3, cex.n.risk = 0.6) # Ad
d to shor 6-yrs, xlim = c(0, 72) # Remove CI c("none","bands","bars")[1]
```

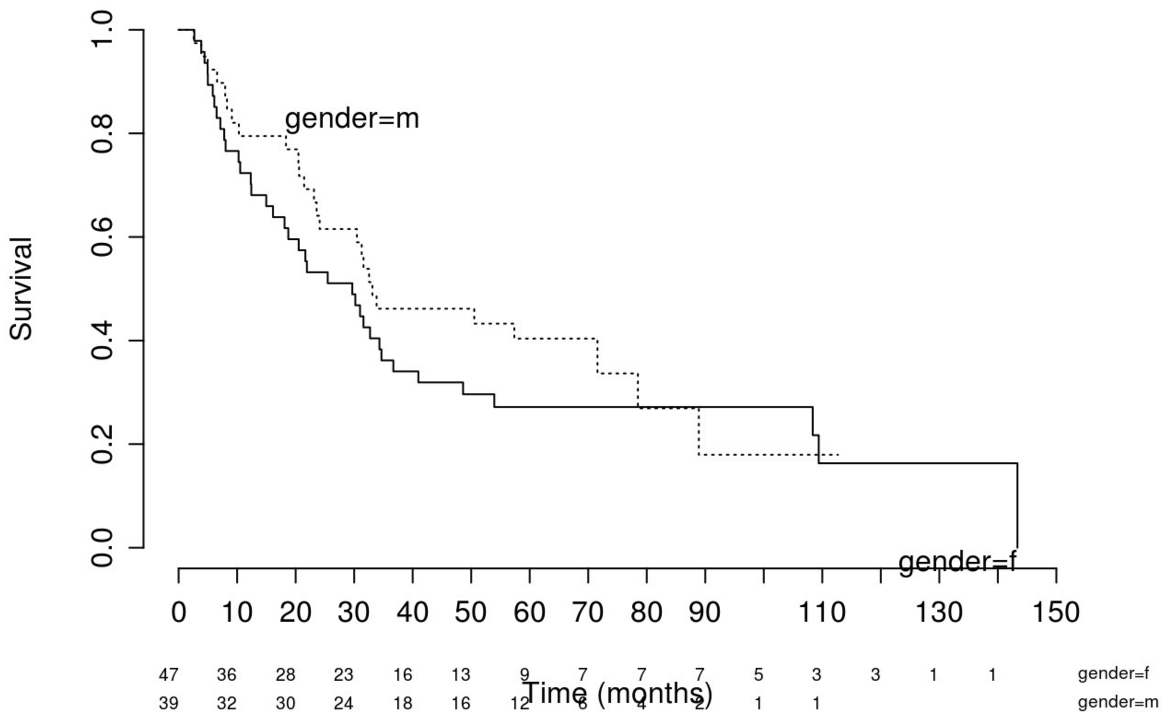


```
# Univariate:
coxGender <- coxph(Surv(surv_time, death_observed) ~ gender, data = resCompl
eteSurvData)
summary(coxGender)
```

```
## Call:
## coxph(formula = Surv(surv_time, death_observed) ~ gender, data = resCompl
eteSurvData)
##
##   n= 86, number of events= 63
##
##            coef exp(coef) se(coef)      z Pr(>|z|)
## genderm -0.2565    0.7738   0.2595 -0.988    0.323
##
##         exp(coef) exp(-coef) lower .95 upper .95
## genderm    0.7738      1.292    0.4653     1.287
##
## Concordance= 0.546  (se = 0.035 )
## Rsquare= 0.011   (max possible= 0.996 )
## Likelihood ratio test= 0.99  on 1 df,   p=0.3204
## Wald test            = 0.98  on 1 df,   p=0.3229
## Score (logrank) test = 0.98  on 1 df,   p=0.3216
```

pT

```
# Missing data check
table(is.na(resCompleteSurvData$p_t))
```

```
##
## FALSE
##    86
```

```
# descriptives p_t
summary(resCompleteSurvData$p_t)
```

```
##  3  1  2  4 is
## 39 10 27  9  1
```

```
# KM
p_t_resPcbilSurvFit <- npsurv(formula = Surv(surv_time, death_observed) ~ p_
t, data = resCompleteSurvData)
print(p_t_resPcbilSurvFit, print.rmean = TRUE)
```

```
## Call: npsurv(formula = Surv(surv_time, death_observed) ~ p_t, data = resC
ompleteSurvData)
##
##          records n.max n.start events *rmean *se(rmean) median 0.95LCL
## p_t=3         39    39      39     31   42.6       6.30  25.48   21.45
## p_t=1         10    10      10      6   75.3      11.62  78.49   32.55
## p_t=2         27    27      27     18   55.5       8.38  34.32   29.68
## p_t=4          9     9       9      8   18.4      11.14   7.13    5.83
## p_t=is         1     1       1      0  112.7       0.00     NA      NA
##          0.95UCL
## p_t=3       36.7
## p_t=1         NA
## p_t=2         NA
## p_t=4         NA
## p_t=is        NA
##      * restricted mean with upper limit =  113
```

```
survplot(p_t_resPcbilSurvFit, conf = c("none","bands","bars")[1], xlim = c
(0, 150), main = "KM - By pT", xlab = "Time (months)", ylab = "Survival", ti
me.inc = 10, n.risk   = TRUE,  y.n.risk = -0.3, cex.n.risk = 0.6) # Add to s
hor 6-yrs, xlim = c(0, 72) # Remove CI c("none","bands","bars")[1]
```

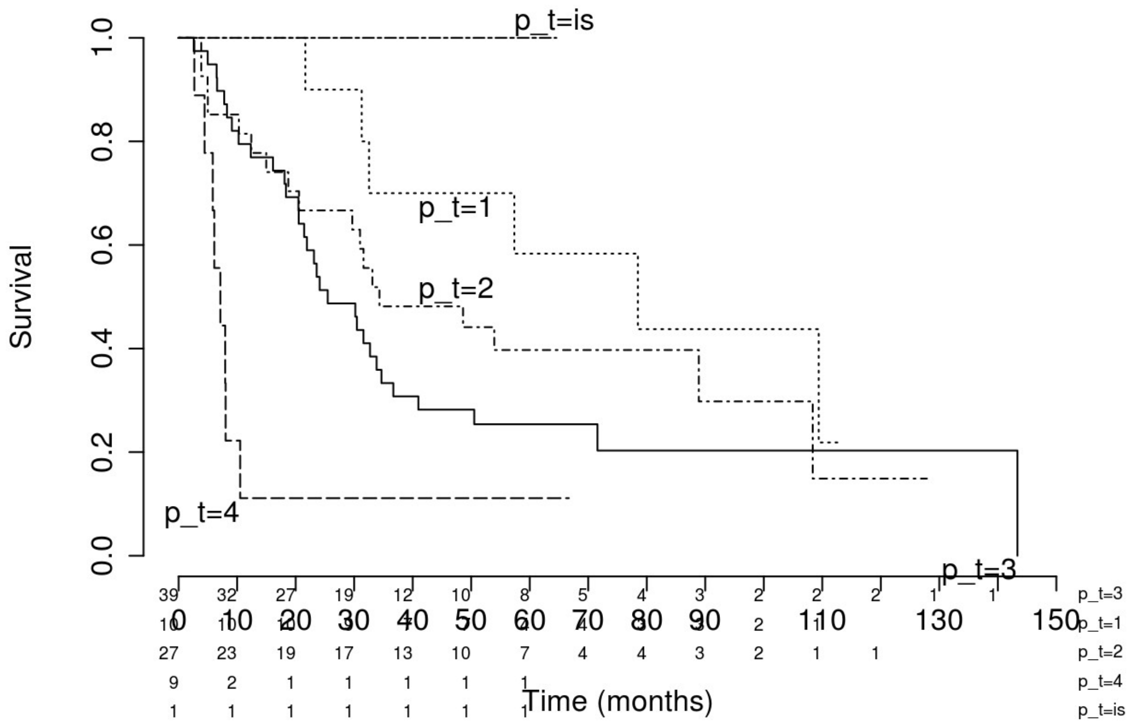


```
# Univariate
coxpT <- coxph(Surv(surv_time, death_observed) ~ p_t, data = resCompleteSurv
Data)
```

```
## Warning in fitter(X, Y, strats, offset, init, control, weights = weights,
:
## Loglik converged before variable 4 ; beta may be infinite.
```

```
summary(coxpT)
```

```
## Call:
## coxph(formula = Surv(surv_time, death_observed) ~ p_t, data = resComplete
SurvData)
##
##   n= 86, number of events= 63
##
##              coef  exp(coef)   se(coef)      z Pr(>|z|)
## p_t1  -7.858e-01  4.558e-01  4.507e-01 -1.743  0.08129 .
## p_t2  -2.917e-01  7.470e-01  2.992e-01 -0.975  0.32956
## p_t4   1.215e+00  3.369e+00  4.095e-01  2.966  0.00302 **
## p_tis -1.653e+01  6.633e-08  3.463e+03 -0.005  0.99619
## ---
## Signif. codes:  0 '***' 0.001 '**' 0.01 '*' 0.05 '.' 0.1 ' ' 1
##
##       exp(coef) exp(-coef) lower .95 upper .95
## p_t1  4.558e-01  2.194e+00    0.1884     1.103
## p_t2  7.470e-01  1.339e+00    0.4155     1.343
## p_t4  3.369e+00  2.968e-01    1.5097     7.517
## p_tis 6.633e-08  1.508e+07    0.0000       Inf
##
## Concordance= 0.644  (se = 0.037 )
## Rsquare= 0.165   (max possible= 0.996 )
## Likelihood ratio test= 15.47  on 4 df,   p=0.003816
## Wald test            = 15.81  on 4 df,   p=0.003287
## Score (logrank) test = 19.81  on 4 df,   p=0.0005448
```

```
# Test the Proportional Hazards Assumption of a Cox Regression
# when the test is non-significant, indicates an absence of evidence to cont
radict the proportionality assumption
cox_pha <- cox.zph(coxpT)
print(cox_pha)
```

```
##             rho    chisq      p
## p_t1    0.1986 2.29e+00 0.1303
## p_t2   -0.0172 1.80e-02 0.8932
## p_t4   -0.2203 2.75e+00 0.0971
## p_tis  -0.1545 3.07e-09 1.0000
## GLOBAL      NA 6.05e+00 0.1955
```

```
plot(cox_pha)
```

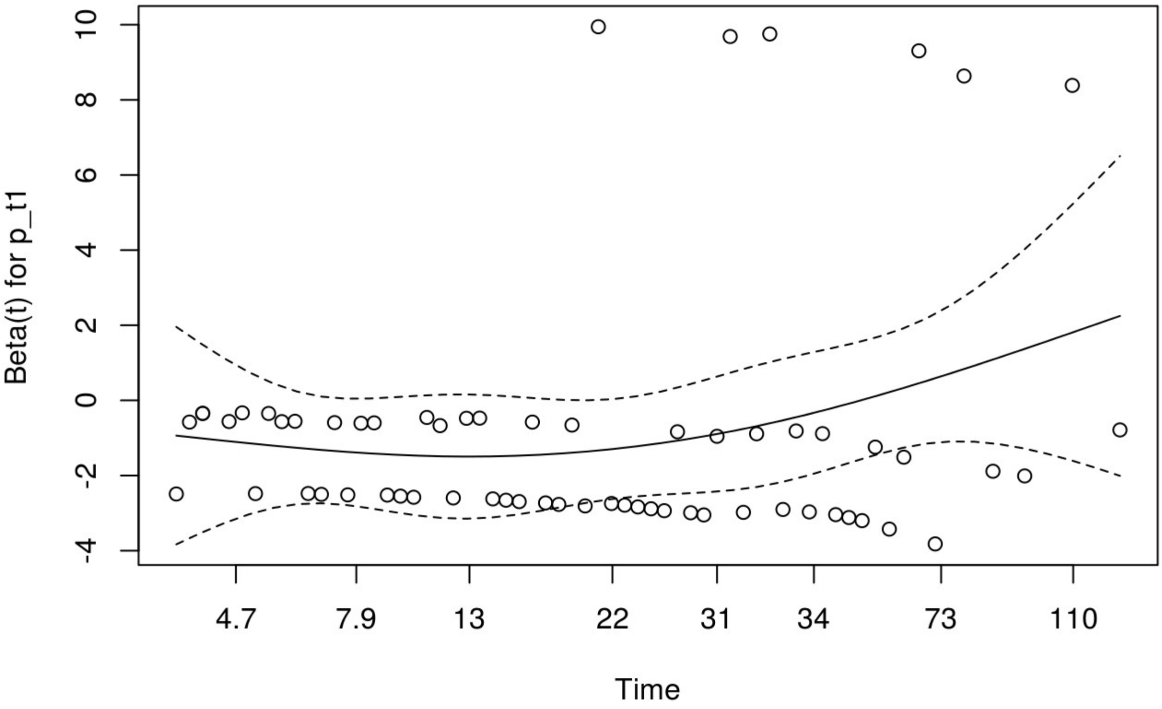
Beta(t) for p_t1
10
8
6
4
2
0
-2
-4
4.7
7.9
13
22
31
34
73
110
Time

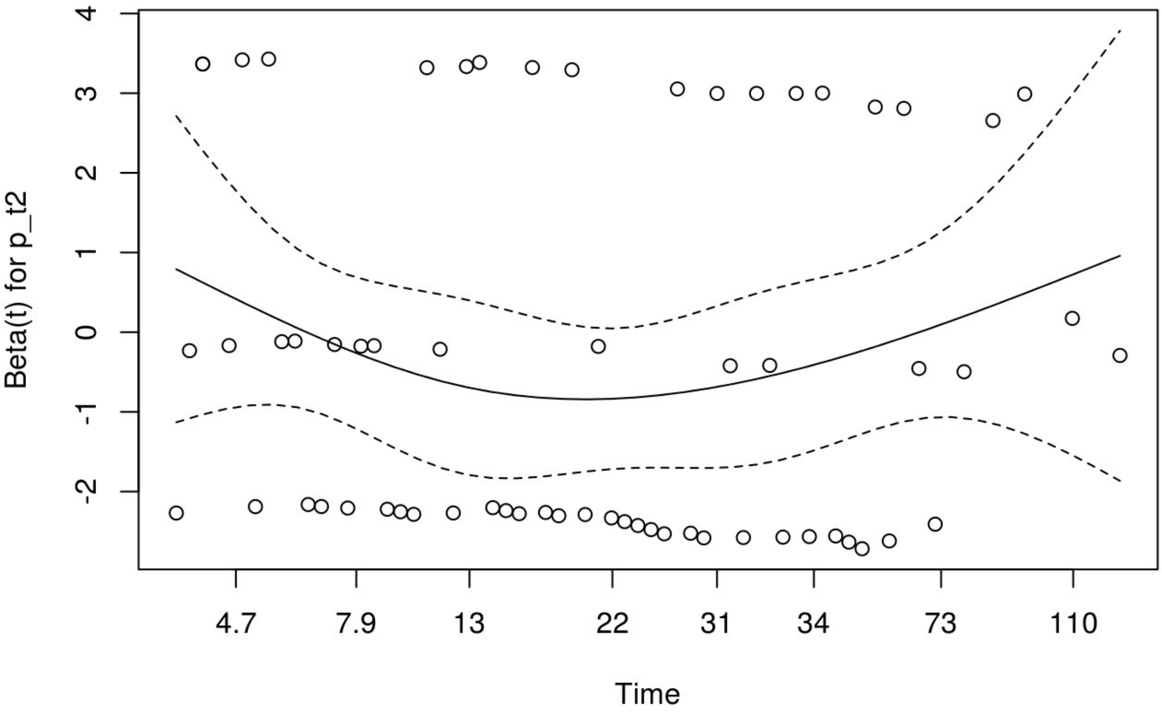
Beta(t) for p_t2
4
3
2
1
0
-1
-2
4.7
7.9
13
22
31
34
73
110
Time

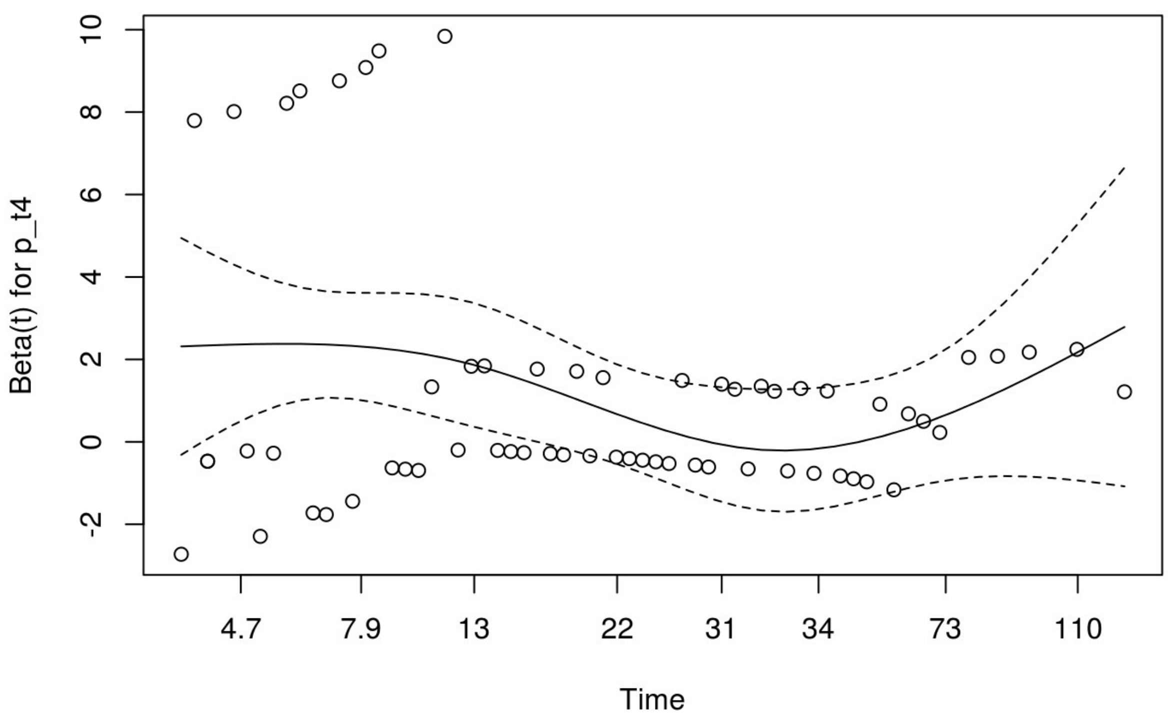

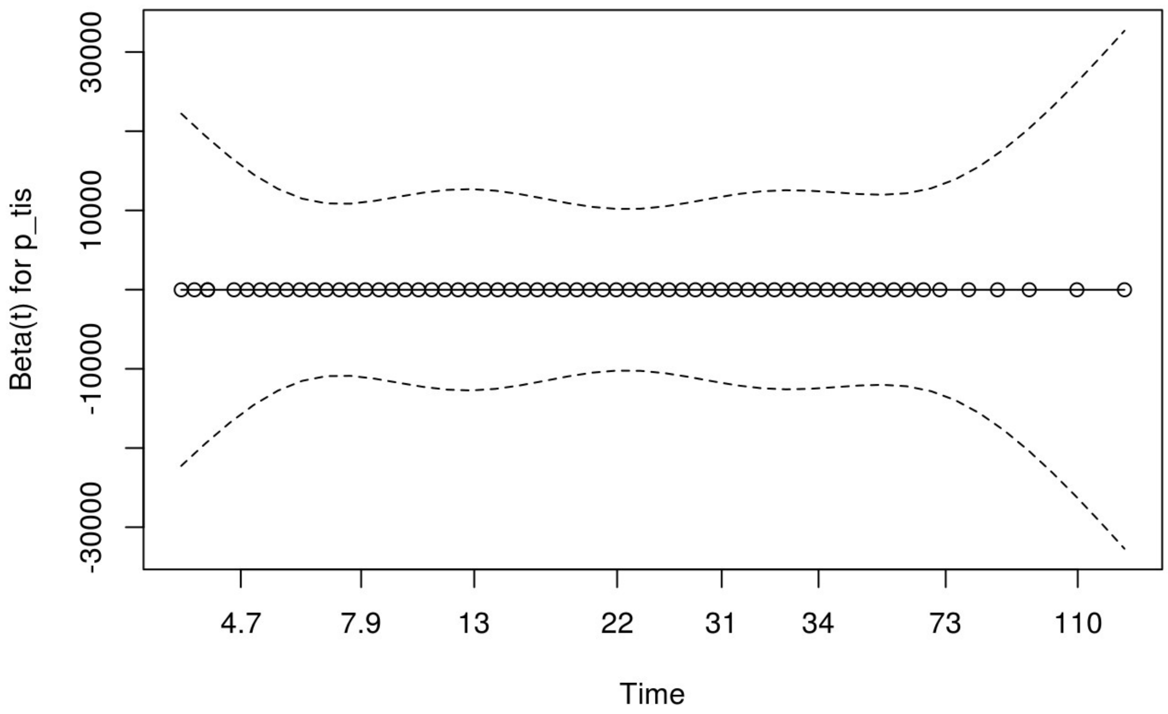

Survival table data: p_t

```
rowsOfInterest <- c( paste("p_t", levels(resCompleteSurvData$p_t)[2 : length
(levels(resCompleteSurvData$p_t))], sep=""))
myXtable <- xtable(getCoxTable(coxpT, rowsOfInterest), caption = 'Survival t
able data: p_t')
digits(myXtable) <- c(0,2,2,2,3)
print(myXtable, type = "html", caption.placement = 'top')
```

Survival table data: p_t

	HR	LCI	UCI	p
p_t1	0.46	0.19	1.10	0.081
p_t2	0.75	0.42	1.34	0.330
p_t4	3.37	1.51	7.52	0.003
p_tis	0.00	0.00	Inf	0.996

pN

```
# Missing data check
table(is.na(resCompleteSurvData$p_n))
```

```
##
## FALSE
##    86
```

```
# descriptives p_t
summary(resCompleteSurvData$p_n)
```

```
##  0  1  2  x
## 28 46  2 10
```

```
# Filter out pNX, as we don't know pN-status and group pN1/2
resCompleteSurvData[ resCompleteSurvData$p_n == "2", ]$p_n <- "1"
resCompleteSurvData[ resCompleteSurvData$p_n == "x", ]$p_n <- NA
resCompleteSurvData$p_n <- droplevels(resCompleteSurvData$p_n)

resCompleteSurvData$p_n <- relevel(resCompleteSurvData$p_n, "1")
summary(resCompleteSurvData$p_n)
```

```
##    1    0 NA's
##   48   28   10
```

```
# KM
p_n_resPcbilSurvFit <- npsurv(formula = Surv(surv_time, death_observed) ~ p_
n, data = resCompleteSurvData)
print(p_n_resPcbilSurvFit, print.rmean = TRUE)
```

```
## Call: npsurv(formula = Surv(surv_time, death_observed) ~ p_n, data = resC
ompleteSurvData)
##
##    10 observations deleted due to missingness
##        records n.max n.start events *rmean *se(rmean) median 0.95LCL
## p_n=1      48    48      48     41   31.9       5.18   19.4    10.3
## p_n=0      28    28      28     17   66.2       9.73   48.6    32.5
##        0.95UCL
## p_n=1    25.5
## p_n=0      NA
##     * restricted mean with upper limit =  126
```

```
survplot(p_n_resPcbilSurvFit, conf = c("none","bands","bars")[1], xlim = c
(0, 150), main = "KM - By pN", xlab = "Time (months)", ylab = "Survival", ti
me.inc = 10, n.risk   = TRUE,  y.n.risk = -0.3, cex.n.risk = 0.6) # Add to s
hor 6-yrs, xlim = c(0, 72) # Remove CI c("none","bands","bars")[1]
```

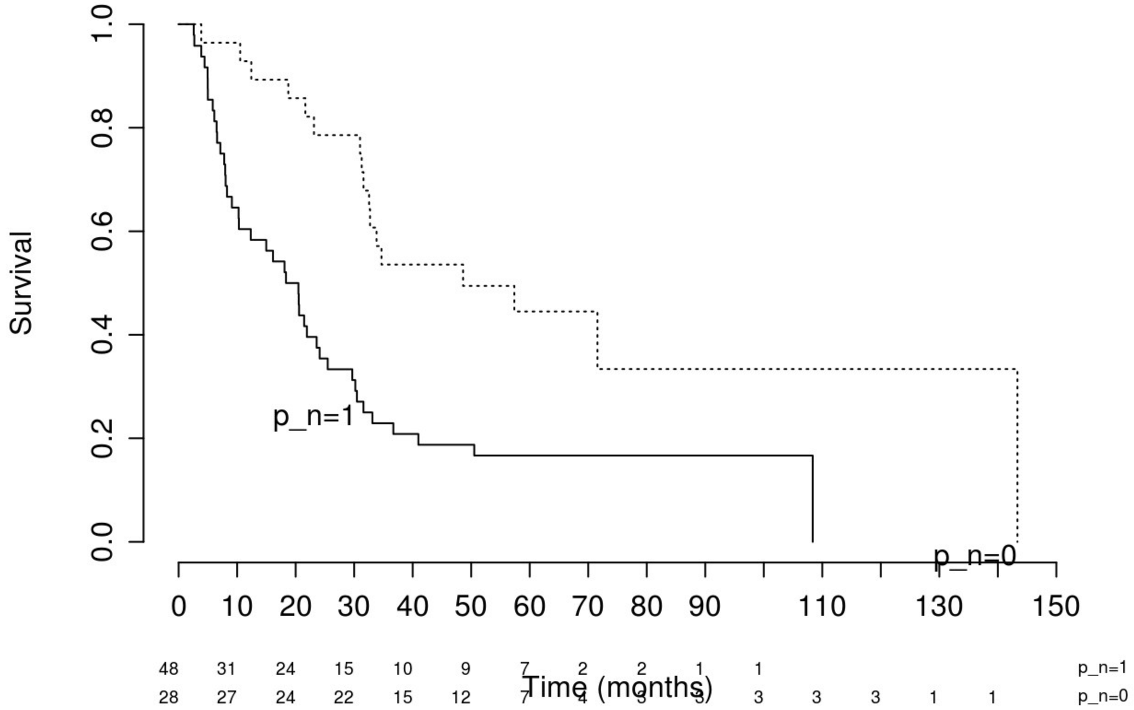


```
# Univariate
coxpT <- coxph(Surv(surv_time, death_observed) ~ p_n, data = resCompleteSurv
Data)
summary(coxpT)
```

```
## Call:
## coxph(formula = Surv(surv_time, death_observed) ~ p_n, data = resComplete
SurvData)
##
##   n= 76, number of events= 58
##    (10 observations deleted due to missingness)
##
##         coef exp(coef) se(coef)      z Pr(>|z|)
## p_n0 -1.0115    0.3637   0.2988 -3.385 0.000712 ***
## ---
## Signif. codes:  0 '***' 0.001 '**' 0.01 '*' 0.05 '.' 0.1 ' ' 1
##
##      exp(coef) exp(-coef) lower .95 upper .95
## p_n0    0.3637       2.75    0.2025    0.6532
##
## Concordance= 0.634  (se = 0.036 )
## Rsquare= 0.155   (max possible= 0.996 )
## Likelihood ratio test= 12.76  on 1 df,   p=0.0003535
## Wald test            = 11.46  on 1 df,   p=0.0007117
## Score (logrank) test = 12.38  on 1 df,   p=0.0004335
```

```
# Test the Proportional Hazards Assumption of a Cox Regression
# when the test is non-significant, indicates an absence of evidence to cont
radict the proportionality assumption
cox_pha <- cox.zph(coxpT)
print(cox_pha)
```

```
##        rho chisq      p
## p_n0 0.237     3 0.0834
```

```
plot(cox_pha)
```

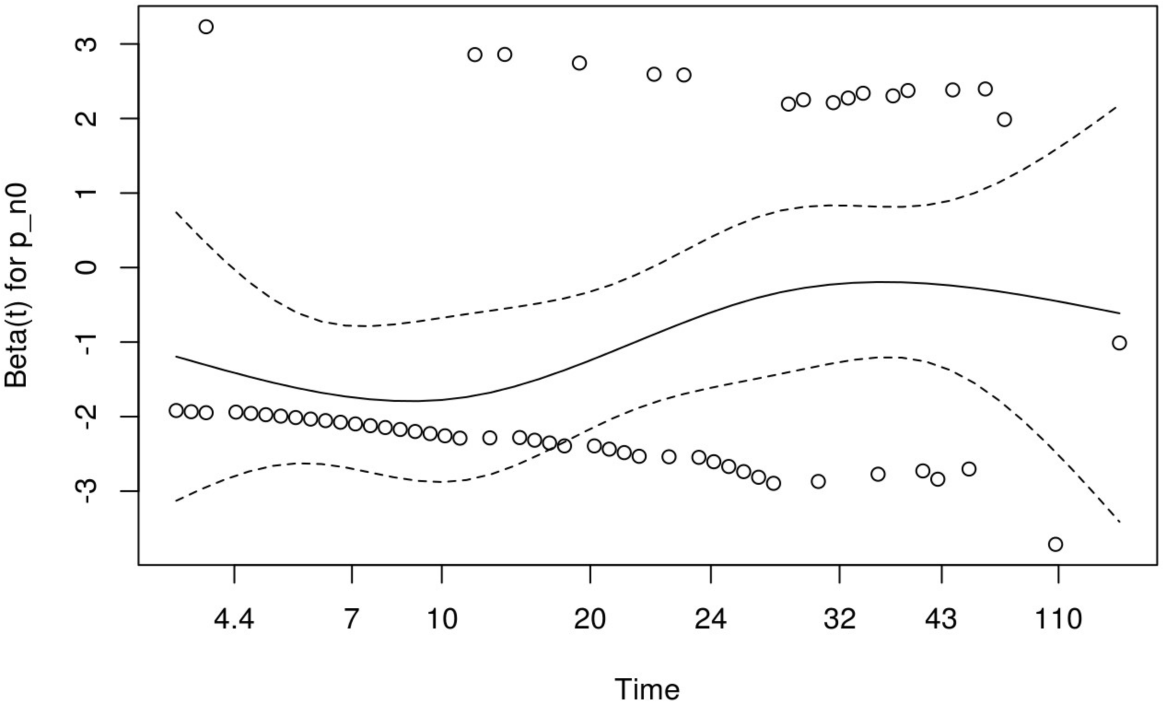

Immunohistochemical type

```
# Missing data check
table(is.na(resCompleteSurvData$cluster))
```

```
##
## FALSE  TRUE
##    79     7
```

```
#   -Resected patients, by cluster
cluster_res_PcbilSurvFit <- npsurv(formula = Surv(surv_time, death_observe
d) ~ cluster, data = resCompleteSurvData)
print(cluster_res_PcbilSurvFit, print.rmean = TRUE)
```

```
## Call: npsurv(formula = Surv(surv_time, death_observed) ~ cluster, data =
resCompleteSurvData)
##
##    7 observations deleted due to missingness
##                                           records n.max n.start events
## cluster=extrahepatic pancreatobiliary          57    57      57     49
## cluster=intestinal                              8     8       8      4
## cluster=intrahepatic cholangiocarcinoma        14    14      14      7
##                                           *rmean *se(rmean) median 0.95LCL
## cluster=extrahepatic pancreatobiliary       33.2       4.24   23.6    18.4
## cluster=intestinal                          71.9      14.82   54.0    31.0
## cluster=intrahepatic cholangiocarcinoma     76.0      11.54  109.4    32.5
##                                           0.95UCL
## cluster=extrahepatic pancreatobiliary        32.7
## cluster=intestinal                             NA
## cluster=intrahepatic cholangiocarcinoma        NA
##     * restricted mean with upper limit =  113
```

```
# KM
sampleSize <- sum(!is.na(resCompleteSurvData$cluster))
survplot(cluster_res_PcbilSurvFit, conf = "none", label.curves = FALSE, tim
e.inc = 10, n.risk = TRUE,
         col = c("red", "blueviolet", "blue", "green"), lty = 1, lwd = 3, ce
x.n.risk = 0.8,
         xlab="", ylab = "Survival probability", y.n.risk = -0.4, adj.n.ris
k = 0)
mtext("Months", side=1, line=2)
title(main=paste("Overall survival by immunohistochemical type\n(n = ", samp
leSize,")", sep = ""))
```

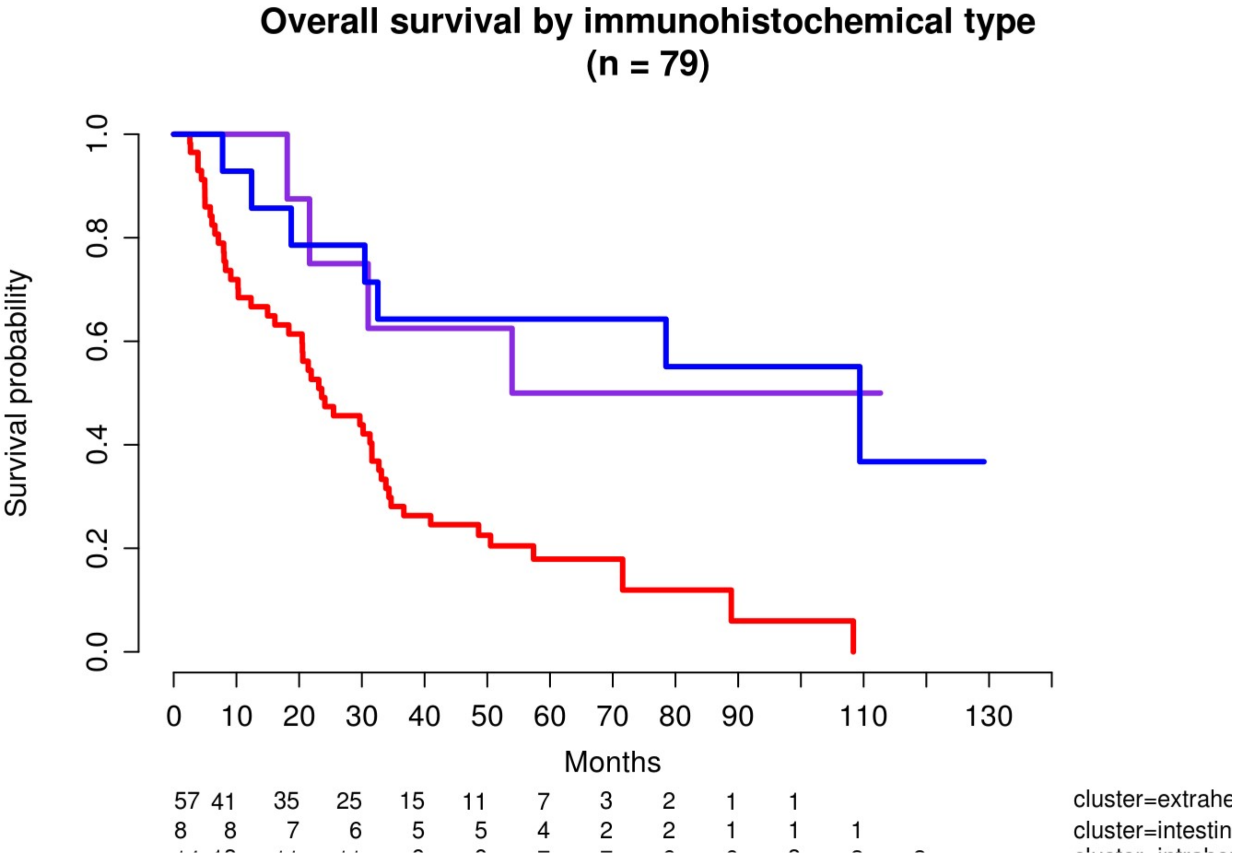


```
# Univariate Cox proportional hazards regression model.
coxClusterUniv <- coxph(Surv(surv_time, death_observed) ~ cluster, data = re
sCompleteSurvData)
summary(coxClusterUniv)
```

```
## Call:
## coxph(formula = Surv(surv_time, death_observed) ~ cluster, data = resComp
leteSurvData)
##
##   n= 79, number of events= 60
##    (7 observations deleted due to missingness)
##
##                                              coef exp(coef) se(coef)      z
## clusterintestinal                         -1.1754    0.3087   0.5296 -2.220
## clusterintrahepatic cholangiocarcinoma -1.3495    0.2594   0.4335 -3.113
##                                          Pr(>|z|)
## clusterintestinal                         0.02644 *
## clusterintrahepatic cholangiocarcinoma  0.00185 **
## ---
## Signif. codes:  0 '***' 0.001 '**' 0.01 '*' 0.05 '.' 0.1 ' ' 1
##
##                                          exp(coef) exp(-coef) lower .95
## clusterintestinal                            0.3087      3.240    0.1093
## clusterintrahepatic cholangiocarcinoma    0.2594      3.856    0.1109
##                                          upper .95
## clusterintestinal                            0.8715
## clusterintrahepatic cholangiocarcinoma    0.6066
##
## Concordance= 0.607  (se = 0.034 )
## Rsquare= 0.185   (max possible= 0.997 )
## Likelihood ratio test= 16.16  on 2 df,   p=0.0003099
## Wald test            = 12.79  on 2 df,   p=0.001673
## Score (logrank) test = 14.27  on 2 df,   p=0.0007961
```

```
# Test the Proportional Hazards Assumption of a Cox Regression
cox_pha <- cox.zph(coxClusterUniv)
print(cox_pha)
```

```
##                                              rho  chisq     p
## clusterintestinal                         -0.0142 0.0126 0.911
## clusterintrahepatic cholangiocarcinoma -0.0755 0.3474 0.556
## GLOBAL                                        NA 0.3479 0.840
```

```
plot(cox_pha)
```

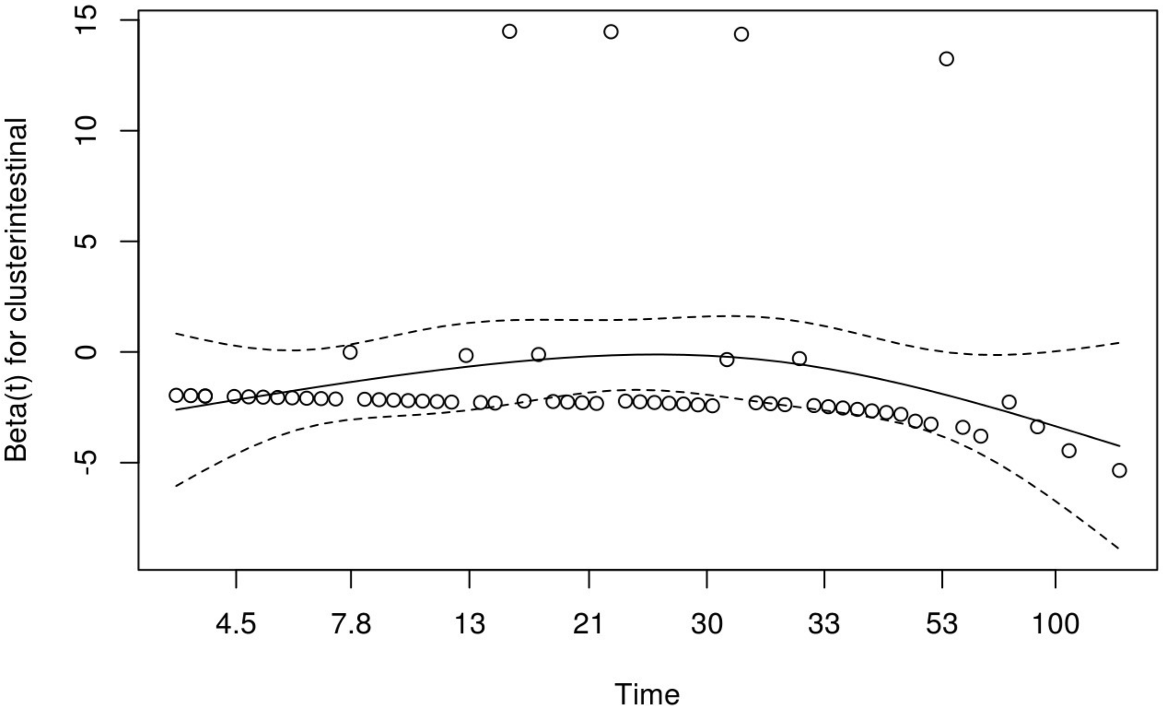

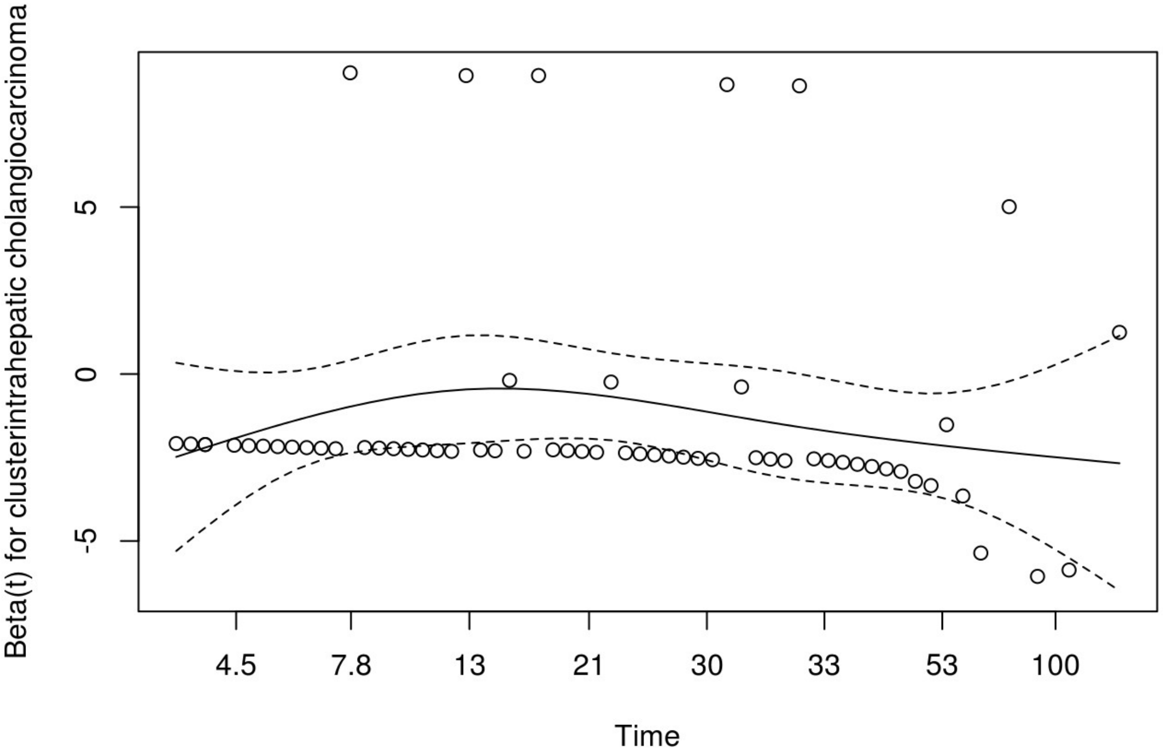


```
# Multivariate Cox proportional hazards regression model.
coxClusterMultiv <- coxph(Surv(surv_time, death_observed) ~ cluster + p_t +
p_n, data = resCompleteSurvData)
```

```
## Warning in fitter(X, Y, strats, offset, init, control, weights = weights,
:
## Loglik converged before variable 6 ; beta may be infinite.
```

```
summary(coxClusterMultiv)
```

```
## Call:
## coxph(formula = Surv(surv_time, death_observed) ~ cluster + p_t +
##     p_n, data = resCompleteSurvData)
##
##   n= 70, number of events= 55
##    (16 observations deleted due to missingness)
##
##                                            coef  exp(coef)   se(coef)
## clusterintestinal                    -1.662e+00  1.897e-01  6.787e-01
## clusterintrahepatic cholangiocarcinoma -4.988e-01  6.072e-01  5.231e-01
## p_t1                                  4.299e-01  1.537e+00  6.115e-01
## p_t2                                  2.360e-03  1.002e+00  3.289e-01
## p_t4                                  1.608e+00  4.995e+00  5.222e-01
## p_tis                                -1.689e+01  4.608e-08  4.652e+03
## p_n0                                 -7.536e-01  4.707e-01  3.753e-01
##                                           z Pr(>|z|)
## clusterintestinal                    -2.449  0.01434 *
## clusterintrahepatic cholangiocarcinoma -0.953  0.34034
## p_t1                                  0.703  0.48207
## p_t2                                  0.007  0.99427
## p_t4                                  3.080  0.00207 **
## p_tis                                -0.004  0.99710
## p_n0                                 -2.008  0.04467 *
## ---
## Signif. codes:  0 '***' 0.001 '**' 0.01 '*' 0.05 '.' 0.1 ' ' 1
##
##                                      exp(coef) exp(-coef) lower .95
## clusterintestinal                    1.897e-01  5.270e+00   0.05017
## clusterintrahepatic cholangiocarcinoma 6.072e-01  1.647e+00   0.21780
## p_t1                                 1.537e+00  6.506e-01   0.46365
## p_t2                                 1.002e+00  9.976e-01   0.52608
## p_t4                                 4.995e+00  2.002e-01   1.79505
## p_tis                                4.608e-08  2.170e+07   0.00000
## p_n0                                 4.707e-01  2.125e+00   0.22556
##                                      upper .95
## clusterintestinal                       0.7177
## clusterintrahepatic cholangiocarcinoma    1.6930
## p_t1                                    5.0954
## p_t2                                    1.9098
## p_t4                                   13.9003
## p_tis                                      Inf
## p_n0                                    0.9822
##
## Concordance= 0.689  (se = 0.042 )
## Rsquare= 0.299   (max possible= 0.997 )
## Likelihood ratio test= 24.9  on 7 df,   p=0.000792
## Wald test            = 20.41  on 7 df,   p=0.00474
## Score (logrank) test = 24  on 7 df,   p=0.001141
```

```
# Test the Proportional Hazards Assumption of a Cox Regression
cox_pha <- cox.zph(coxClusterMultiv)
print(cox_pha)
```

```
##                                               rho    chisq      p
## clusterintestinal                          0.0152 2.00e-02 0.8875
## clusterintrahepatic cholangiocarcinoma -0.1989 2.66e+00 0.1028
## p_t1                                       0.0651 2.24e-01 0.6359
## p_t2                                      -0.2231 2.64e+00 0.1043
## p_t4                                      -0.1254 1.19e+00 0.2753
## p_tis                                     -0.4390 6.24e-08 0.9998
## p_n0                                       0.2282 2.85e+00 0.0915
## GLOBAL                                         NA 9.26e+00 0.2345
```

```
plot(cox_pha)
```

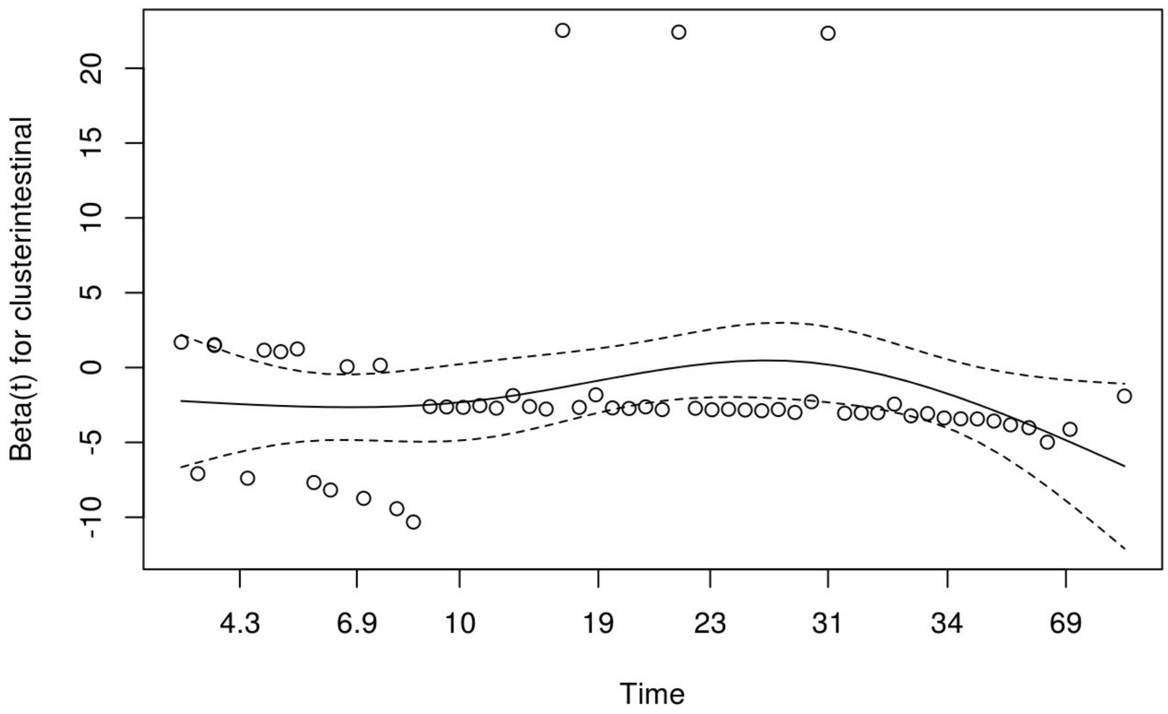

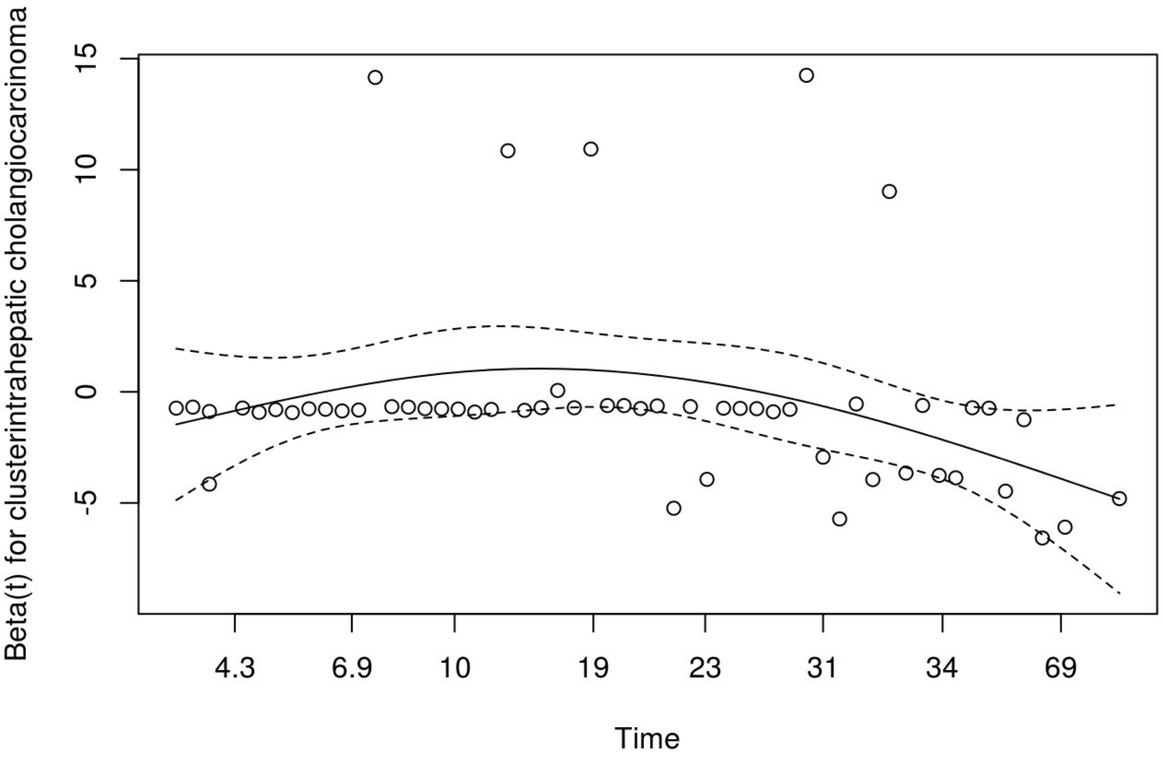
Beta(t) for clusterintrahepatic cholangiocarcinoma
15
10
5
0
-5
4.3
6.9
10
19
23
31
34
69
Time

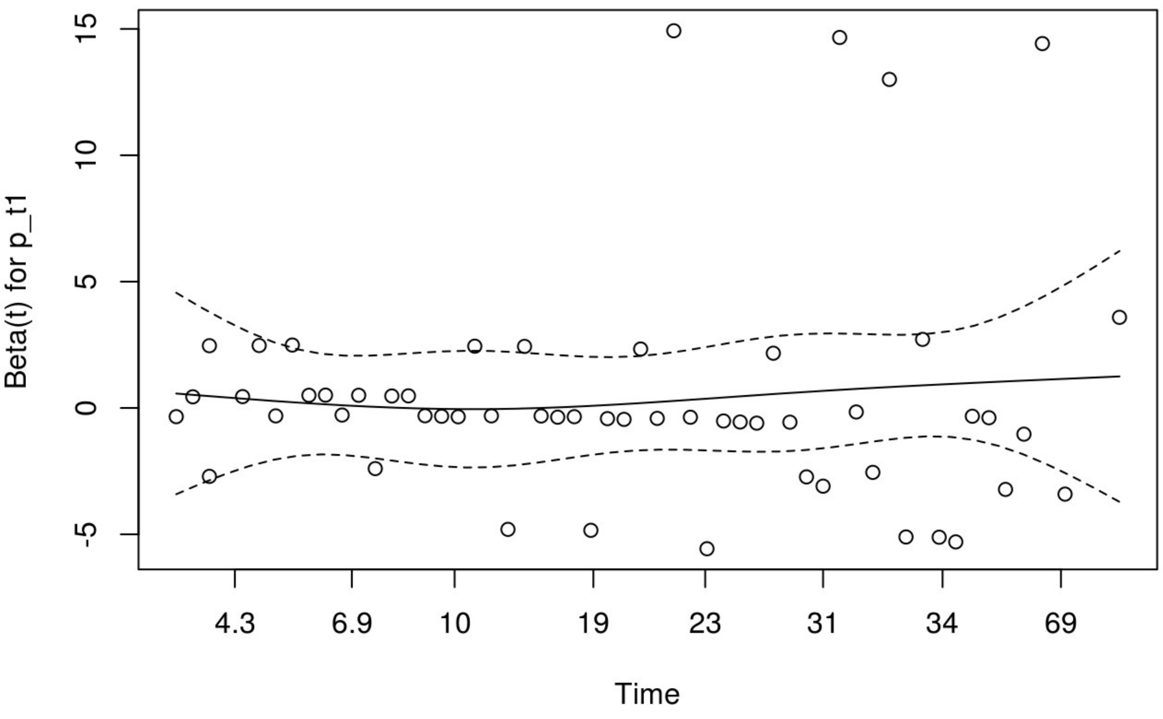
Beta(t) for p_t1
15
10
5
0
-5
4.3
6.9
10
19
23
31
34
69
Time

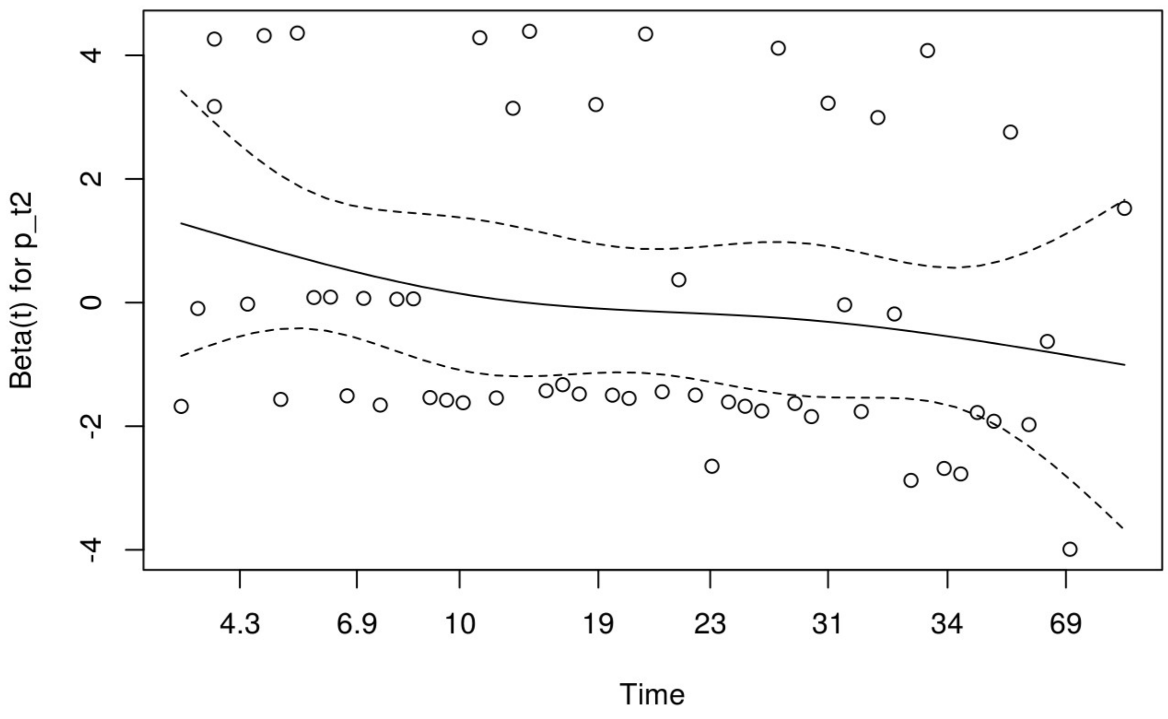
Beta(t) for p_t2
Time
4.3
6.9
10
19
23
31
34
69
4
2
0
-2
-4

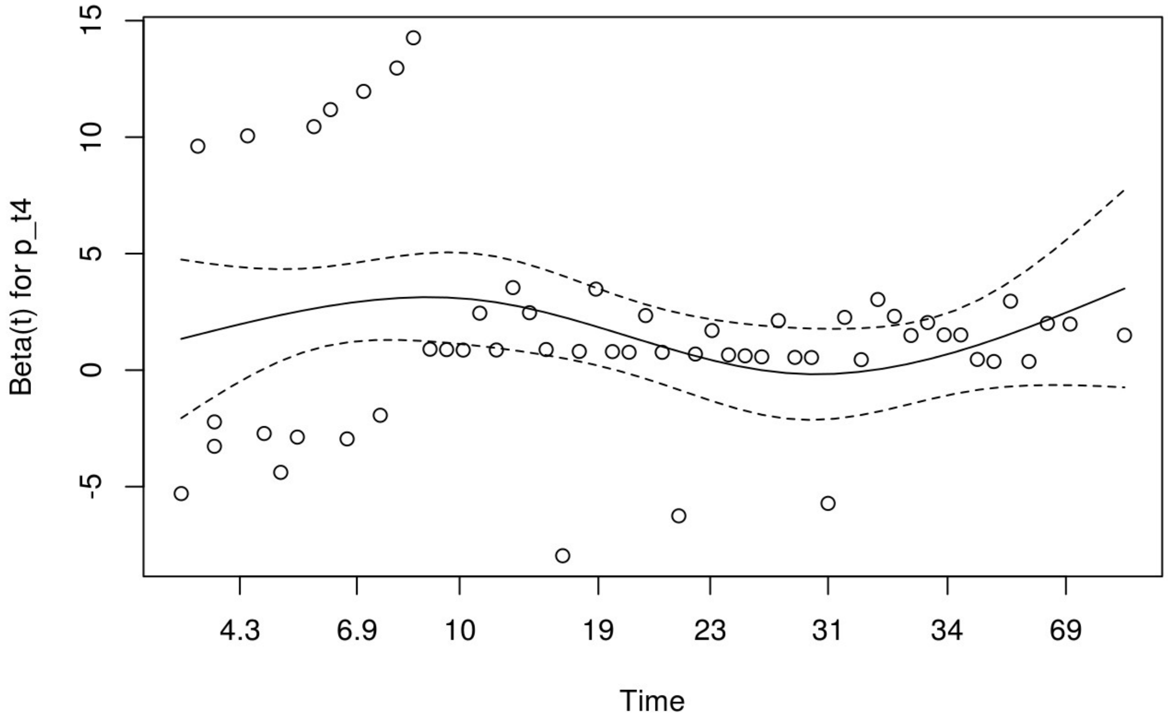
Beta(t) for p_t4
Time
4.3
6.9
10
19
23
31
34
69
15
10
5
0
-5

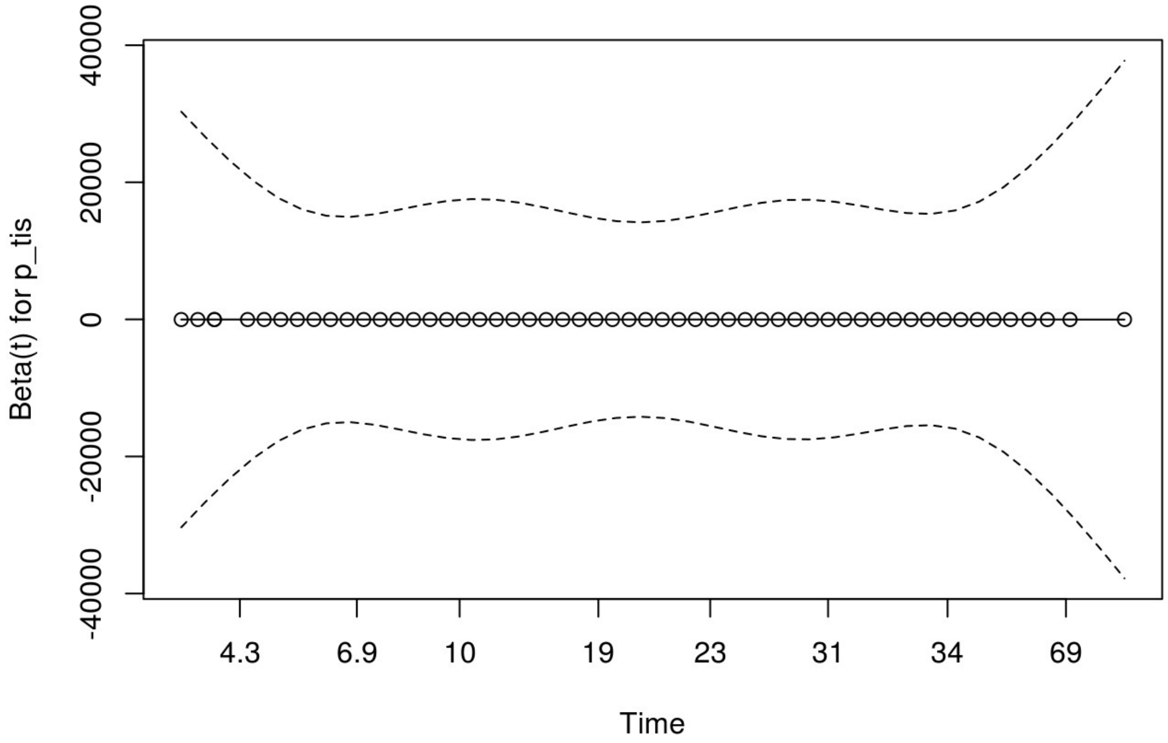

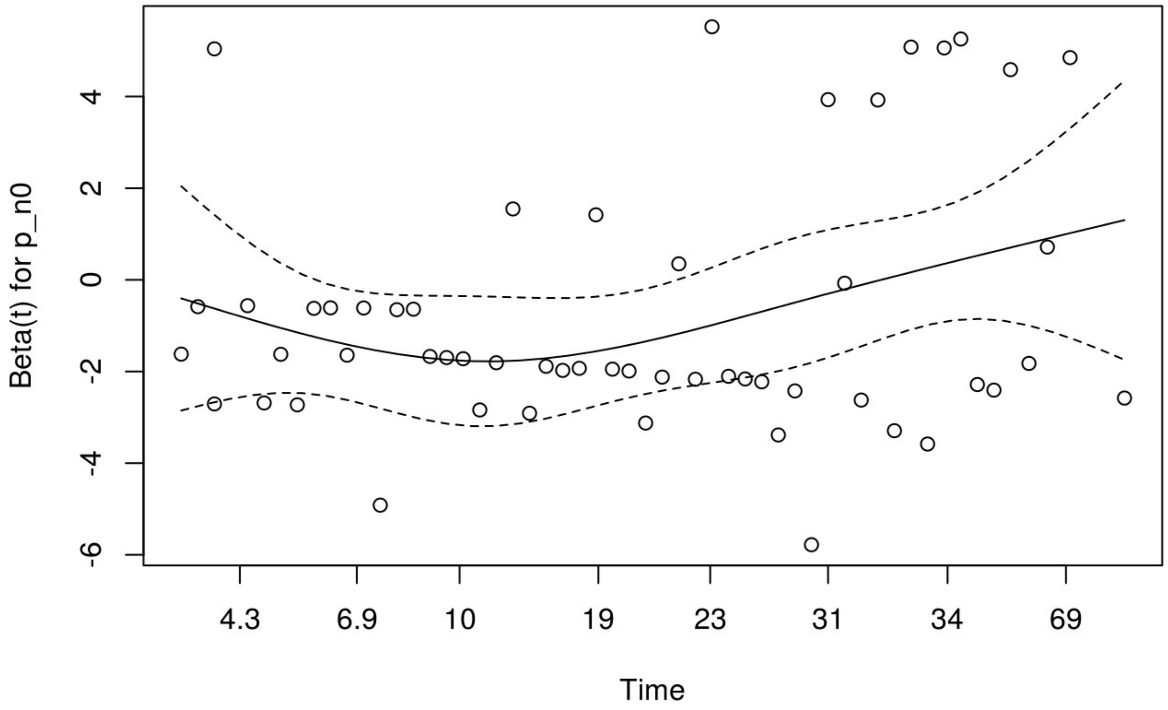


```
# Adjusted Akaike information criterion, Bayesian information criteria. The
smaller the AIC or BIC, the better the fit.
round(AICc(coxClusterMultiv, return.K = FALSE, second.ord = TRUE))
```

```
## [1] 389
```

```
round(BIC(coxClusterMultiv))
```

```
## [1] 403
```

Survival table data - univariate: immunohistochemical type (cluster)

```
rowsOfInterest <- c( paste("cluster", levels(resCompleteSurvData$cluster)
[2 : length(levels(resCompleteSurvData$cluster))], sep=""))
myXtable <- xtable(getCoxTable(coxClusterUniv, rowsOfInterest), caption = 'S
urvival table data - univariate: immunohistochemical type (cluster)')
digits(myXtable) <- c(0,2,2,2,3)
print(myXtable, type = "html", caption.placement = 'top')
```

Survival table data - univariate: immunohistochemical type (cluster)

	HR	LCI	UCI	p
clusterintestinal	0.31	0.11	0.87	0.026
clusterintrahepatic cholangiocarcinoma	0.26	0.11	0.61	0.002

Survival table data - multivariate: immunohistochemical type (cluster)

```
myXtable <- xtable(getCoxTable(coxClusterMultiv, rowsOfInterest), caption =
'Survival table data - multivariate: immunohistochemical type (cluster)')
digits(myXtable) <- c(0,2,2,2,3)
print(myXtable, type = "html", caption.placement = 'top')
```

Survival table data - multivariate: immunohistochemical type (cluster)

	HR	LCI	UCI	p
clusterintestinal	0.19	0.05	0.72	0.014
clusterintrahepatic cholangiocarcinoma	0.61	0.22	1.69	0.340

Anatomically-based diagnosis

```
# Missing data check
table(is.na(resCompleteSurvData$clin_diag))
```

```
##
## FALSE
##    86
```

```
#   -Resected patients, by clin_diag
clin_res_PcbilSurvFit <- npsurv(formula = Surv(surv_time, death_observed) ~
clin_diag, data = resCompleteSurvData)
print(clin_res_PcbilSurvFit, print.rmean = TRUE)
```

```
## Call: npsurv(formula = Surv(surv_time, death_observed) ~ clin_diag,
##     data = resCompleteSurvData)
##
##                                            records n.max n.start events
## clin_diag=Ductal pancreatic adenocarcinoma      30    30      30     25
## clin_diag=Ampullary carcinoma                   12    12      12     10
## clin_diag=Distal bile duct cancer                6     6       6      4
## clin_diag=Gallbladder cancer                    11    11      11      9
## clin_diag=Perihilar cholangiocarcinoma           9     9       9      4
## clin_diag=Intrahepatic cholangiocarcinoma       18    18      18     11
##                                            *rmean *se(rmean) median
## clin_diag=Ductal pancreatic adenocarcinoma   33.8       6.74   19.4
## clin_diag=Ampullary carcinoma                46.0      11.26   31.4
## clin_diag=Distal bile duct cancer            57.0      13.09   59.7
## clin_diag=Gallbladder cancer                 36.8      10.32   25.5
## clin_diag=Perihilar cholangiocarcinoma       77.3      12.39     NA
## clin_diag=Intrahepatic cholangiocarcinoma    59.0      11.05   55.8
##                                            0.95LCL 0.95UCL
## clin_diag=Ductal pancreatic adenocarcinoma    10.2      41
## clin_diag=Ampullary carcinoma                 21.7      NA
## clin_diag=Distal bile duct cancer             23.1      NA
## clin_diag=Gallbladder cancer                  20.5      NA
## clin_diag=Perihilar cholangiocarcinoma        33.8      NA
## clin_diag=Intrahepatic cholangiocarcinoma     12.4      NA
##     * restricted mean with upper limit =  111
```

```
# KM
sampleSize <- sum(!is.na(resCompleteSurvData$clin_diag))
survplot(clin_res_PcbilSurvFit, conf = "none", label.curves = FALSE, time.in
c = 10, n.risk = TRUE,
         col = c("red", "black", "deeppink", "purple", "yellow", "blue", "gr
een"), lty = 1, lwd = 3, cex.n.risk = 0.8, y.n.risk = -0.6,
         xlab="", ylab = "Survival probability", adj.n.risk = 0)
mtext("Months", side=1, line=2)
title(main=paste("Overall survival by anatomical diagnosis\n(n = ", sampleSi
ze,")", sep = ""))
```

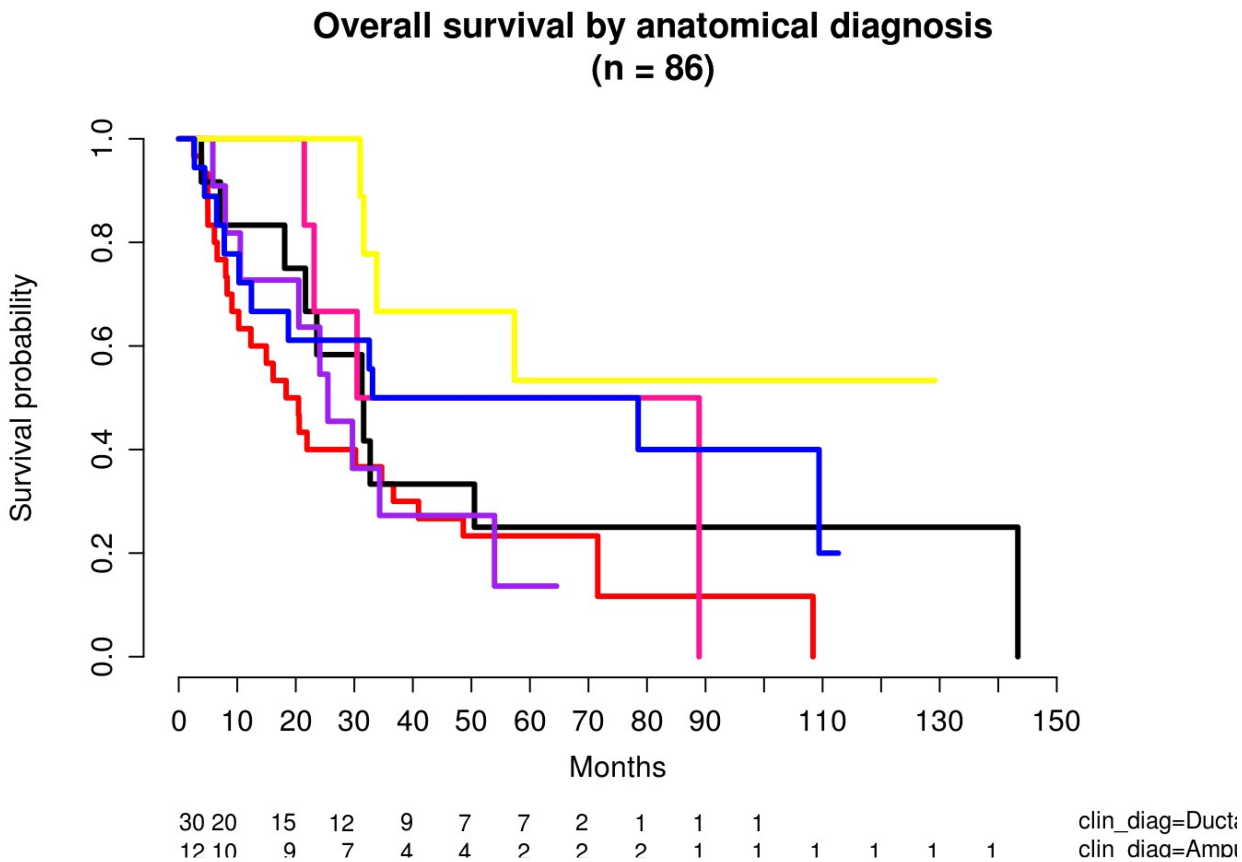


```
# Univariate:
coxClinDiagUniv <- coxph(Surv(surv_time, death_observed) ~ clin_diag, data
= resCompleteSurvData)
summary(coxClinDiagUniv)
```

```
## Call:
## coxph(formula = Surv(surv_time, death_observed) ~ clin_diag,
##     data = resCompleteSurvData)
##
##   n= 86, number of events= 63
##
##                                            coef exp(coef) se(coef)
## clin_diagAmpullary carcinoma            -0.41458   0.66062  0.39196
## clin_diagDistal bile duct cancer        -0.65762   0.51808  0.53993
## clin_diagGallbladder cancer             -0.09732   0.90727  0.39026
## clin_diagPerihilar cholangiocarcinoma   -1.40524   0.24531  0.54573
## clin_diagIntrahepatic cholangiocarcinoma -0.72080   0.48636  0.36897
##                                             z Pr(>|z|)
## clin_diagAmpullary carcinoma            -1.058   0.2902
## clin_diagDistal bile duct cancer        -1.218   0.2232
## clin_diagGallbladder cancer             -0.249   0.8031
## clin_diagPerihilar cholangiocarcinoma   -2.575   0.0100 *
## clin_diagIntrahepatic cholangiocarcinoma -1.954   0.0508 .
## ---
## Signif. codes:  0 '***' 0.001 '**' 0.01 '*' 0.05 '.' 0.1 ' ' 1
##
##                                         exp(coef) exp(-coef) lower .95
## clin_diagAmpullary carcinoma               0.6606      1.514   0.30642
## clin_diagDistal bile duct cancer           0.5181      1.930   0.17981
## clin_diagGallbladder cancer                0.9073      1.102   0.42223
## clin_diagPerihilar cholangiocarcinoma      0.2453      4.076   0.08418
## clin_diagIntrahepatic cholangiocarcinoma    0.4864      2.056   0.23599
##                                         upper .95
## clin_diagAmpullary carcinoma               1.4242
## clin_diagDistal bile duct cancer           1.4928
## clin_diagGallbladder cancer                1.9495
## clin_diagPerihilar cholangiocarcinoma      0.7149
## clin_diagIntrahepatic cholangiocarcinoma    1.0024
##
## Concordance= 0.616  (se = 0.039 )
## Rsquare= 0.119   (max possible= 0.996 )
## Likelihood ratio test= 10.94  on 5 df,   p=0.05249
## Wald test            = 9.46  on 5 df,   p=0.09216
## Score (logrank) test = 10.25  on 5 df,   p=0.06846
```

```
# Test the Proportional Hazards Assumption of a Cox Regression
cox_pha <- cox.zph(coxClinDiagUniv)
print(cox_pha)
```

```
##                                                rho chisq     p
## clin_diagAmpullary carcinoma              0.0415 0.110 0.740
## clin_diagDistal bile duct cancer          0.1641 1.604 0.205
## clin_diagGallbladder cancer               0.1402 1.212 0.271
## clin_diagPerihilar cholangiocarcinoma     0.1392 1.249 0.264
## clin_diagIntrahepatic cholangiocarcinoma -0.0904 0.508 0.476
## GLOBAL                                        NA 5.032 0.412
```

```
plot(cox_pha)
```

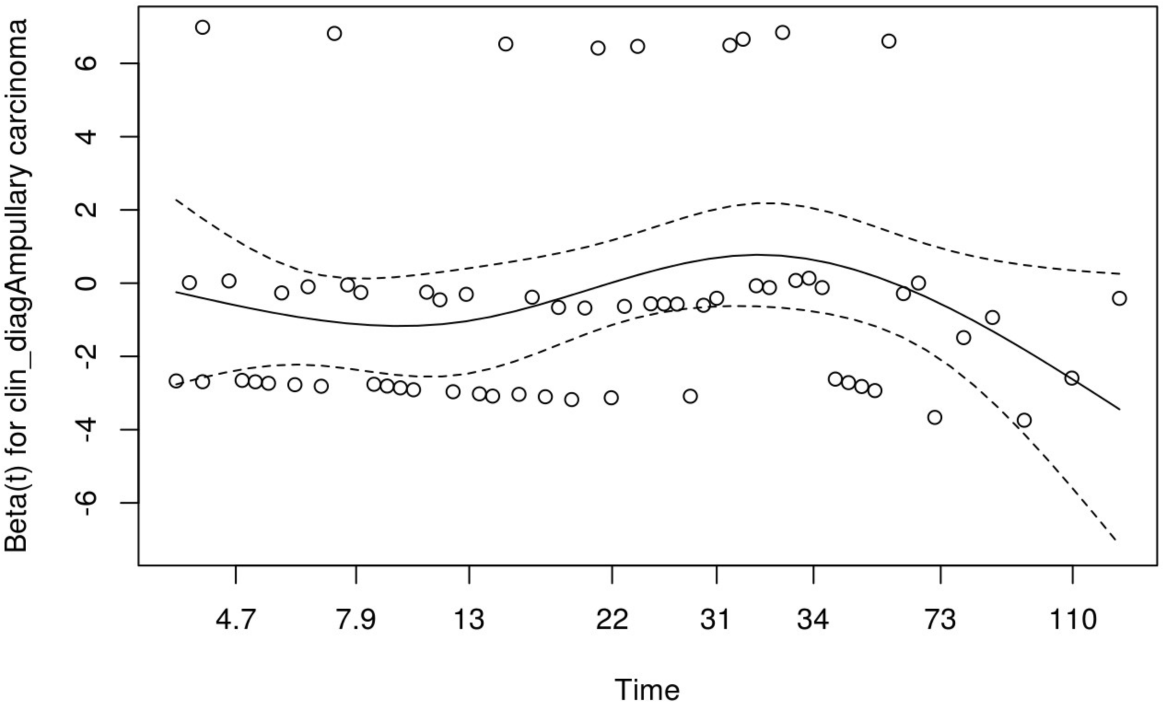

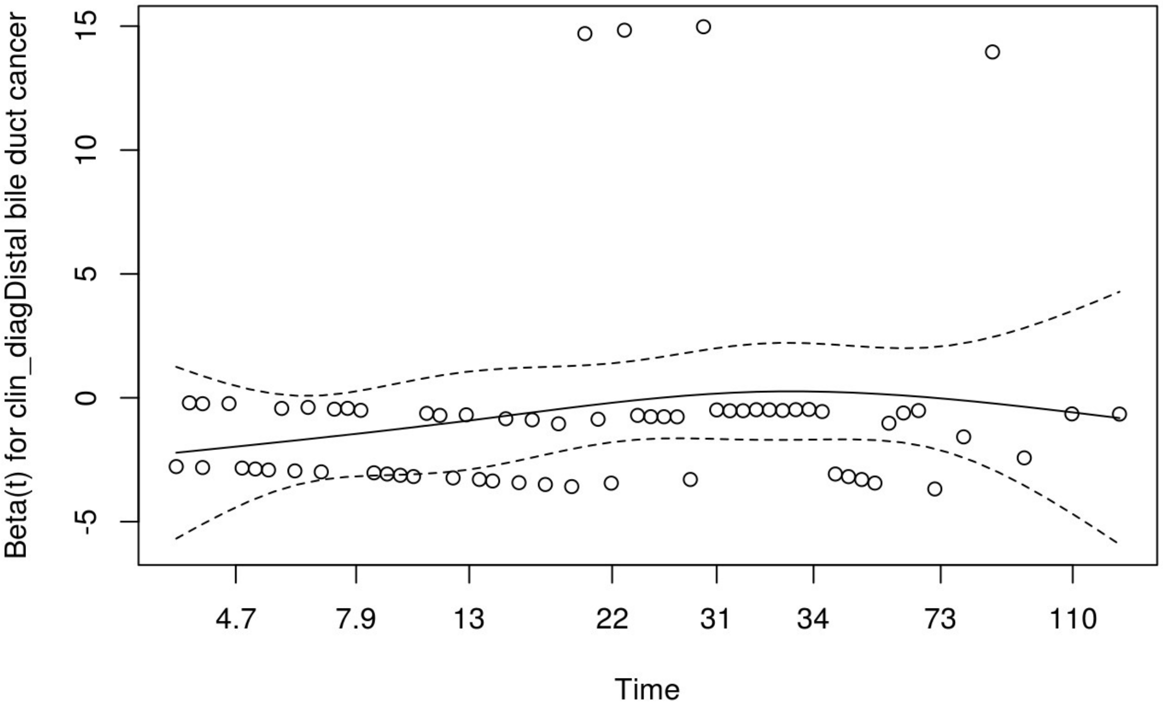

Beta(t) for clin_diagDistal bile duct cancer
15
10
5
0
-5
4.7
7.9
13
22
31
34
73
110
Time

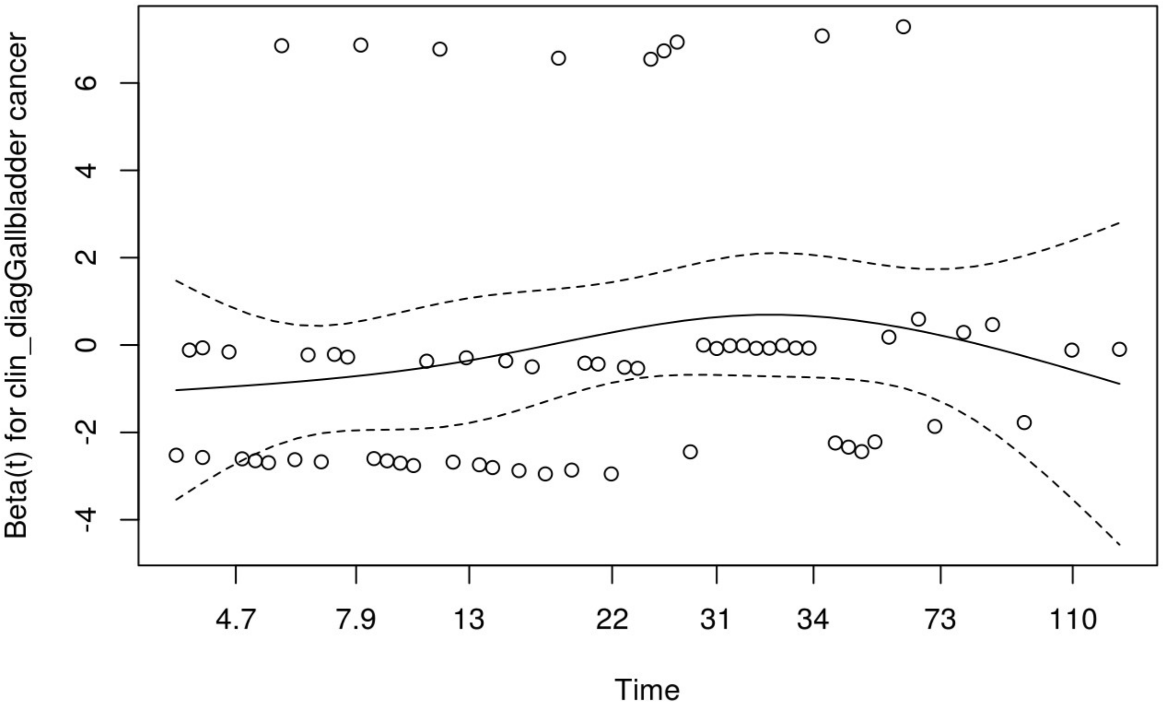

Beta(t) for clin_diagGallbladder cancer
6
4
2
0
-2
-4
4.7
7.9
13
22
31
34
73
110
Time

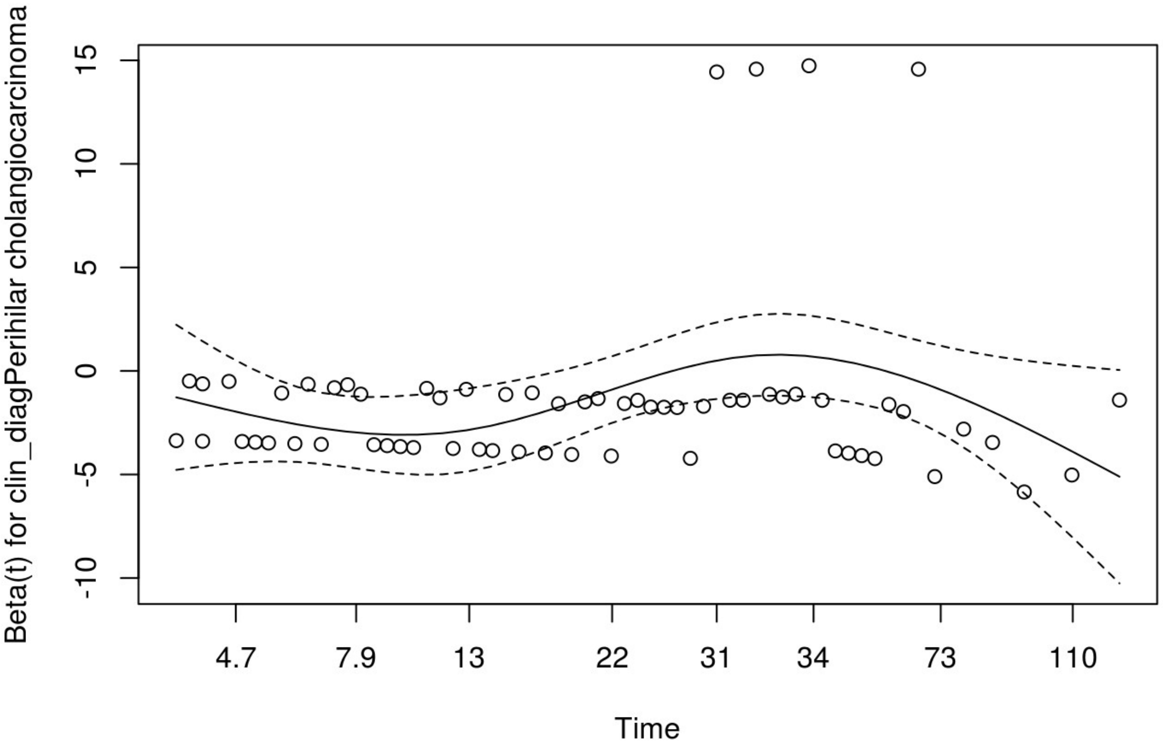

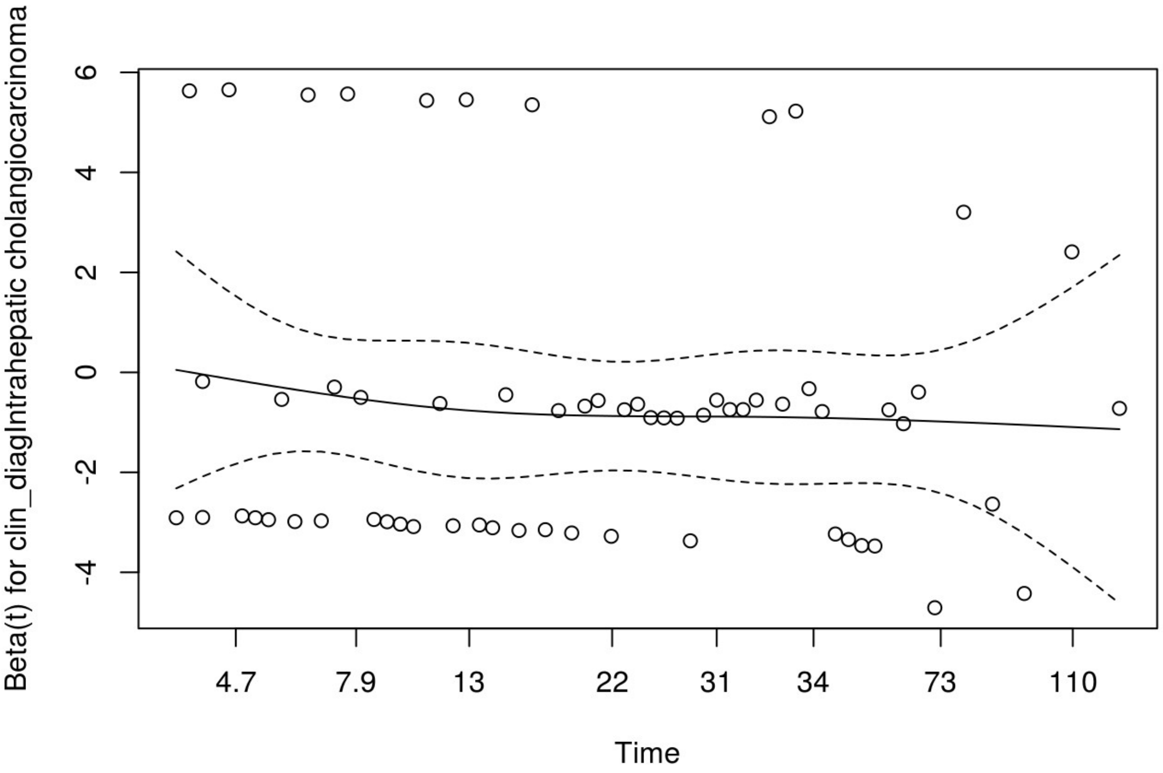


```
# Multivariate Cox proportional hazards regression model.
coxClinDIagMultiv <- coxph(Surv(surv_time, death_observed) ~ clin_diag + p_
t + p_n, data = resCompleteSurvData)
```

```
## Warning in fitter(X, Y, strats, offset, init, control, weights = weights,
:
## Loglik converged before variable 9 ; beta may be infinite.
```

```
summary(coxClinDIagMultiv)
```

```
## Call:
## coxph(formula = Surv(surv_time, death_observed) ~ clin_diag +
##     p_t + p_n, data = resCompleteSurvData)
##
##   n= 76, number of events= 58
##    (10 observations deleted due to missingness)
##
##                                              coef  exp(coef)   se(coef)
## clin_diagAmpullary carcinoma            -1.062e-01  8.993e-01  4.175e-01
## clin_diagDistal bile duct cancer        -3.861e-01  6.797e-01  6.233e-01
## clin_diagGallbladder cancer              3.349e-01  1.398e+00  4.408e-01
## clin_diagPerihilar cholangiocarcinoma   -5.372e-01  5.844e-01  5.822e-01
## clin_diagIntrahepatic cholangiocarcinoma  5.767e-01  1.780e+00  4.455e-01
## p_t1                                    -1.768e-02  9.825e-01  6.289e-01
## p_t2                                    -1.153e-01  8.911e-01  3.507e-01
## p_t4                                     8.956e-01  2.449e+00  4.239e-01
## p_tis                                   -1.614e+01  9.739e-08  3.088e+03
## p_n0                                    -8.557e-01  4.250e-01  3.675e-01
##                                              z Pr(>|z|)
## clin_diagAmpullary carcinoma            -0.254   0.7992
## clin_diagDistal bile duct cancer        -0.620   0.5356
## clin_diagGallbladder cancer              0.760   0.4474
## clin_diagPerihilar cholangiocarcinoma   -0.923   0.3561
## clin_diagIntrahepatic cholangiocarcinoma  1.295   0.1955
## p_t1                                    -0.028   0.9776
## p_t2                                    -0.329   0.7423
## p_t4                                     2.113   0.0346 *
## p_tis                                   -0.005   0.9958
## p_n0                                    -2.329   0.0199 *
## ---
## Signif. codes:  0 '***' 0.001 '**' 0.01 '*' 0.05 '.' 0.1 ' ' 1
##
##                                         exp(coef) exp(-coef) lower .95
## clin_diagAmpullary carcinoma            8.993e-01  1.112e+00    0.3968
## clin_diagDistal bile duct cancer        6.797e-01  1.471e+00    0.2003
## clin_diagGallbladder cancer             1.398e+00  7.154e-01    0.5892
## clin_diagPerihilar cholangiocarcinoma   5.844e-01  1.711e+00    0.1867
## clin_diagIntrahepatic cholangiocarcinoma 1.780e+00  5.618e-01    0.7435
## p_t1                                    9.825e-01  1.018e+00    0.2864
## p_t2                                    8.911e-01  1.122e+00    0.4481
## p_t4                                    2.449e+00  4.083e-01    1.0670
## p_tis                                   9.739e-08  1.027e+07    0.0000
## p_n0                                    4.250e-01  2.353e+00    0.2068
##                                         upper .95
## clin_diagAmpullary carcinoma               2.0381
## clin_diagDistal bile duct cancer           2.3059
## clin_diagGallbladder cancer                3.3165
## clin_diagPerihilar cholangiocarcinoma      1.8291
## clin_diagIntrahepatic cholangiocarcinoma    4.2623
## p_t1                                       3.3699
## p_t2                                       1.7718
## p_t4                                       5.6203
```

```
## p_tis                                          Inf
## p_n0                                        0.8733
##
## Concordance= 0.705  (se = 0.042 )
## Rsquare= 0.271   (max possible= 0.996 )
## Likelihood ratio test= 23.97  on 10 df,   p=0.007671
## Wald test            = 20.43  on 10 df,   p=0.02542
## Score (logrank) test = 25.14  on 10 df,   p=0.005084
```

```
# Test the Proportional Hazards Assumption of a Cox Regression
cox_pha <- cox.zph(coxClinDIagMultiv)
print(cox_pha)
```

```
##                                                 rho    chisq      p
## clin_diagAmpullary carcinoma             -0.0251 3.99e-02 0.8416
## clin_diagDistal bile duct cancer          0.0712 2.84e-01 0.5940
## clin_diagGallbladder cancer               0.1811 1.95e+00 0.1629
## clin_diagPerihilar cholangiocarcinoma     0.0947 4.89e-01 0.4845
## clin_diagIntrahepatic cholangiocarcinoma -0.1326 1.24e+00 0.2646
## p_t1                                      0.1180 8.35e-01 0.3609
## p_t2                                     -0.1171 8.33e-01 0.3614
## p_t4                                     -0.2832 4.72e+00 0.0298
## p_tis                                    -0.3621 2.06e-07 0.9996
## p_n0                                      0.1456 1.14e+00 0.2859
## GLOBAL                                        NA 1.43e+01 0.1596
```

```
plot(cox_pha)
```

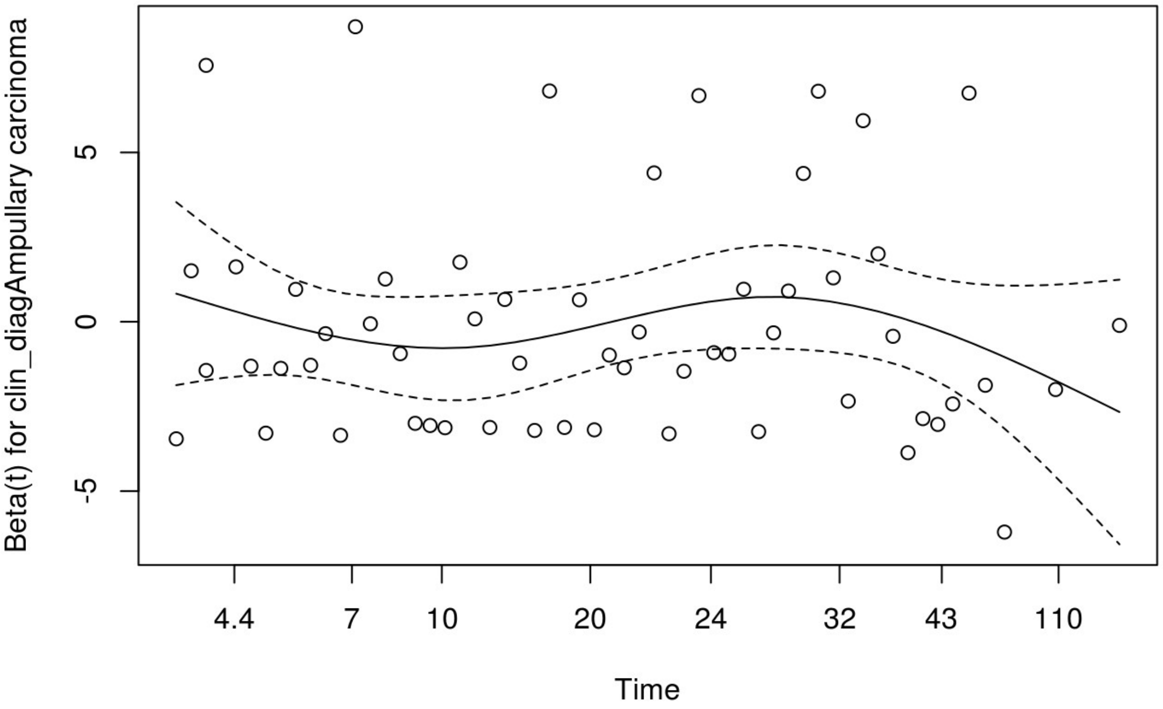

Beta(t) for clin_diagAmpullary carcinoma
5
0
-5
4.4
7
10
20
24
32
43
110
Time

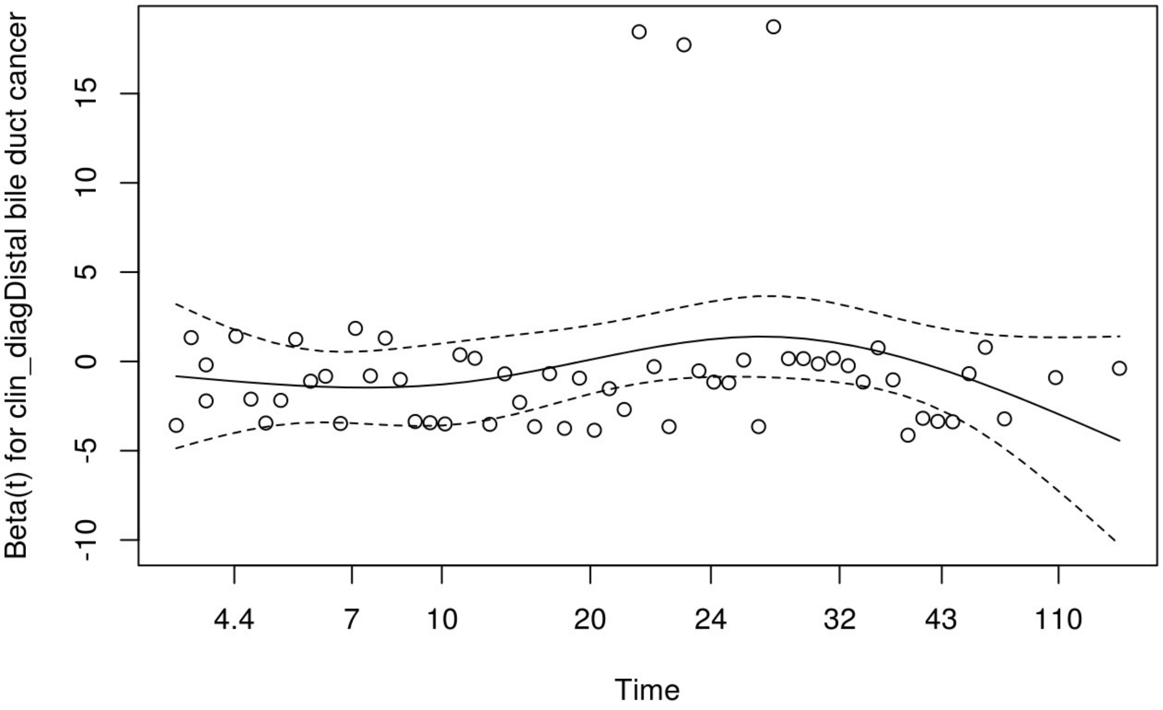

Beta(t) for clin_diagDistal bile duct cancer
15
10
5
0
-5
-10
4.4
7
10
20
24
32
43
110
Time

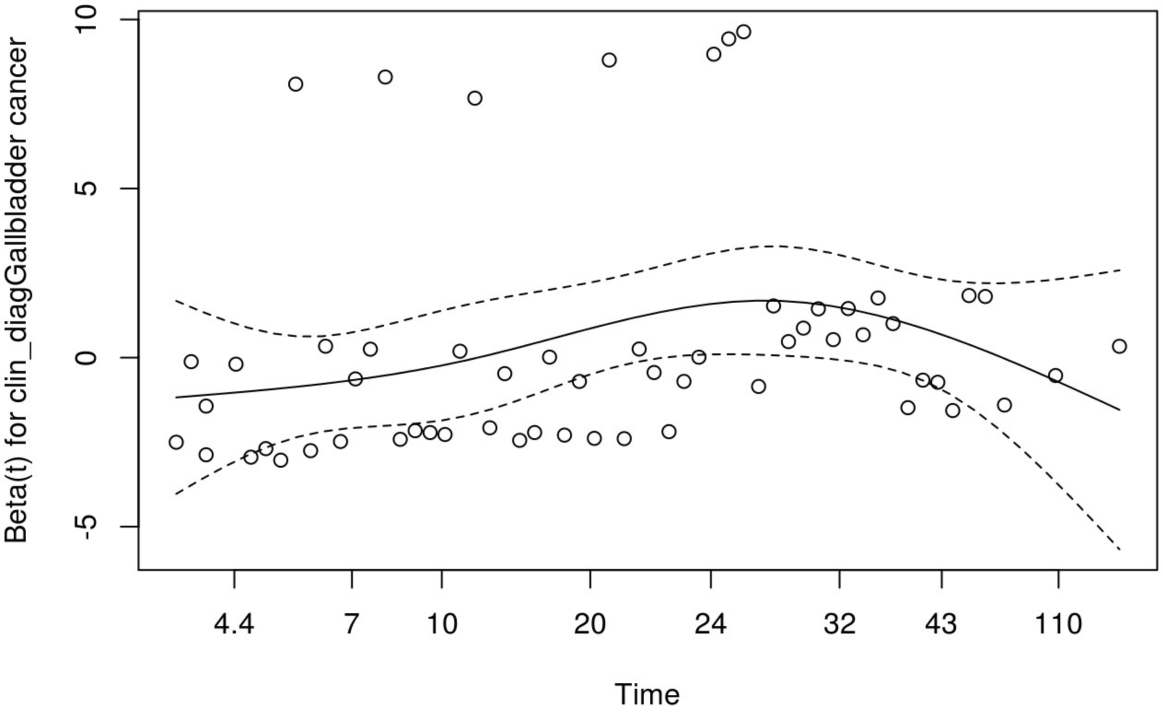

10
5
0
-5
Beta(t) for clin_diagGallbladder cancer
4.4
7
10
20
24
32
43
110
Time

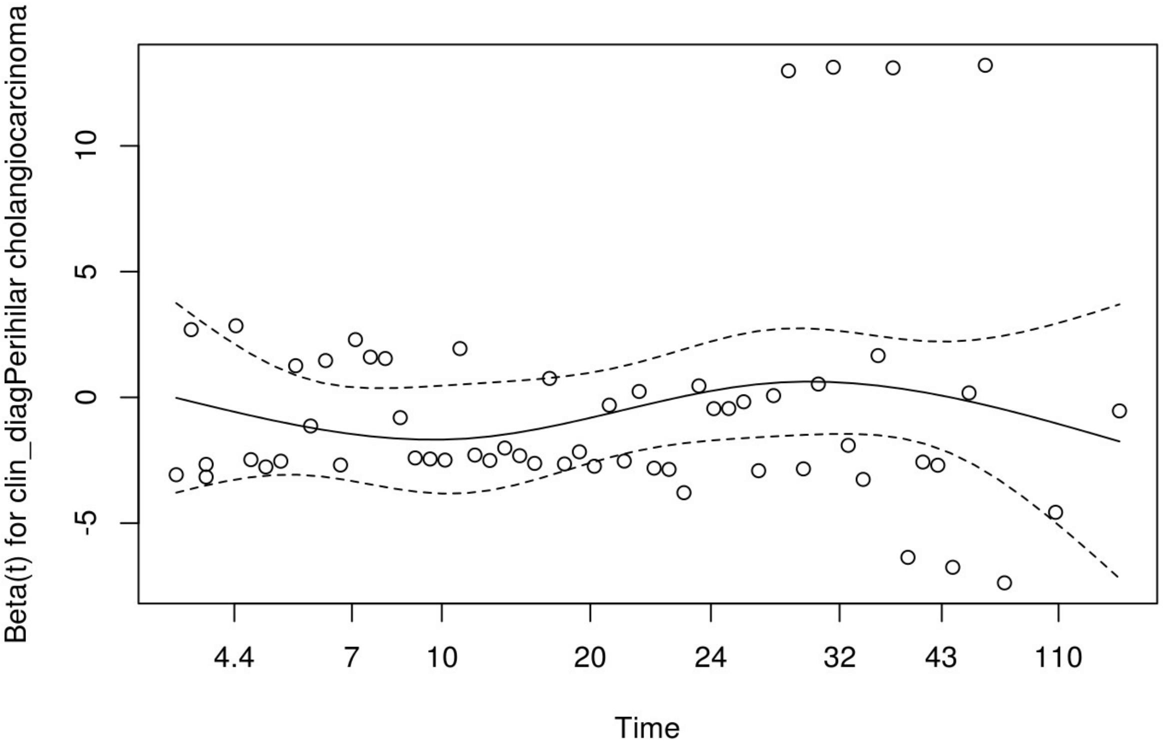

10
5
0
-5
Beta(t) for clin_diagPerihilar cholangiocarcinoma
4.4
7
10
20
24
32
43
110
Time

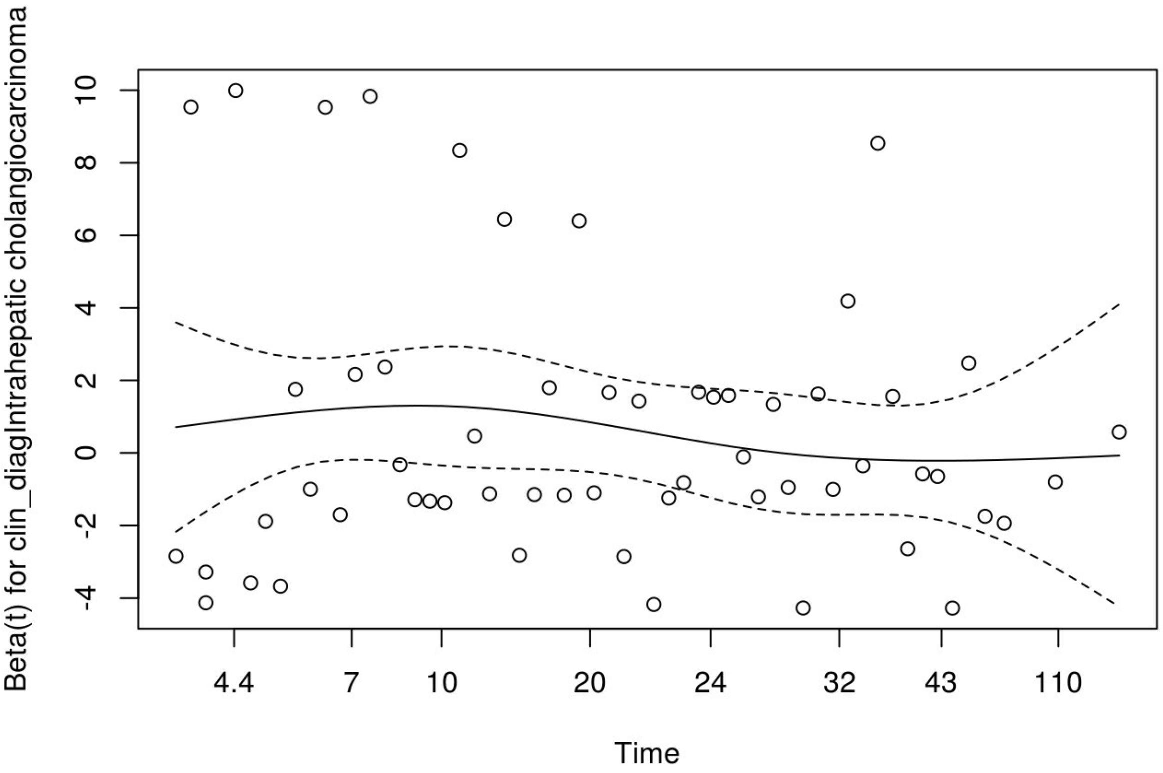
Beta(t) for clin_diagIntrahepatic cholangiocarcinoma
10
8
6
4
2
0
-2
-4
4.4
7
10
20
24
32
43
110
Time

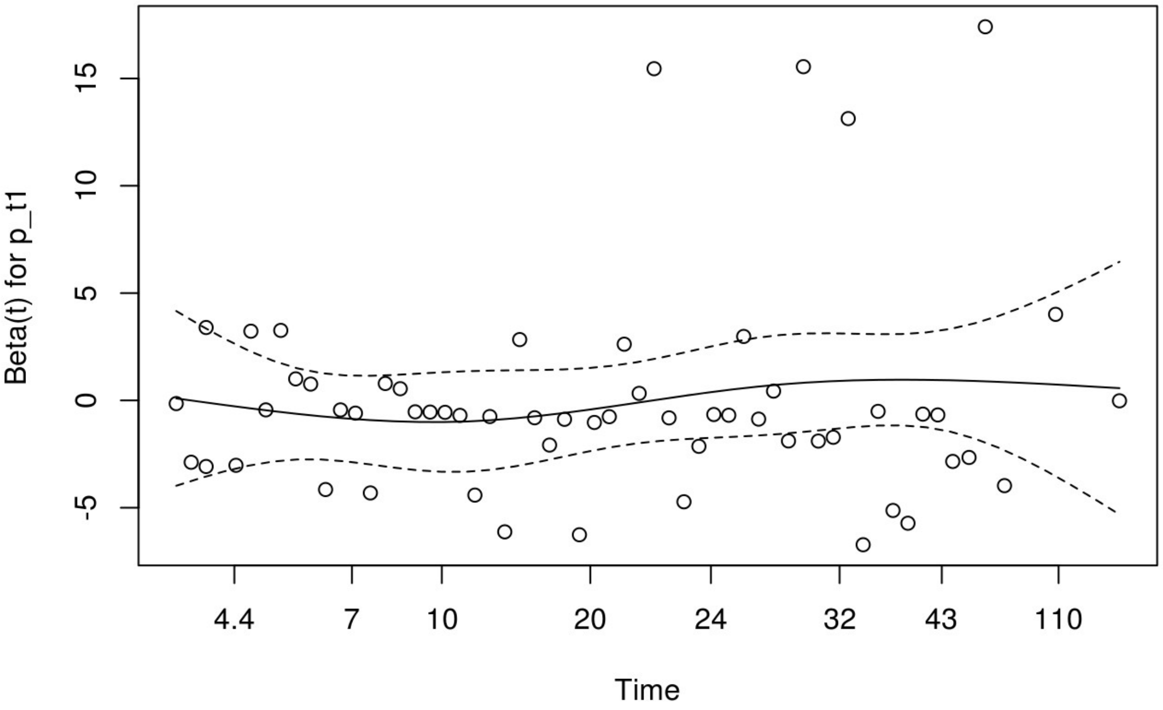
Beta(t) for p_t1
15
10
5
0
-5
4.4
7
10
20
24
32
43
110
Time

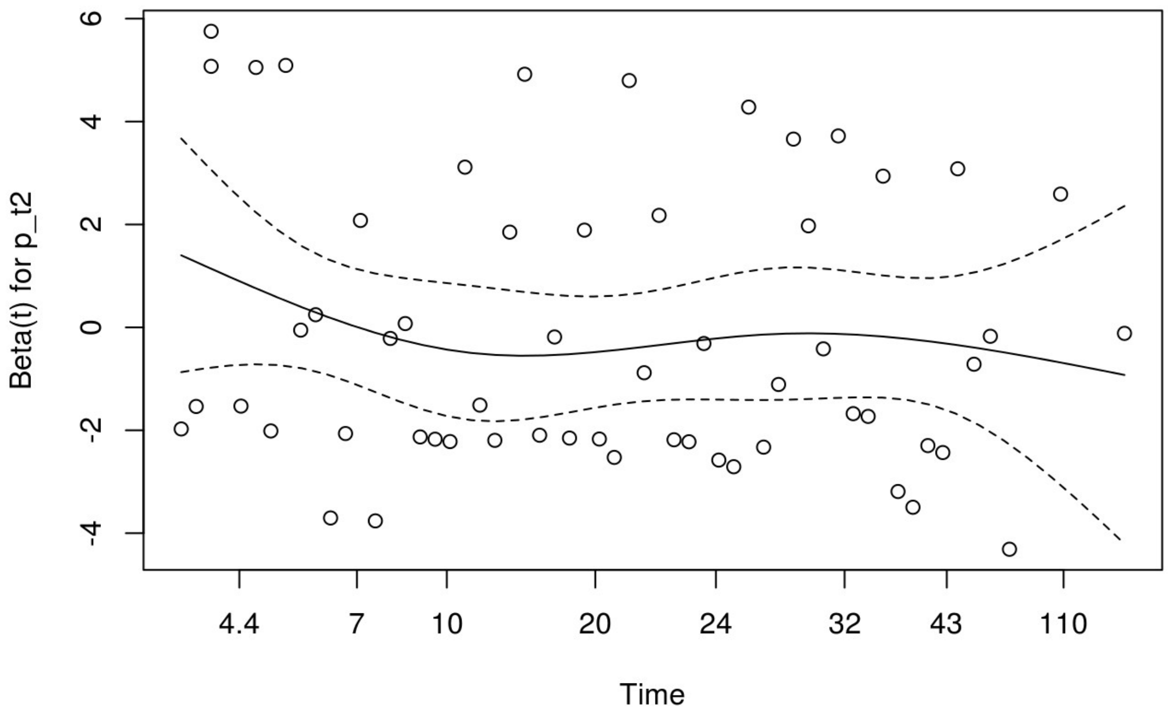

Beta(t) for p_t2
6
4
2
0
-2
-4
4.4
7
10
20
24
32
43
110
Time

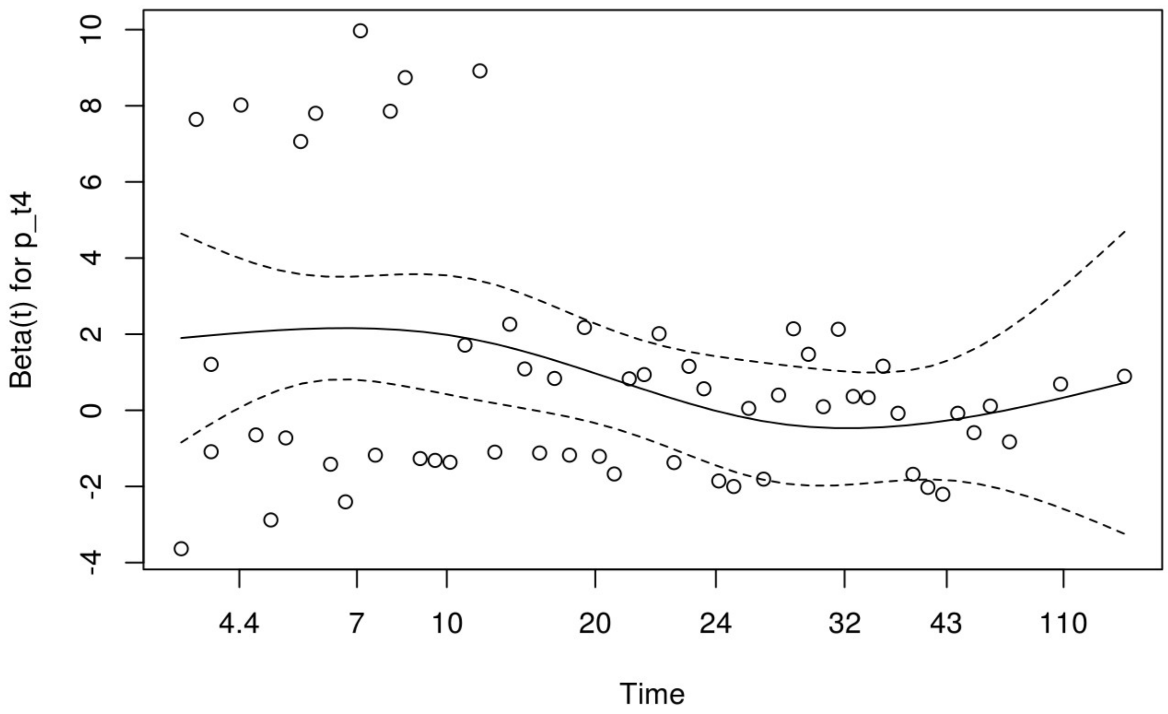

Beta(t) for p_t4
10
8
6
4
2
0
-2
-4
4.4
7
10
20
24
32
43
110
Time

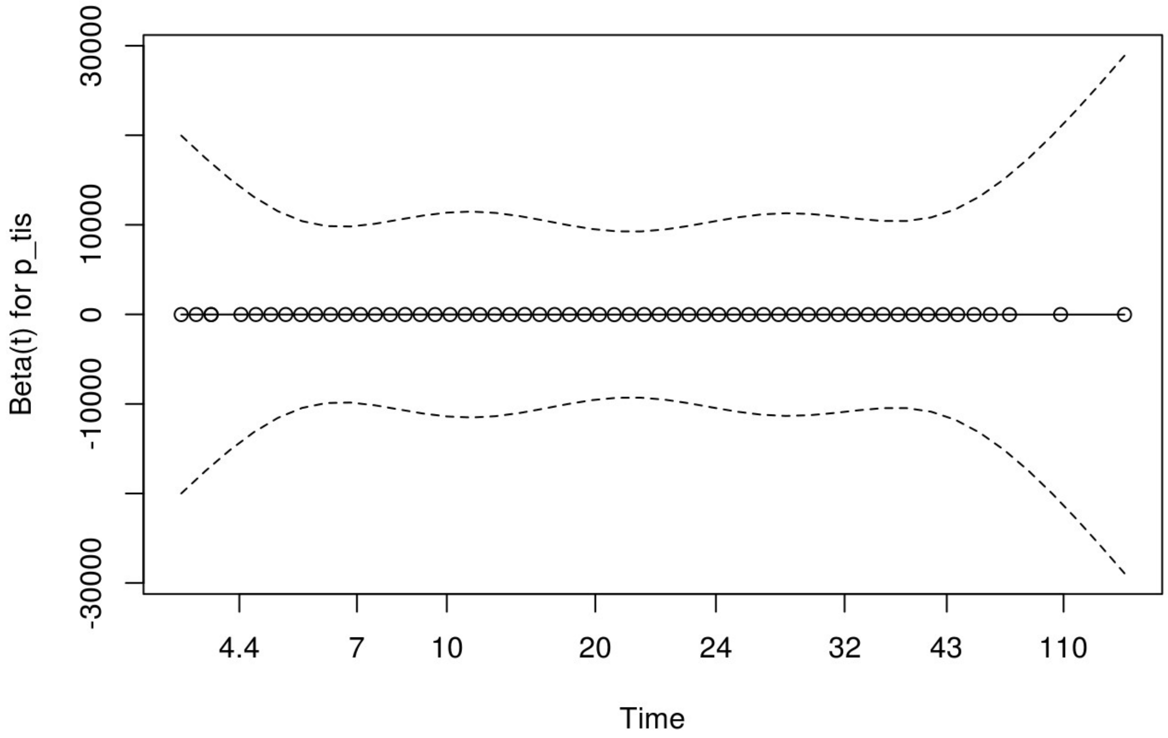

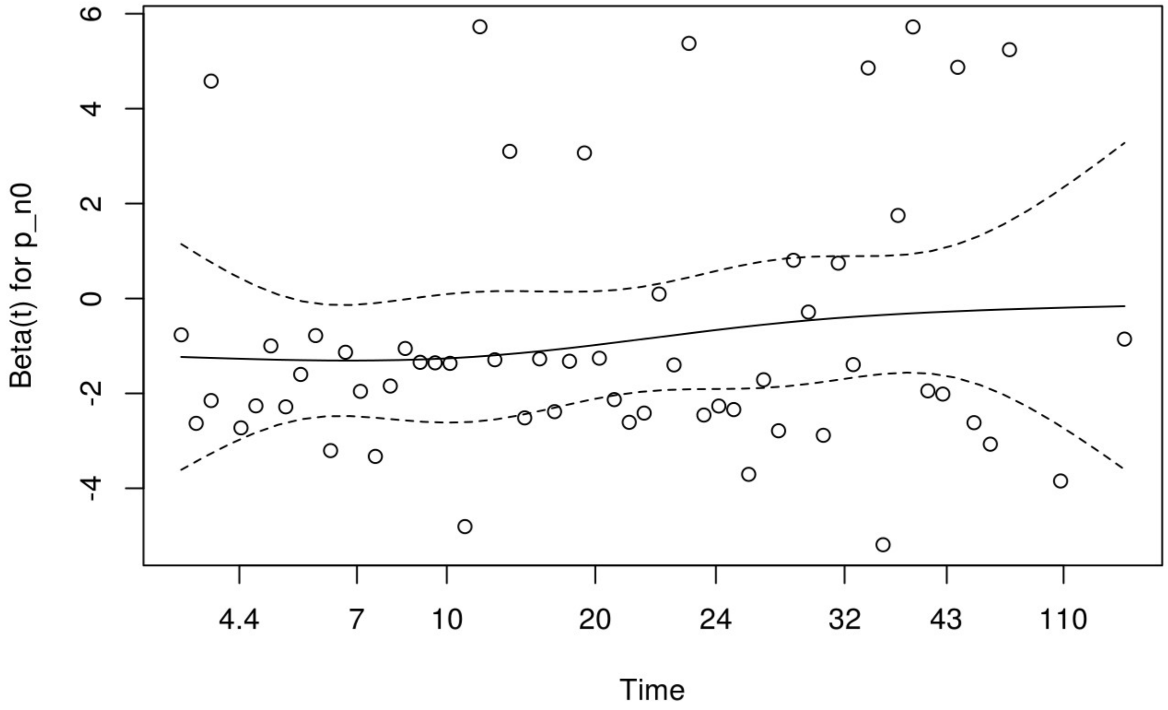


```
# Adjusted Akaike information criterion, Bayesian information criteria. The
smaller the AIC or BIC, the better the fit.
round(AICc(coxClinDIagMultiv, return.K = FALSE, second.ord = TRUE))
```

```
## [1] 427
```

```
round(BIC(coxClinDIagMultiv))
```

```
## [1] 447
```

Survival table data - univariate: anatomical-based diagnosis (clin_diag)

```
rowsOfInterest <- c( paste("clin_diag", levels(resCompleteSurvData$clin_dia
g)[2 : length(levels(resCompleteSurvData$clin_diag))], sep=""))
myXtable <- xtable(getCoxTable(coxClinDiagUniv, rowsOfInterest), caption =
'Survival table data - univariate: anatomical-based diagnosis (clin_diag)')
digits(myXtable) <- c(0,2,2,2,3)
print(myXtable, type = "html", caption.placement = 'top')
```

Survival table data - univariate: anatomical-based diagnosis (clin_diag)

	HR	LCI	UCI	p
clin_diagAmpullary carcinoma	0.66	0.31	1.42	0.290
clin_diagDistal bile duct cancer	0.52	0.18	1.49	0.223
clin_diagGallbladder cancer	0.91	0.42	1.95	0.803
clin_diagPerihilar cholangiocarcinoma	0.25	0.08	0.71	0.010
clin_diagIntrahepatic cholangiocarcinoma	0.49	0.24	1.00	0.051

Survival table data - multivariate: anatomical-based diagnosis (clin_diag)

```
myXtable <- xtable(getCoxTable(coxClinDIagMultiv, rowsOfInterest), caption
= 'Survival table data - multivariate: anatomical-based diagnosis (clin_dia
g)')
digits(myXtable) <- c(0,2,2,2,3)
print(myXtable, type = "html", caption.placement = 'top')
```

Survival table data - multivariate: anatomical-based diagnosis (clin_diag)

	HR	LCI	UCI	p
clin_diagAmpullary carcinoma	0.90	0.40	2.04	0.799
clin_diagDistal bile duct cancer	0.68	0.20	2.31	0.536
clin_diagGallbladder cancer	1.40	0.59	3.32	0.447
clin_diagPerihilar cholangiocarcinoma	0.58	0.19	1.83	0.356
clin_diagIntrahepatic cholangiocarcinoma	1.78	0.74	4.26	0.195

Resected patients: extrahepatic pancreatobiliary vs intestinal immunohistochemical types

```
# Obtain and attach data set
int_ehpcbiltucResCompleteSurvData <- subset(resCompleteSurvData, (cluster =
= "extrahepatic pancreatobiliary" | cluster == "intestinal"))

# Missing data check
table(is.na(int_ehpcbiltucResCompleteSurvData$cluster))
```

```
##
## FALSE
##    65
```

```
# Check composition of intestinal by clin diag
table(int_ehpcbiltucResCompleteSurvData[ int_ehpcbiltucResCompleteSurvData$c
luster == "intestinal" ,]$clin_diag)
```

```
##
## Ductal pancreatic adenocarcinoma              Ampullary carcinoma
##                                1                                3
##          Distal bile duct cancer               Gallbladder cancer
##                                1                                1
##     Perihilar cholangiocarcinoma  Intrahepatic cholangiocarcinoma
##                                1                                1
```

```
# drop unused cluster levels
int_ehpcbiltucResCompleteSurvData$cluster <- droplevels(int_ehpcbiltucResCom
pleteSurvData$cluster)

#   -Resected -tuc patients, by clin_diag
int_ehpcbiltuc_res_PcbilSurvFit <- npsurv(formula = Surv(surv_time, death_ob
served) ~ cluster, data = int_ehpcbiltucResCompleteSurvData)
print(int_ehpcbiltuc_res_PcbilSurvFit, print.rmean = TRUE)
```

```
## Call: npsurv(formula = Surv(surv_time, death_observed) ~ cluster, data =
int_ehpcbiltucResCompleteSurvData)
##
##                                        records n.max n.start events *rmean
## cluster=extrahepatic pancreatobiliary      57    57      57     49   33.2
## cluster=intestinal                          8     8       8      4   70.8
##                                        *se(rmean) median 0.95LCL 0.95UCL
## cluster=extrahepatic pancreatobiliary       4.24   23.6    18.4    32.7
## cluster=intestinal                         14.45   54.0    31.0      NA
##     * restricted mean with upper limit =  111
```

```
# KM
sampleSize <- nrow(int_ehpcbiltucResCompleteSurvData)
survplot(int_ehpcbiltuc_res_PcbilSurvFit, conf = "none", label.curves = FALS
E, time.inc = 10, n.risk = TRUE,
         col = c("red", "blueviolet"), lty = 1, lwd = 3, cex.n.risk = 0.8,
         xlab="", ylab = "Survival probability", y.n.risk = -0.2, adj.n.ris
k = 0)
mtext("Months", side=1, line=2)
title(main=paste("Overall survival extrahepatic pancreatobiliary vs. intesti
nal\n(n = ", sampleSize,")", sep = ""))
```

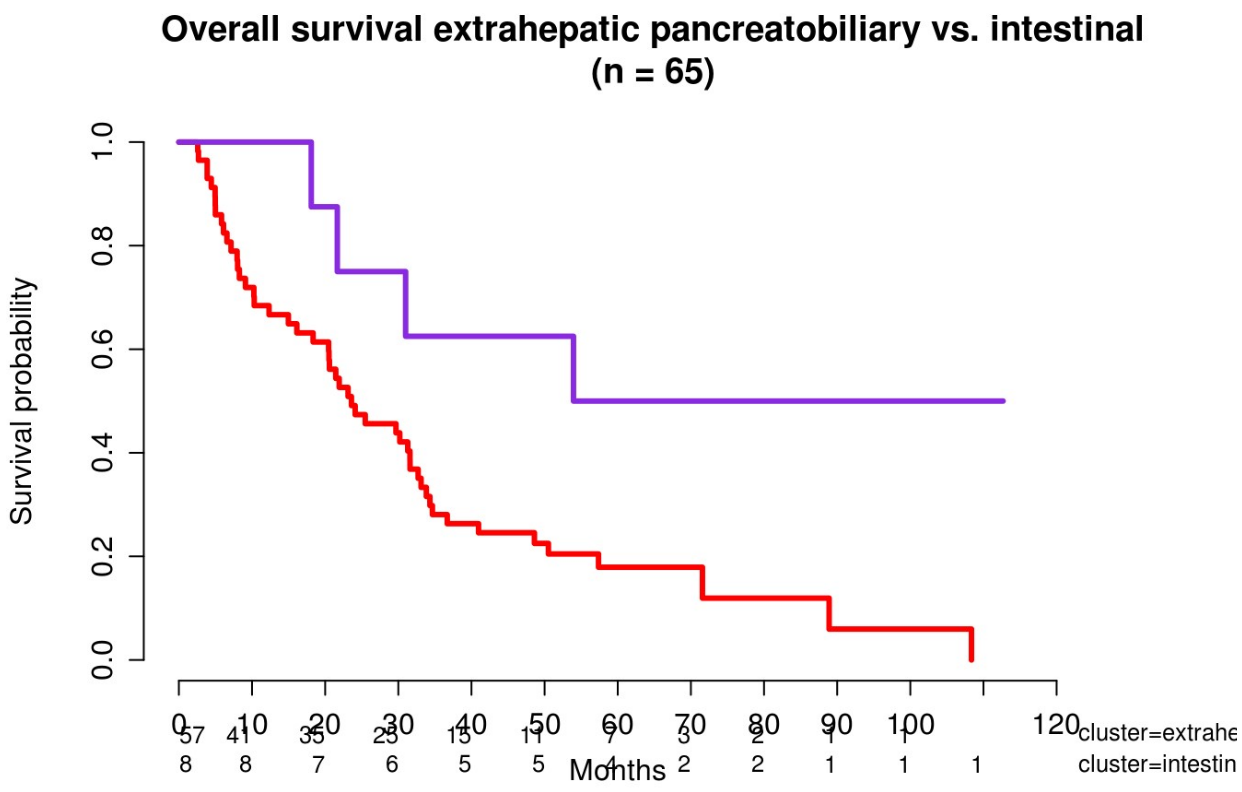


```
# Univariate:
coxPcbilIntUniv <- coxph(Surv(surv_time, death_observed) ~ cluster, data = i
nt_ehpcbiltucResCompleteSurvData)
summary(coxPcbilIntUniv)
```

```
## Call:
## coxph(formula = Surv(surv_time, death_observed) ~ cluster, data = int_ehp
cbiltucResCompleteSurvData)
##
##   n= 65, number of events= 53
##
##                       coef exp(coef) se(coef)      z Pr(>|z|)
## clusterintestinal -1.1207    0.3261   0.5252 -2.134   0.0329 *
## ---
## Signif. codes:  0 '***' 0.001 '**' 0.01 '*' 0.05 '.' 0.1 ' ' 1
##
##                    exp(coef) exp(-coef) lower .95 upper .95
## clusterintestinal    0.3261      3.067    0.1165    0.9128
##
## Concordance= 0.554  (se = 0.028 )
## Rsquare= 0.091   (max possible= 0.997 )
## Likelihood ratio test= 6.19  on 1 df,   p=0.01286
## Wald test            = 4.55  on 1 df,   p=0.03287
## Score (logrank) test = 5.02  on 1 df,   p=0.02506
```

```
# Test the Proportional Hazards Assumption of a Cox Regression
cox_pha <- cox.zph(coxPcbilIntUniv)
print(cox_pha)
```

```
##                       rho   chisq     p
## clusterintestinal 0.0124 0.00822 0.928
```

```
plot(cox_pha)
```

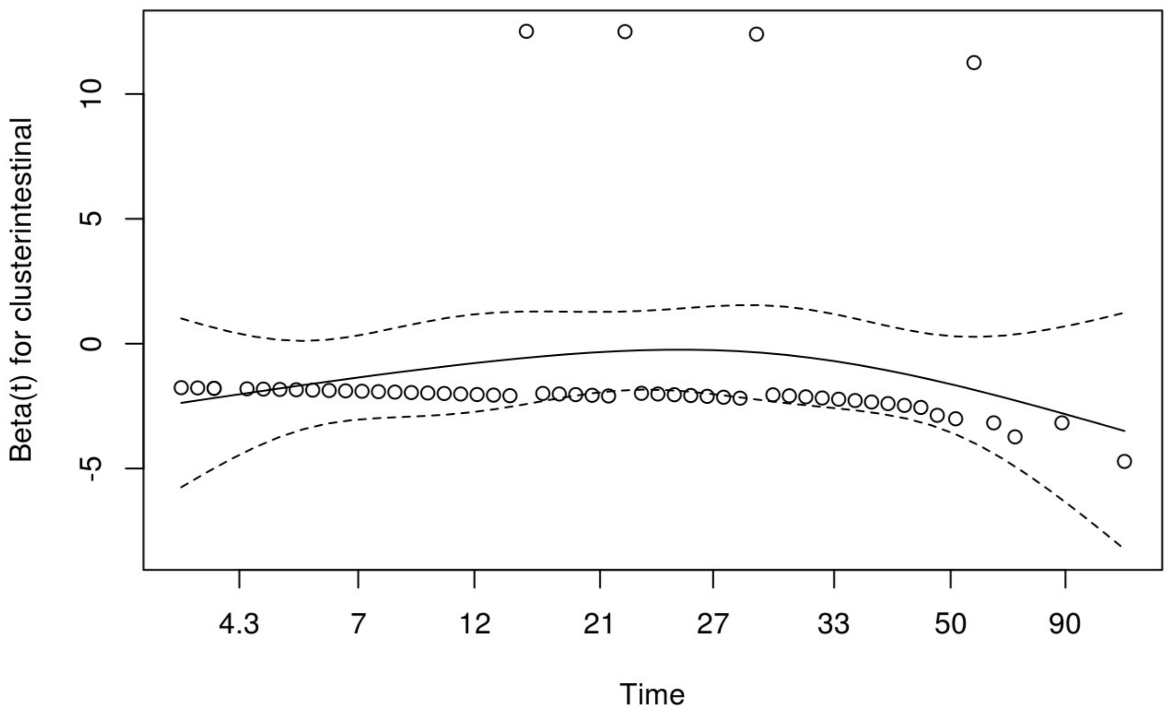


```
# Multivariate Cox proportional hazards regression model.
coxPcbilIntMultiv <- coxph(Surv(surv_time, death_observed) ~ cluster + p_t
+ p_n, data = int_ehpcbiltucResCompleteSurvData)
```

```
## Warning in fitter(X, Y, strats, offset, init, control, weights = weights,
:
## Loglik converged before variable 5 ; beta may be infinite.
```

```
summary(coxPcbilIntMultiv)
```

```
## Call:
## coxph(formula = Surv(surv_time, death_observed) ~ cluster + p_t +
##     p_n, data = int_ehpcbiltucResCompleteSurvData)
##
##   n= 61, number of events= 50
##    (4 observations deleted due to missingness)
##
##                         coef  exp(coef)   se(coef)      z Pr(>|z|)
## clusterintestinal -1.665e+00  1.892e-01  6.782e-01 -2.455  0.01409 *
## p_t1               4.913e-01  1.634e+00  6.854e-01  0.717  0.47351
## p_t2              -6.248e-02  9.394e-01  3.522e-01 -0.177  0.85918
## p_t4               1.616e+00  5.031e+00  5.289e-01  3.055  0.00225 **
## p_tis             -1.688e+01  4.661e-08  4.341e+03 -0.004  0.99690
## p_n0              -6.016e-01  5.479e-01  3.870e-01 -1.554  0.12009
## ---
## Signif. codes:  0 '***' 0.001 '**' 0.01 '*' 0.05 '.' 0.1 ' ' 1
##
##                   exp(coef) exp(-coef) lower .95 upper .95
## clusterintestinal 1.892e-01  5.285e+00   0.05008    0.7149
## p_t1              1.634e+00  6.118e-01   0.42653    6.2628
## p_t2              9.394e-01  1.064e+00   0.47108    1.8734
## p_t4              5.031e+00  1.987e-01   1.78431   14.1879
## p_tis             4.661e-08  2.145e+07   0.00000       Inf
## p_n0              5.479e-01  1.825e+00   0.25662    1.1699
##
## Concordance= 0.67  (se = 0.044 )
## Rsquare= 0.277   (max possible= 0.996 )
## Likelihood ratio test= 19.81  on 6 df,   p=0.002991
## Wald test            = 15.09  on 6 df,   p=0.01953
## Score (logrank) test = 17.9  on 6 df,   p=0.00649
```

```
# Test the Proportional Hazards Assumption of a Cox Regression
cox_pha <- cox.zph(coxPcbilIntMultiv)
print(cox_pha)
```

```
##                        rho    chisq      p
## clusterintestinal  0.00691 3.75e-03 0.9512
## p_t1              -0.03004 4.14e-02 0.8387
## p_t2              -0.23768 2.84e+00 0.0919
## p_t4              -0.10888 7.99e-01 0.3714
## p_tis             -0.49216 7.95e-08 0.9998
## p_n0               0.27800 3.52e+00 0.0608
## GLOBAL                  NA 7.65e+00 0.2649
```

```
plot(cox_pha)
```

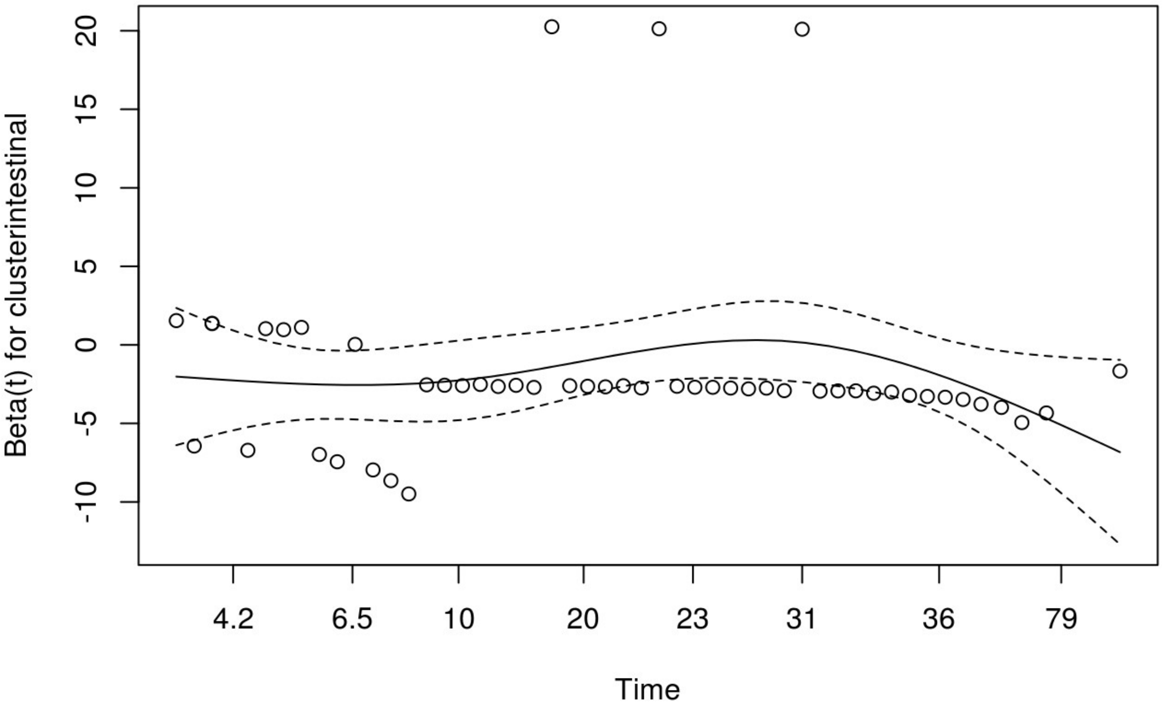
Beta(t) for clusterintestinal
20
15
10
5
0
-5
-10
4.2
6.5
10
20
23
31
36
79
Time

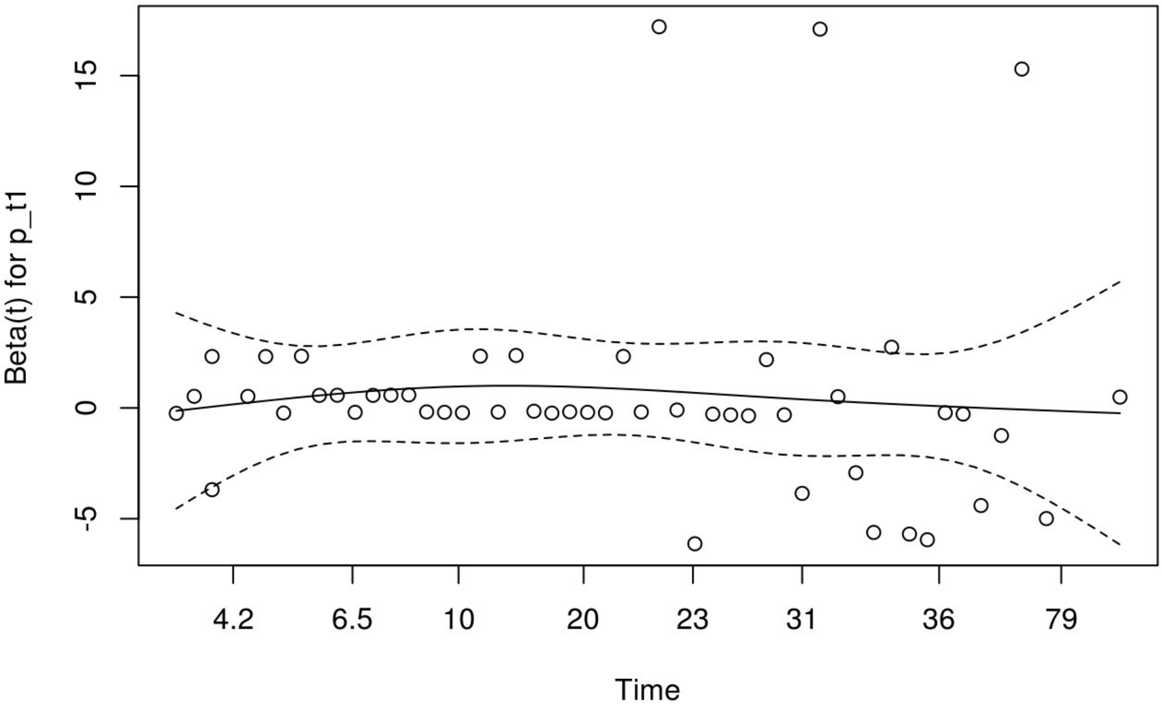
Beta(t) for p_t1
15
10
5
0
-5
4.2
6.5
10
20
23
31
36
79
Time

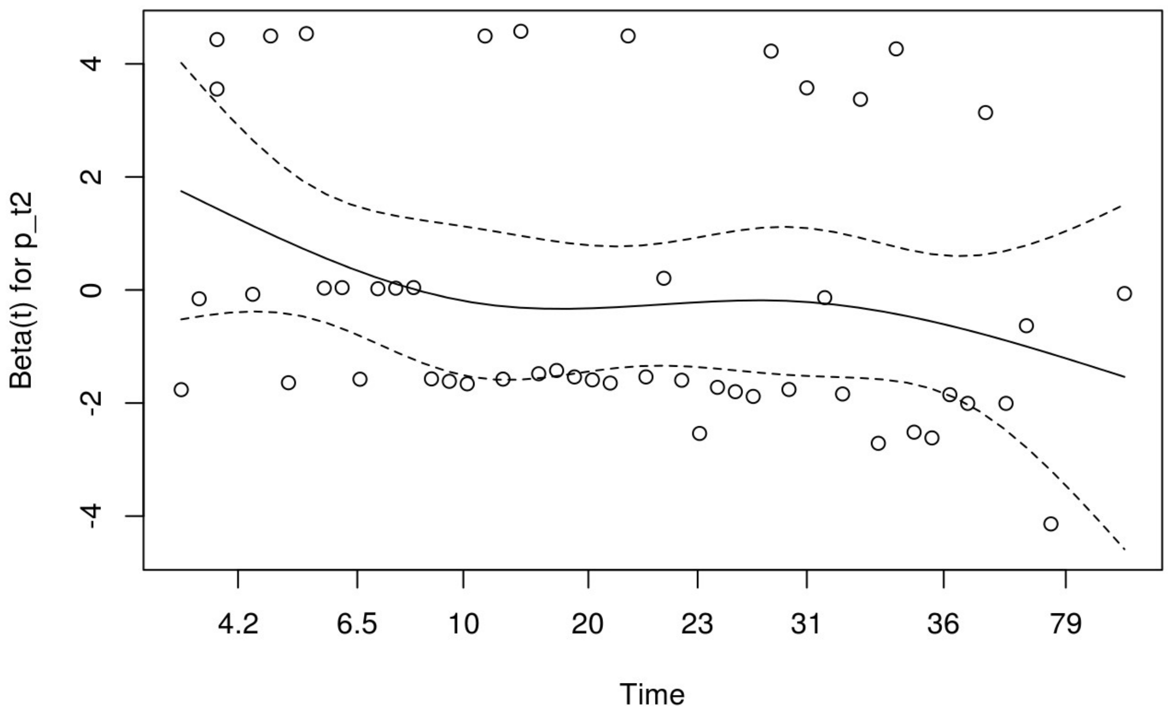

Beta(t) for p_t2
4
2
0
-2
-4
4.2
6.5
10
20
23
31
36
79
Time

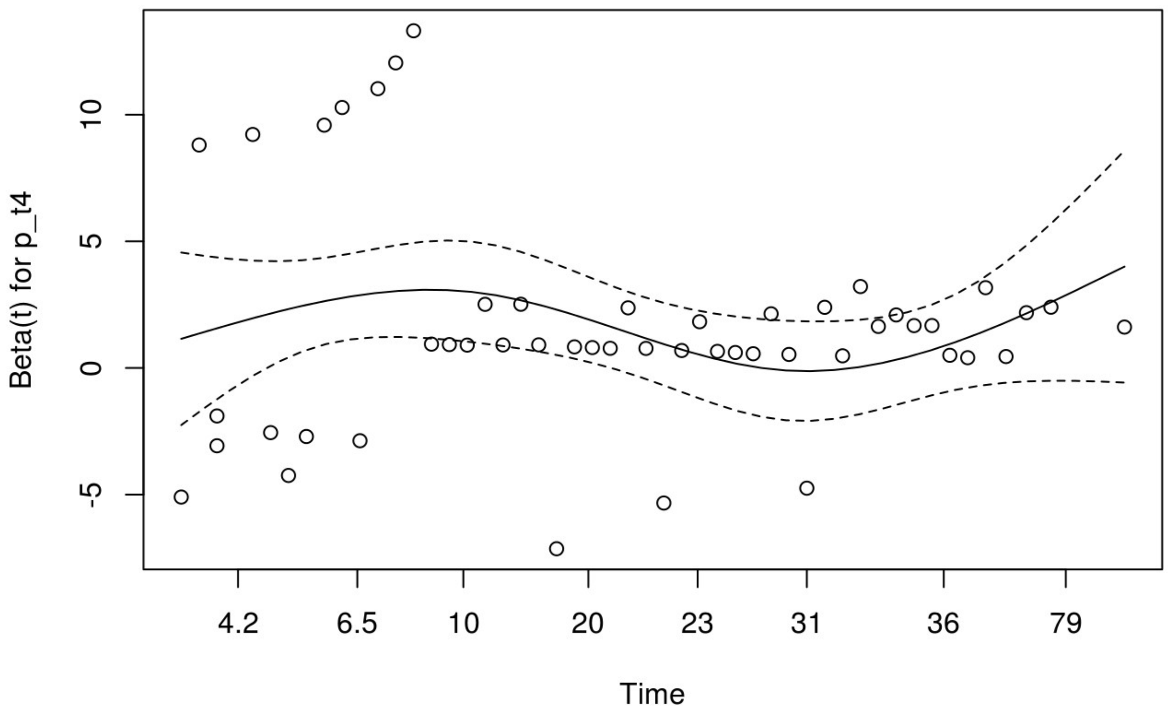

Beta(t) for p_t4
10
5
0
-5
4.2
6.5
10
20
23
31
36
79
Time

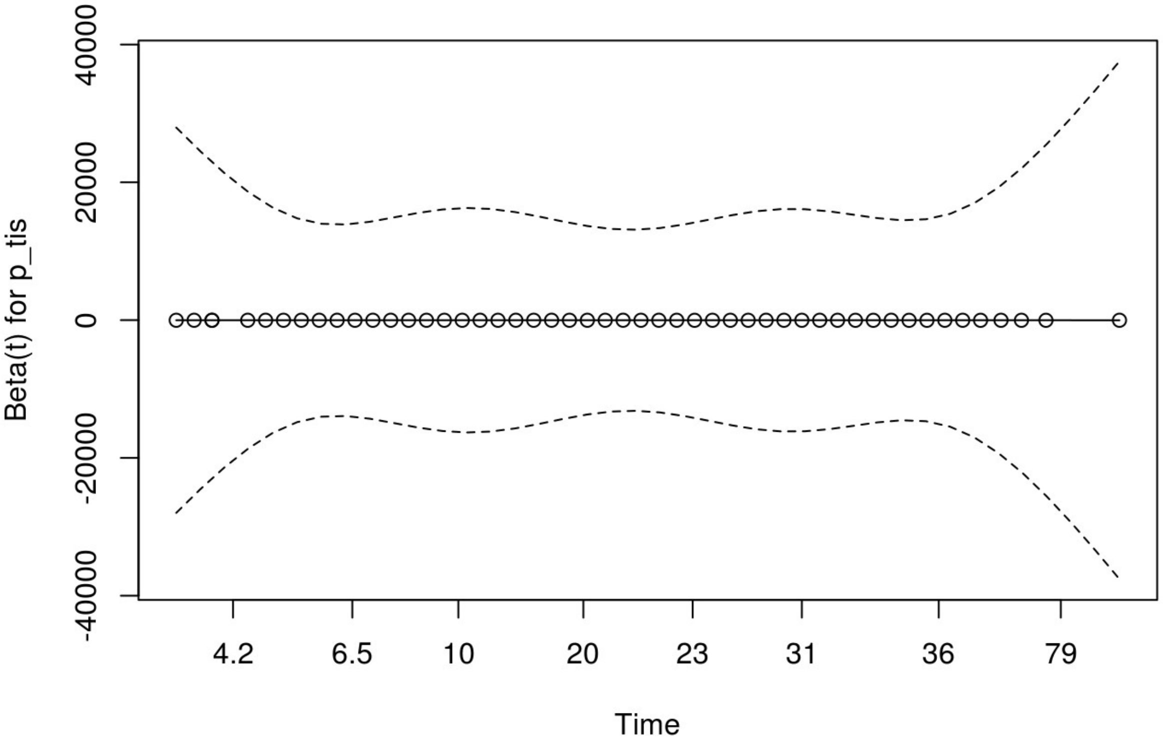

Beta(t) for p_tis
40000
20000
0
-20000
-40000
4.2
6.5
10
20
23
31
36
79
Time

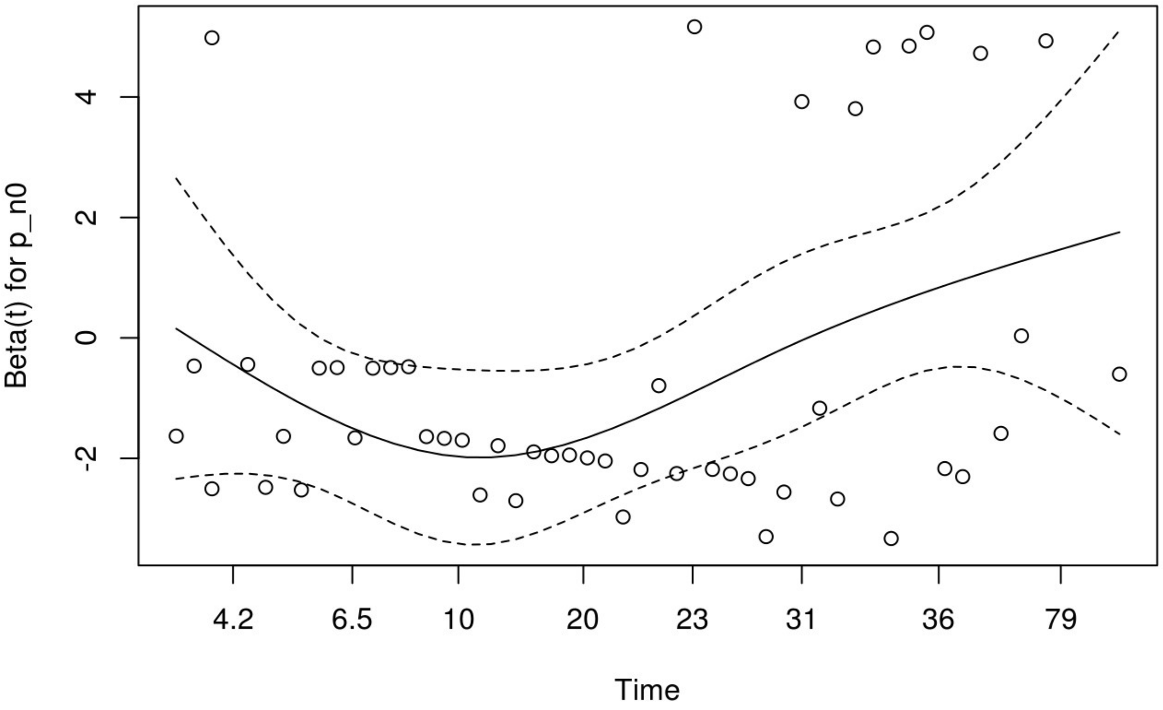

Beta(t) for p_n0
4
2
0
-2
4.2
6.5
10
20
23
31
36
79
Time

Resected patients with intrahepatic cholangiocarcinoma (anatomical-based diagnosis): extrahepatic pancreatobiliary vs intrahepatic cholangiocarcinoma immunohistochemical types

```
# Obtain and attach data set
iccResCompleteSurvData <- subset(resCompleteSurvData, (clin_diag == "Intrahe
patic cholangiocarcinoma" & (cluster == "extrahepatic pancreatobiliary" | cl
uster == "intrahepatic cholangiocarcinoma")))

# Missing data check
table(is.na(iccResCompleteSurvData$cluster))
```

```
##
## FALSE
##    15
```

```
# drop unused levels
iccResCompleteSurvData$cluster <- droplevels(iccResCompleteSurvData$cluster)
iccResCompleteSurvData$p_t <- droplevels(iccResCompleteSurvData$p_t)

#   -Resected ICC patients, by cluster
sampleSize <- nrow(iccResCompleteSurvData)
cluster_icc_res_PcbilSurvFit <- npsurv(formula = Surv(surv_time, death_obser
ved) ~ cluster, data = iccResCompleteSurvData)
print(cluster_icc_res_PcbilSurvFit, print.rmean = TRUE)
```

```
## Call: npsurv(formula = Surv(surv_time, death_observed) ~ cluster, data =
iccResCompleteSurvData)
##
##                                          records n.max n.start events
## cluster=extrahepatic pancreatobiliary          5     5       5      4
## cluster=intrahepatic cholangiocarcinoma       10    10      10      6
##                                          *rmean *se(rmean) median 0.95LCL
## cluster=extrahepatic pancreatobiliary      25.4      12.44   10.3    4.43
## cluster=intrahepatic cholangiocarcinoma    53.1       9.29   78.5   18.74
##                                          0.95UCL
## cluster=extrahepatic pancreatobiliary         NA
## cluster=intrahepatic cholangiocarcinoma       NA
##     * restricted mean with upper limit =  76.6
```

```
survplot(cluster_icc_res_PcbilSurvFit, conf = "none", label.curves = FALSE,
time.inc = 10, n.risk = TRUE,
         col = c("red", "blue"), lty = 1, lwd = 3, cex.n.risk = 0.8,
         xlab="", ylab = "Survival probability", y.n.risk = -0.2, adj.n.ris
k = 0)
mtext("Months", side=1, line=2)
title(main=paste("Overall survival for intrahepatic cholangiocarcinoma\nby i
mmunohistochemical type\n(n = ", sampleSize,")", sep = ""))
```

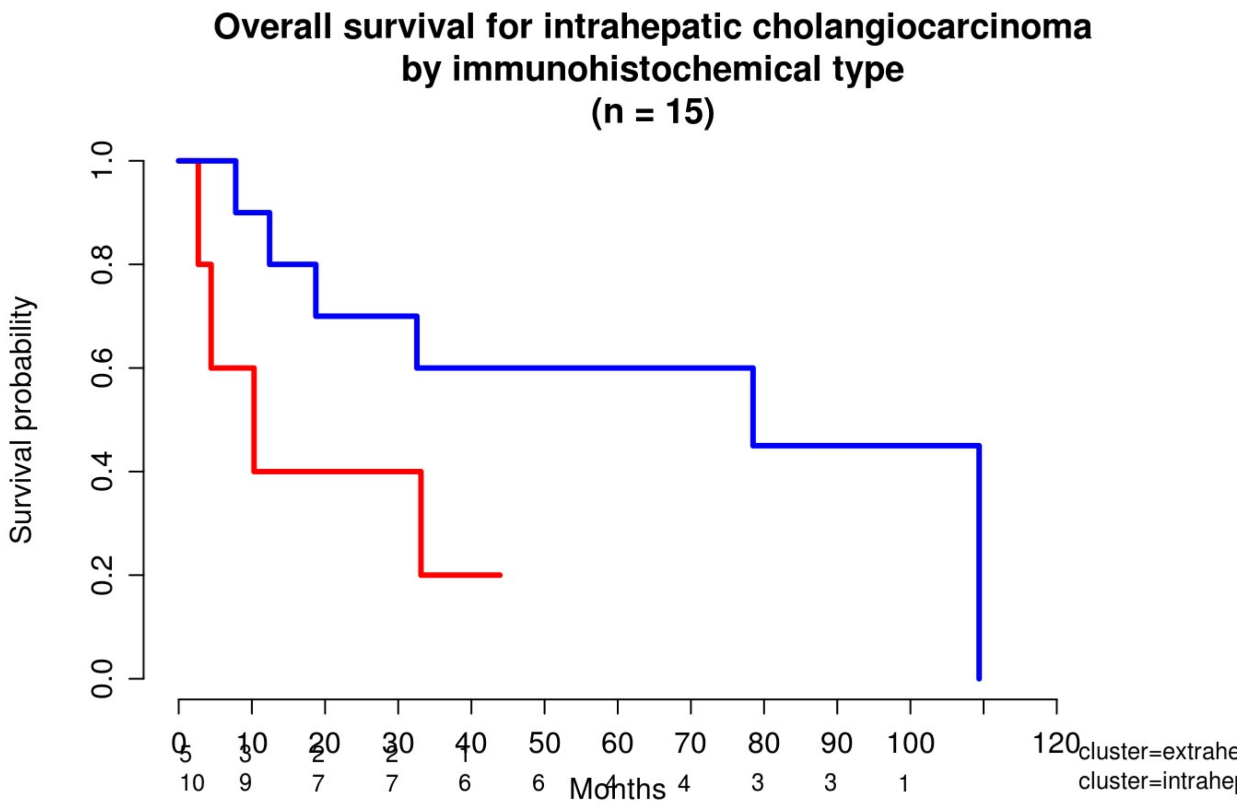


```
survdiff(Surv(surv_time, death_observed) ~ cluster, data = iccResCompleteSur
vData, rho = 0)
```

```
## Call:
## survdiff(formula = Surv(surv_time, death_observed) ~ cluster,
##     data = iccResCompleteSurvData, rho = 0)
##
##                                            N Observed Expected (O-E)^2/E
## cluster=extrahepatic pancreatobiliary      5        4     1.95      2.14
## cluster=intrahepatic cholangiocarcinoma 10        6     8.05      0.52
##                                         (O-E)^2/V
## cluster=extrahepatic pancreatobiliary        2.87
## cluster=intrahepatic cholangiocarcinoma      2.87
##
##  Chisq= 2.9  on 1 degrees of freedom, p= 0.0904
```

```r
# Univariate: Log-rank or Mantel-Haenszel test
coxICCUniv <- coxph(Surv(surv_time, death_observed) ~ cluster, data = iccRes
CompleteSurvData)
summary(coxICCUniv)
```

```
## Call:
## coxph(formula = Surv(surv_time, death_observed) ~ cluster, data = iccResC
ompleteSurvData)
##
##   n= 15, number of events= 10
##
##                                            coef exp(coef) se(coef)      z
## clusterintrahepatic cholangiocarcinoma -1.1446    0.3183   0.7122 -1.607
##                                        Pr(>|z|)
## clusterintrahepatic cholangiocarcinoma    0.108
##
##                                        exp(coef) exp(-coef) lower .95
## clusterintrahepatic cholangiocarcinoma    0.3183      3.141   0.07883
##                                        upper .95
## clusterintrahepatic cholangiocarcinoma     1.286
##
## Concordance= 0.649  (se = 0.083 )
## Rsquare= 0.151   (max possible= 0.937 )
## Likelihood ratio test= 2.46  on 1 df,   p=0.1169
## Wald test            = 2.58  on 1 df,   p=0.108
## Score (logrank) test = 2.87  on 1 df,   p=0.09043
```

```r
# Test the Proportional Hazards Assumption of a Cox Regression
cox_pha <- cox.zph(coxICCUniv)
print(cox_pha)
```

```
##                                          rho chisq     p
## clusterintrahepatic cholangiocarcinoma 0.191 0.305 0.581
```

```r
plot(cox_pha)
```

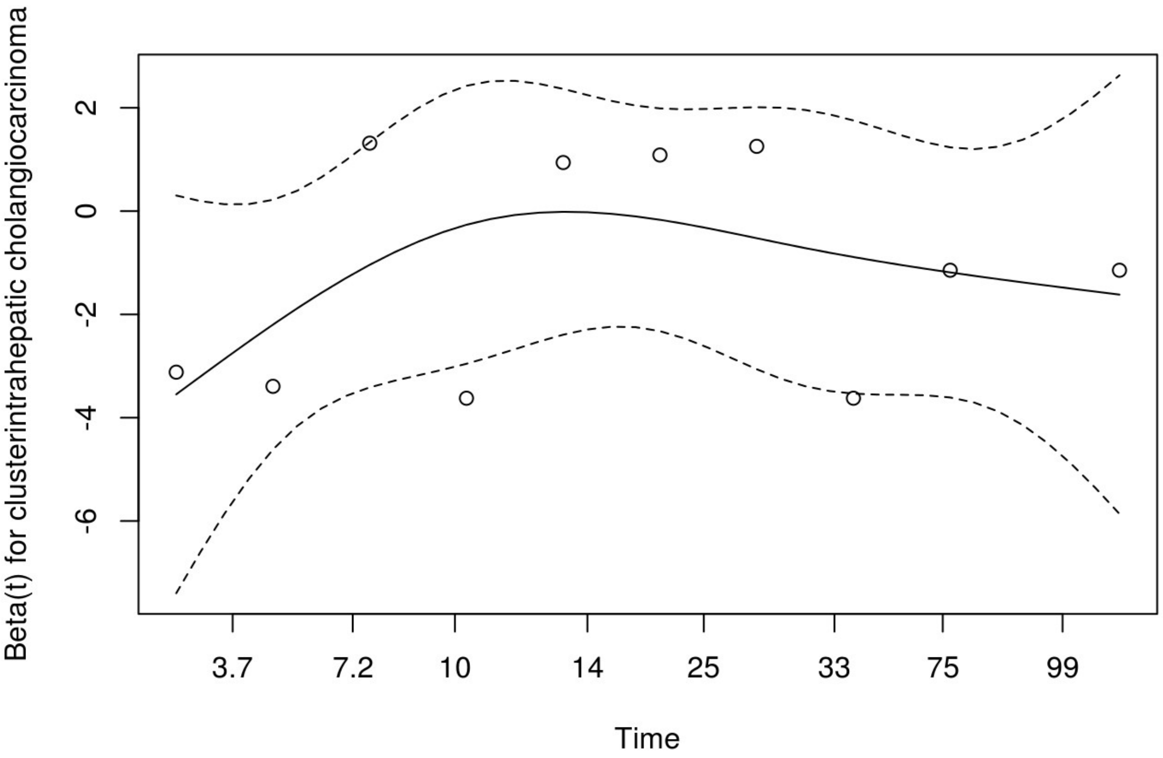


```
# Multivariate Cox proportional hazards regression model.
coxICCMultiv <- coxph(Surv(surv_time, death_observed) ~ cluster + p_t + p_
n, data = iccResCompleteSurvData)
```

```
## Warning in fitter(X, Y, strats, offset, init, control, weights = weights,
:
## Ran out of iterations and did not converge
```

```
summary(coxICCMultiv)
```

```
## Call:
## coxph(formula = Surv(surv_time, death_observed) ~ cluster + p_t +
##     p_n, data = iccResCompleteSurvData)
##
##   n= 11, number of events= 8
##    (4 observations deleted due to missingness)
##
##                                               coef  exp(coef)   se(coef)
## clusterintrahepatic cholangiocarcinoma  2.106e+01  1.397e+09  1.782e+04
## p_t1                                   -1.648e+00  1.925e-01  1.782e+04
## p_t2                                   -9.745e-01  3.774e-01  1.782e+04
## p_t4                                    3.903e+01  8.950e+16  1.898e+04
## p_n0                                   -2.150e+01  4.591e-10  1.782e+04
##                                             z Pr(>|z|)
## clusterintrahepatic cholangiocarcinoma  0.001    0.999
## p_t1                                    0.000    1.000
## p_t2                                    0.000    1.000
## p_t4                                    0.002    0.998
## p_n0                                   -0.001    0.999
##
##                                        exp(coef) exp(-coef) lower .95
## clusterintrahepatic cholangiocarcinoma 1.397e+09  7.156e-10         0
## p_t1                                   1.925e-01  5.194e+00         0
## p_t2                                   3.774e-01  2.650e+00         0
## p_t4                                   8.950e+16  1.117e-17         0
## p_n0                                   4.591e-10  2.178e+09         0
##                                        upper .95
## clusterintrahepatic cholangiocarcinoma       Inf
## p_t1                                         Inf
## p_t2                                         Inf
## p_t4                                         Inf
## p_n0                                         Inf
##
## Concordance= 0.865  (se = 0.119 )
## Rsquare= 0.75   (max possible= 0.943 )
## Likelihood ratio test= 15.26  on 5 df,   p=0.009309
## Wald test            = 0.82  on 5 df,   p=0.9754
## Score (logrank) test = 16.8  on 5 df,   p=0.0049
```

```
# Test the Proportional Hazards Assumption of a Cox Regression
#cox_pha <- cox.zph(coxICCMultiv)
#print(cox_pha)
#plot(cox_pha)
```

– End of survival analysis resected patients –
