# Supplementary material for: Immunohistochemical Typing of Adenocarcinomas of the Pancreatobiliary System Improves Diagnosis and Prognostic Stratification
Source: PLoS One. 2016 Nov 9;11(11):e0166067. doi: 10.1371/journal.pone.0166067 (PMC5102456; doi:10.1371/journal.pone.0166067)
Supplement: S3 Table — (PDF) [file pone.0166067.s009.pdf]

Supplementary Table 3: Machine learning classification/internal validation

	original								reduced							
	No. instances	No. attributes	metric	Classifier					No. instances	No. attributes	metric	Classifer				
				Bayes net	Simple Logistic	SMO	Random Tree	**Average**				Bayes net	Simple Logistic	SMO	Random Tree	**Average**
extrahepatic-pcbil-tuc vs intrahepatic-ccc-tuc	281	27	Av. TP	0,947	0,950	0,957	0,918	**0,943**	281	11	Av. TP	0,940	0,947	0,947	0,900	**0,934**
			Av. FP	0,072	0,071	0,068	0,120	**0,083**			Av. FP	0,104	0,072	0,079	0,163	**0,105**
			Precision A	0,965	0,965	0,965	0,940	**0,959**			Precision A	0,946	0,965	0,960	0,917	**0,947**
			Precision B	0,904	0,915	0,938	0,864	**0,905**			Precision B	0,922	0,904	0,914	0,855	**0,899**
			Recall A	0,960	0,965	0,975	0,945	**0,961**			Recall A	0,970	0,960	0,965	0,945	**0,960**
			Recall B	0,915	0,915	0,915	0,854	**0,900**			Recall B	0,866	0,915	0,902	0,793	**0,869**
			F-measure A	0,962	0,965	0,970	0,942	**0,960**			F-measure	0,958	0,962	0,962	0,931	**0,953**
			F-measure B	0,909	0,915	0,926	0,859	**0,902**			F-measure	0,893	0,909	0,908	0,823	**0,883**
			ROC	0,982	0,981	0,945	0,942	**0,963**			ROC	0,983	0,982	0,934	0,936	**0,959**
extrahepatic-pcbil-tuc vs?intestinal-tuc	223	27	Av. TP	0,951	0,973	0,978	0,933	**0,959**	223	7	Av. TP	0,955	0,964	0,973	0,951	**0,961**
			Av. FP	0,262	0,186	0,149	0,265	**0,216**			Av. FP	0,262	0,188	0,223	0,262	**0,234**
			Precision A	0,965	0,975	0,980	0,965	**0,971**			Precision A	0,966	0,975	0,971	0,965	**0,969**
			Precision B	0,810	0,950	0,952	0,680	**0,848**			Precision B	0,850	0,864	1,000	0,810	**0,881**
			Recall A	0,980	0,995	0,995	0,960	**0,983**			Recall A	0,985	0,985	1,000	0,980	**0,988**
			Recall B	0,708	0,792	0,833	0,708	**0,760**			Recall B	0,708	0,792	0,750	0,708	**0,740**
			F-measure A	0,973	0,985	0,988	0,962	**0,977**			F-measure	0,975	0,980	0,985	0,973	**0,978**
			F-measure B	0,756	0,864	0,889	0,694	**0,801**			F-measure	0,773	0,826	0,857	0,756	**0,803**
			ROC	0,974	0,966	0,914	0,829	**0,921**			ROC	0,982	0,951	0,875	0,844	**0,913**
intrahepatic-ccc-tuc vs? hcc-tuc	150	27	Av. TP	0,987	0,987	1,000	0,960	**0,984**	150	12	Av. TP	0,980	0,987	0,993	0,973	**0,983**
			Av. FP	0,016	0,016	0,000	0,043	**0,019**			Av. FP	0,024	0,016	0,008	0,025	**0,018**
			Precision A	0,976	0,976	1,000	0,952	**0,976**			Precision A	0,965	0,976	0,988	0,988	**0,979**
			Precision B	1,000	1,000	1,000	0,970	**0,993**			Precision B	1,000	1,000	1,000	0,957	**0,989**
			Recall A	1,000	1,000	1,000	0,976	**0,994**			Recall A	1,000	1,000	1,000	0,963	**0,991**
			Recall B	0,971	0,971	1,000	0,941	**0,971**			Recall B	0,956	0,971	0,985	0,985	**0,974**
			F-measure A	0,988	0,988	1,000	0,964	**0,985**			F-measure	0,982	0,988	0,994	0,975	**0,985**
			F-measure B	0,985	0,985	1,000	0,955	**0,981**			F-measure	0,977	0,985	0,993	0,971	**0,982**
			ROC	1,000	0,998	1,000	0,971	**0,992**			ROC	1,000	0,999	0,993	0,974	**0,992**

Clinical

Bayes net

Accuracy 62,84%

Class	TP Rate	FP Rate	Precision	Recall	F-Measure	ROC Area
Pancreas Ac	0,827	0,263	0,618	0,827	0,708	0,841
Hepatocellular Cancer	0,91	0,018	0,922	0,91	0,916	0,99
Gallbladder Ac	0	0,051	0	0	0	0,663
Intrahepatic Cholangiocarcinoma	0,684	0,116	0,65	0,684	0,667	0,854
Ampulla Ac	0,167	0,026	0,286	0,167	0,211	0,837
Perihilary Ac	0	0,021	0	0	0	0,695
Distal Bile Duct Ac	0	0,005	0	0	0	0,751
Weighted Avg.	0,628	0,128	0,559	0,628	0,587	0,845

Simple Logic

Accuracy 64,79%

Class	TP Rate	FP Rate	Precision	Recall	F-Measure	ROC Area
Pancreas Ac	0,835	0,27	0,614	0,835	0,707	0,849
Hepatocellular Cancer	0,936	0,021	0,913	0,936	0,924	0,993
Gallbladder Ac	0,081	0,046	0,15	0,081	0,105	0,685
Intrahepatic Cholangiocarcinoma	0,673	0,122	0,635	0,673	0,653	0,843
Ampulla Ac	0,25	0,008	0,667	0,25	0,364	0,855
Perihilary Ac	0,037	0,016	0,143	0,037	0,059	0,71
Distal Bile Duct Ac	0	0	0	0	0	0,631
Weighted Avg.	0,648	0,131	0,597	0,648	0,608	0,848

SVM-SMO

Accuracy 65,04%

Class	TP Rate	FP Rate	Precision	Recall	F-Measure	ROC Area
Pancreas Ac	0,871	0,311	0,59	0,871	0,703	0,807
Hepatocellular Cancer	0,949	0,015	0,937	0,949	0,943	0,984
Gallbladder Ac	0,162	0,043	0,273	0,162	0,203	0,532
Intrahepatic Cholangiocarcinoma	0,643	0,1	0,67	0,643	0,656	0,75
Ampulla Ac	0,083	0,008	0,4	0,083	0,138	0,783
Perihilary Ac	0	0,01	0	0	0	0,537
Distal Bile Duct Ac	0	0	0	0	0	0,568
Weighted Avg.	0,65	0,138	0,588	0,65	0,603	0,78

Random forest

Accuracy 64,06%

Class	TP Rate	FP Rate	Precision	Recall	F-Measure	ROC Area
Pancreas Ac	0,871	0,326	0,579	0,871	0,695	0,83
Hepatocellular Cancer	0,949	0,03	0,881	0,949	0,914	0,987
Gallbladder Ac	0,054	0,024	0,182	0,054	0,083	0,534
Intrahepatic Cholangiocarcinoma	0,622	0,106	0,649	0,622	0,635	0,812
Ampulla Ac	0,125	0,01	0,429	0,125	0,194	0,731
Perihilary Ac	0,037	0,008	0,25	0,037	0,065	0,668
Distal Bile Duct Ac	0	0	0	0	0	0,433
Weighted Avg.	0,641	0,145	0,578	0,641	0,586	0,807

Classifier-average

Accuracy 64,18%

Class	TP Rate	FP Rate	Precision	Recall	F-Measure	ROC Area
Pancreas Ac	0,851	0,2925	0,60025	0,851	0,70325	0,83175
Hepatocellular Cancer	0,936	0,021	0,91325	0,936	0,92425	0,9885
Gallbladder Ac	0,07425	0,041	0,15125	0,07425	0,09775	0,6035
Intrahepatic Cholangiocarcinoma	0,6555	0,111	0,651	0,6555	0,65275	0,81475
Ampulla Ac	0,15625	0,013	0,4455	0,15625	0,22675	0,8015
Perihilary Ac	0,0185	0,01375	0,09825	0,0185	0,031	0,6525
Distal Bile Duct Ac	0	0,00125	0	0	0	0,59575
Weighted Avg.	0,64175	0,1355	0,5805	0,64175	0,596	0,82

Clustered

Bayes Net

Accuracy 93,83%

Class	TP Rate	FP Rate	Precision	Recall	F-Measure	ROC Area
extrahepatic-pcbil-tuc	0,955	0,075	0,936	0,955	0,945	0,98
hcc-tuc	0,985	0	1	0,985	0,993	1
intrahepatic-ccc-tuc	0,902	0,021	0,925	0,902	0,914	0,988
intestinal-tuc	0,792	0,011	0,826	0,792	0,809	0,976
Weighted Avg.	0,938	0,045	0,938	0,938	0,938	0,985

Simple Logic

Accuracy 94,64%

Class	TP Rate	FP Rate	Precision	Recall	F-Measure	ROC Area
extrahepatic-pcbil-tuc	0,97	0,075	0,937	0,97	0,953	0,987
hcc-tuc	0,985	0	1	0,985	0,993	1
intrahepatic-ccc-tuc	0,915	0,024	0,915	0,915	0,915	0,99
intestinal-tuc	0,75	0	1	0,75	0,857	0,991
Weighted Avg.	0,946	0,045	0,948	0,946	0,946	0,99

SVM-SMO

Accuracy 94,64%

Class	TP Rate	FP Rate	Precision	Recall	F-Measure	ROC Area
extrahepatic-pcbil-tuc	0,955	0,063	0,945	0,955	0,95	0,955
hcc-tuc	1	0	1	1	1	1
intrahepatic-ccc-tuc	0,939	0,021	0,928	0,939	0,933	0,972
intestinal-tuc	0,75	0,009	0,857	0,75	0,8	0,945
Weighted Avg.	0,946	0,039	0,946	0,946	0,946	0,966

Random forest

Accuracy 91,15%

Class	TP Rate	FP Rate	Precision	Recall	F-Measure	ROC Area
extrahepatic-pcbil-tuc	0,975	0,155	0,878	0,975	0,924	0,972
hcc-tuc	0,985	0	1	0,985	0,993	1
intrahepatic-ccc-tuc	0,829	0,017	0,932	0,829	0,877	0,98
intestinal-tuc	0,458	0,003	0,917	0,458	0,611	0,958
Weighted Avg.	0,912	0,087	0,914	0,912	0,906	0,978

Classifier-average

Accuracy 93,48%

Class	TP Rate	FP Rate	Precision	Recall	F-Measure	ROC Area
extrahepatic-pcbil-tuc	0,96375	0,092	0,924	0,96375	0,943	0,9735
hcc-tuc	0,98875	0	1	0,98875	0,99475	1
intrahepatic-ccc-tuc	0,89625	0,02075	0,925	0,89625	0,90975	0,9825
intestinal-tuc	0,6875	0,00575	0,9	0,6875	0,76925	0,9675
Weighted Avg.	0,9355	0,054	0,9365	0,9355	0,934	0,97975

Summary table

Anatomical based diagnosis classes - averaged results over four classifers

Accuracy 64,18%

Class	No. of instances	TP Rate	FP Rate	Precision	Recall	F-Measure	ROC Area
Pancreas Ac	139	0,851	0,2925	0,60025	0,851	0,70325	0,83175
Hepatocellular Cancer	78	0,936	0,021	0,91325	0,936	0,92425	0,9885
Gallbladder Ac	37	0,07425	0,041	0,15125	0,07425	0,09775	0,6035
Intrahepatic Cholangiocarcinoma	98	0,6555	0,111	0,651	0,6555	0,65275	0,81475
Ampulla Ac	24	0,15625	0,013	0,4455	0,15625	0,22675	0,8015
Perihilary Ac	27	0,0185	0,01375	0,09825	0,0185	0,031	0,6525
Distal Bile Duct Ac	6	0	0,00125	0	0	0	0,59575
Weighted Avg.		**0,64175**	**0,1355**	**0,5805**	**0,64175**	**0,596**	**0,82**

Proposed new classification system - averaged results over four classifers

Accuracy 93,48%

Class	No. of instances	TP Rate	FP Rate	Precision	Recall	F-Measure	ROC Area
extrahepatic-pcbil-tuc	199	0,96375	0,092	0,924	0,96375	0,943	0,9735
hcc-tuc	68	0,98875	0	1	0,98875	0,99475	1
intrahepatic-ccc-tuc	82	0,89625	0,02075	0,925	0,89625	0,90975	0,9825
intestinal-tuc	24	0,6875	0,00575	0,9	0,6875	0,76925	0,9675
Weighted Avg.		**0,9355**	**0,054**	**0,9365**	**0,9355**	**0,934**	**0,97975**
